# S100 A16 promotes the progression of osteosarcoma by activating the PI3 K/AKT signaling pathway through ANXA2

**DOI:** 10.1038/s41598-025-05293-6

**Published:** 2025-06-06

**Authors:** Ying-Ying Xiang, Jiang-Hua Liu, Xin Yi, Jing-Yao Luo, Yi Yu, Guo-Liang Yi

**Affiliations:** 1 The First Affiliated Hospital, Department of Orthopaedics, Hengyang Medical School, University of South China, Hengyang, 421001 Hunan China; 2 The First Affiliated Hospital, Department of Metabolism and Endocrinology, Hengyang Medical School, University of South China, Hengyang, 421001 Hunan China; 3 The First Affiliated Hospital, Department of Pain, Hengyang Medical School, University of South China, Hengyang, 421001 Hunan China

**Keywords:** S100 A16, ANXA2, PI3 K/AKT signaling pathway, Osteosarcoma, Tumor progression, Bone cancer, Tumour biomarkers

## Abstract

Osteosarcoma is a common primary malignant bone tumor. S100A16 gene was reported to highly expressed in several tumor tissues while the relationship between S100A16 and osteosarcoma remains less well-understood. This study aimed to investigate the expression characteristics of S100A16 in osteosarcoma and the mechanism by which it promotes osteosarcoma progression. Firstly, by analyzing databases and assessing mRNA and protein level, we found that the expression of S100A16 was significantly promoted in osteosarcoma, as compared with normal tissue. Then transfection techniques were employed to upregulate and downregulate S100A16 in osteosarcoma cells, the results demonstrated that S100A16 can increase osteosarcoma cell viability, migration and invasion capacities, while decline osteosarcoma cell apoptosis. GSEA (gene set enrichment analysis) revealed that increased expression of S100A16 was enriched in the PI3K/AKT pathway. Cellular experiments showed that the S100A16 promoted osteosarcoma progression by activating the PI3K/AKT signaling pathway, and upregulated expression of ANXA2, a crucial protein in occurrence and development of tumors. We also found that overexpression of ANXA2 can restore the decreased levels of p-PI3K and p-AKT induced by S100A16 inhibition, which indicated that S100A16 stimulates PI3K/AKT pathway activation via ANXA2. To sum up, S100A16 can promotes osteosarcoma progression by activating the PI3K/AKT signaling pathway through ANXA2, suggesting that the S100A16/ANXA2 axis may represent a novel therapeutic target for osteosarcoma.

**Supplementary Information:**

The online version contains supplementary material available at 10.1038/s41598-025-05293-6.

Osteosarcoma is a highly malignant primary bone tumor that primarily originates in the metaphyseal regions of long bones, especially the distal femur, proximal tibia, and proximal humerus^1^. This disease predominantly affects adolescents and young adults but can occur at any age, including in the elderly^2^. Symptoms of osteosarcoma typically include localized pain, which may initially be intermittent but progressively intensifies, becoming persistent and particularly noticeable at night. Swelling and functional impairment, such as restricted joint movement, may also be present^3^. The standard treatment regimen for osteosarcoma usually involves a multidisciplinary approach, including neoadjuvant chemotherapy, surgical resection of the tumor and surrounding affected tissues (such as limb-sparing surgery or amputation), followed by adjuvant chemotherapy^4^. With these treatment protocols, the 5-year survival rate for children and young adults with localized disease has reached 78%, whereas it remains at just 20% for patients diagnosed with metastatic or recurrent disease^5^. Moreover, over the past 40 years, there has been no significant improvement in the survival rate of patients without metastasis, nor has there been any substantial improvement for those with metastatic disease^6^. Given the high rates of recurrence and metastatic potential of osteosarcoma, an in-depth exploration of its molecular mechanisms is critical for the development of new therapeutic strategies.

S100A16 is a member of the S100 family of calcium-binding proteins, which comprises 25 known members^7^. The S100A16 gene was initially isolated from an astrocytoma cell line and encodes a small acidic protein consisting of 103 amino acids, with a molecular weight of 11,801.4 Da and an isoelectric point of 6.28^8^. This protein is highly conserved among mammals and is ubiquitously expressed in various human tissues, where it is believed to participate in multiple biological processes such as cell proliferation, differentiation, migration, and inflammatory responses^9^. Several studies have shown that abnormal expression of the S100A16 gene is associated with tumor progression and prognosis. Zhang et al. explored the role of S100A16 in cervical cancer through bioinformatics analysis and found high expression of this gene in cervical cancer^10^. Other research has indicated that S100A16 is overexpressed in pancreatic cancer tissues compared to normal pancreatic tissues, suggesting that elevated levels of this gene may be related to poor prognosis in patients with pancreatic cancer^11^. A study on gastric cancer found that overexpression of S100A16 promotes the proliferation and migration of gastric cancer cells both in vitro and in vivo, indicating that S100A16 is a promising candidate biomarker for early diagnosis and prediction of metastasis in gastric cancer^12^. In contrast, reports regarding the impact of S100A16 on osteosarcoma remain scarce. Given the important role of S100A16 in tumor progression, further investigation of this protein could not only contribute to a better understanding of the molecular mechanisms underlying malignancies such as osteosarcoma but also potentially provide valuable targets for the development of novel therapeutic strategies.

ANXA2, also known as Annexin A2, is a member of the annexin family and is a 36 kDa calcium-dependent phospholipid-binding protein that is ubiquitously expressed in various eukaryotic cells^13^. ANXA2 is involved in numerous pathophysiological processes, including epithelial-mesenchymal transition, fibrinolysis, and cancer resistance^14^. ANXA2 is also thought to play a crucial role in the occurrence and development of tumors, particularly in enhancing the invasiveness and metastatic capability of tumor cells. For instance, high-level expression of ANXA2 has been found to promote the invasion and migration of gastric cancer cells^15^. Researchers have also discovered that overexpression of ANXA2 can promote the proliferation, migration, and invasion of esophageal cancer cells in vitro by activating the MYC-HIF1A-VEGF cascade^16^. Zhou et al. found that ANXA2 is highly expressed in colorectal cancer tissues and cells, with significant implications for the prognosis of colorectal cancer patients; silencing ANXA2 can inhibit M2 macrophage polarization, thereby reducing the proliferation, migration, and invasion capabilities of colorectal cancer cells^17^. In osteosarcoma, studies have shown that high-level expression of ANXA2 promotes the proliferation, migration, and invasion of tumor cells^18^. ANXA2 knockdown re-activated autophagy and attenuated CDDP resistance in osteosarcoma^19^.Therefore, ANXA2 appears to be a key molecule in studying the biological behavior of tumors and may represent a potential target for future therapeutic interventions, warranting further exploration of its mechanisms within osteosarcoma.

In our study, we focused on the role and mechanism of S100A16 in osteosarcoma and found that S100A16 is highly expressed in osteosarcoma samples, with its expression levels closely associated with the proliferation, migration, and invasion capabilities of osteosarcoma cells. Further research indicates that S100A16 activates the PI3K/AKT signaling pathway by regulating the expression of ANXA2, thereby promoting the development of osteosarcoma. These findings reveal the therapeutic potential of targeting S100A16 for the treatment of osteosarcoma.

## Results

### The expression level of S100 A16 is significantly up-regulated in osteosarcoma

**Fig. 1:**
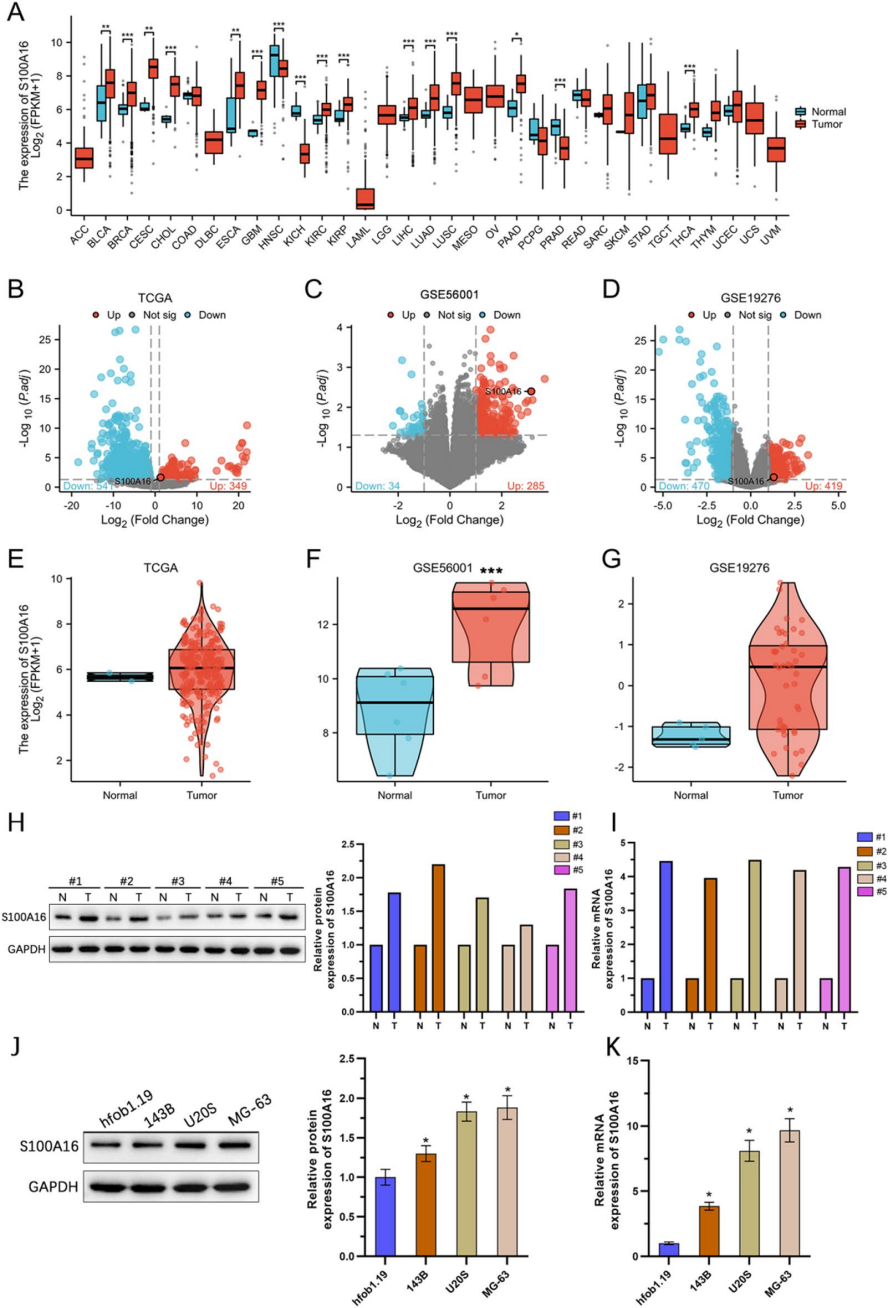
Upregulation of S100 A16 in osteosarcoma. (**A**) Analysis of S100 A16 expression across various malignancies using data from TCGA. (**B**) Differential expression gene analysis of osteosarcoma using TCGA-OS data, visualized with a volcano plot. (**C**, **D**) Differential expression gene analysis of osteosarcoma using datasets GSE56001 and GSE19276 from the GEO database, illustrated with volcano plots. (**E**) Comparison of S100 A16 mRNA expression levels between osteosarcoma and healthy tissues using TCGA-OS data. (**F**, **G**) Comparison of S100 A16 mRNA expression levels between osteosarcoma and healthy tissues using GEO datasets GSE56001 and GSE19276. (**H**, **I**) Assessment of S100 A16 levels in osteosarcoma samples and adjacent normal tissues by western blotting and qRT-PCR (*n* = 5). (J) Evaluation of S100 A16 levels in various cell lines through western blotting and qRT-PCR (*n* = 3). *p < 0.05; **p < 0.01; ***p < 0.001.

In our study, we investigated the expression levels of S100 A16 in osteosarcoma. Bioinformatics analysis revealed that S100 A16 was upregulated in multiple cancers (Fig. 1A). Differential expression analysis using the TCGA and GEO databases (GSE56001 and GSE19276) identified significant upregulation of S100 A16 in osteosarcoma, as shown by volcano plots (Figs. 1B-D). Unpaired sample tests confirmed that S100 A16 expression was significantly higher in tumor tissues compared to normal tissues (Figs. 1E-G). WB and qRT-PCR analyses of human samples further validated these findings, showing elevated S100 A16 expression in tumor tissues (Figs. 1H, I). Additionally, WB and qRT-PCR analyses of S100 A16 expression in hfOB1.19 (normal osteoblasts), 143B, U2OS, and MG-63 cell lines demonstrated that S100 A16 was up-regulated in the osteosarcoma cell lines, with the highest expression levels observed in U2OS and MG-63 cells (Figs. 1J, K). These results collectively indicate that S100 A16 is significantly upregulated in osteosarcoma, and U2OS and MG-63 cell lines were selected for subsequent experiments due to their high S100 A16 expression levels.

### Upregulation of S100 A16 enhances osteosarcoma cell proliferation, migration, and invasion

**Fig. 2:**
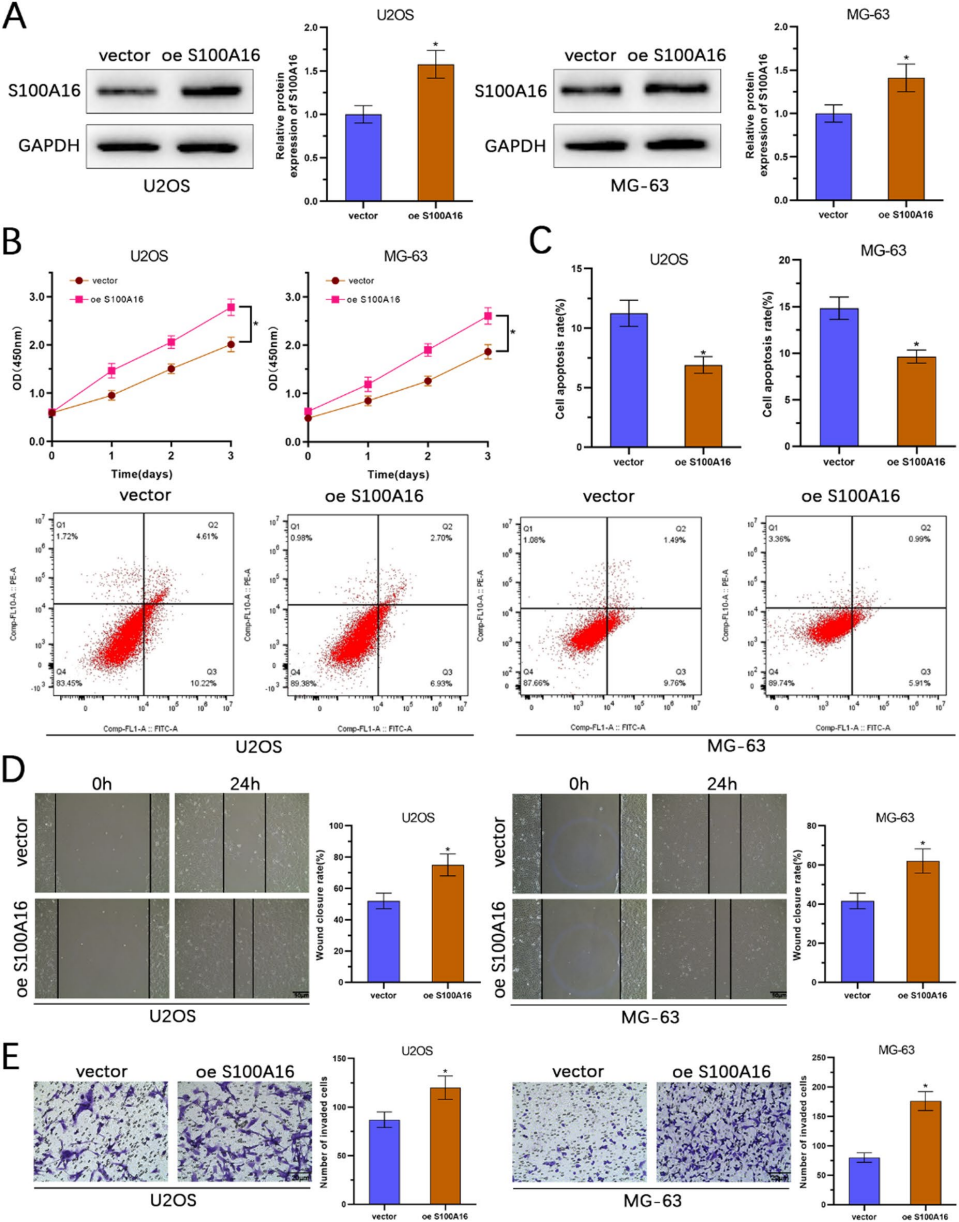
S100 A16 promotes proliferation, migration, and invasion of osteosarcoma cells while inhibiting apoptosis. (**A**) Overexpression efficiency of S100 A16 in U2OS and MG-63 cells was evaluated using Western blotting analysis. (**B**) Cell viability of U2OS and MG-63 cells following S100 A16 overexpression was examined by CCK-8 assay. (**C**) The rate of apoptosis was assessed via flow cytometry after S100 A16 overexpression. (**D**) Wound healing assays were performed to evaluate the migratory capability of cells after S100 A16 overexpression. (**E**) Transwell assays were used to assess the invasive potential of cells following S100 A16 overexpression (*n* = 3). *p < 0.05.

To investigate the functional roles of S100 A16 in osteosarcoma, we conducted experiments in U2OS and MG-63 cell lines. WB analysis confirmed successful overexpression of S100 A16 in both cell lines (Fig. 2A). CCK-8 assays demonstrated that overexpression of S100 A16 significantly enhanced cell viability (Fig. 2B). Flow cytometry analysis revealed a reduction in apoptosis rates in cells overexpressing S100 A16 (Fig. 2C). Wound healing assays showed that S100 A16 overexpression increased the migration capacity of the cells (Fig. 2D). Furthermore, transwell invasion assays indicated that overexpression of S100 A16 significantly enhanced the invasive ability of the cells (Fig. 2E). These results collectively suggest that upregulation of S100 A16 promotes osteosarcoma cell proliferation, migration, and invasion.

## Downregulation of S100 A16 inhibits osteosarcoma cell proliferation, migration, and invasion

**Fig. 3:**
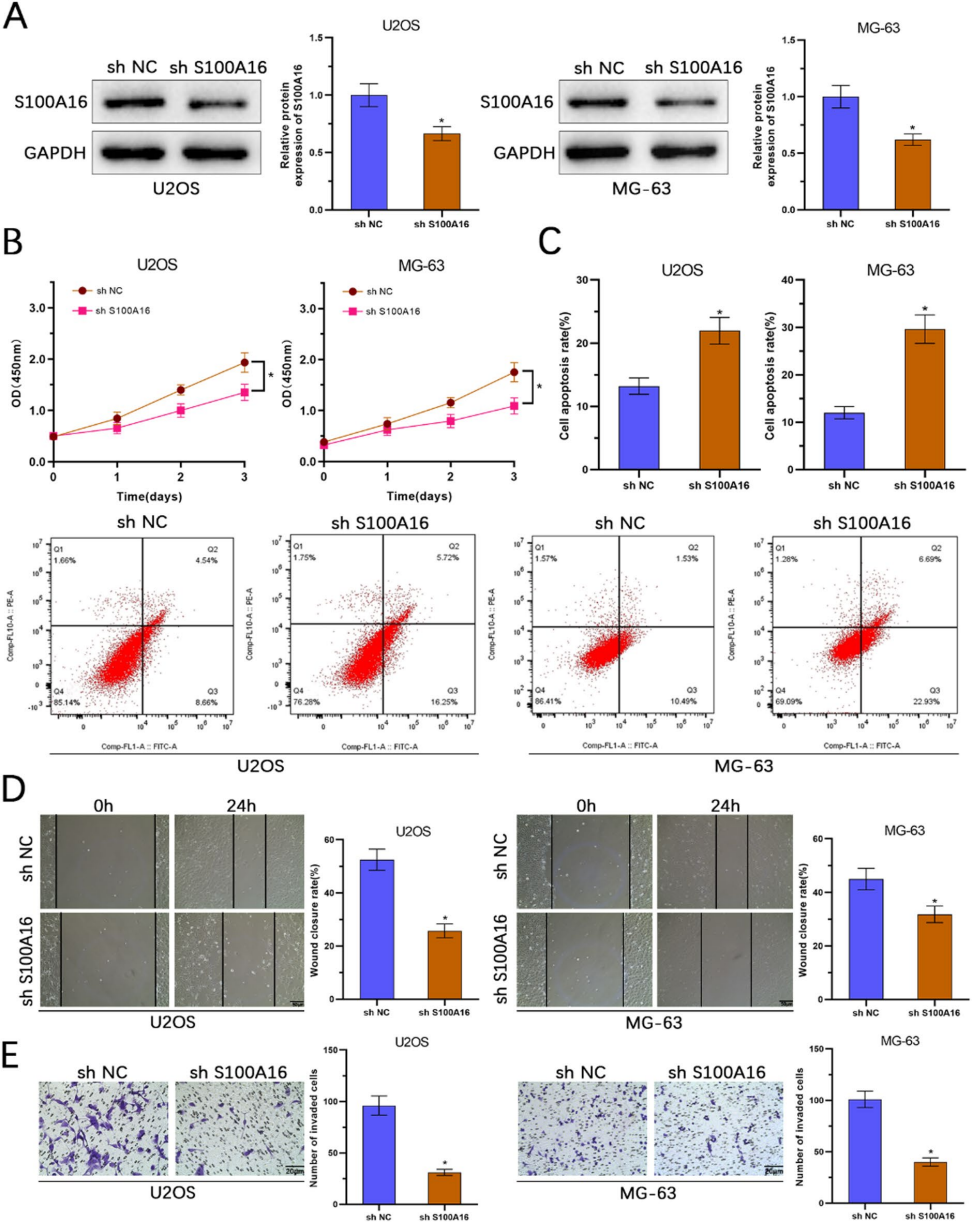
Suppression of S100 A16 reduces proliferation, migration, and invasion of osteosarcoma cells while promoting apoptosis. (**A**) The efficiency of S100 A16 knockdown in U2OS and MG-63 cells was evaluated using Western blotting analysis. (**B**) Cell viability of U2OS and MG-63 cells following S100 A16 knockdown was assessed by CCK-8 assay. (**C**) The rate of apoptosis was determined via flow cytometry after S100 A16 knockdown. (**D**) Wound healing assays were conducted to examine the migratory capability of cells post-S100 A16 knockdown. (**E**) Transwell invasion assays were performed to evaluate the invasive potential of cells following S100 A16 knockdown (*n* = 3). *p < 0.05.

To further elucidate the role of S100 A16 in osteosarcoma, we investigated the effects of S100 A16 knockdown in U2OS and MG-63 cell lines. WB analysis confirmed efficient knockdown of S100 A16 expression (Fig. 3A). CCK-8 assays showed that downregulation of S100 A16 significantly reduced cell viability (Fig. 3B). Flow cytometry analysis revealed an increase in apoptosis rates in cells with S100 A16 knockdown (Fig. 3C). Wound healing assays demonstrated that S100 A16 knockdown decreased the migration capacity of the cells (Fig. 3D). Additionally, transwell invasion assays indicated that downregulation of S100 A16 significantly reduced the invasive ability of the cells (Fig. 3E). These results collectively suggest that downregulation of S100 A16 inhibits osteosarcoma cell proliferation, migration, and invasion.

## TCGA data set and functional enrichment analysis to identify S100 A16-Related genes

**Fig. 4:**
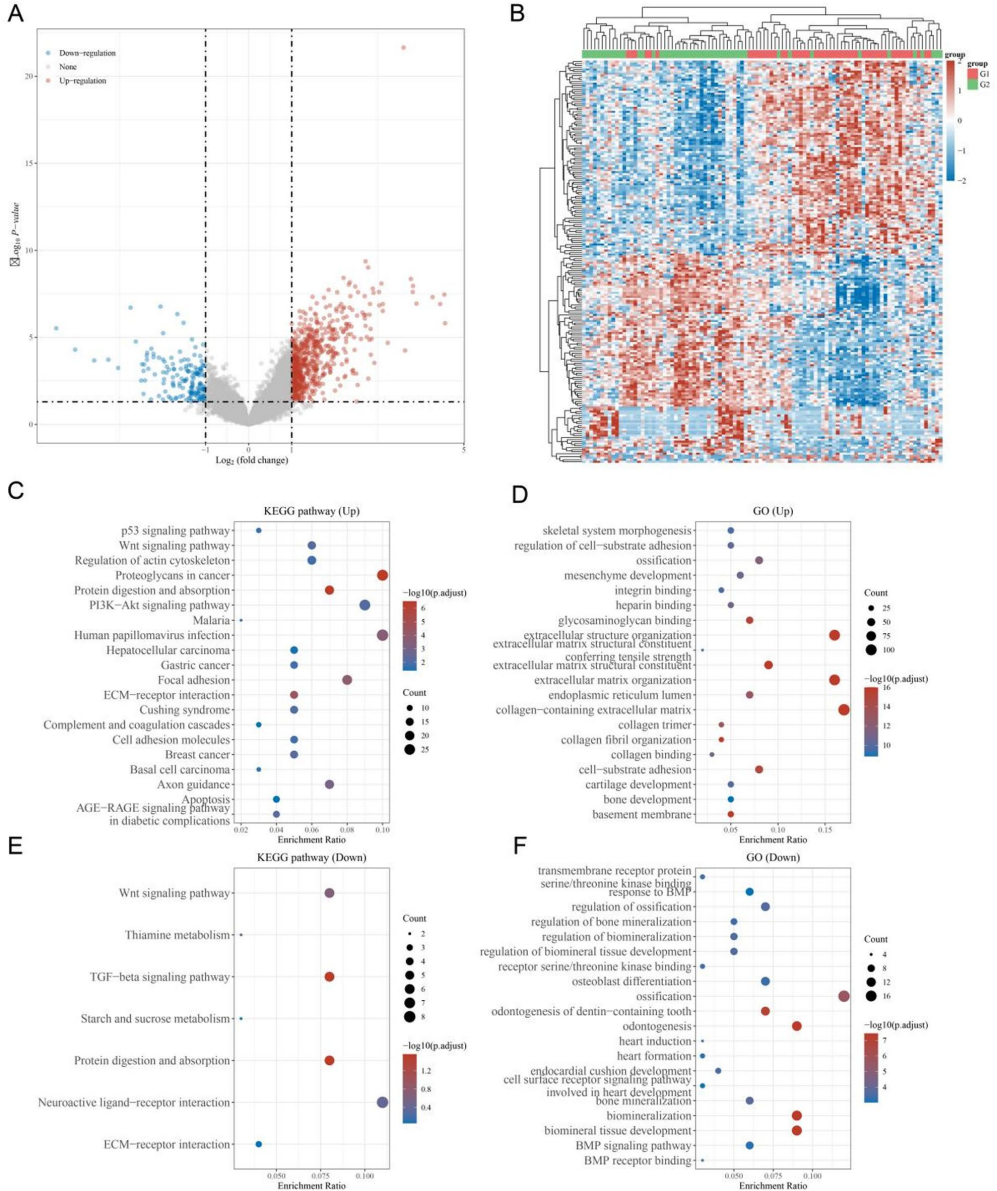
Identification of S100 A16-associated genes using the TCGA-OS dataset and functional enrichment analysis. (**A**, **B**) The TCGA-OS dataset was utilized to identify genes that exhibit a strong correlation with S100 A16 in osteosarcoma, with particular emphasis on those showing |log_2_FC| > 2 for significant upregulation or downregulation. The top 50 genes were visualized using heatmaps. (**C**, **D**) Comprehensive KEGG and GO analyses were performed for genes positively correlated with S100 A16 in osteosarcoma. (**E**, **F**) Comprehensive KEGG and GO analyses were also conducted for genes negatively correlated with S100 A16 in osteosarcoma.

To identify genes strongly correlated with S100 A16 in osteosarcoma, we utilized the TCGA-OS data set. Genes with a significant fold change (|log_2_FC| > 2) were identified as being either upregulated or downregulated in association with S100 A16 (Fig. 4A). The top 50 differentially expressed genes were visualized using a heatmap (Fig. 4B). Functional enrichment analysis was performed on these differentially expressed genes. KEGG (Kyoto Encyclopedia of Genes and Genomes)^20^ pathway analysis revealed that upregulated genes were primarily enriched in the PI3 K-AKT signaling pathway (Fig. 4C), while GO (Gene Ontology) analysis indicated that these genes were mainly involved in biological processes related to extracellular matrix containing collagen (Fig. 4D). Conversely, KEGG analysis of downregulated genes showed enrichment in the neuroactive ligand-receptor interaction pathway (Fig. 4E), and GO analysis indicated that these genes were primarily associated with ossification (Fig. 4F). These findings provide insights into the potential mechanisms through which S100 A16 influences osteosarcoma development and progression.

## S100 A16 regulates the PI3 K/AKT pathway in osteosarcoma

**Fig. 5:**
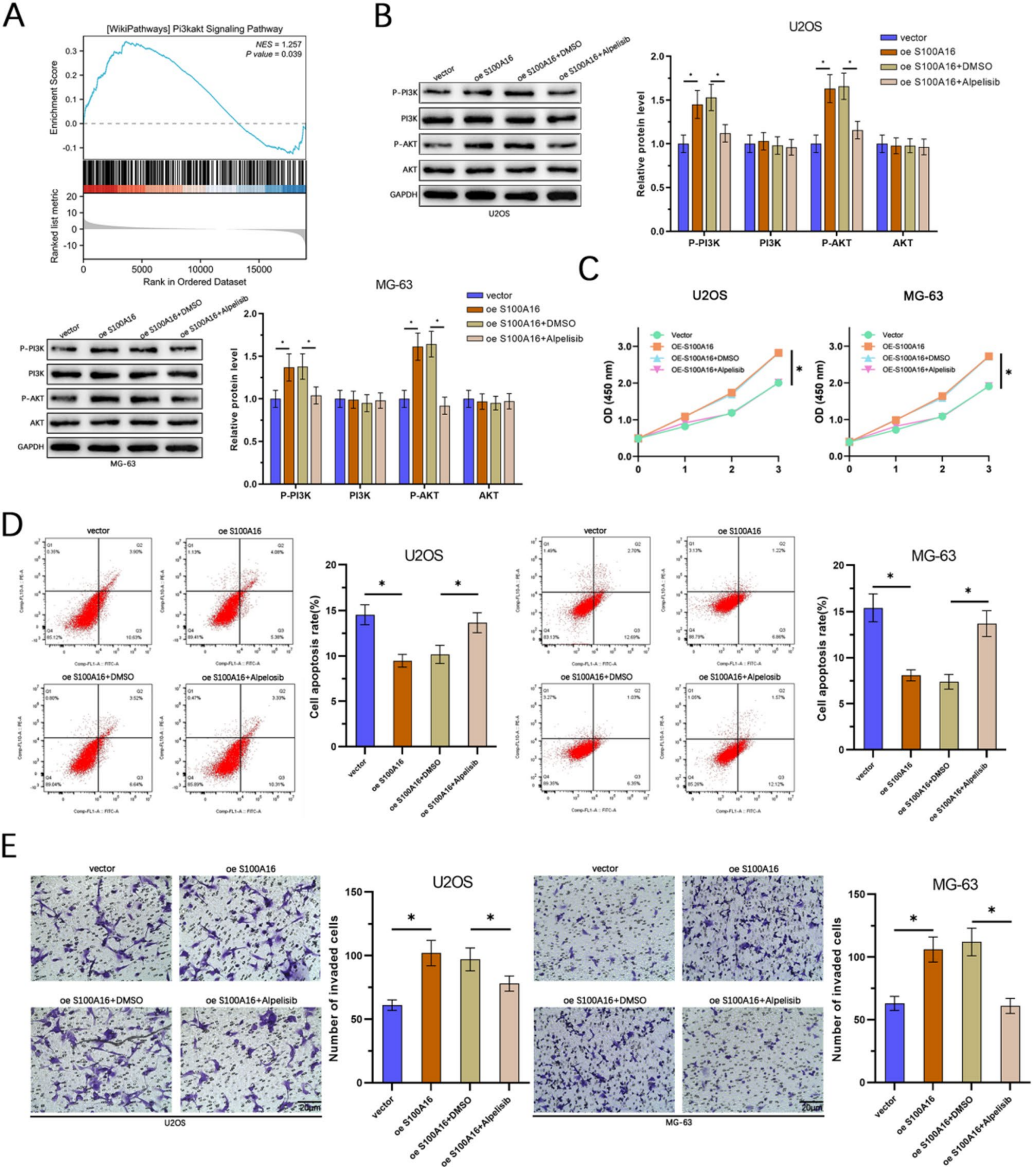
S100 A16 modulates the PI3 K/AKT pathway in osteosarcoma. (**A**) GSEA revealed that elevated expression of S100 A16 was associated with enrichment in the PI3 K/AKT signaling pathway. (**B**) Western blot analysis was conducted to examine the expression levels of four key proteins related to the PI3 K/AKT pathway—p-PI3 K, PI3 K, p-AKT, and AKT—in U2OS and MG-63 cell lines across four groups. (**C**) Cell viability changes among the four groups were assessed using the CCK-8 assay. (**D**) The apoptosis capability alterations in the four groups were determined through flow cytometry analysis. (**E**) Changes in cell invasion ability were evaluated using transwell invasion assays (*n* = 3). *p < 0.05.

GSEA (Gene Set Enrichment Analysis) revealed that elevated expression of S100 A16 was significantly enriched in the PI3 K/AKT signaling pathway (Fig. 5A). Compared to other pathways, the PI3 K/AKT signaling pathway demonstrated higher enrichment ratio and greater number of enriched genes. Consequently, we prioritized the PI3 K/AKT pathway for further investigation. To further investigate this finding, we conducted experiments using the U2OS and MG-63 cell lines, dividing the cells into four groups: control (Vector), overexpression of S100 A16 (OE S100 A16), OE S100 A16 + DMSO, and OE S100 A16 + Alpelisib (a PI3 K pathway inhibitor). WB analysis demonstrated that the levels of p-PI3 K and p-AKT were significantly increased in the OE S100 A16 and OE S100 A16 + DMSO groups compared to the control group. However, the addition of Alpelisib led to a marked reduction in p-PI3 K levels, while the total levels of PI3 K and AKT remained unchanged across all groups (Fig. 5B). Cell viability assays using CCK-8 showed that the cell proliferation activity was significantly enhanced in the OE S100 A16 and OE S100 A16 + DMSO groups, and this effect was reversed upon the addition of Alpelisib (Fig. 5C). Flow cytometry analysis revealed a decrease in the apoptosis rate in the OE S100 A16 and OE S100 A16 + DMSO groups, which was restored to higher levels when Alpelisib was introduced (Fig. 5D). Transwell invasion assays indicated that the invasive capability of the cells was significantly increased in the OE S100 A16 and OE S100 A16 + DMSO groups, and this increase was attenuated by the addition of Alpelisib (Fig. 5E). These results collectively suggest that S100 A16 promotes osteosarcoma cell proliferation, survival, and invasion by activating the PI3 K/AKT signaling pathway.

## S100 A16 regulates ANXA2 expression in osteosarcoma

**Fig. 6:**
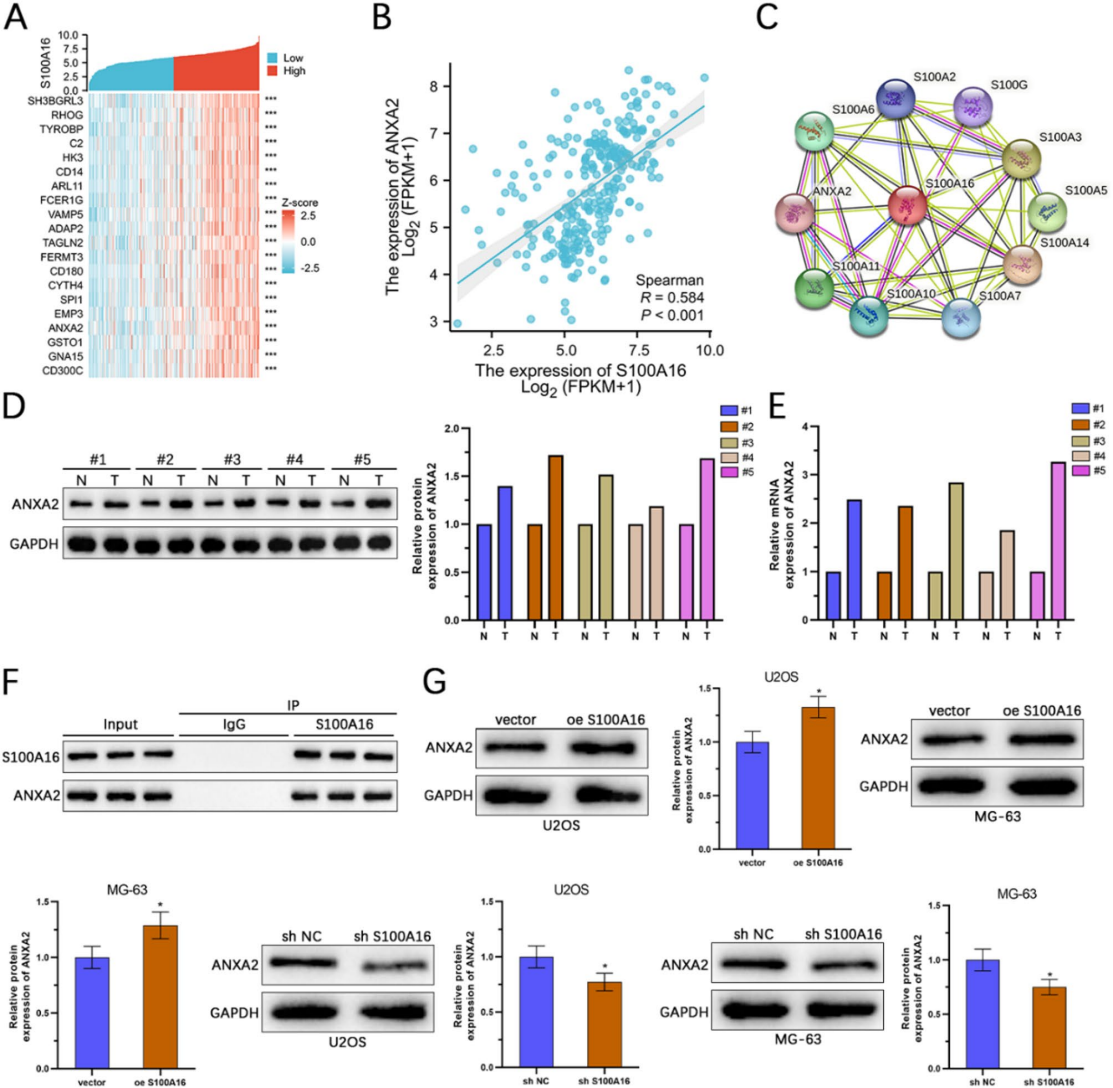
S100 A16 regulates the expression of ANXA2 in osteosarcoma. (**A**) A heatmap displays the top 20 genes that exhibit a strong positive correlation with S100 A16 in osteosarcoma. (**B**) The expression of ANXA2 shows a robust positive correlation with S100 A16 (Spearman correlation coefficient = 0.584). (**C**) The interaction between S100 A16 and ANXA2 was identified through PPI analysis using the STRING database. (**D**, **E**) Western blotting and qRT-PCR analyses were performed to evaluate the expression levels of ANXA2 in osteosarcoma tissue samples as well as in adjacent normal tissues (*n* = 5). (**F**) The interaction between S100 A16 and ANXA2 was validated using Co-IP as-says. (**G**) Western blot analysis was used to assess the expression of ANXA2 in U2OS and MG-63 cells following overexpression or knockdown of S100 A16 (*n* = 3). *p < 0.05.

To gain a deeper understanding of the potential biological functions of S100 A16 in osteosarcoma, we performed co-expression analysis using data from the TCGA-OS dataset. A heatmap was generated to visually represent the top 20 genes significantly positively correlated with S100 A16 in osteosarcoma (Fig. 6A). We examined the relationship between S100 A16 and ANXA2 expression levels in patient data and found a strong positive correlation (Spearman correlation coefficient = 0.584) (Fig. 6B). To further explore the biological processes associated with S100 A16, we used the STRING database to generate a protein-protein interaction (PPI) network, focusing on S100 A16 in osteosarcoma. Interestingly, our analysis revealed potential interactions between S100 A16 and other proteins, such as ANXA2 and S100 A2 (Fig. 6C). WB and qRT-PCR analyses confirmed that ANXA2 expression was significantly upregulated in osteosarcoma tissues compared to normal tissues (Figs. 6D, E). Co-IP experiments verified the physical interaction between S100 A16 and ANXA2 (Figs. 6F). Additionally, WB analysis showed that overexpression of S100 A16 led to an increase in ANXA2 expression, while knockdown of S100 A16 resulted in a decrease in ANXA2 expression (Fig. 6G). These results collectively indicate that S100 A16 regulates ANXA2 expression in osteosarcoma, suggesting a potential mechanism through which S100 A16 contributes to the progression of the disease.

### S100 A16 activates the PI3 K/AKT pathway via ANXA2 to promote osteosarcoma cell proliferation and invasion

**Fig. 7:**
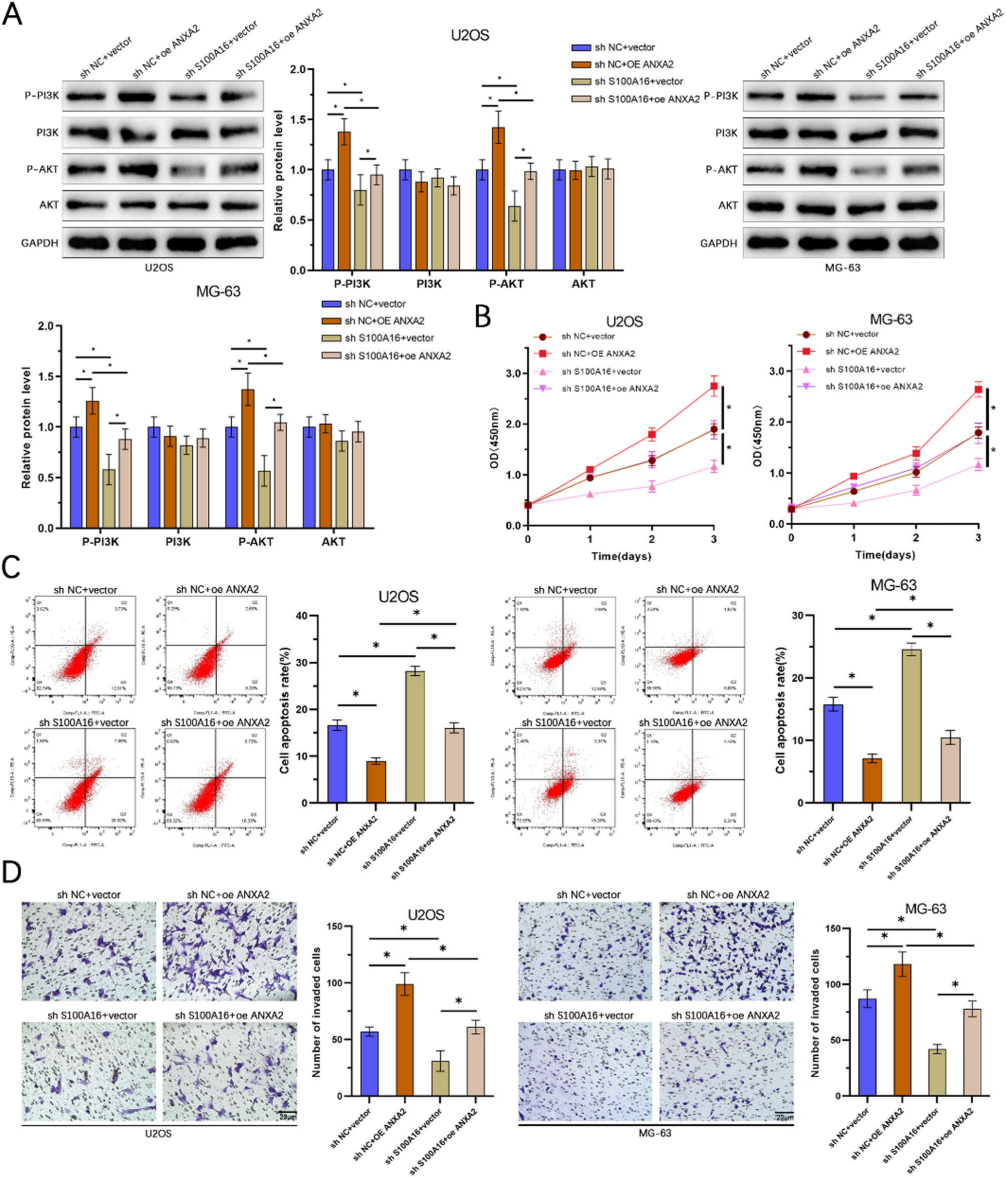
S100 A16 promotes osteosarcoma cell proliferation and invasion by activating the PI3 K/AKT pathway through interaction with ANXA2. (**A**) Western blot analysis was performed to evaluate the expression levels of p-PI3 K, PI3 K, p-AKT, and AKT proteins in U2OS and MG-63 cells following S100 A16 overexpression and/or ANXA2 knockdown. (**B**) Cell growth was assessed using the CCK-8 assay for all groups. (**C**) The level of apoptosis was evaluated via flow cytometry for each group. (**D**) Transwell assays were conducted to assess the invasive capabilities of cells across all groups (*n* = 3). *p < 0.05.

To investigate the mechanism by which S100 A16 activates the PI3 K/AKT pathway through ANXA2, we conducted experiments in U2OS and MG-63 cell lines, dividing the cells into four groups: Sh NC + Vector, Sh S100 A16 + Vector, Sh NC + OE ANXA2, and Sh S100 A16 + OE ANXA2. WB analysis revealed that the levels of p-PI3 K and p-AKT were significantly reduced in the Sh S100 A16 + Vector group compared to the Sh NC + Vector group. However, overexpression of ANXA2 in the Sh S100 A16 + OE ANXA2 group restored the expression levels of p-PI3 K and p-AKT, indicating that S100 A16 activates the PI3 K/AKT pathway through ANXA2 (Fig. 7A). Cell viability assays using CCK-8 showed that the cell proliferation activity was significantly decreased in the Sh S100 A16 + Vector group compared to the Sh NC + Vector group. This reduction in cell viability was reversed by overexpression of ANXA2 in the Sh S100 A16 + OE ANXA2 group (Fig. 7B). Flow cytometry analysis revealed that the apoptosis rate was significantly increased in the Sh S100 A16 + Vector group, and this increase was reversed by overexpression of ANXA2 in the Sh S100 A16 + OE ANXA2 group (Fig. 7C). Transwell invasion assays indicated that the invasive capability of the cells was significantly reduced in the Sh S100 A16 + Vector group, and this reduction was reversed by overexpression of ANXA2 in the Sh S100 A16 + OE ANXA2 group (Fig. 7D). These results collectively demonstrate that S100 A16 activates the PI3 K/AKT pathway via ANXA2, thereby promoting osteosarcoma cell proliferation and invasion.

## Discussion

Osteosarcoma is a primary malignant bone tumor that primarily affects children and adolescents. Its incidence is relatively low, with approximately 3–4 cases per million people per year^21^. Typical symptoms include local pain, swelling, and the formation of a mass. Diagnosis relies on a combination of clinical presentation, imaging, and pathology^22^. Treatment typically involves a standard regimen of chemotherapy combined with surgery, which has improved outcomes, leading to a 5-year survival rate of 75–80% since the introduction of chemotherapy^23^. Despite advances in treatment, the molecular mechanisms of osteosarcoma are not yet fully understood, making this a focus of ongoing research. The aim is to gain a deeper understanding of the causes and progression of the disease and to explore more effective treatment strategies.

S100 proteins generally consist of two homodimers or heterodimers and are considered multifunctional signaling factors involved in regulating various intracellular or extracellular processes, including cell proliferation, differentiation, migration, apoptosis, and autoimmunity^24^. S100 A16 is a member of the S100 family and is widely present in various types of cells, participating in numerous physiological and pathological processes^25^. Similar to the structure of other S100 proteins, S100 A16 contains two EF-hand motifs, forming a helix-loop-helix domain, where the N-terminal domain is interconnected with the C-terminal domain via a “hinge” linker. Both EF-hand motifs serve as binding sites for Ca^2+^ and Zn^2+^, though specifically noted for S100 A6^26^. It has been reported that the mRNA and protein levels of S100 A16 are differentially expressed in most human cancers. Studies have shown that the expression level of S100 A16 is increased in Wilms’ tumors, and the deficiency of S100 A16 can inhibit the proliferation, invasion, migration, and angiogenic capabilities of Wilms’ tumor cells^27^. Additionally, it has been reported that S100 A16 is overexpressed in breast cancer, and S100 A16 can promote the invasive activity of breast cancer cells through cytoskeleton dynamics^28^. Xu et al. also discovered that S100 A16 facilitates brain metastasis in small cell lung cancer by modulating mitochondrial function^29^. In our study, we detected elevated expression levels of S100 A16 in osteosarcoma and demonstrated that high expression of this gene correlates positively with poor OS, DSS, and PFI in osteosarcoma. Furthermore, we investigated the biological functions of S100 A16 and elucidated its role in promoting proliferation, metastasis, invasion, and anti-apoptotic characteristics in osteosarcoma cells. Our data collectively showed that S100 A16 may serve as a potential molecular target for osteosarcoma therapy. Recently, it has been identified that S100 A16 serves as a novel and potential therapeutic target in pancreatic ductal adenocarcinoma (PDAC), and its downregulation synergistically suppresses PDAC progression in combination with gemcitabine^30^. Gemcitabine is one of the commonly chemotherapeutic agents for treatment of relapsed and refractory osteosarcoma^31^. The study by Li et al. has provided novel therapeutic strategies for the future treatment of osteosarcoma^30^. S100 A16-targeted inhibition combine with chemotherapy may emerge as a novel clinical therapeutic regimen for osteosarcoma.

The ANXA family is a member of the annexin superfamily of calcium ion-dependent phospholipid-binding proteins, which are involved in various cellular functions including membrane transport^32^. ANXA2 is one of the members of this family, and the ANXA2 protein is a roughly 36 kDa protein composed of 339 amino acid residues^33^. As a multifunctional protein, ANXA2 can interact with various ligands and influence different cellular processes such as membrane trafficking, endocytosis, exocytosis, tissue remodeling, angiogenesis, and immune modulation^34^. Studies have found that high expression of ANXA2 is associated with the invasive behavior of breast cancer and serves as an important indicator of poor prognosis in triple-negative breast cancer^35^knockdown of ANXA2 inhibited cell migration and invasion ability^36^. It has also been reported that ANXA2 is highly expressed in bladder cancer tissues, and inhibiting ANXA2 can enhance the sensitivity of bladder cancer to doxorubicin, thereby improving the efficacy of chemotherapy^37^. Additionally, researchers have found that TIM-4 interacts with ANXA2 to activate the PI3 K/AKT signaling pathway, promoting oxidative phosphorylation in lung cancer cells to accelerate tumor progression^38^. In our study, we identified that S100 A16 promotes the progression of osteosarcoma through its interaction with ANXA2. Overexpression of S100 A16 increases the levels of ANXA2, thereby enhancing the proliferation, migration, invasion, and anti-apoptotic capabilities of osteosarcoma cells. Protein-protein interactions serve as a critical biological mechanism underlying the regulation of protein homeostasis and functional activities in living organisms^39^. Such interactions may modulate protein degradation, subcellular localization, and functional activities through mechanisms encompassing post-translational modifications, chaperone-mediated conformational regulation, and transcriptional co-regulatory complexes^40^. Studies have demonstrated that S100 A4, a member of the S100 calcium-binding protein family, suppresses p53 activity by modulating its post-translational modifications (e.g., phosphorylation and acetylation), thereby promoting pancreatic cancer cell proliferation, migration, and disease progression^41^. Moreover, the interaction of ANXA2 with other proteins, such as NUSAP1^42^ and RECQL4^43^, protecting it against protein degradation via impeding its ubiquitination process. Collectively, these findings suggest that the S100 A16-ANXA2 interaction may enhance ANXA2 expression by modulating its post-translational modifications, particularly ubiquitination. In future, studies elucidating the molecular mechanisms underlying S100 A16-mediated regulation of ANXA2 expression could provide critical insights into the pathogenesis of osteosarcoma progression.

The PI3 K/AKT signaling pathway is a crucial intracellular signaling route that plays a pivotal role in multiple biological processes, including cell growth, proliferation, differentiation, survival, and metabolism^44^. This pathway consists of several molecules, with the most central being phosphatidylinositol 3-kinase (PI3 K) and protein kinase B (PKB or AKT)^45^. When cell surface receptors receive signals from growth factors or other stimuli, PI3 K becomes activated, catalyzing the conversion of substrate phosphatidylinositol (PIP2) into phosphatidylinositol triphosphate (PIP3). PIP3 recruits and activates AKT, which becomes fully active after phosphorylation under specific conditions. Activated AKT can further phosphorylate a series of downstream target proteins, affecting their functional states and thus regulating the cell cycle, inhibiting apoptosis, and promoting glycogen synthesis, among other cellular activities^46^. The PI3 K/AKT pathway is also critically important in cancer progression. Research has shown that the PI3 K/AKT signaling pathway is involved in various cancers and malignant tumor, such as prostate cancer^47^renal cancer^48^breast cancer^49^bladder cancer and osteosarcoma^50^. Li et al. reported that USP3 promotes the progression of osteosarcoma by deubiquitinating EPHA2 and activating the PI3 K/AKT signaling pathway^51^. Enhanced phosphorylation of PI3 K/AKT pathway components promotes osteosarcoma cell proliferation, migration, and invasion^52^. In this investigation, we conducted KEGG, GO, and GSEA analyses and identified a significant correlation between increased S100 A16 expression and the PI3 K/AKT pathway. Consequently, we conducted further studies and observed activation of the PI3 K/AKT pathway when S100 A16 was overexpressed, while knockdown of S100 A16 led to inhibition of the PI3 K/AKT pathway in osteosarcoma cells. Therefore, our results suggest that S100 A16 promotes the activation of the PI3 K/AKT pathway, contributing to the progression of osteosarcoma. The PI3 K/AKT is the downstream signaling pathways of ANXA2. According to reports in the literature, ANXA2 regulates chondrocyte differentiation via PI3 K/AKT pathway^53^. By activating the PI3 K/AKT pathway, ANXA2 enhanced the migration and invasion capability of breast cancer cells^54^. In this study, we found that overexpression of ANXA2 reversed the inhibitory effect of S100 A16 knockdown on PI3 K/AKT pathway. Functional cellular assays demonstrated that S100 A16 promotes osteosarcoma cell proliferation and invasion by activating the PI3 K/AKT pathway via ANXA2.

## Conclusion

This study shows that S100 A16 is significantly upregulated in osteosarcoma and promotes cell proliferation, migration, and invasion by regulating ANXA2 and activating the PI3 K/AKT signaling pathway. Bioinformatics analysis confirmed elevated S100 A16 levels in osteosarcoma datasets, validated in human samples. Upregulation of S100 A16 enhanced cell viability and reduced apoptosis, while its downregulation had opposite effects. Functional enrichment analysis indicated high enrichment of osteosarcoma-related genes in the PI3 K-AKT pathway. Treatment with the PI3 K inhibitor Alpelisib reduced proliferation and invasion in S100 A16-overexpressing cells, suggesting S100 A16’s reliance on PI3 K/AKT for promoting osteosarcoma. Strong positive correlations between S100 A16 and ANXA2 were found, with co-IP confirming direct interactions. Knockdown of S100 A16 followed by ANXA2 overexpression rescued cell viability, apoptosis, and invasion, indicating S100 A16 activates PI3 K/AKT through ANXA2. In conclusion, our study reveals the mechanism of S100 A16 in osteosarcoma and suggests that targeting the S100 A16/ANXA2/PI3 K/AKT axis could be a feasible strategy for osteosarcoma therapy.

## Materials and methods

### Bioinformatics

To investigate the expression patterns and potential functions of S100 A16 in osteosarcoma, we utilized The Cancer Genome Atlas (TCGA) database (https://portal.gdc.cancer.gov) and the Gene Expression Omnibus data base (GEO) database (https://www.ncbi.nlm.nih.gov/geo/). TCGA-OS along with datasets GSE56001 and GSE19276 provided extensive gene expression data and other clinical information. From these sources, we obtained RNA sequencing data and corresponding clinical information for osteosarcoma patients. The RNA sequencing data were provided in the form of fragments per kilobase of transcript per million mapped reads (FPKM), allowing us to accurately quantify the expression levels of the S100 A16 gene across different samples. By comparing the data from tumor samples with those from normal tissue samples, we analyzed the differential expression of S100 A16 and visually presented these differences using volcano plots. Additionally, we used the “Spearman rank correlation coefficient” (implemented via the “cor.test” package in R) to evaluate the correlation between S100 A16 and other potentially associated genes, revealing their synergistic roles in the development of osteosarcoma. To further explore the functional aspects of S100 A16 and its interactions with other genes, we conducted Gene Ontology (GO), Kyoto Encyclopedia of Genes and Genomes (KEGG), and Gene Set Enrichment Analysis (GSEA) using the “clusterProfiler” package. These analyses helped us understand the role of S100 A16 in the PI3 K/AKT signaling pathway, particularly how it influences cell proliferation, migration, and invasion to promote the progression of osteosarcoma. To investigate potential protein-protein interactions, we utilized the STRING database (https://string-db.org/) to examine the interaction networks between S100 A16 and proteins such as ANXA2.

### Cell culture and transfection

We utilized the normal osteoblastic cell line hfOB1.19 and three osteosarcoma cell lines: 143B, U2OS, and MG-63, all acquired from the American Type Culture Collection (ATCC). The hfOB1.19, 143B, and MG-63 cell lines were cultured in DMEM supplemented with 10% fetal bovine serum (FBS) and 1% penicillin/streptomycin (both supplied by Beyotime Biotechnology). The U2OS cell line was cultured in McCoy’s 5a medium with the same FBS and antibiotic mixture. All cell lines were incubated at 37 °C in a humidified atmosphere containing 5% CO₂. The cell culture media used in this study were supplied by Beyotime Biotechnology.

For the overexpression and knockdown experiments of the S100 A16 and ANXA2 genes, we used lentiviral vectors provided by GeneChem (Shanghai, China). Lentiviral transfections for the U2OS and MG-63 cell lines were performed using HiTransG P infection enhancer solution, and the transfection conditions were optimized with a multiplicity of infection (MOI) of 20. To verify the transfection efficiency, we evaluated the mRNA levels of the target genes using quantitative real-time polymerase chain reaction (qRT-PCR) and assessed the protein expression levels via Western blot (WB) analysis to determine if the puromycin selection (at a concentration of 2 µg/ml) successfully achieved the intended gene expression regulation.

### Western blot assay

To validate the expression levels and regulatory effects of S100 A16 and ANXA2 in osteosarcoma cells, we used WB technology. U2OS and MG-63 cells were collected and lysed with RIPA buffer. Protein concentrations were determined using the BCA assay to ensure consistency across samples. Protein samples were mixed with 5X SDS-PAGE loading buffer, denatured by boiling for 5 min, and separated by SDS-PAGE. Pro-teins were then transferred onto PVDF membranes. The membranes were blocked for 1 h in TBST containing 5% non-fat milk to reduce non-specific binding. The membranes were incubated overnight at 4 °C with primary antibodies: S100 A16 (1:1000; 11456-1-AP; PROTEINTECH GROUP, USA), ANXA2 (1:5000; 11256-1-AP; PRO-TEINTECH GROUP, USA), p-PI3 K (1:2000; ab182651; Abcam), PI3 K (1:2000; 20584-1-AP; PROTEINTECH GROUP, USA), p-AKT (1:2000; 66444-1-Ig; PROTEINTECH GROUP, USA), and AKT (1:2000; 10176-2-AP; PROTEINTECH GROUP, USA). After washing with TBST, the membranes were incubated with HRP-conjugated secondary antibodies at room temperature for 1 h. Following additional washes, the membranes were developed using ECL reagents and imaged using a chemiluminescence imaging system. GAPDH served as a loading control. Band intensities were quantified using densitometry analysis software to assess the relative expression levels of the target proteins.

### qRT-PCR experiments

To quantitatively detect the mRNA expression levels of S100 A16 and ANXA2 in osteosarcoma cells, we extracted total RNA from U2OS and MG-63 cells using Trizol reagent. The quality and concentration of the RNA were assessed using 1% agarose gel electrophoresis and a NanoDrop spectrophotometer. Total RNA (1 µg) was reverse-transcribed into cDNA using the GoScript Reverse Transcription System from Promega. qRT-PCR reactions were performed using Bio-Rad iQ SYBR Green Supermix. Each reaction mixture contained 1 µL of cDNA template, 0.5 µL of forward primer (10 µM), 0.5 µL of reverse primer (10 µM), 10 µL of 2× SYBR Green Master Mix, and ddH2O to a final volume of 20 µL. The thermal cycling conditions were as follows: initial denaturation at 95 °C for 3 minutes, followed by 40 cycles of 95 °C for 15 seconds, 60 °C for 30 seconds, and 72 °C for 30 seconds. A melting curve analysis was performed at the end to confirm the specificity of the amplified products. The relative expression levels of the target genes were calculated using the 2^(-ΔΔCt) method, normalized to the reference gene GAPDH. The sequences of the primers used were as follows: S100 A16: Forward: 5’-TGGTCAAGAACAAGATCAGCAAGAG-3’ Reverse: 5’-GATGAGCTTATCCGCAGCCTTC-3’, ANXA2: Forward: 5’-ATGGTCTCCCGCAGTGAAGTG-3’ Reverse: 5’-TGGTAGTCGCCCTTAGTGTCTTG-3’, GAPDH: Forward: 5’-GTGGACCTGACCTGCGTCT-3’, Reverse: 5’-GTGTCGCTGTTGAAGTCAGAGGAG-3’.

### Cell viability assay

To assess cell viability and proliferation, U2OS and MG-63 cells were seeded in 96-well plates at 5,000 cells per well and incubated at 37 °C with 5% CO₂ for 24 h. After 24 h, the medium was replaced with fresh medium containing the desired treatments. At specified time points (24, 48, and 72 h), 10 µL of CCK-8 solution was added to each well, and plates were incubated for 1–4 h at 37 °C. Absorbance was measured at 450 nm using a microplate reader. Cell viability was calculated as the percentage of absorbance relative to the control.

### Scratch assay

To evaluate the effect of S100 A16 on the migratory ability of osteosarcoma cells, we performed a scratch assay. U2OS and MG-63 cells were seeded in 6-well plates at the same density and allowed to grow until they reached 90% confluence. A sterile 200 µL pipette tip was used to create a straight scratch in the center of the cell monolayer. Detached cells were gently removed by washing with PBS, and the wells were then replenished with DMEM containing 2% FBS to minimize cell proliferation. Images of the scratches were captured at 0 and 24 h using an inverted microscope. The changes in scratch width were measured using ImageJ software. Cell migration was assessed by calculating the scratch closure rate.

### Invasion assay

To evaluate the effect of S100 A16 on the invasive capacity of osteosarcoma cells, we performed an invasion assay using transwell chambers (Beyotime Biotechnology, Shanghai, China). First, Matrigel basement membrane matrix (Beyotime Biotechnology, Shanghai, China) was diluted 1:8 in serum-free DMEM. Fifty microliters of the diluted Matrigel were evenly applied to the bottom of the upper chamber of the transwell insert and incubated at 37 °C with 5% CO₂ for 1 h to allow it to solidify. Next, U2OS and MG-63 cells were resuspended in DMEM containing 1% FBS at a density of 2 × 104 cells/100 µL and added to the upper chamber. The lower chamber was filled with 600 µL of DMEM containing 10% FBS to serve as a chemoattractant. The transwell chambers were then incubated at 37 °C with 5% CO₂ for 24 h. After incubation, non-invading cells on the upper surface of the membrane were gently removed with a cotton swab. The invaded cells on the lower surface were fixed with 4% paraformaldehyde for 15 min and stained with 0.1% crystal violet for 15 min. Finally, the cells were observed and photographed under an optical microscope, and the number of cells that had invaded through the Matrigel was counted using ImageJ software.

### Apoptosis assay

To evaluate the effect of S100 A16 on apoptosis in osteosarcoma cells, we performed flow cytometric analysis using the Annexin V/PI double staining method. U2OS and MG-63 cells were seeded in 6-well plates at the same density and allowed to grow until they reached 70–80% confluence. The cells were then treated for 24 h. After treatment, the cells were collected, washed twice with PBS, and resuspended in 1× binding buffer at a concentration of 1 × 10^6^ cells/mL. One hundred microliters of the cell suspension were aliquoted into tubes, and 5 µL of Annexin V-FITC and 5 µL of PI were added to each tube. The samples were incubated in the dark for 15 min. After incubation, 400 µL of 1× binding buffer was added to each tube, and the mixtures were gently vortexed. The samples were immediately analyzed using a BD FACSCalibur flow cytometer. Apoptotic cells were distinguished from live cells, early apoptotic cells, late apoptotic cells, and necrotic cells based on the fluorescence signals of Annexin V-FITC and PI. The apoptosis rates of the different groups were calculated using FlowJo software.

### Endogenous coimmunoprecipitation (Co-IP) experiments

To verify the interaction between S100 A16 and ANXA2, we performed endogenous Co-IP experiments. U2OS and MG-63 cells were seeded in 10 cm dishes at the same density and allowed to grow until they reached 80% confluence. Cells were directly collected and lysed using RIPA buffer. The protein concentration of the lysates was determined using the BCA assay. One milligram of protein lysate was incubated with S100 A16 antibody or ANXA2 antibody at 4 °C with rotation overnight. Protein A/G magnetic beads were then added, and the mixture was incubated for an additional 2 h. The beads were washed, and 2× SDS loading buffer was added. The samples were boiled for 5 min and subjected to SDS-PAGE. Proteins were transferred to PVDF membranes and detected by Western blot using antibodies specific to S100 A16 and ANXA2.

### Statistical analysis

All experiments were independently repeated three times, and data are presented as mean ± standard deviation (mean ± SD). Normality of the data was assessed using the Shapiro-Wilk test. Comparisons between two groups were performed using independent samples t-tests (for normally distributed data). Comparisons among multiple groups were conducted using one-way analysis of variance (ANOVA) (for normally distributed data). Correlation analysis was performed using the Spearman rank correlation coefficient. All statistical analyses were conducted using R software (version 4.0.3) and GraphPad Prism software (version 9.0). *p* < 0.05 were considered statistically significant.

## Electronic supplementary material

Below is the link to the electronic supplementary material.

Supplementary Material 1

## Data Availability

RNA sequencing data and clinical information for all osteosarcoma samples were obtained from the TCGA database (https://portal.gdc.cancer.gov) and the GEO database (GSE56001, https://www.ncbi.nlm.nih.gov/geo/query/acc.cgi? acc=GSE56001 and GSE19276, https://www.ncbi.nlm.nih.gov/geo/query/acc.cgi? acc=GSE19276). All other data generated or analyzed are included in the manuscript and the Supplementary material.
