# Supplementary material for: S100 A16 promotes the progression of osteosarcoma by activating the PI3 K/AKT signaling pathway through ANXA2
Source: Sci Rep. 2025 Jun 6;15:19962. doi: 10.1038/s41598-025-05293-6 (PMC12144199; doi:10.1038/s41598-025-05293-6)

Fig 1 WB(1)
GAPDH

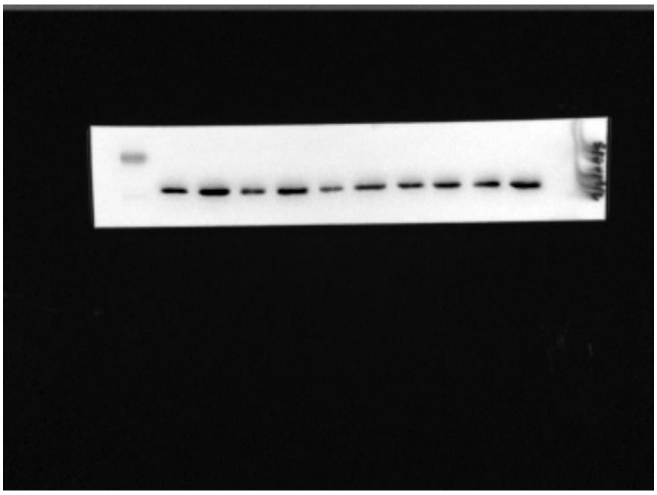

S100A16

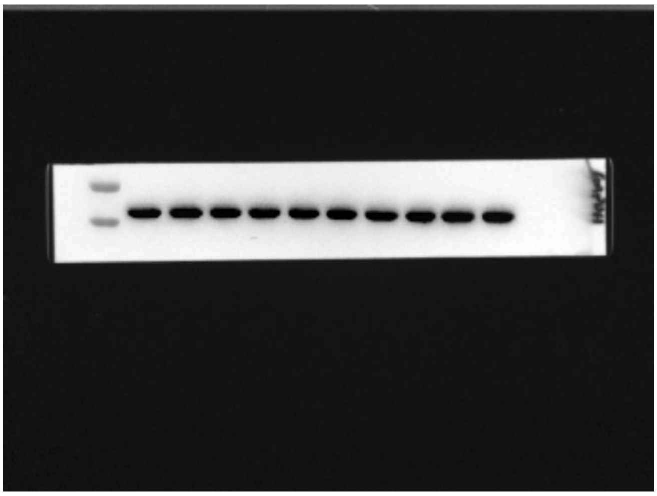

Fig 1 WB(2)
GAPDH

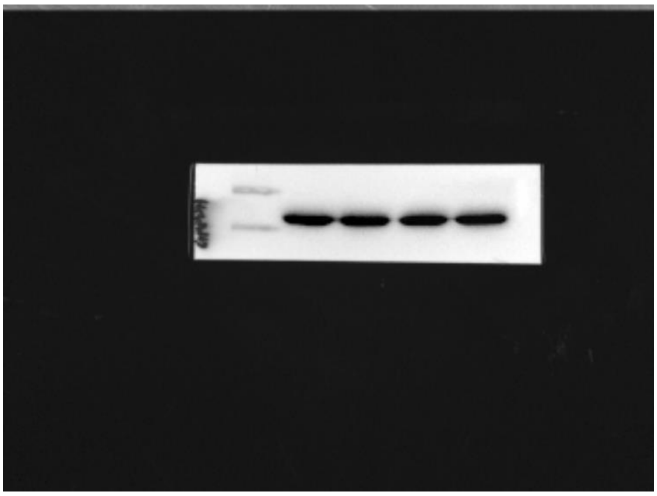

S100A16

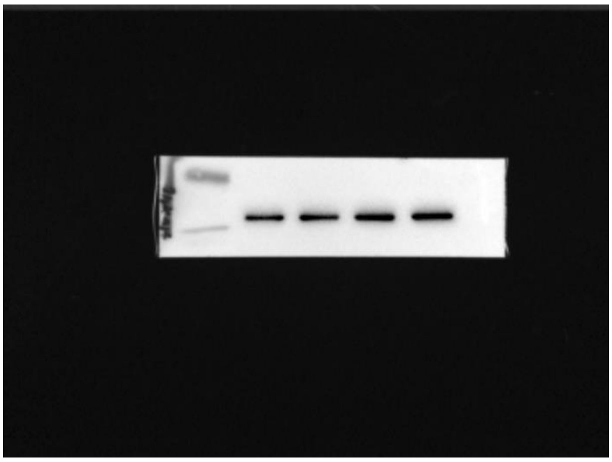

Fig 2 scratch

MG-63 OE 0h 100-1

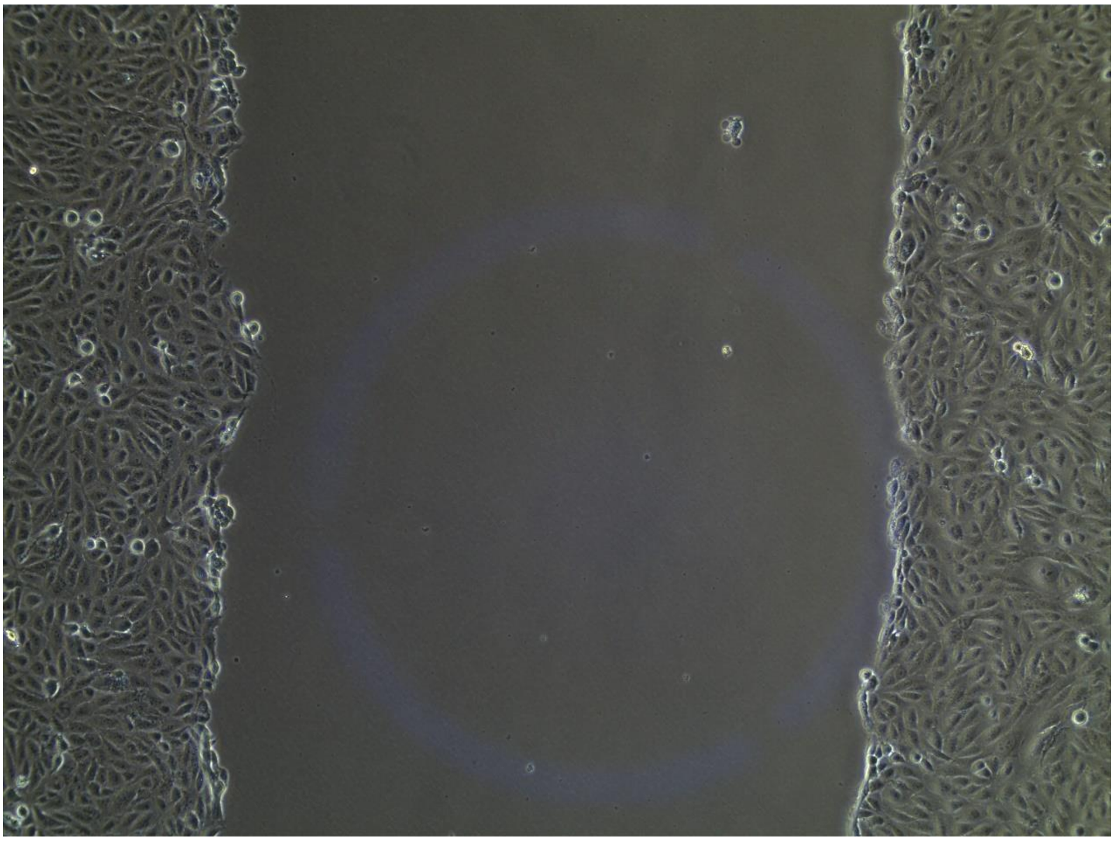

MG-63 OE 0h 100-2

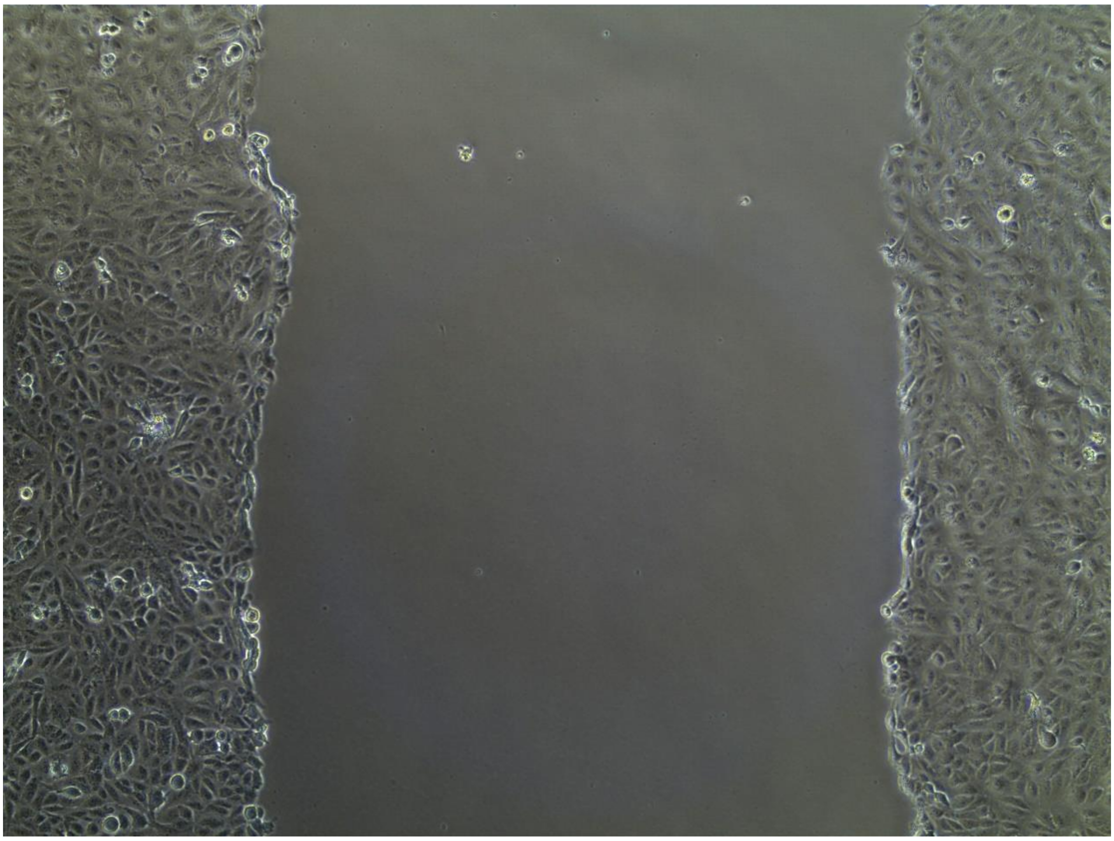

MG-63 OE 0h 100-3

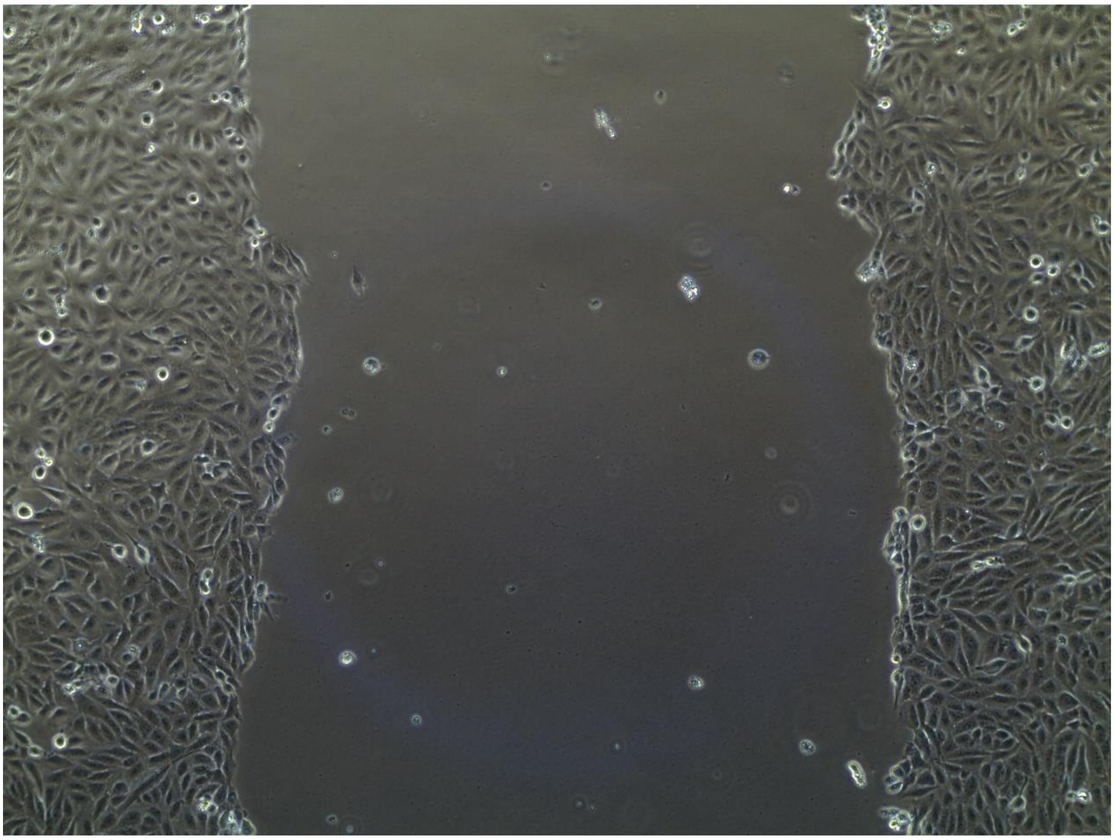

MG-63 OE 24h 100-1

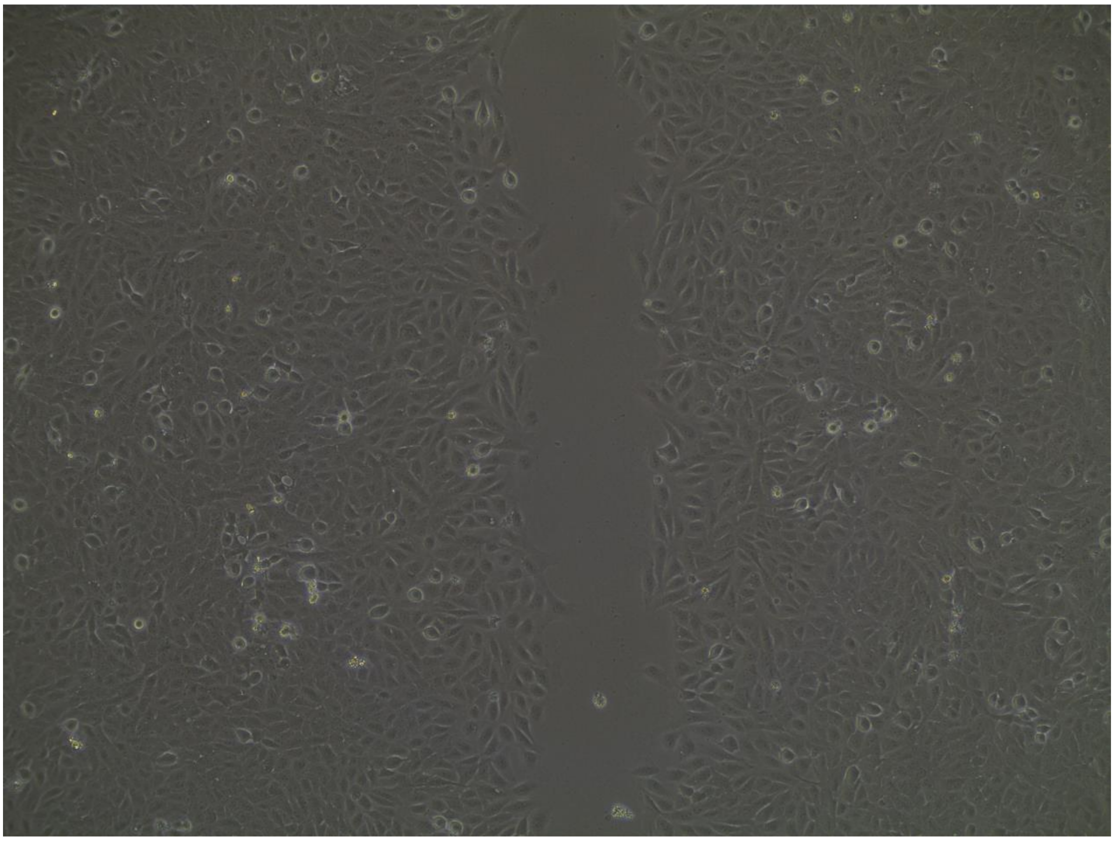

MG-63 OE 24h 100-2

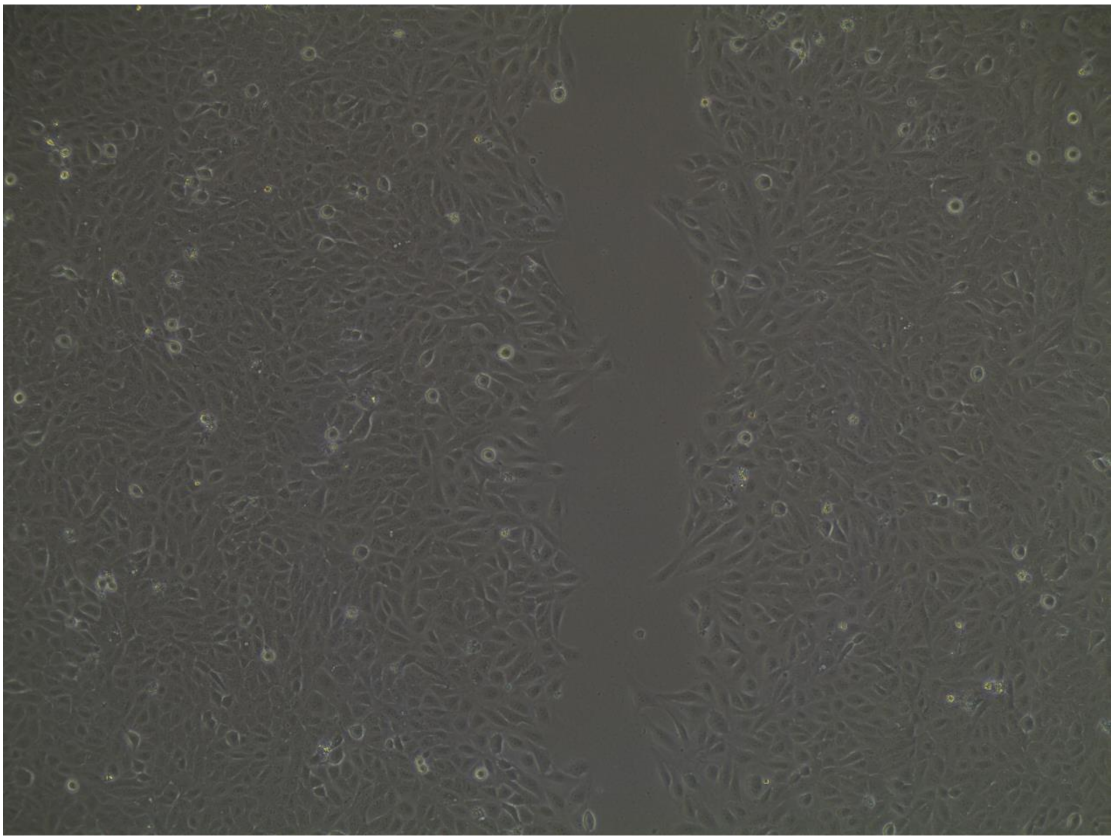

MG-63 OE 24h 100-3

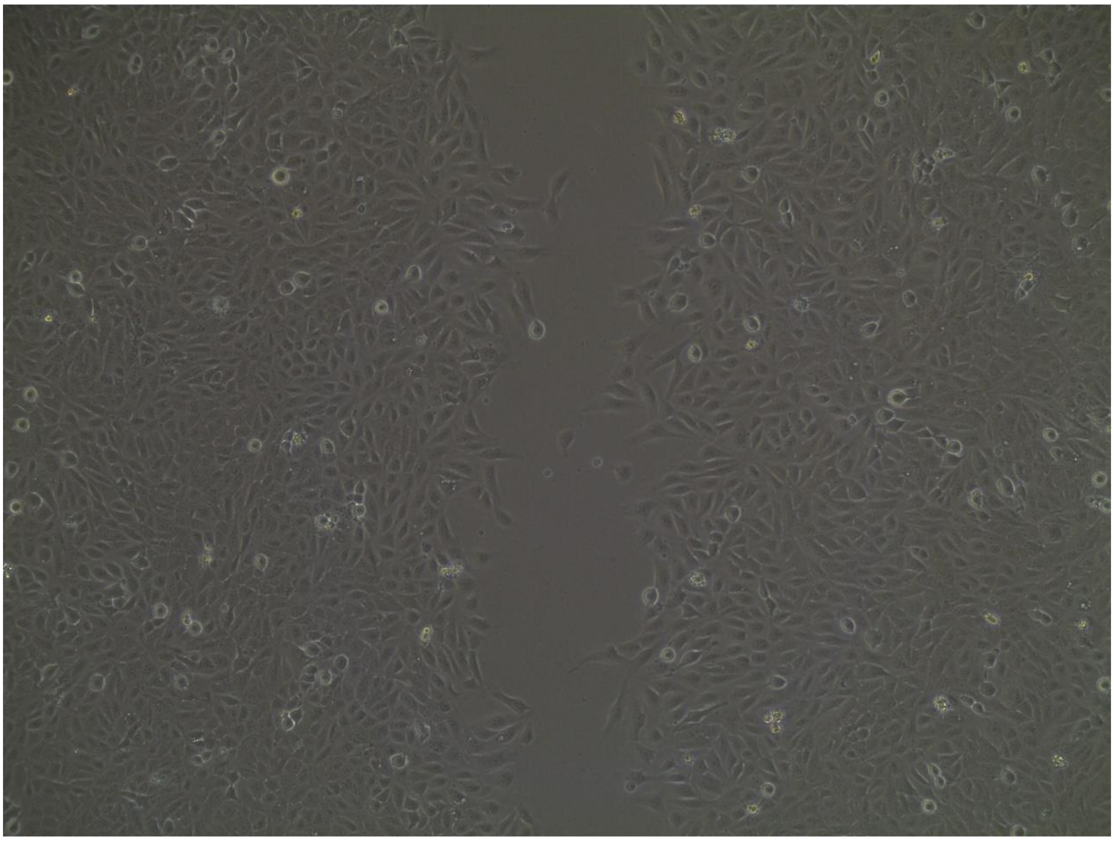

MG-63 vector 0h 100-1

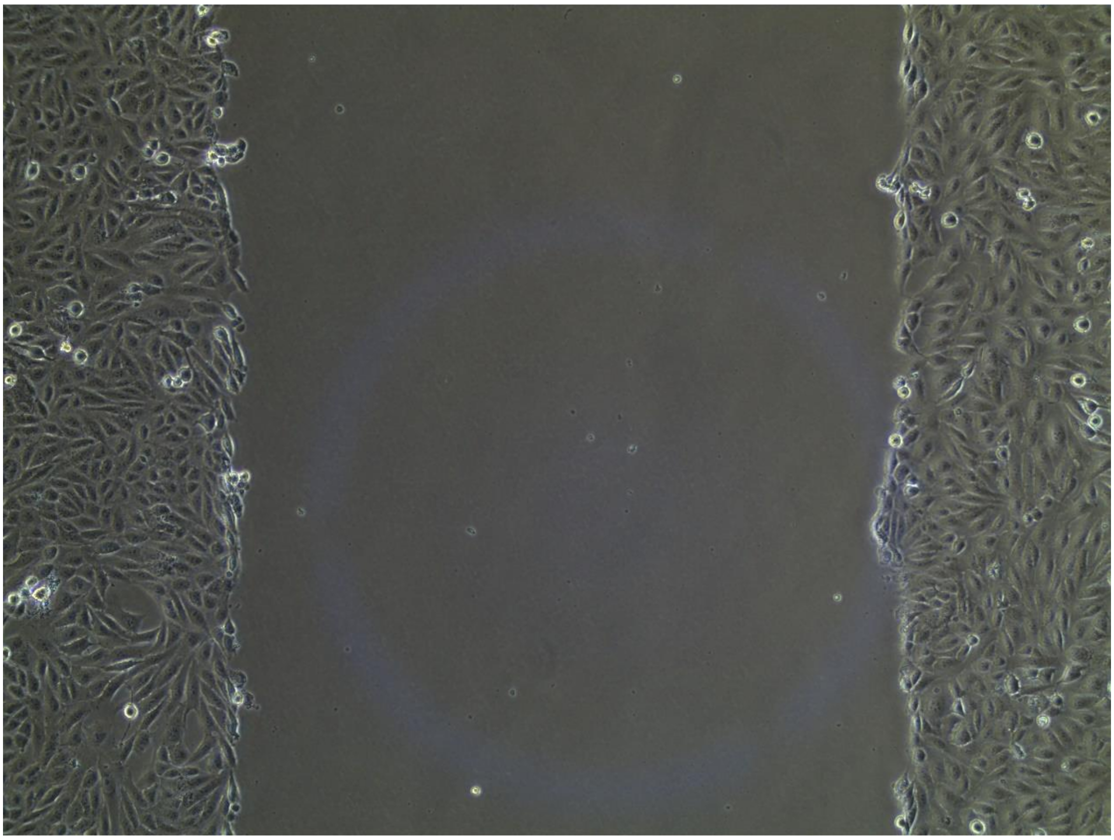

MG-63 vector 0h 100-2

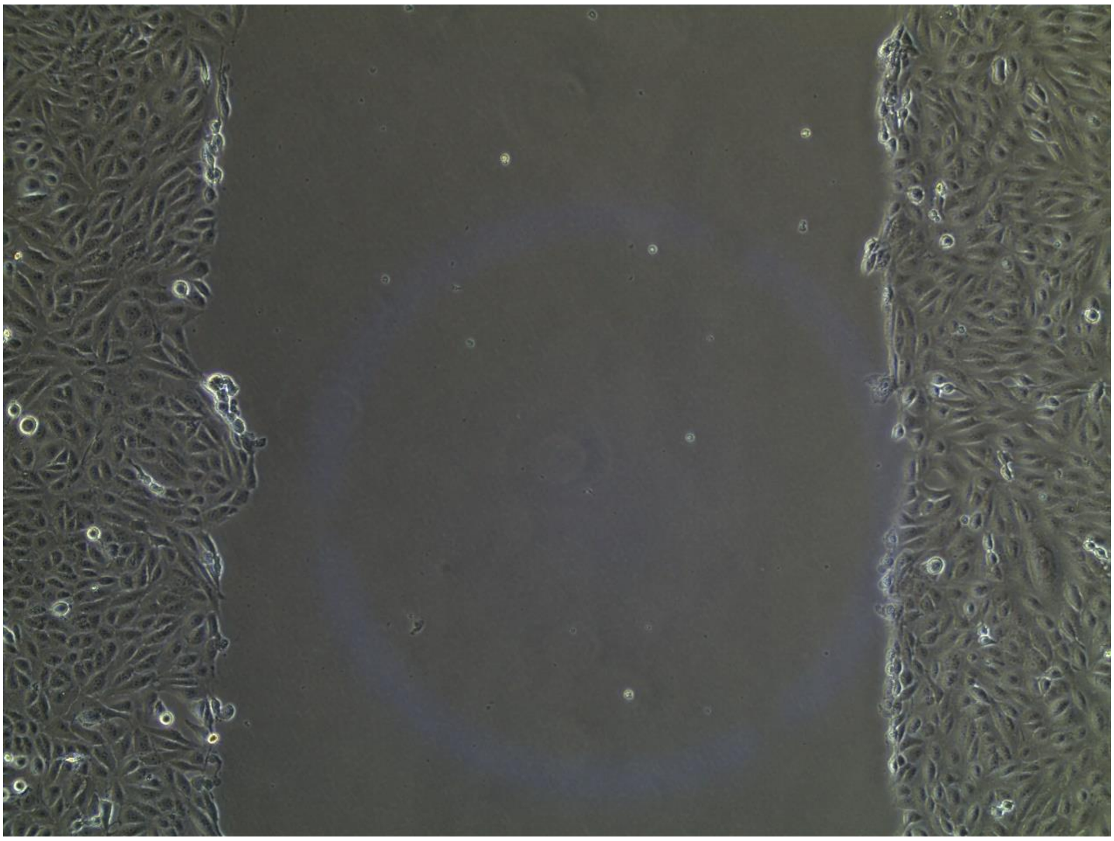

MG-63 vector 0h 100-3

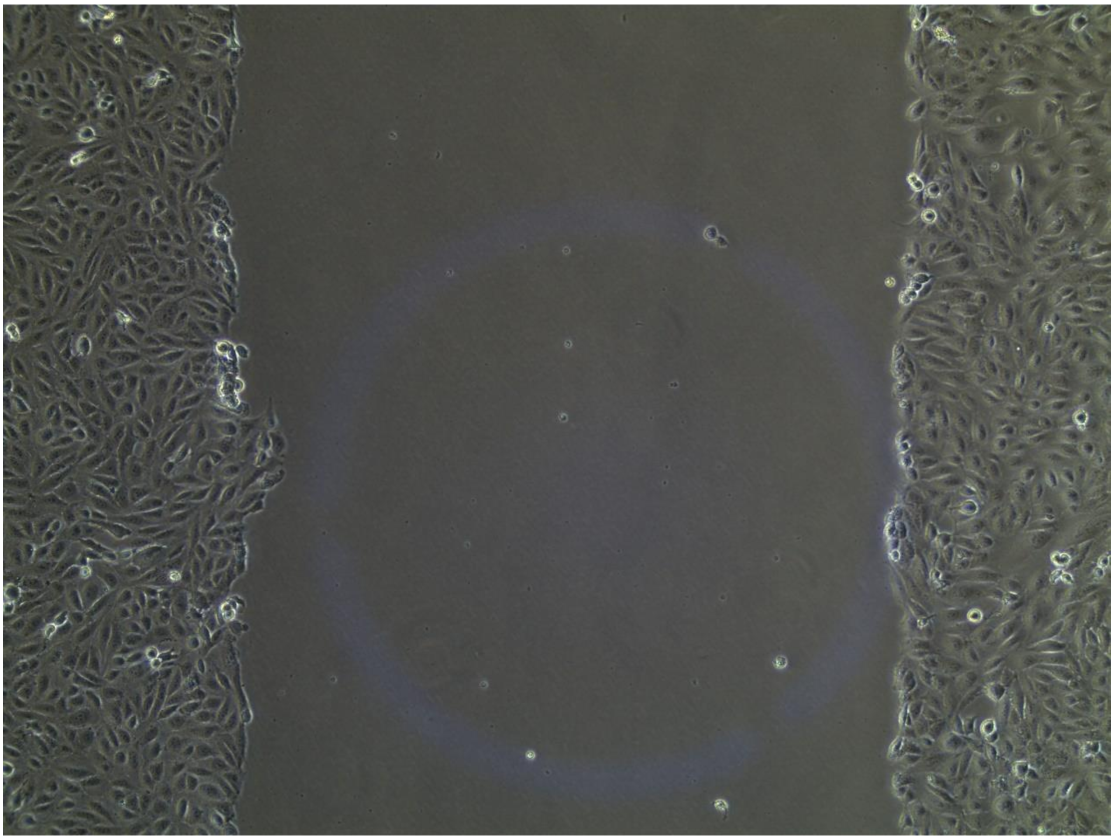

MG-63 vector 24h 100-1

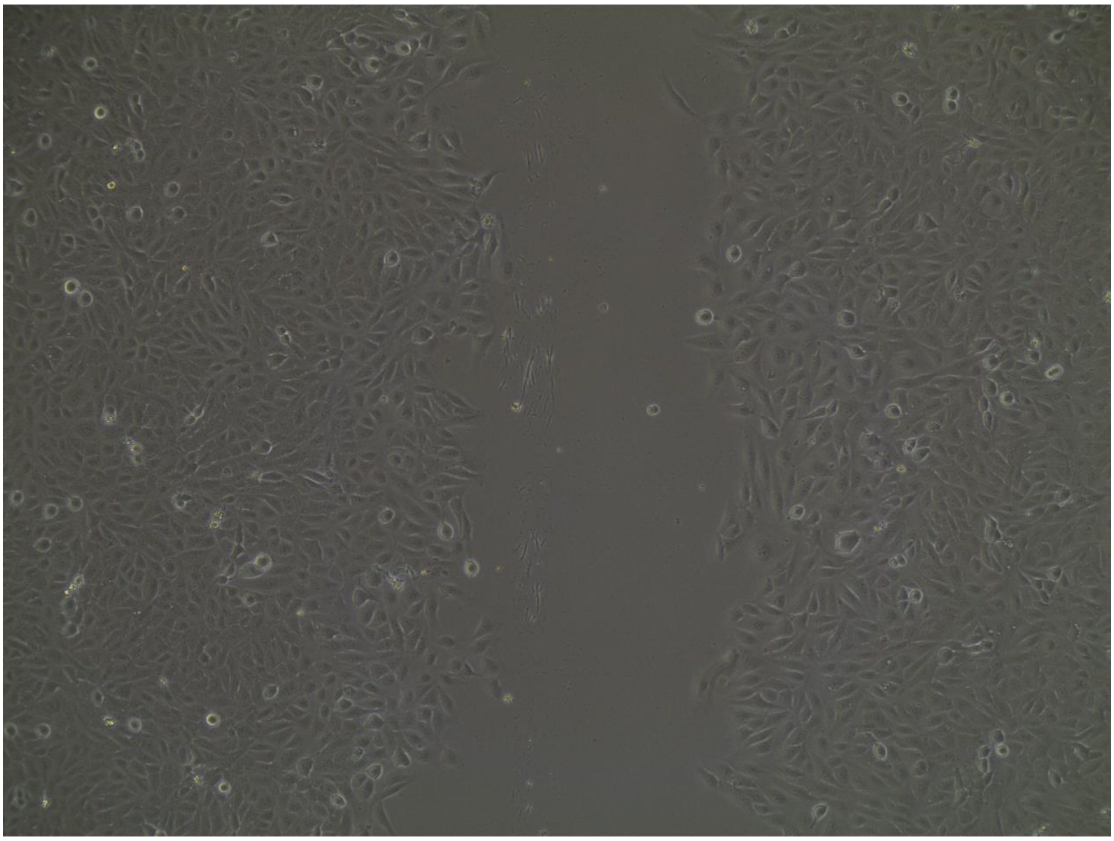

MG-63 vector 24h 100-2

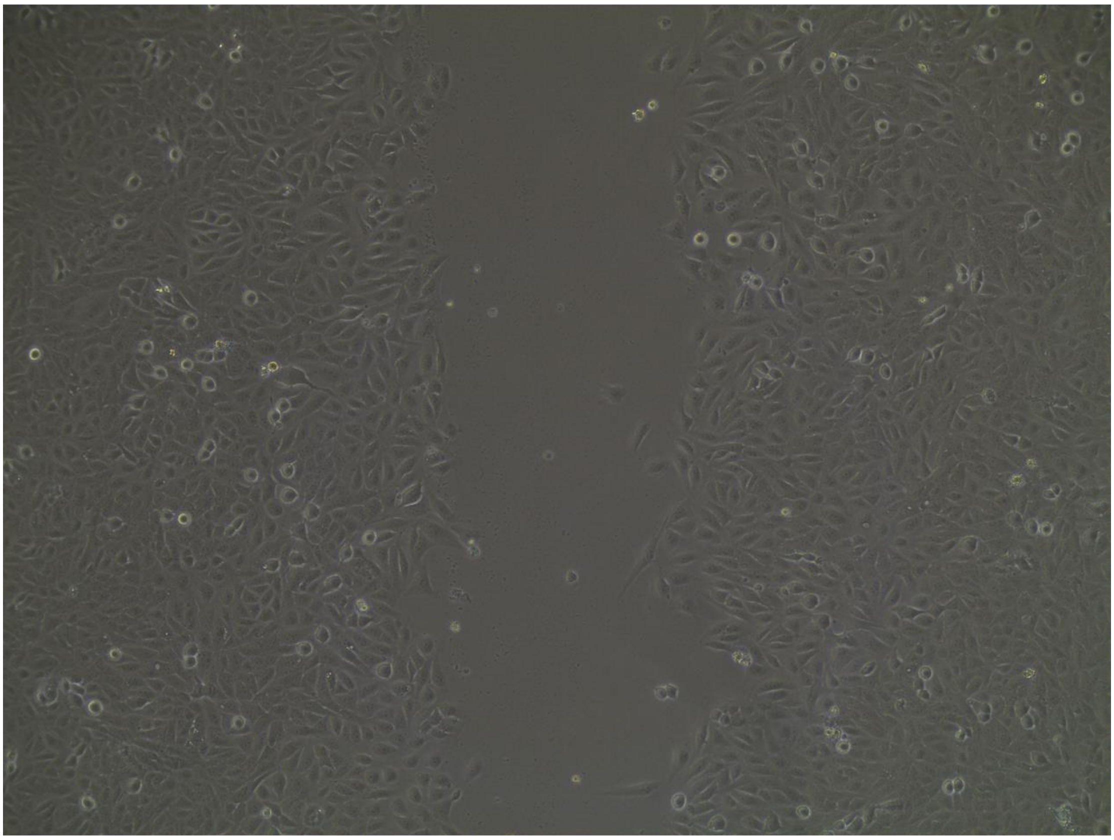

MG-63 vector 24h 100-3

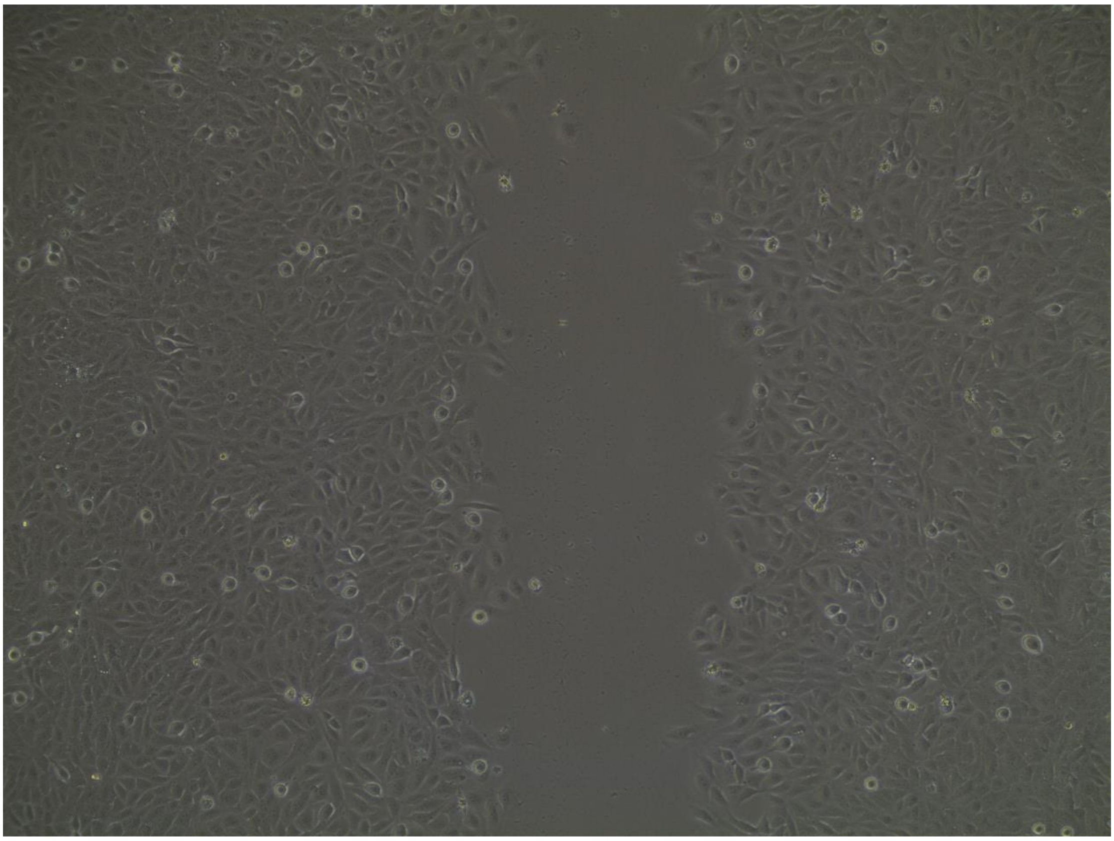

U20S NC 0h 100-1

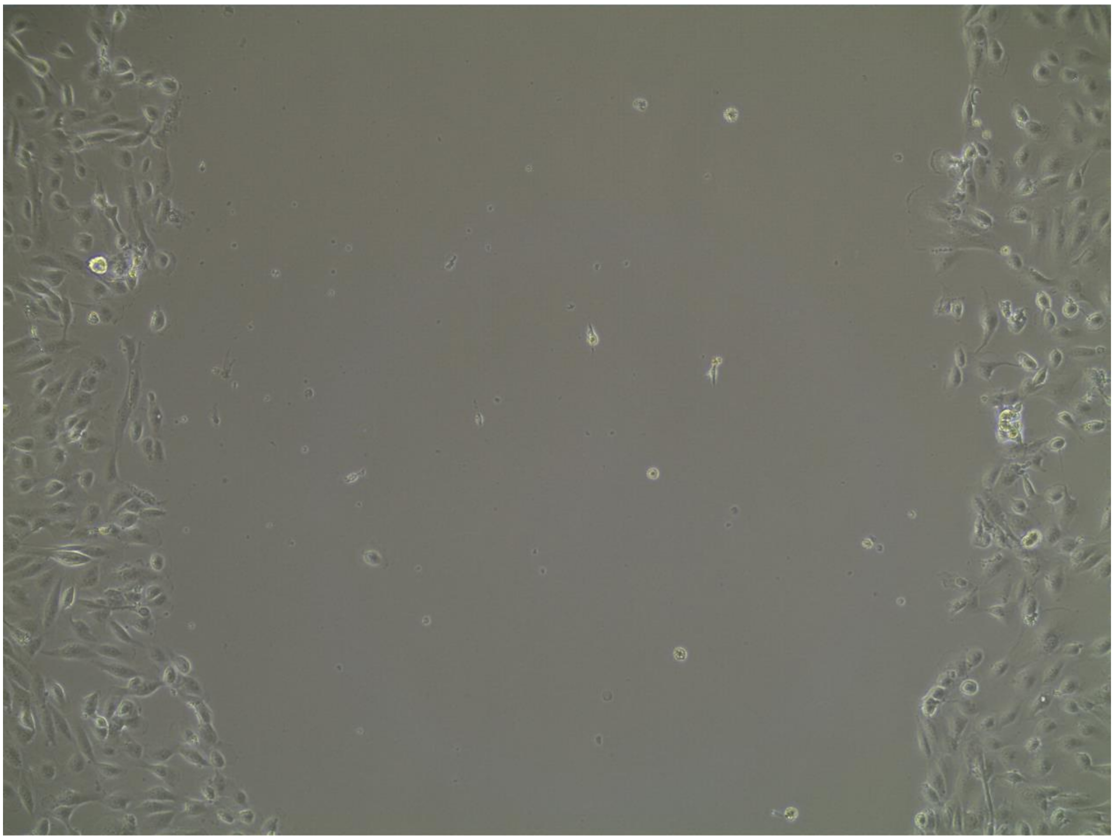

U20S NC 0h 100-2

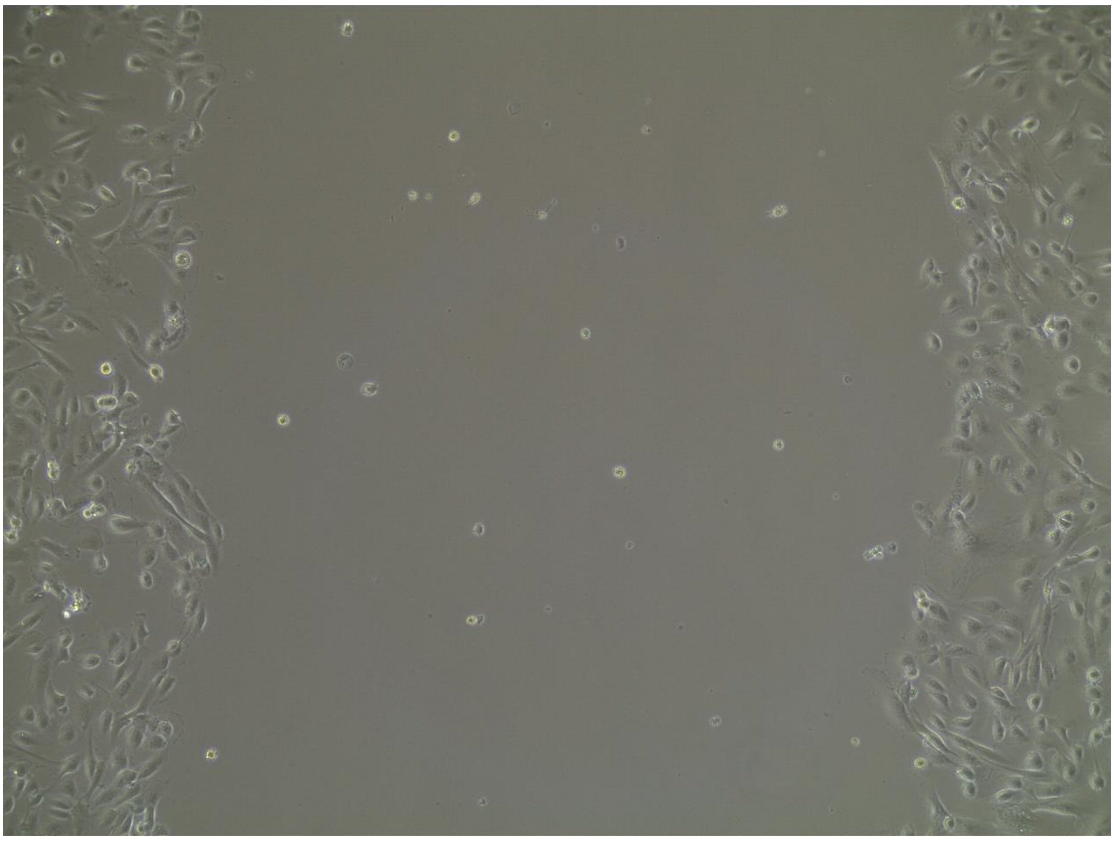

U20S NC 0h 100-3

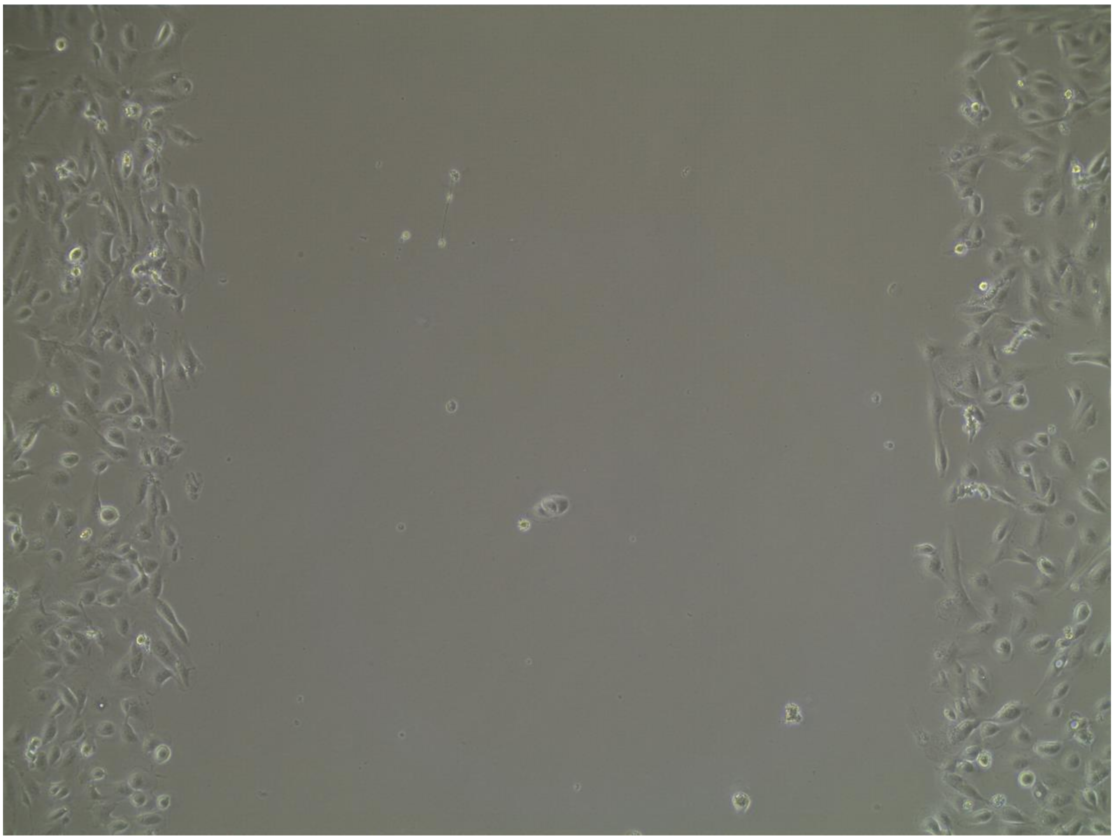

U20S NC 24h 100-1

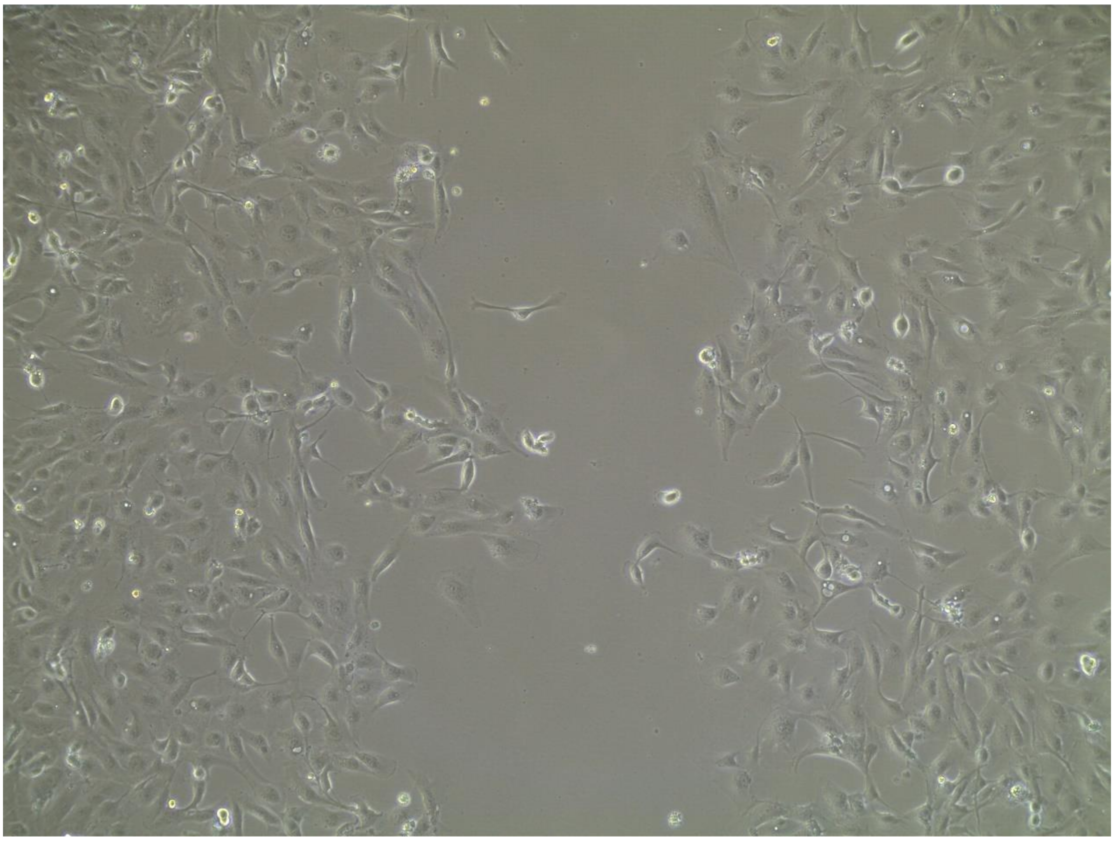

U20S NC 24h 100-2

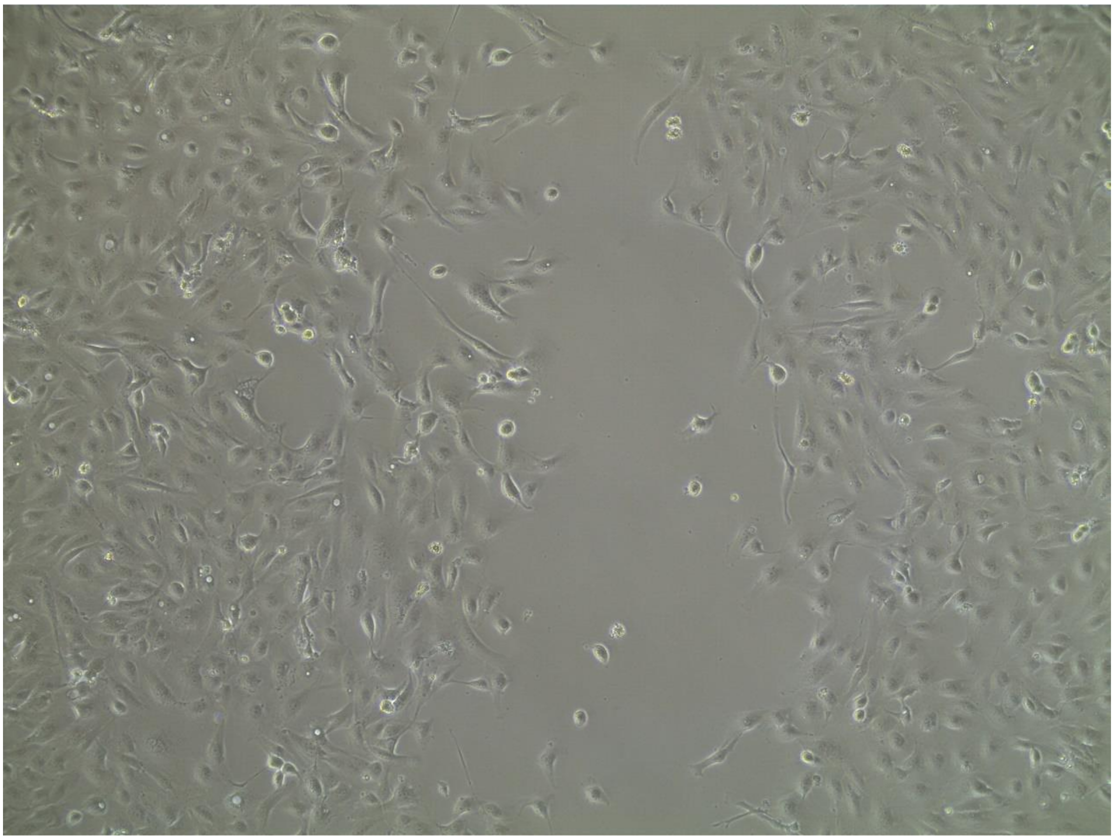

U20S NC 24h 100-3

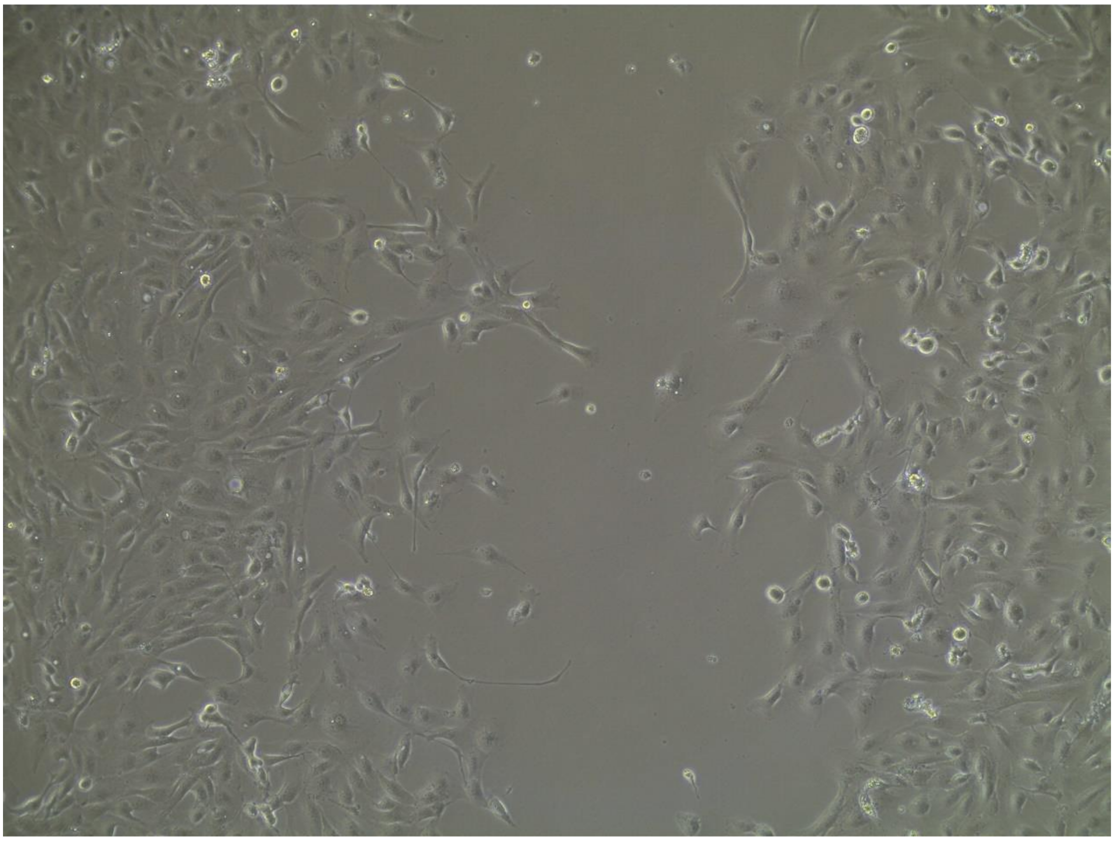

U20S OE 0h 100-1

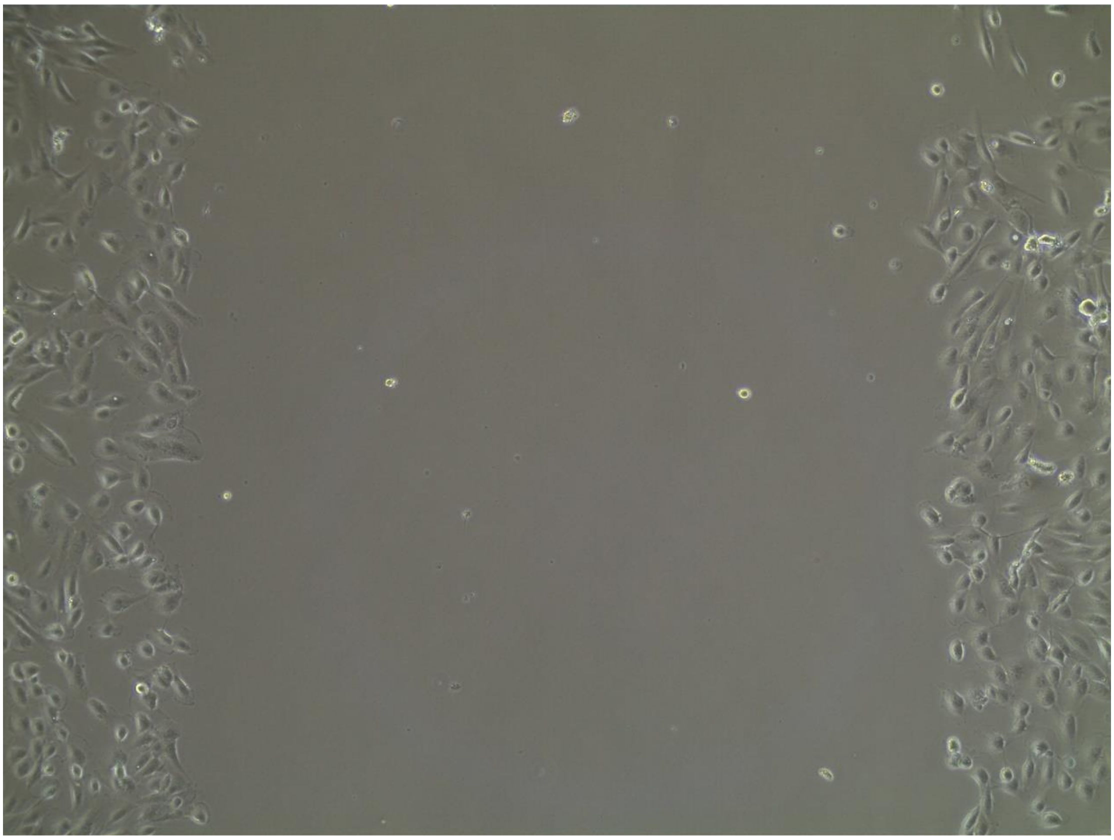

U20S OE 0h 100-2

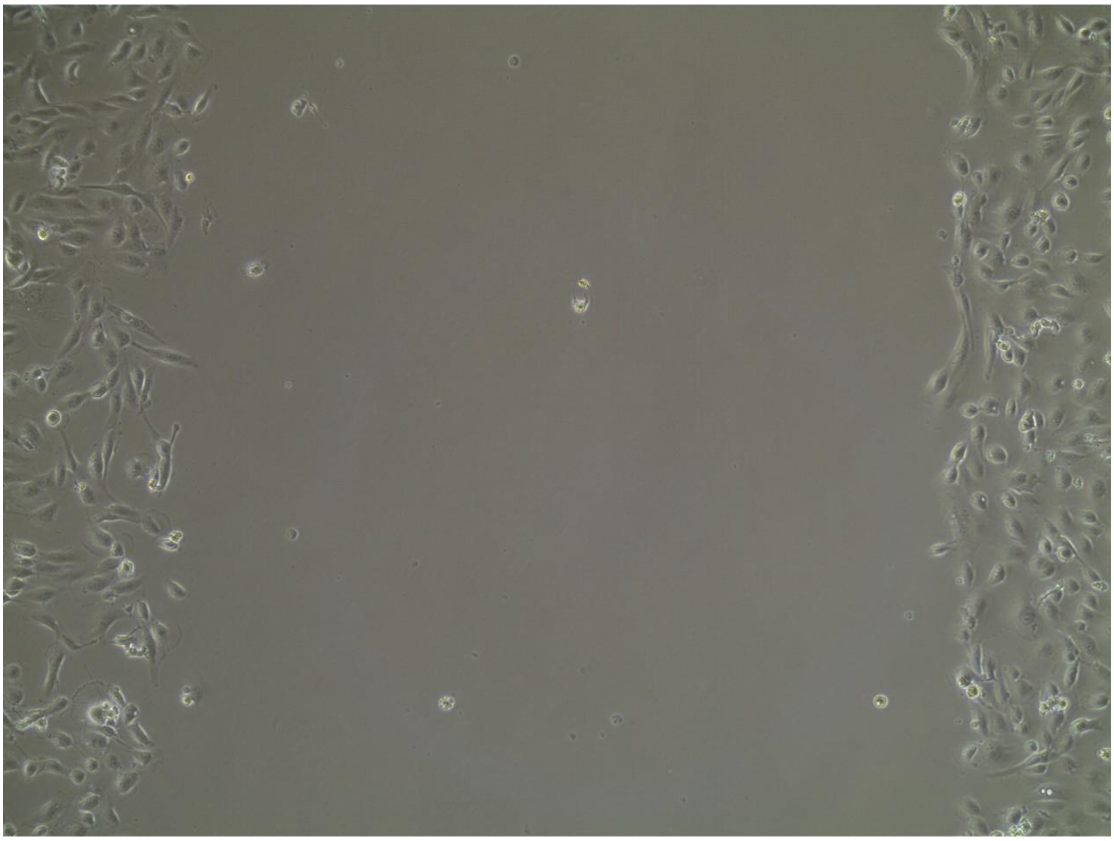

U20S OE 0h 100-3

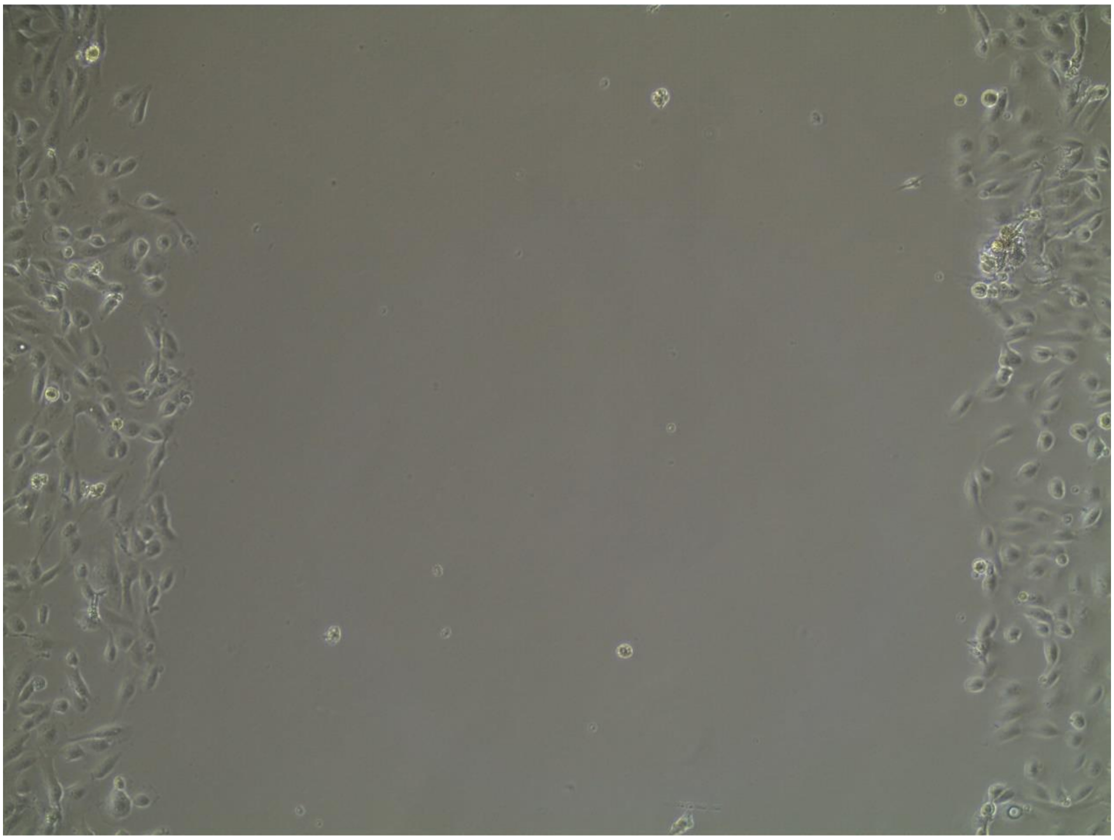

U20S OE 24h 100-1

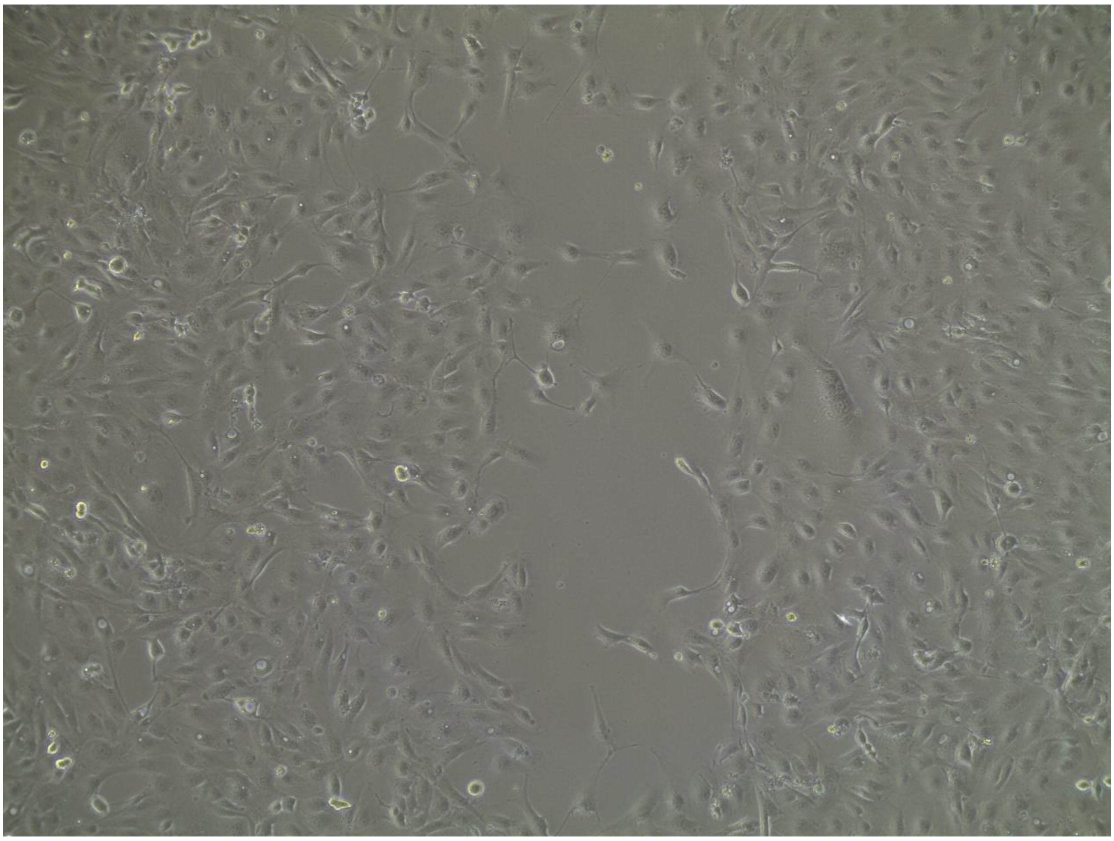

U20S OE 24h 100-2

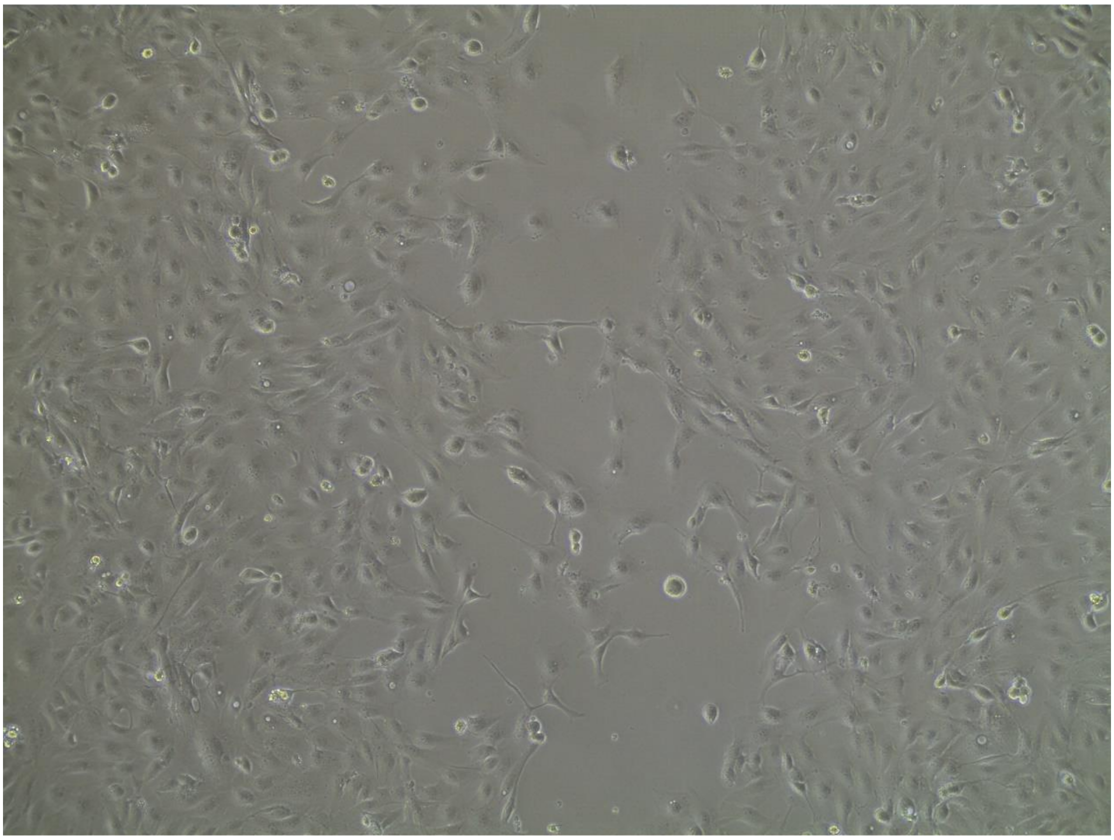

U20S OE 24h 100-3

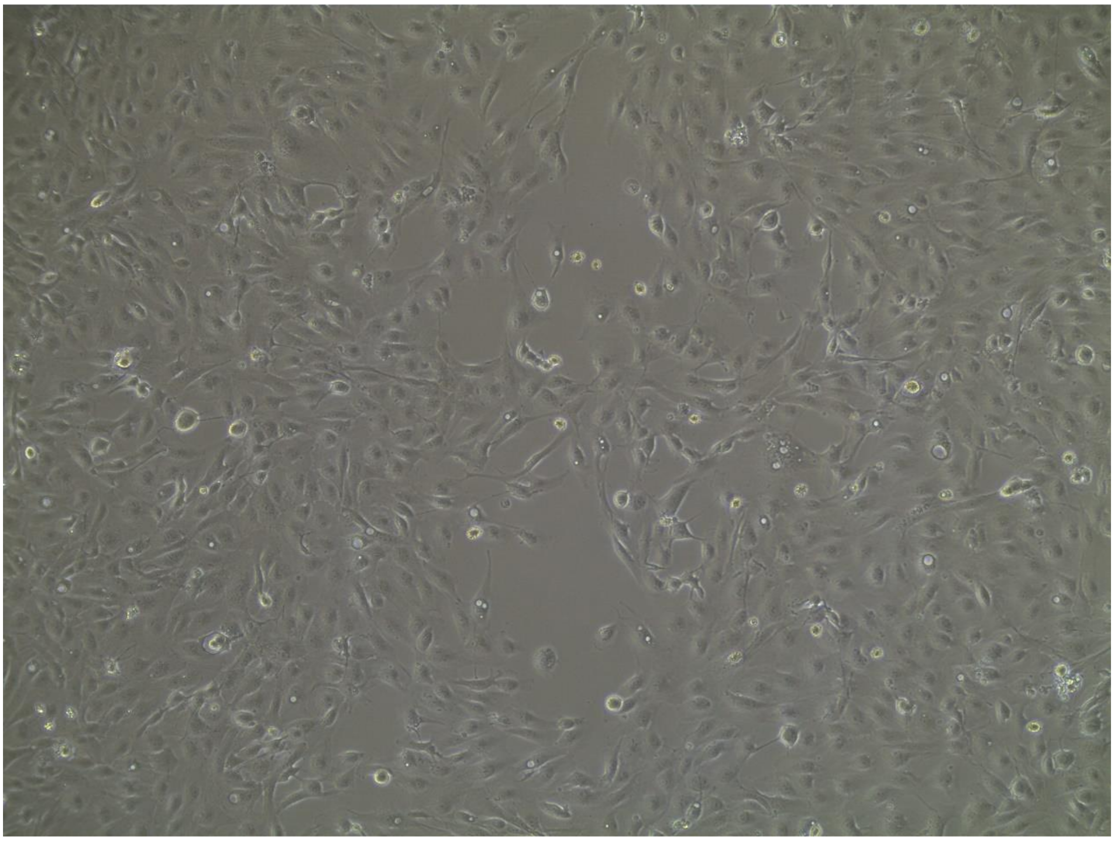

Fig 2 transwell
MG63 NC 200-1

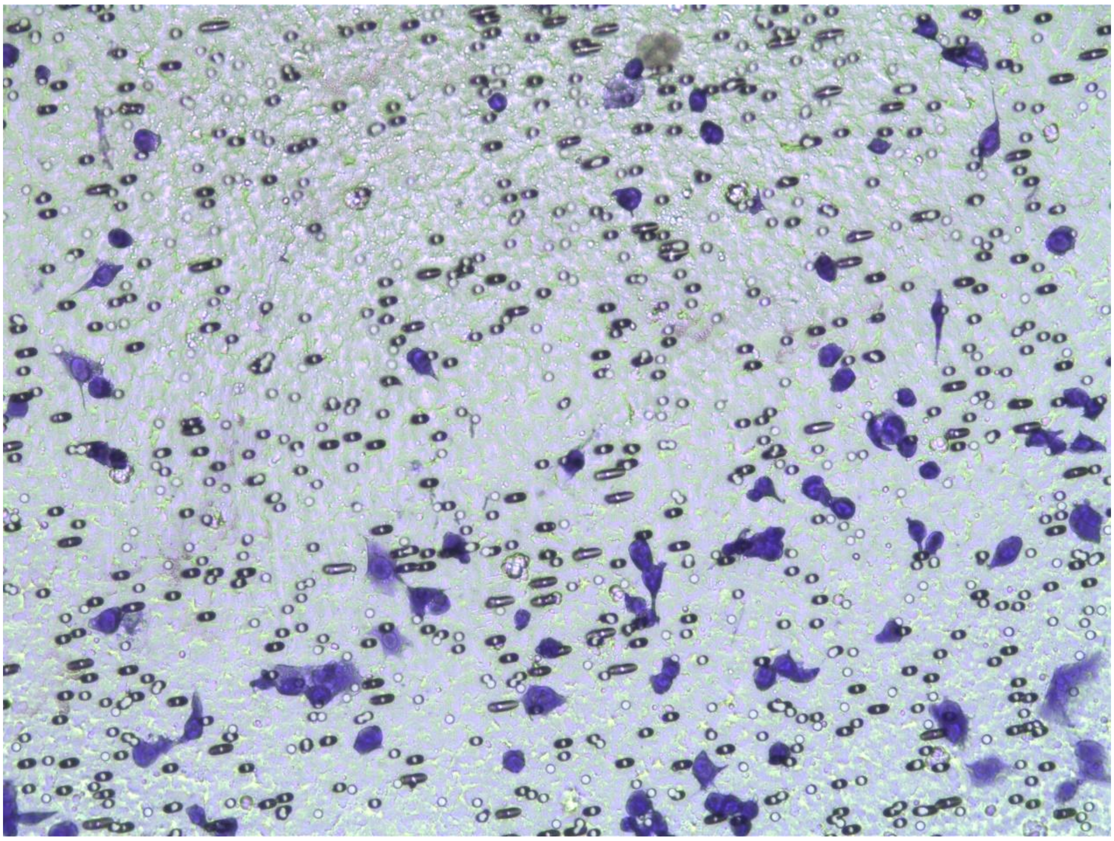

MG63 NC 200-2

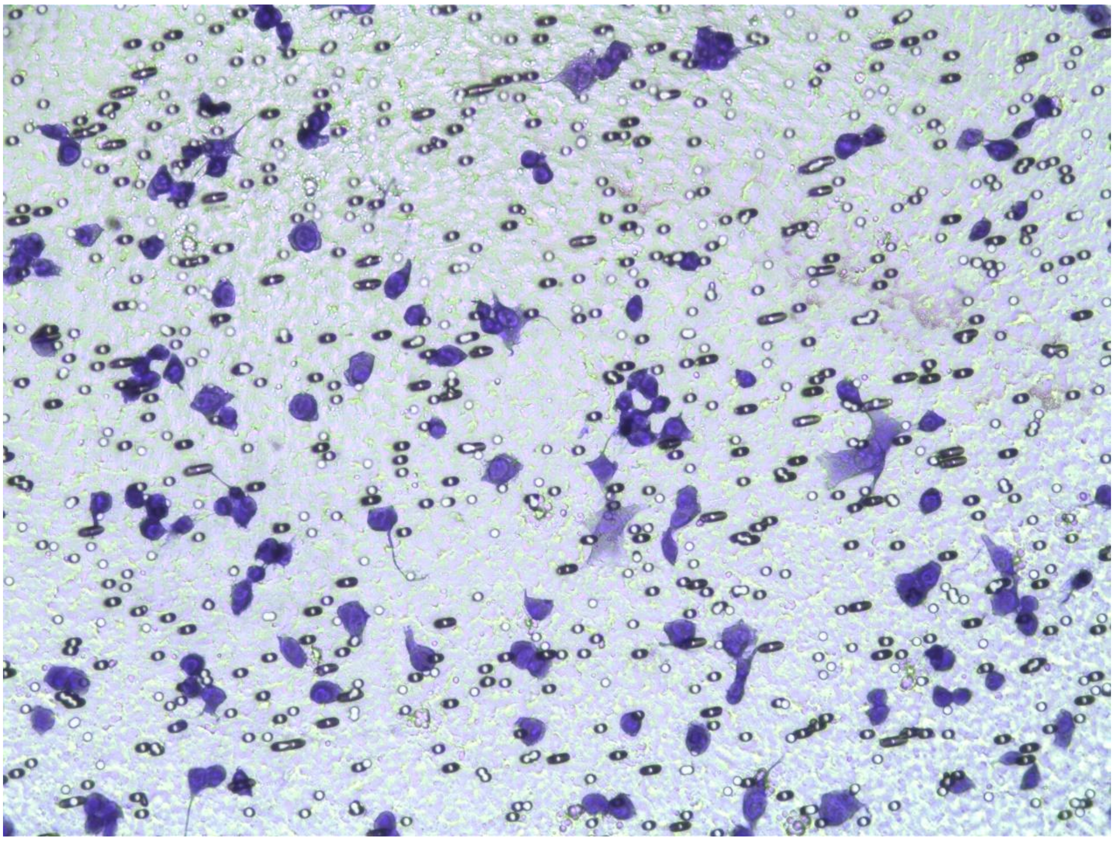

MG63 NC 200-3

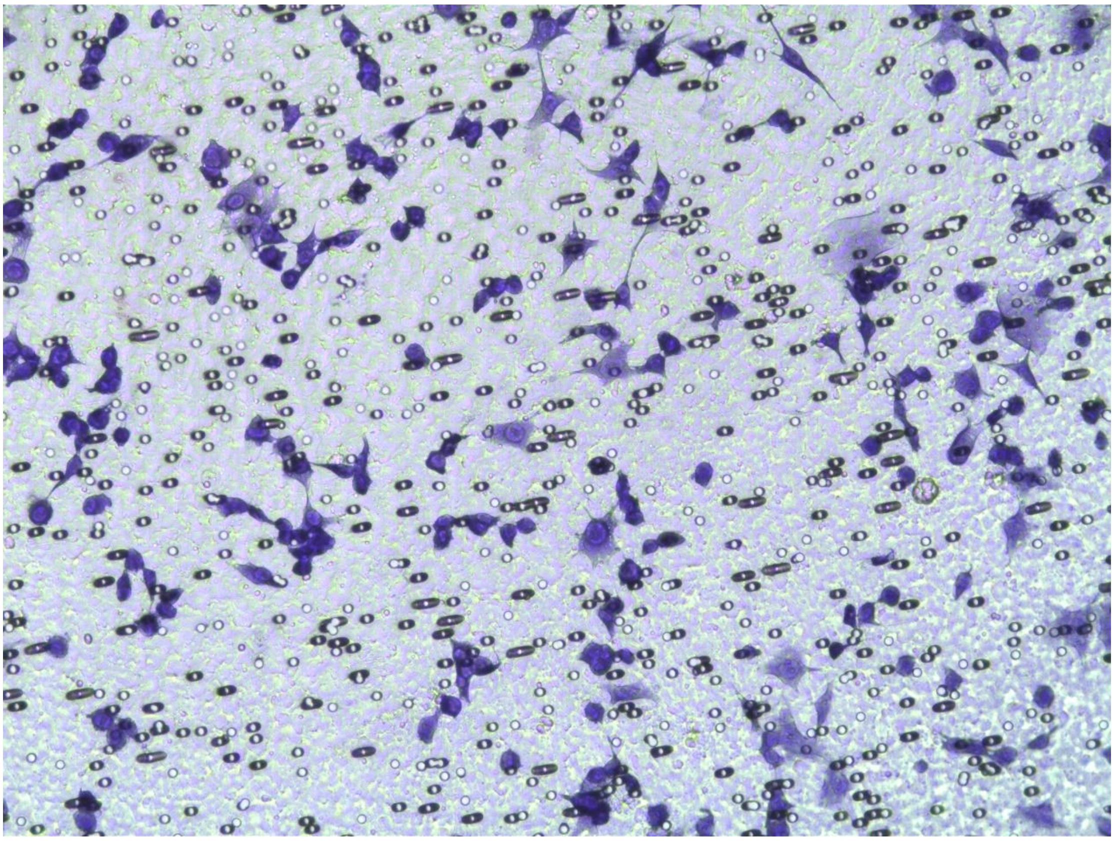

MG63 NC 200-4

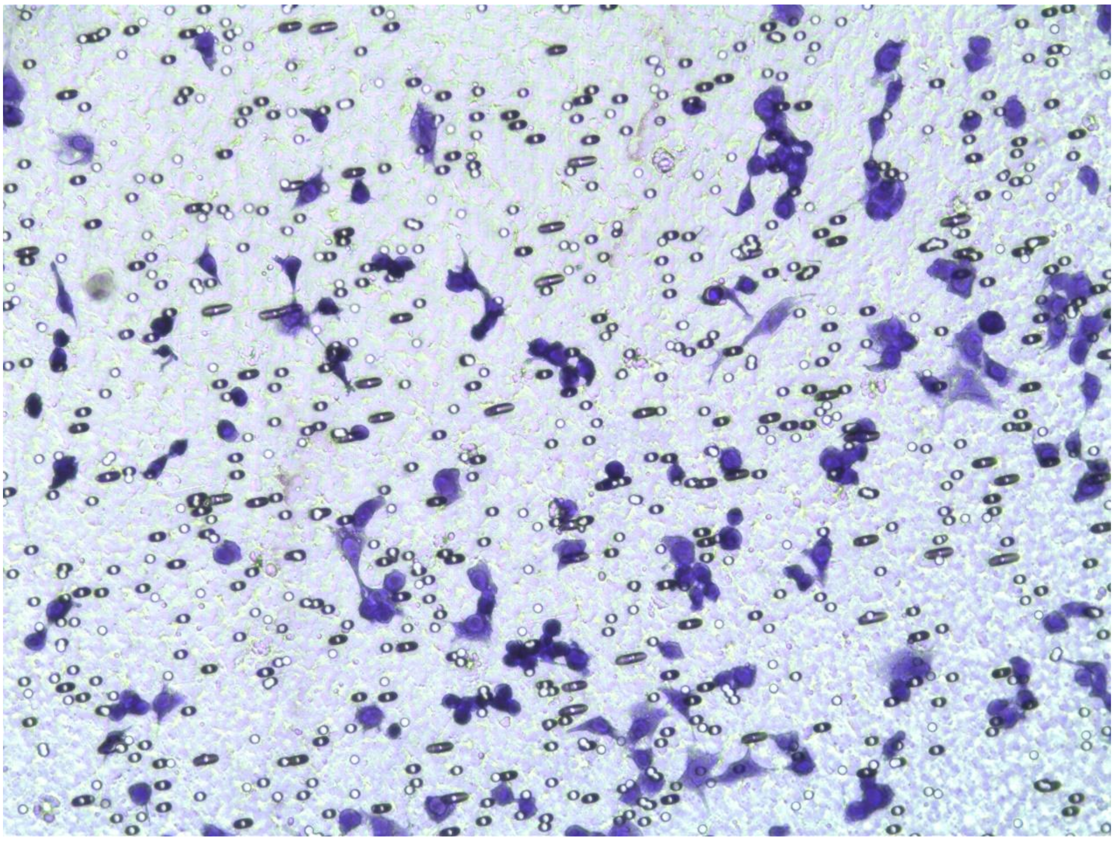

MG63 NC 200-5

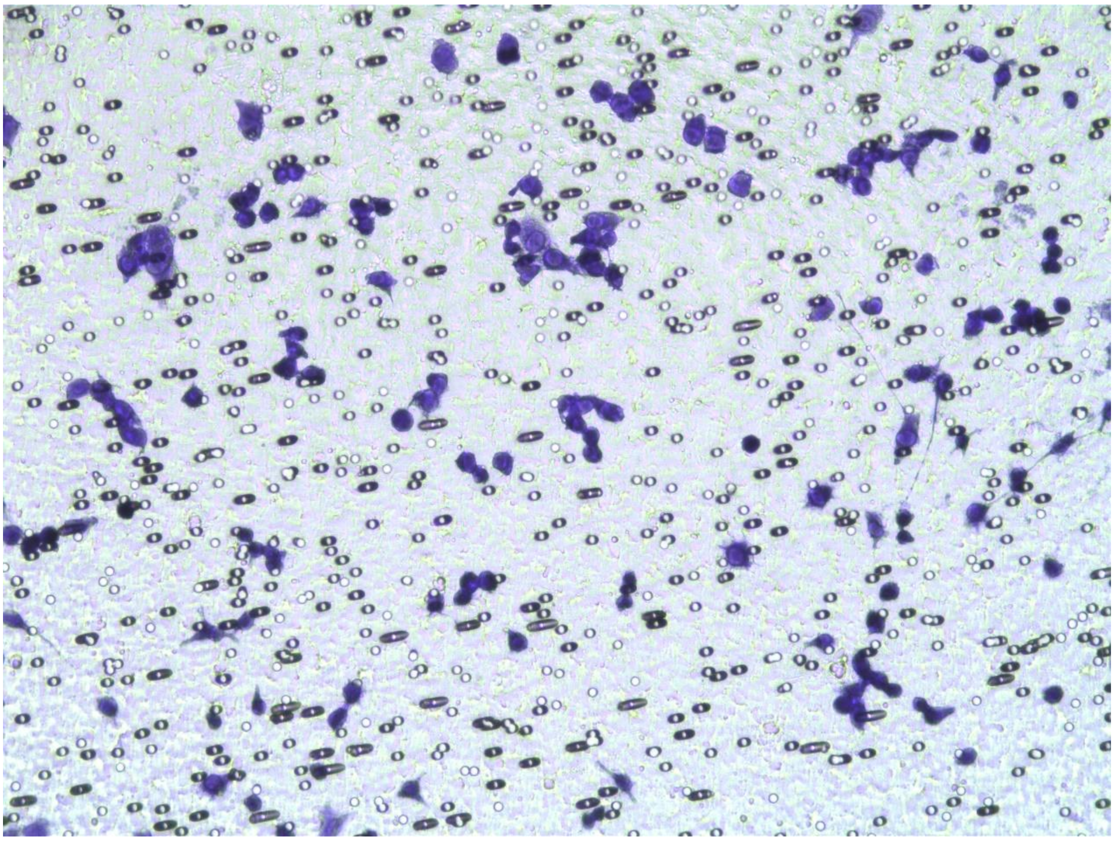

MG63 OE 200-1

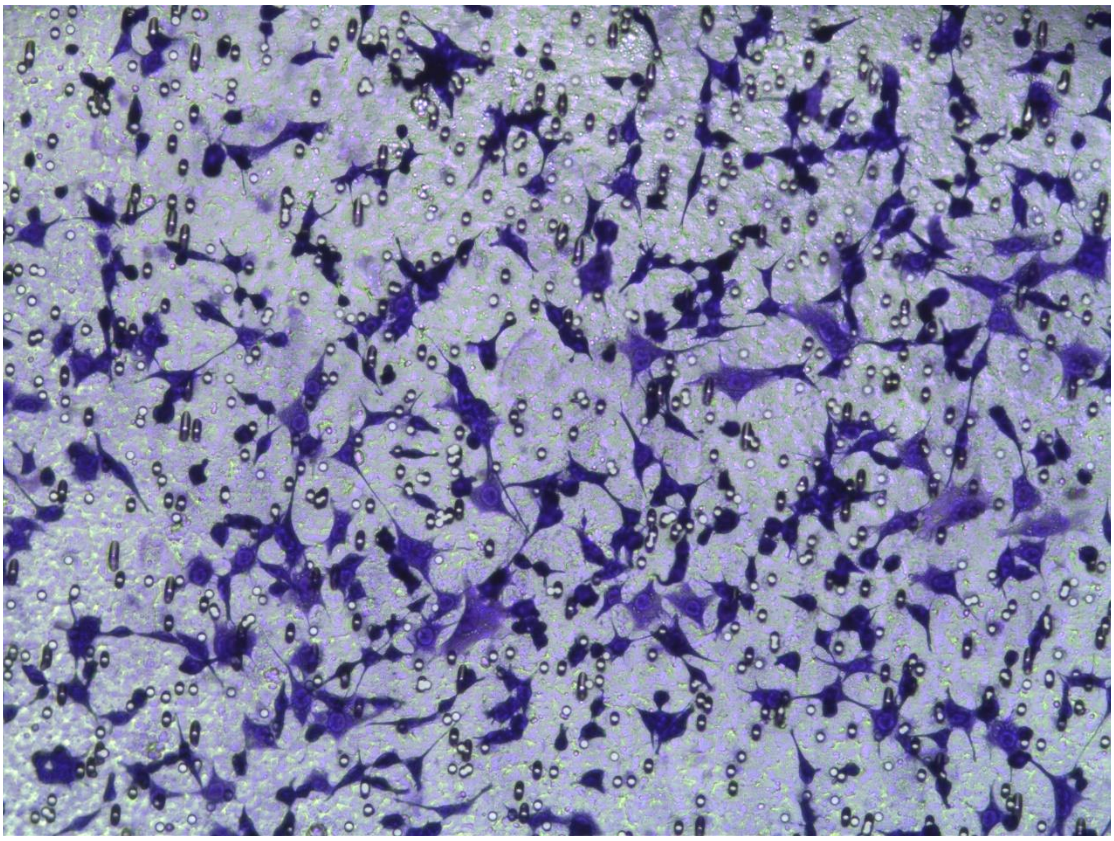

MG63 OE 200-2

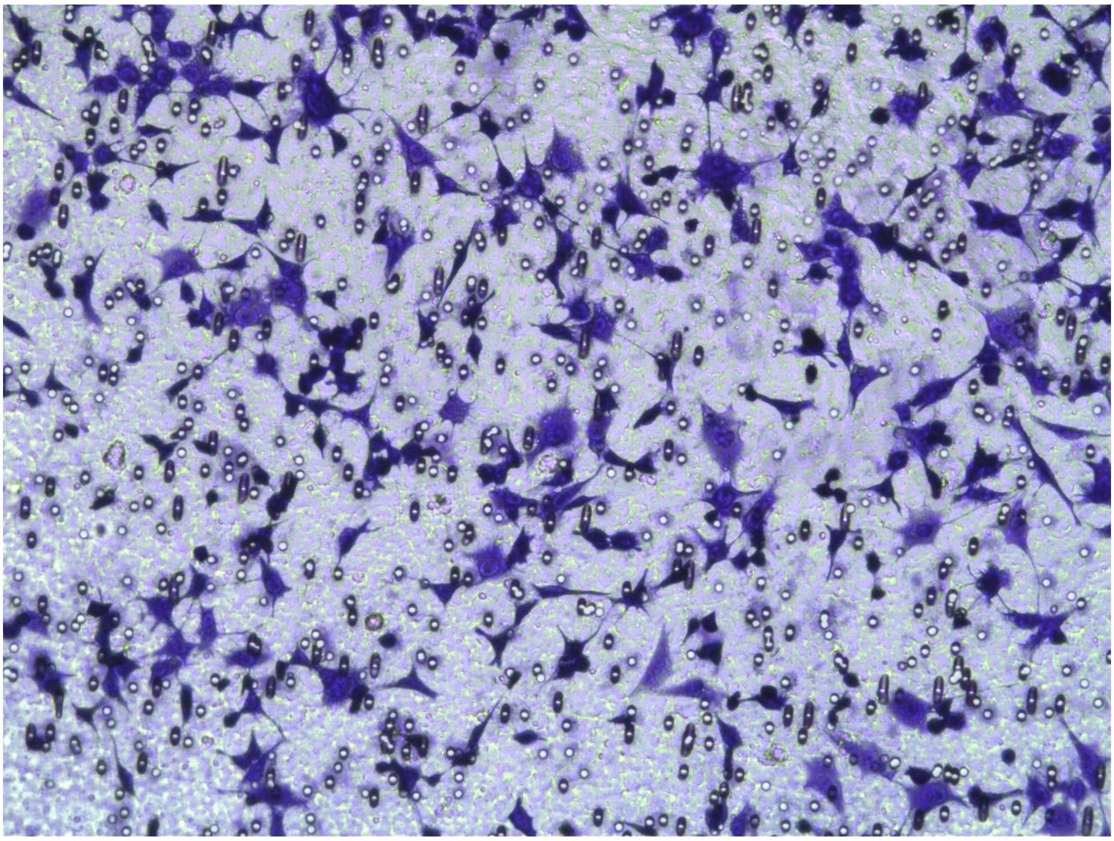

MG63 OE 200-3

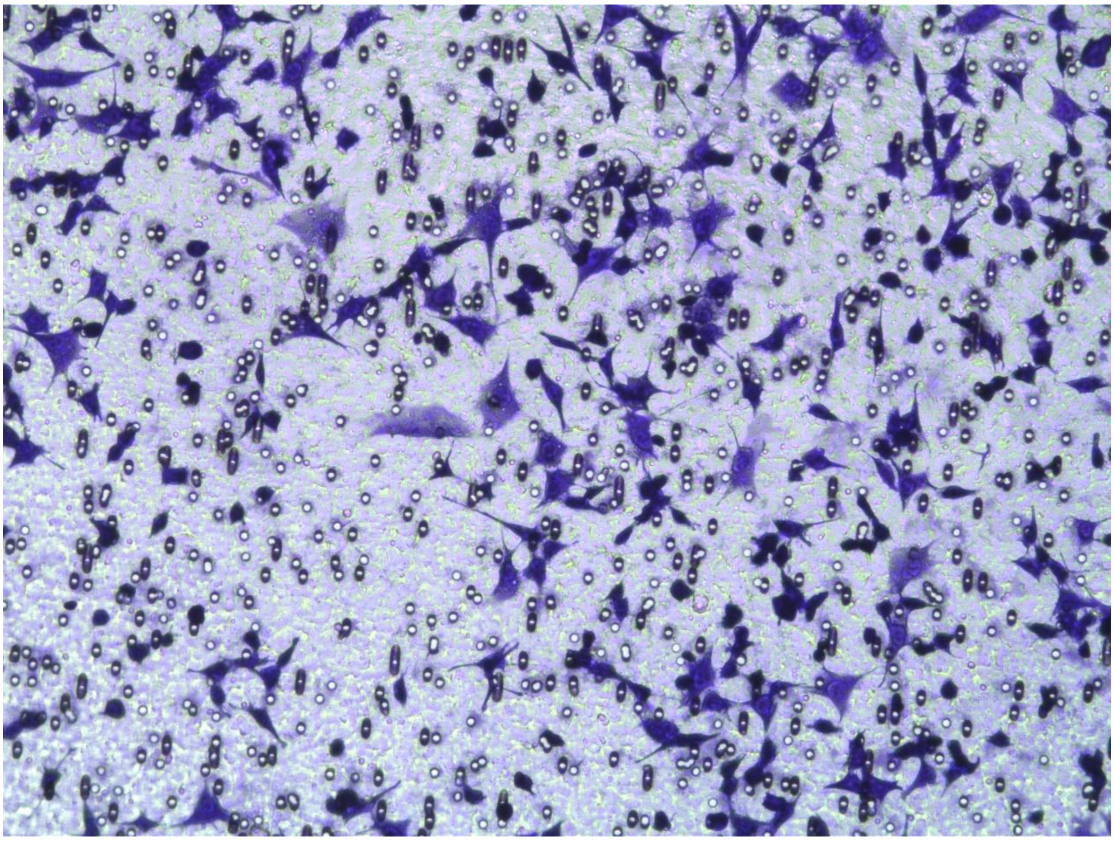

MG63 OE 200-4

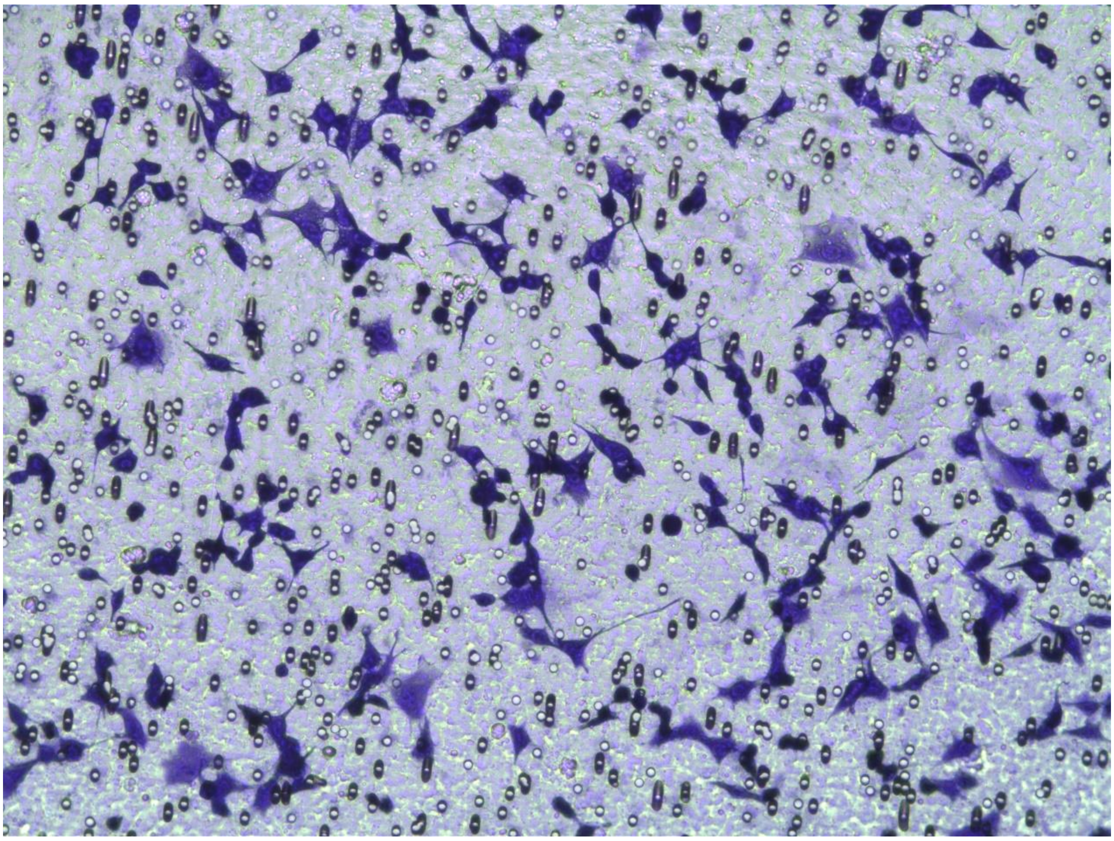

MG63 OE 200-5

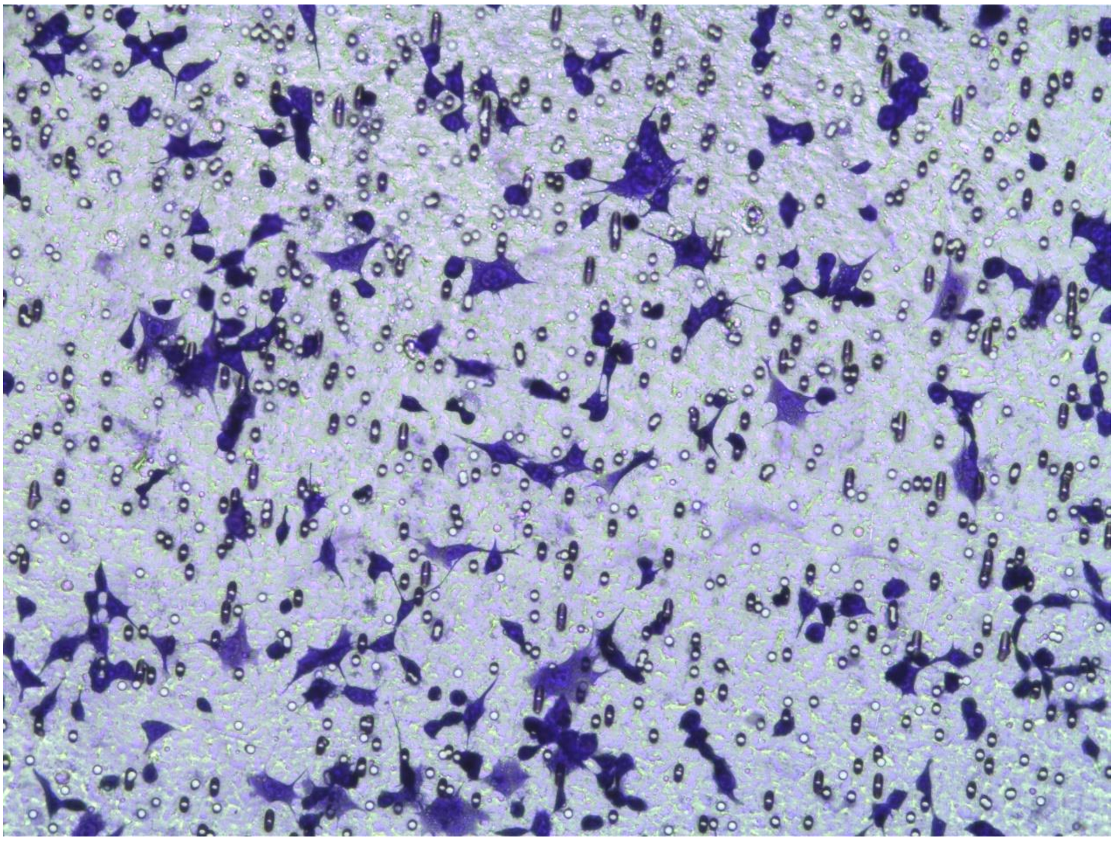

U20S NC 200-1

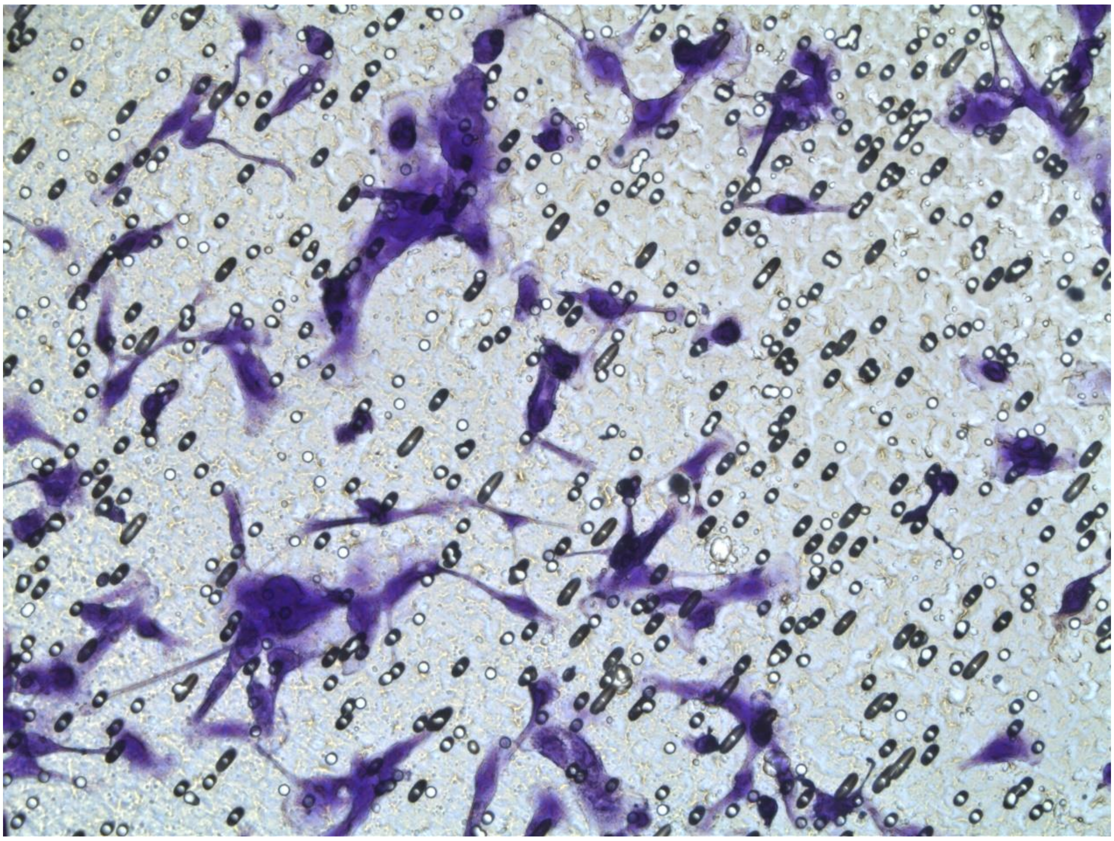

U20S NC 200-2

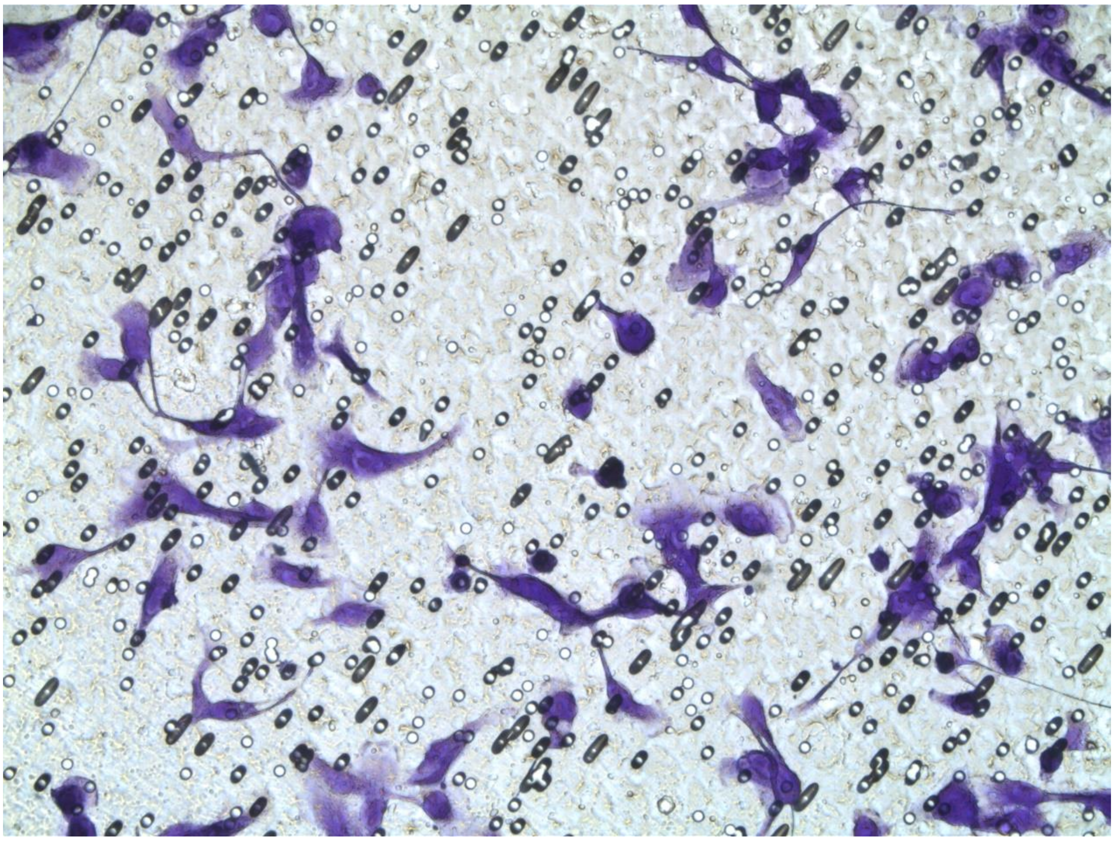

U20S NC 200-3

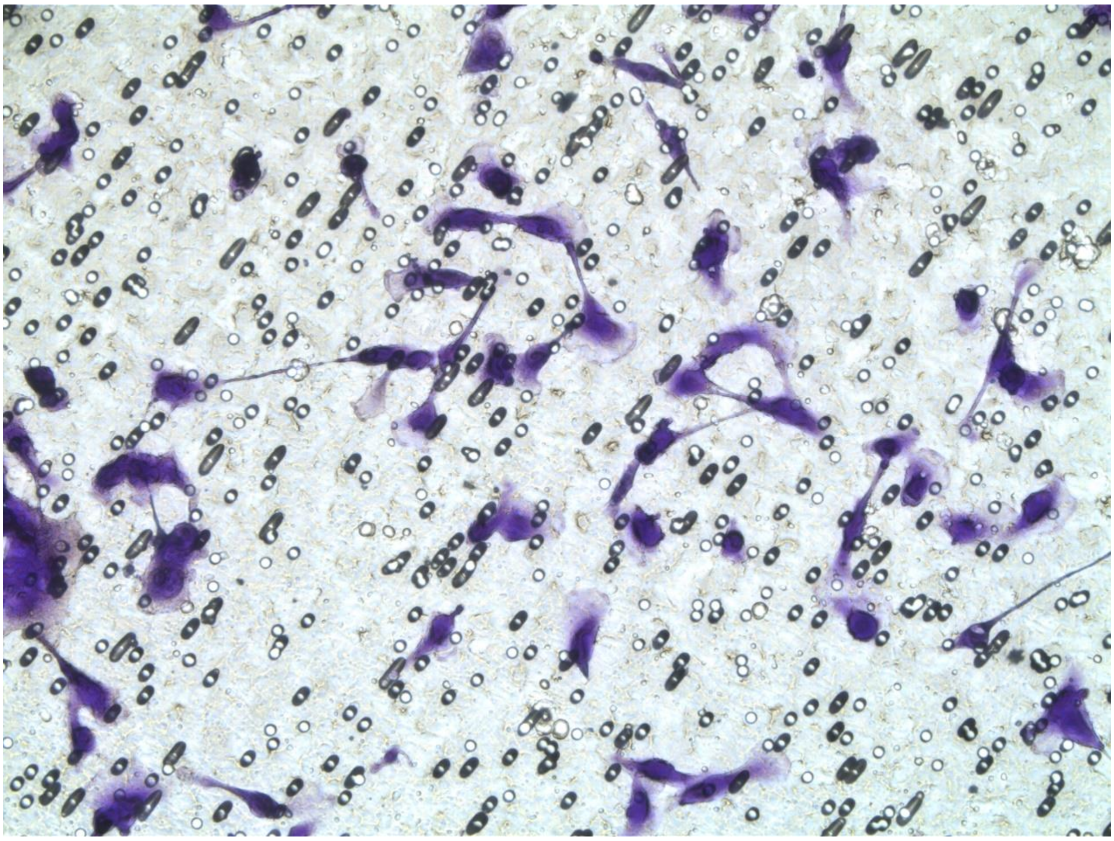

U20S NC 200-4

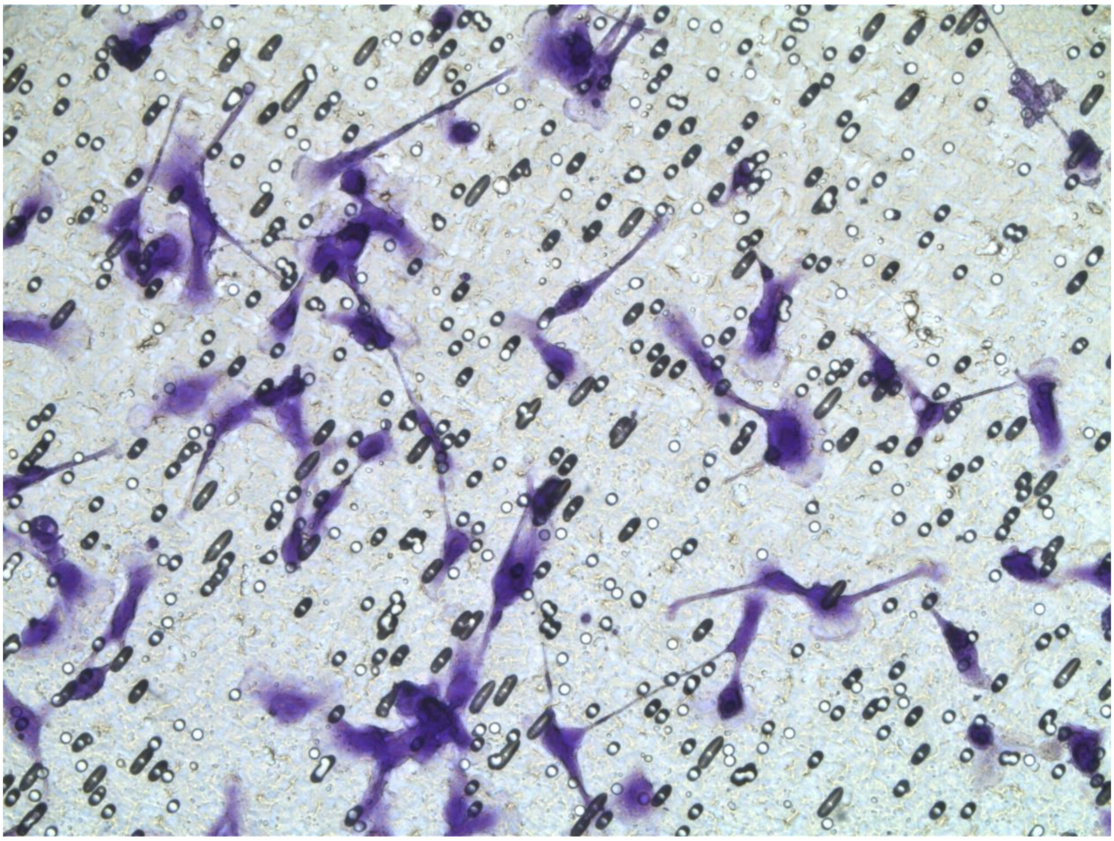

U20S NC 200-5

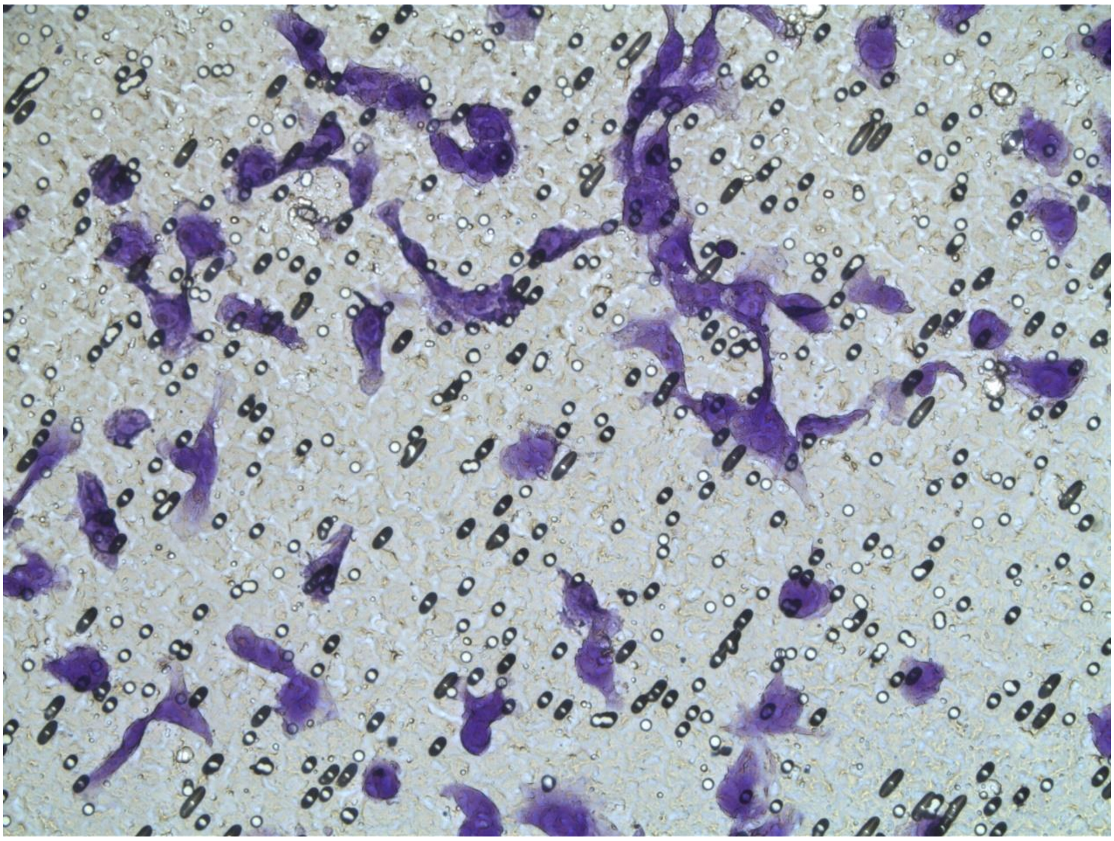

U20S OE 200-1

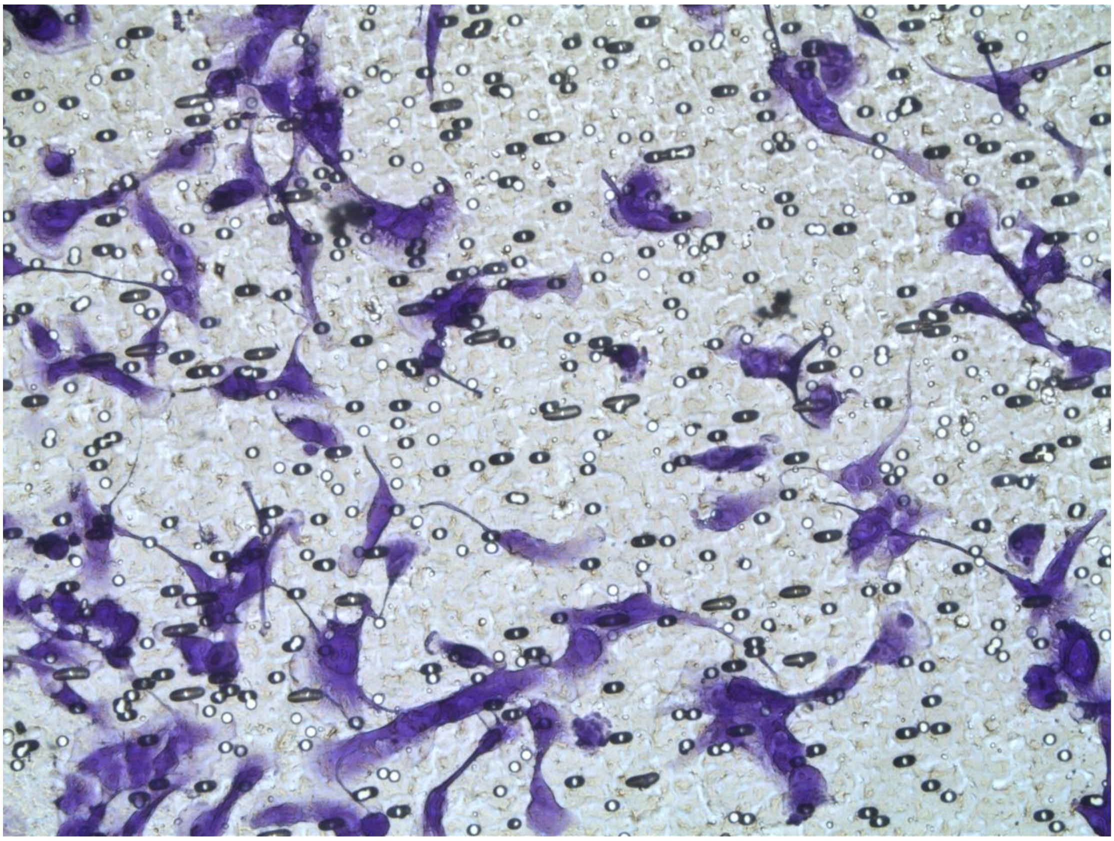

U20S OE 200-2

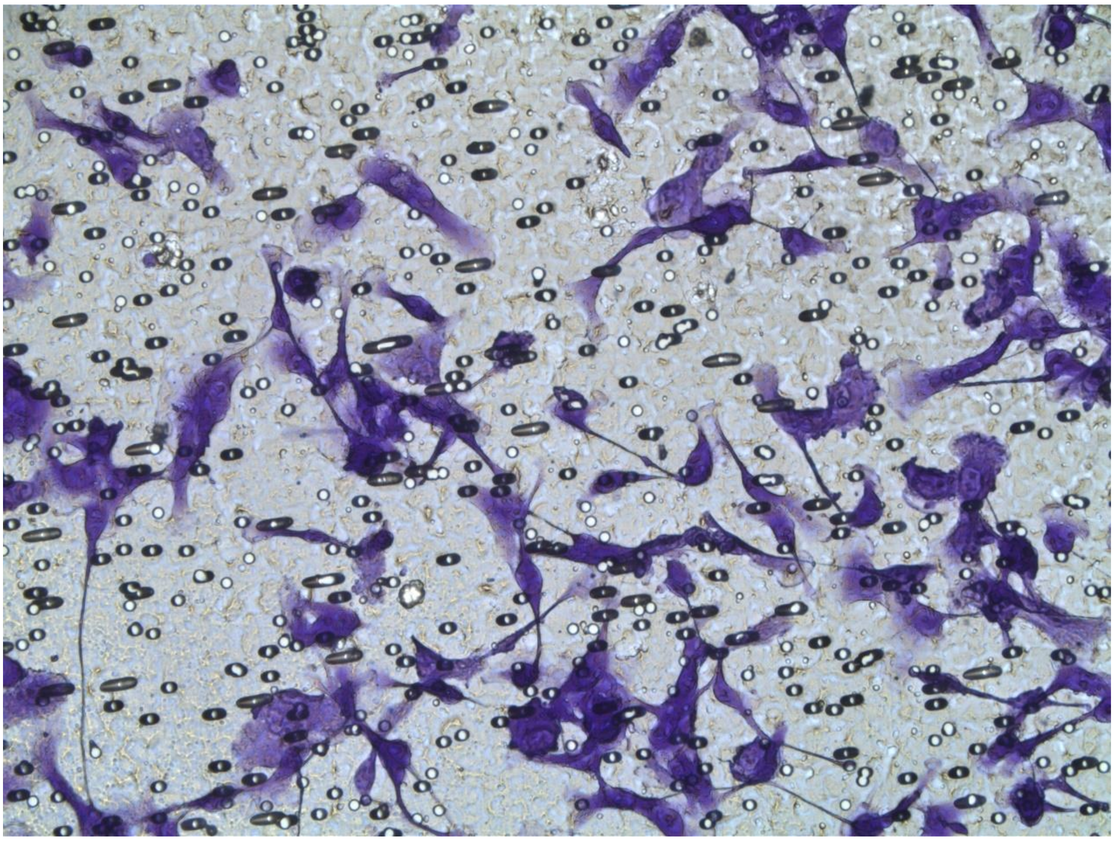

U20S OE 200-3

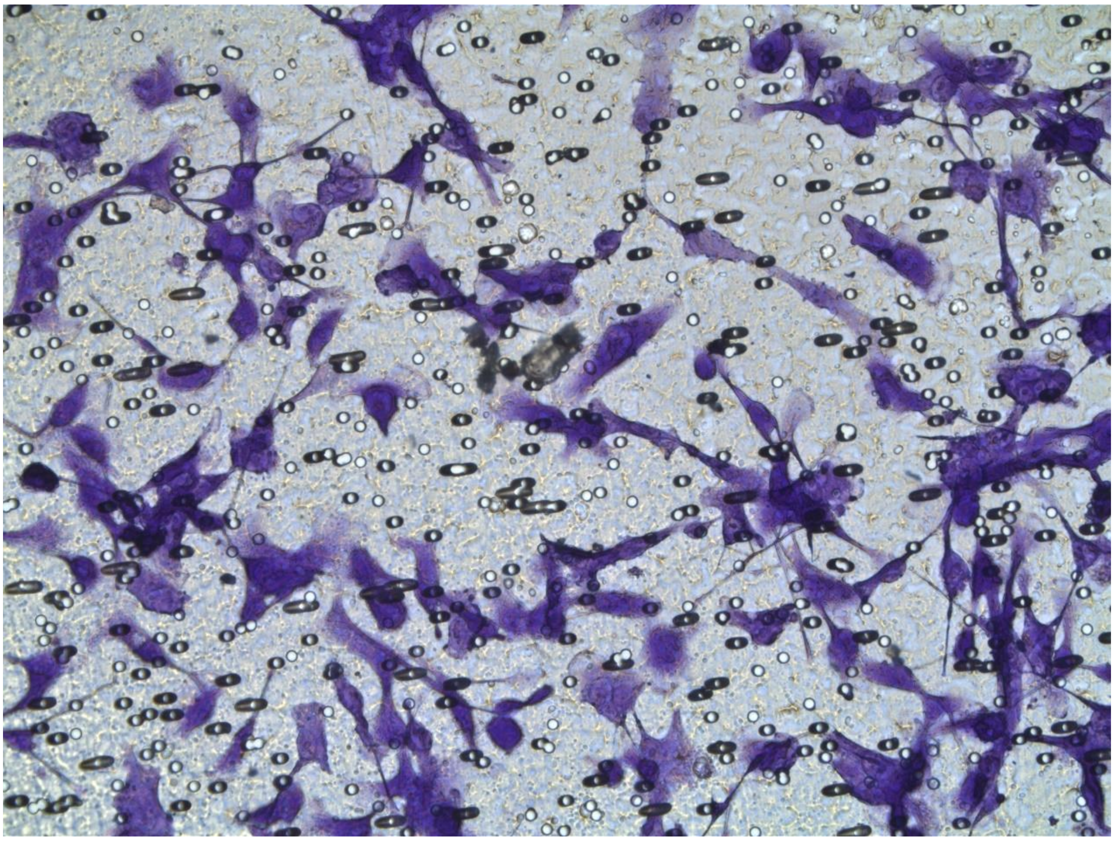

U20S OE 200-4

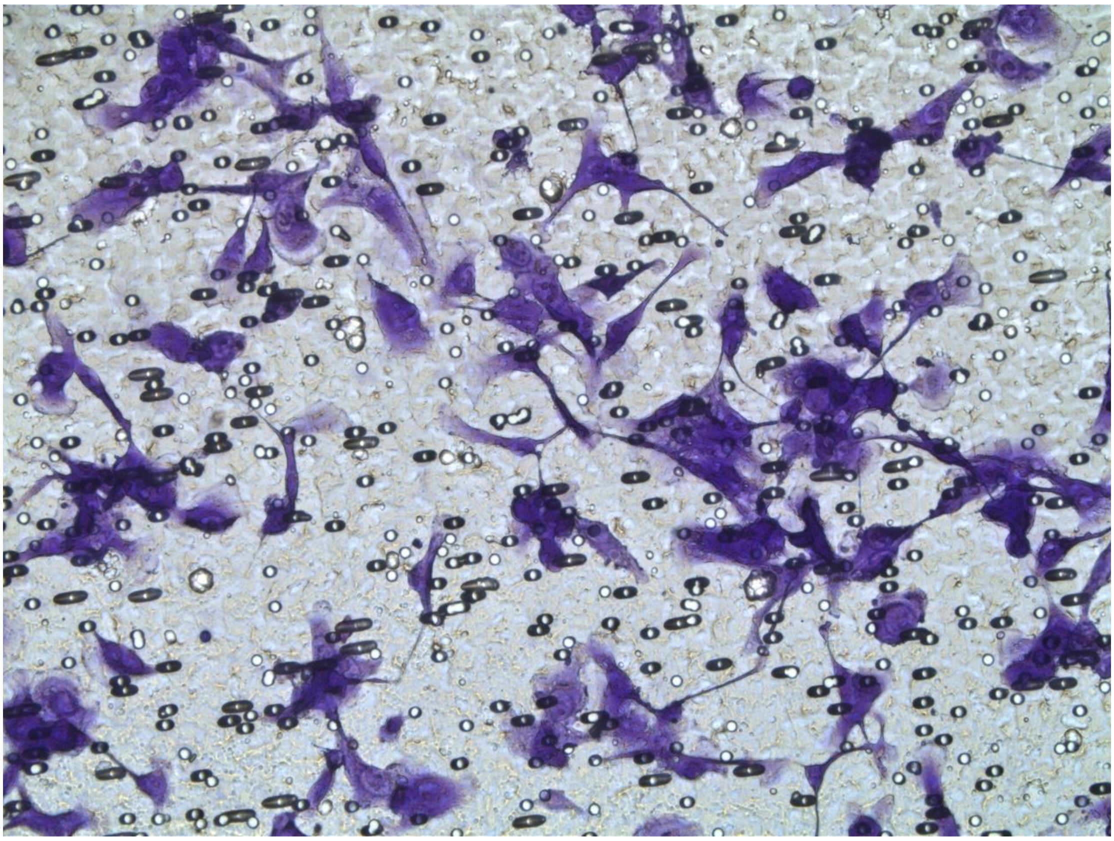

U20S OE 200-5

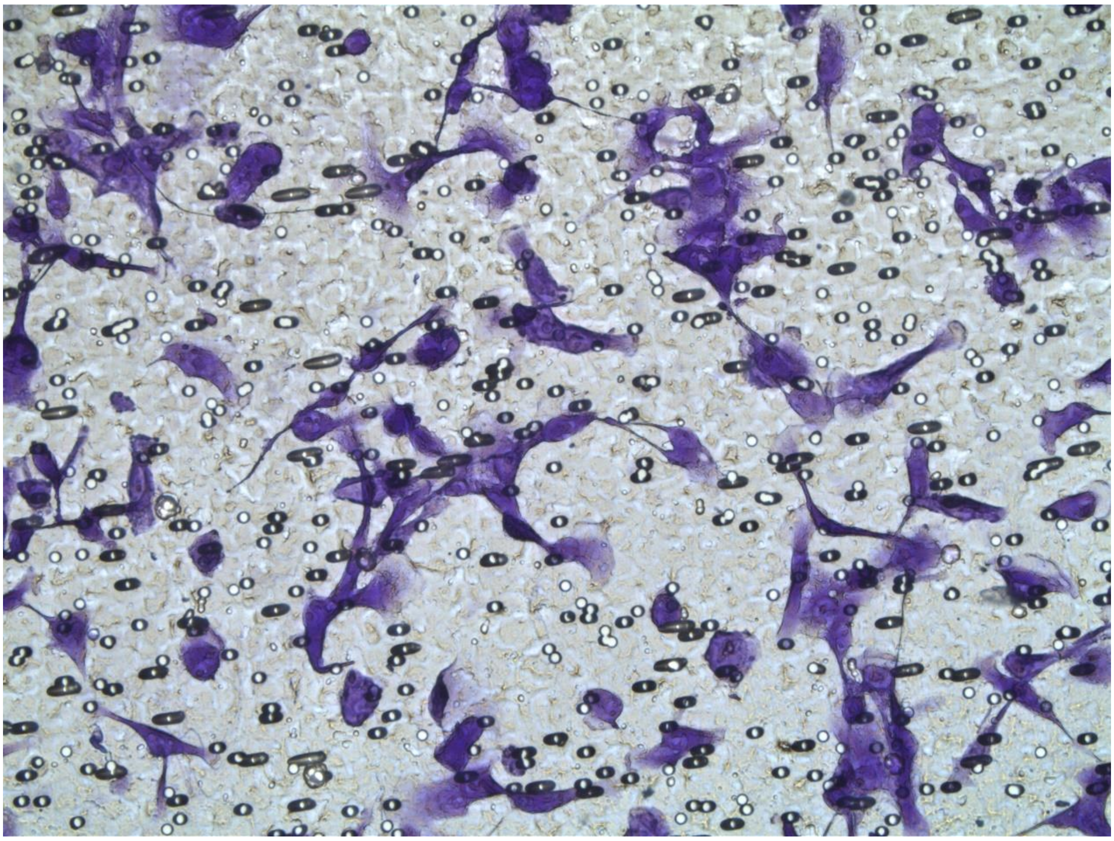

Fig 2 WB

GAPDH

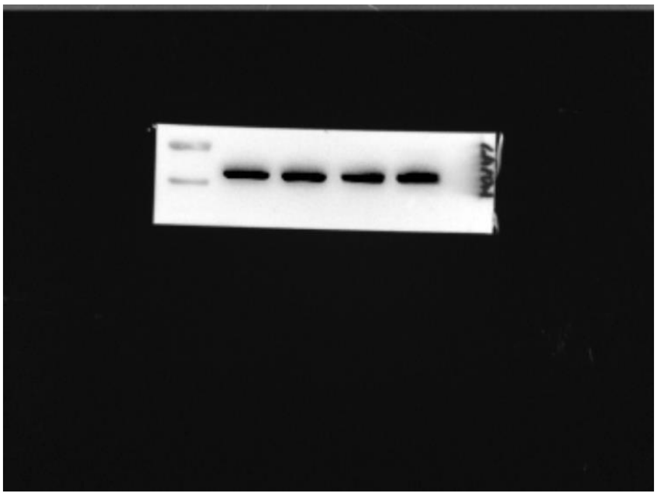

S100A16

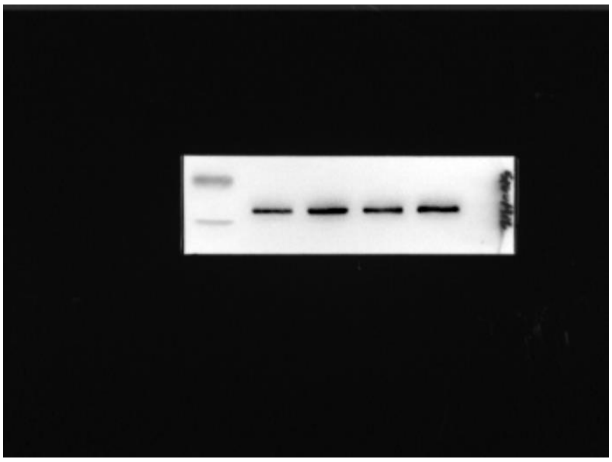

Fig 3 scratch

MG-63 si S100A8   0h 100-1

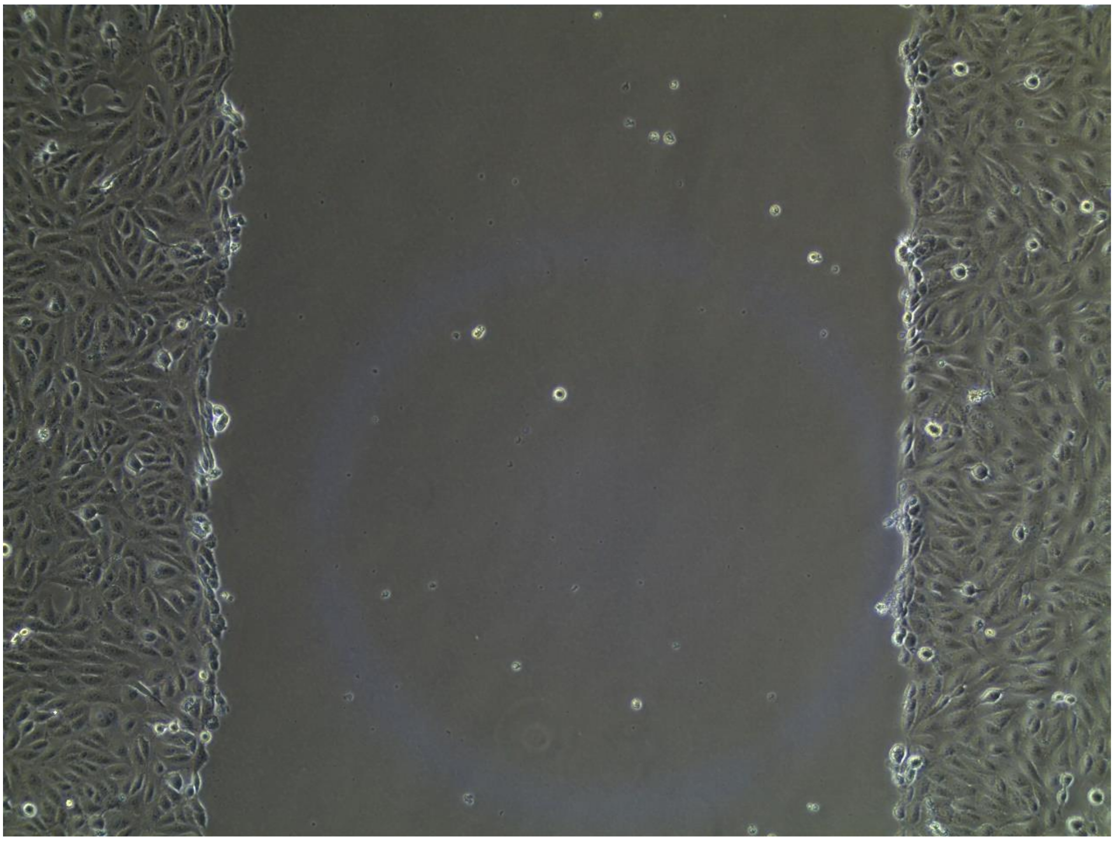

MG-63 si S100A8   0h 100-2

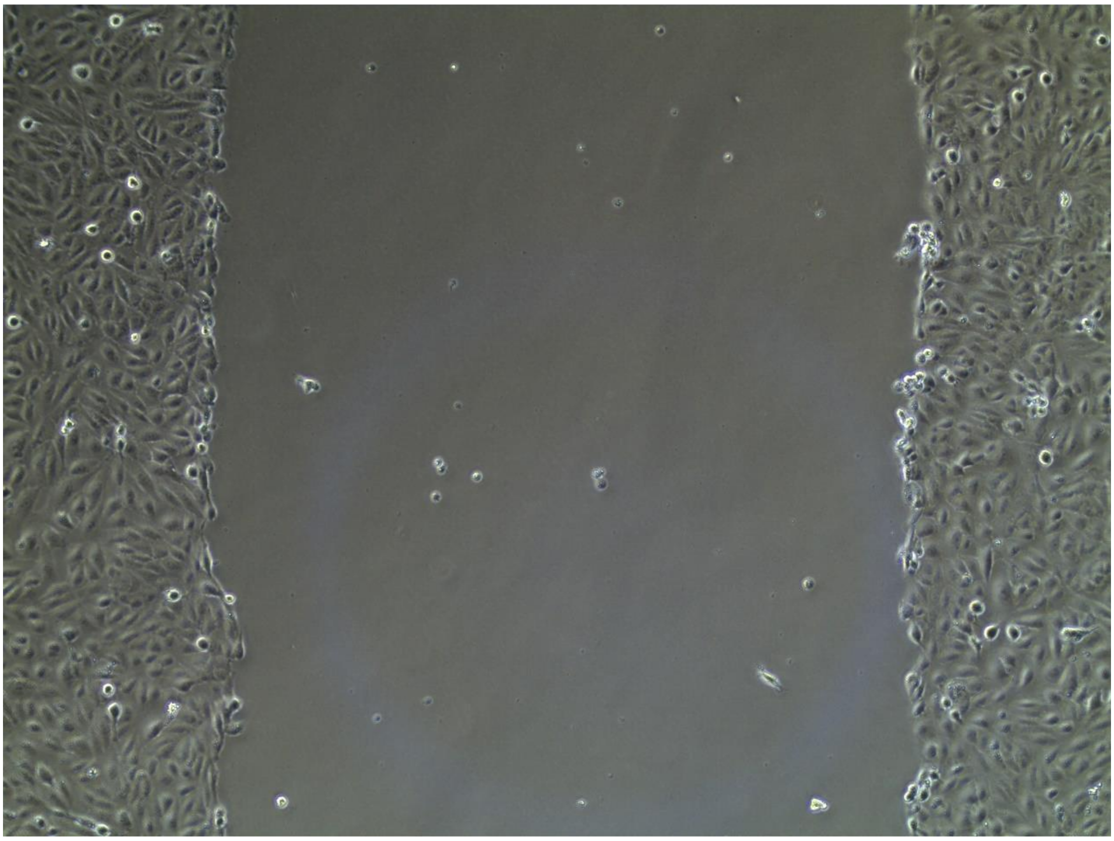

MG-63 si S100A8   0h 100-3

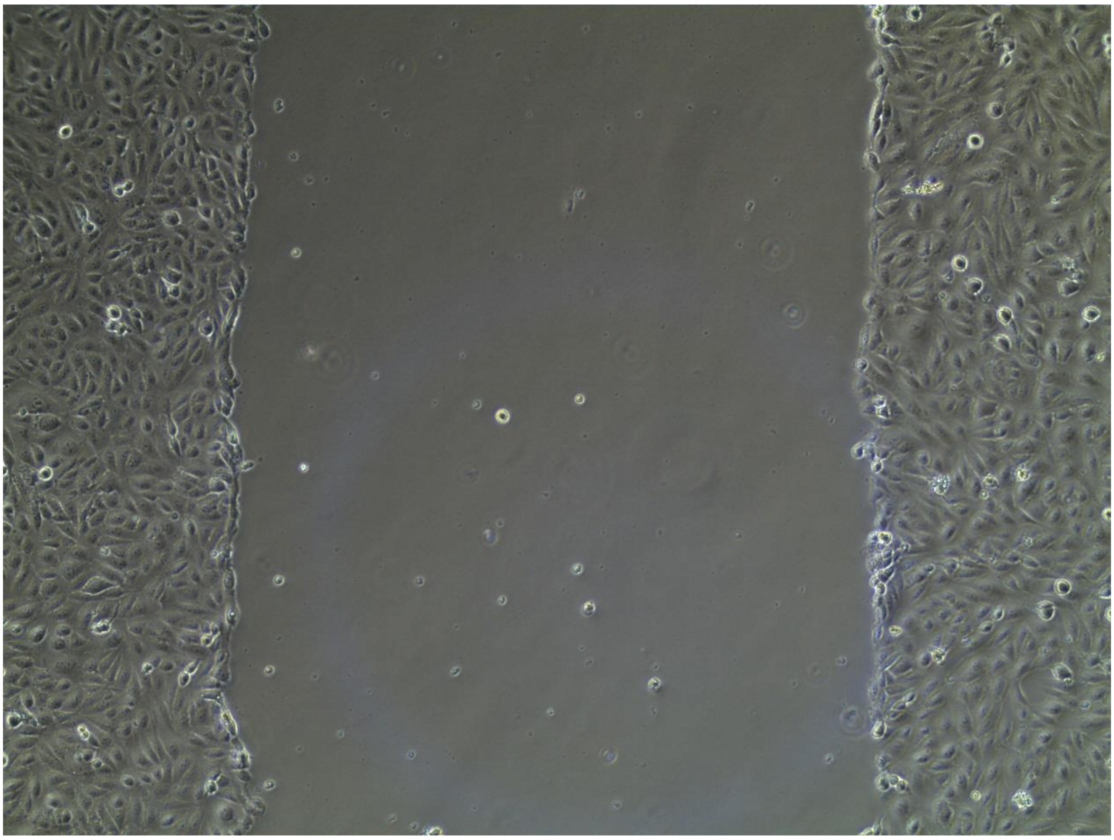

MG-63 si S100A8 24h 100-1

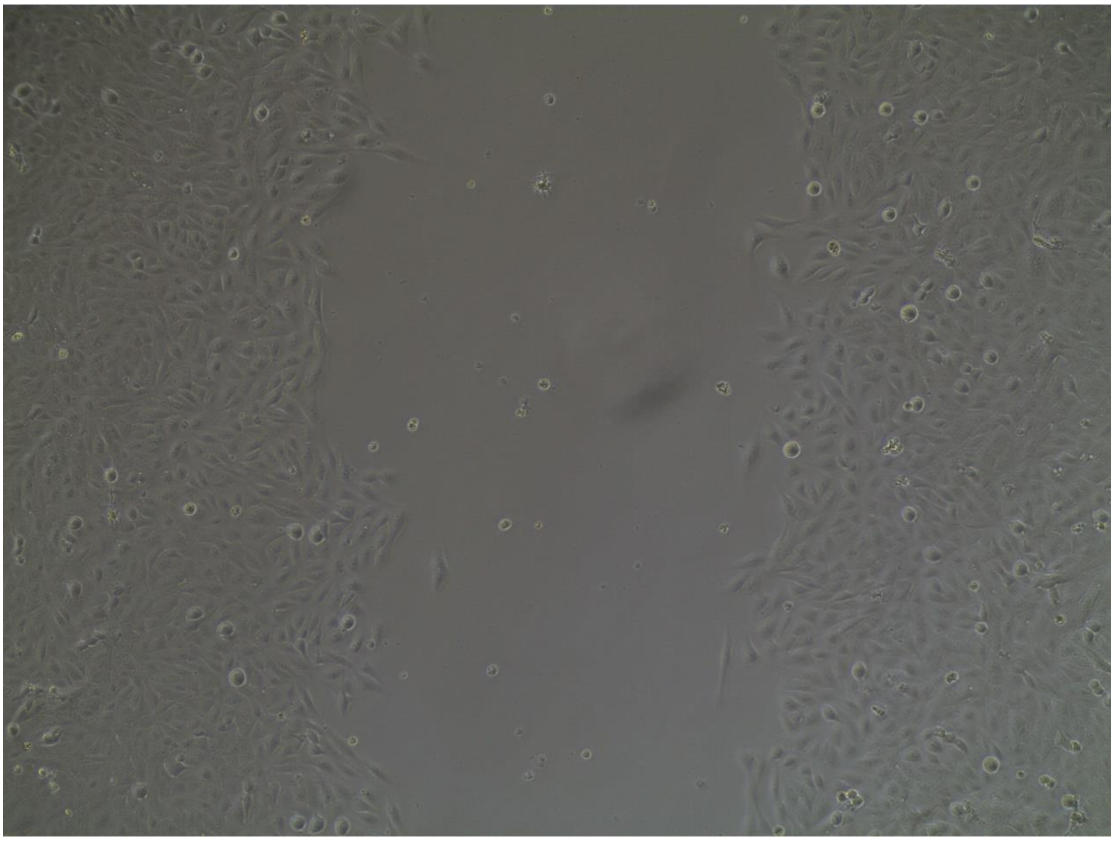

MG-63 si S100A8 24h 100-2

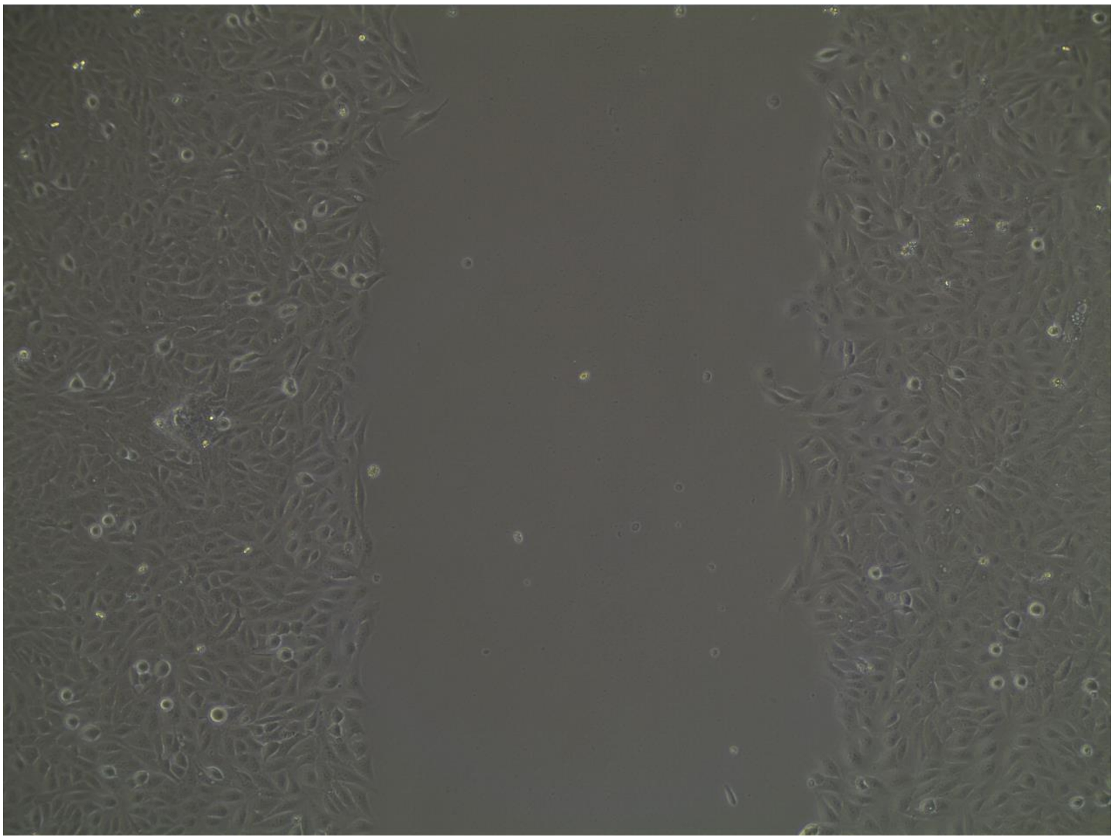

MG-63 si S100A8 24h 100-3

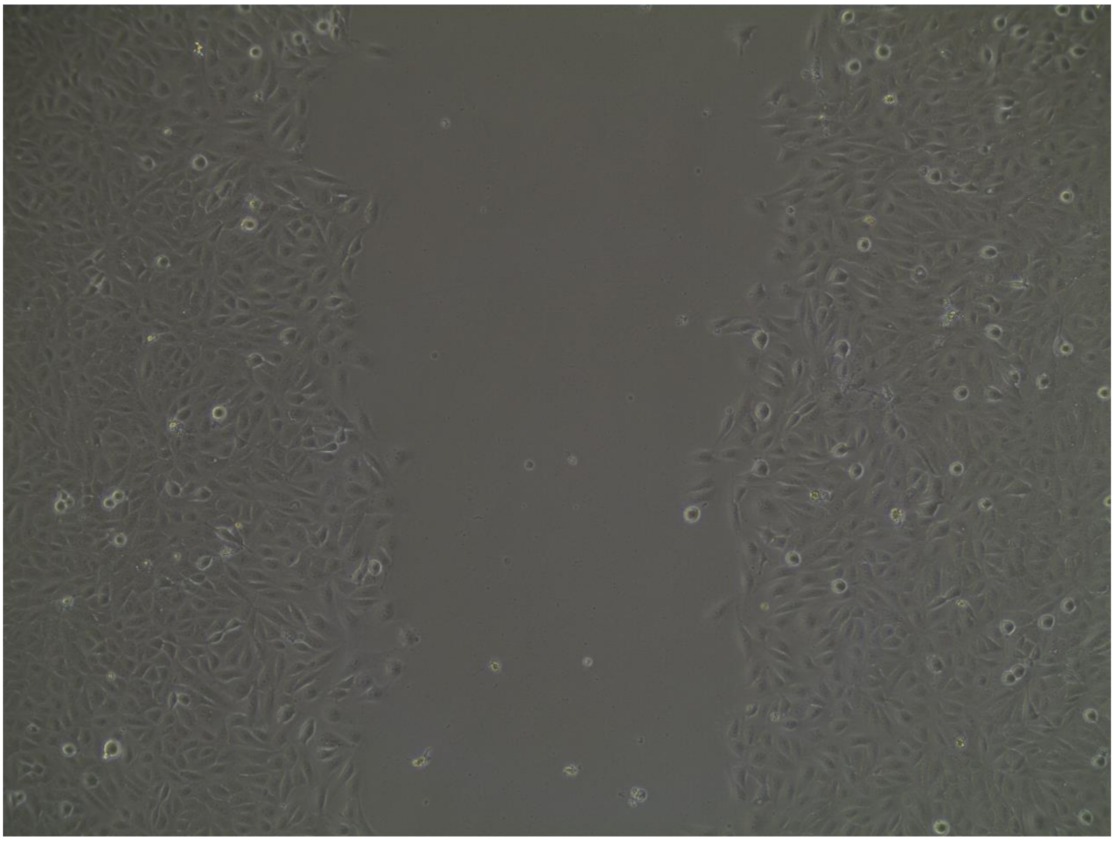

MG-63 siNC 0h 100-1

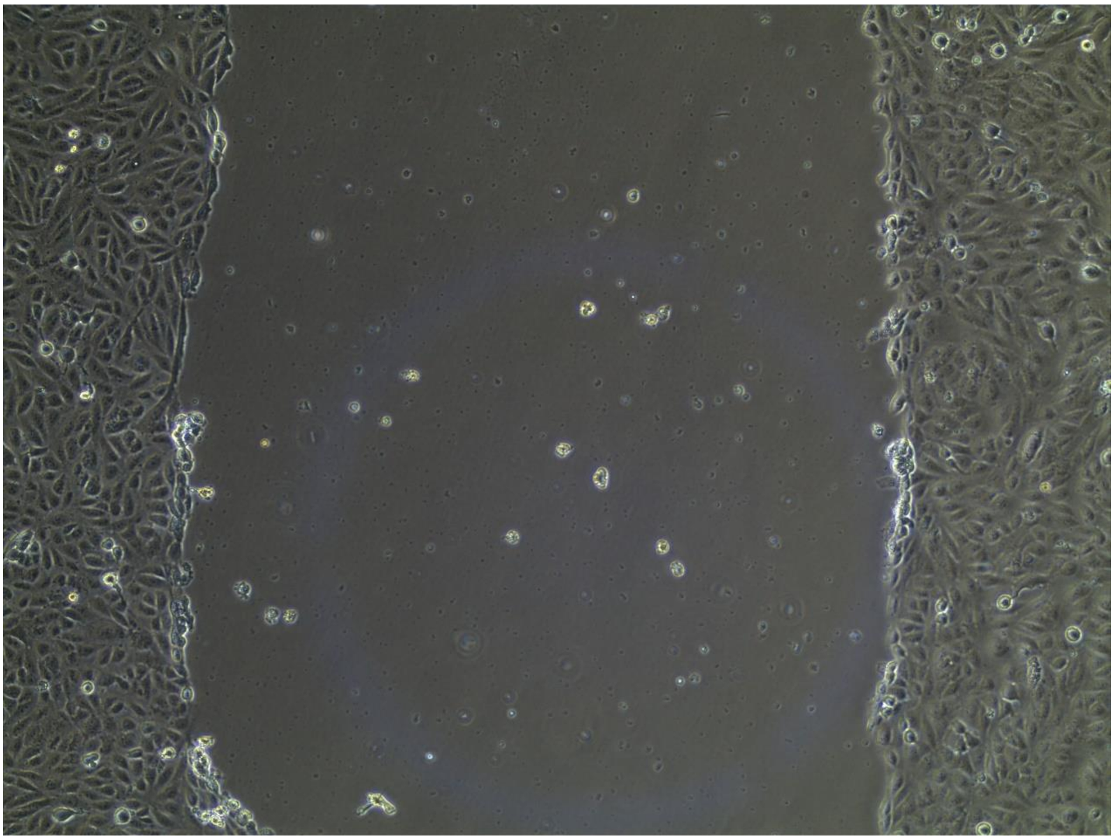

MG-63 siNC 0h 100-2

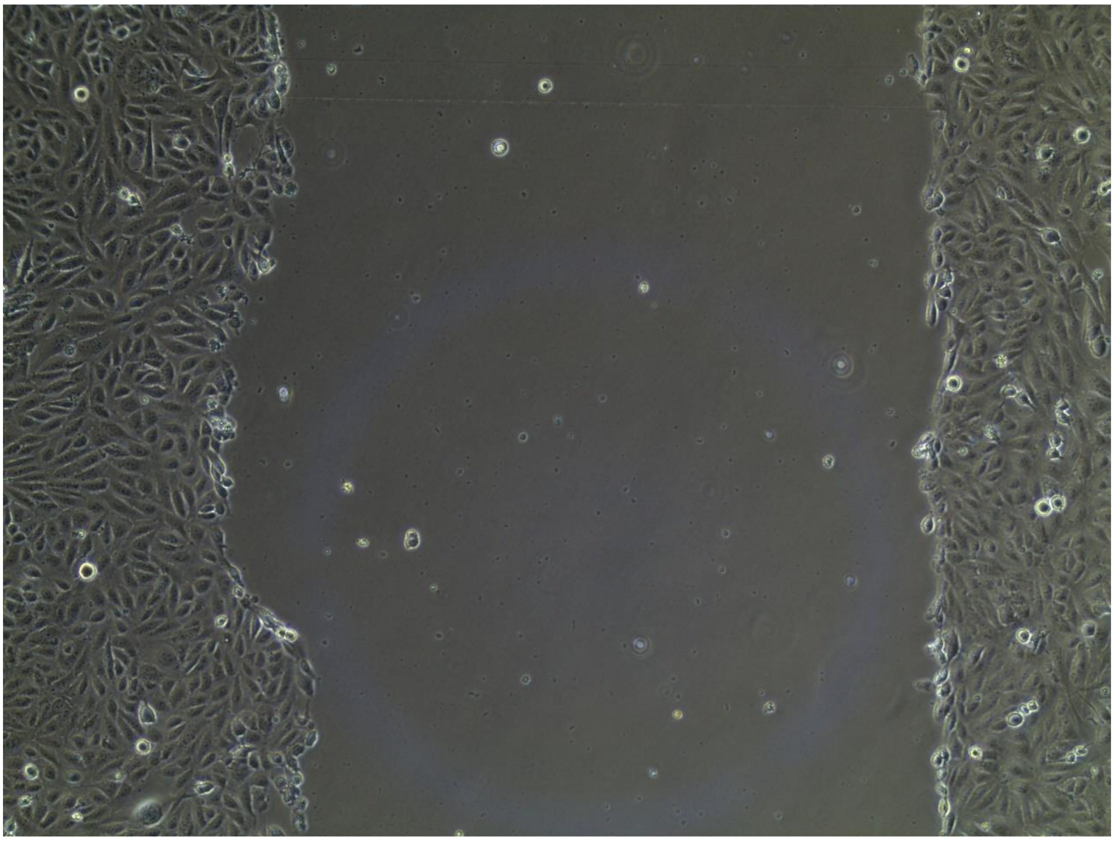

MG-63 siNC 0h 100-3

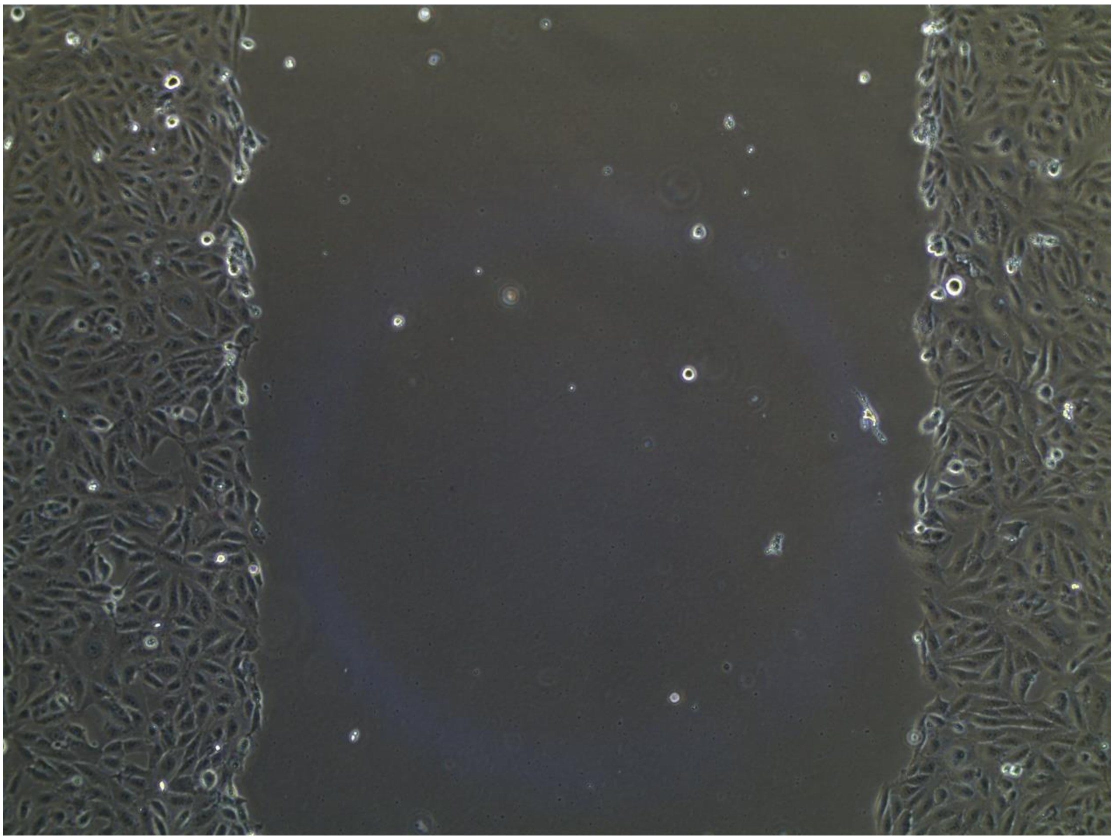

MG-63 siNC 24h 100-1

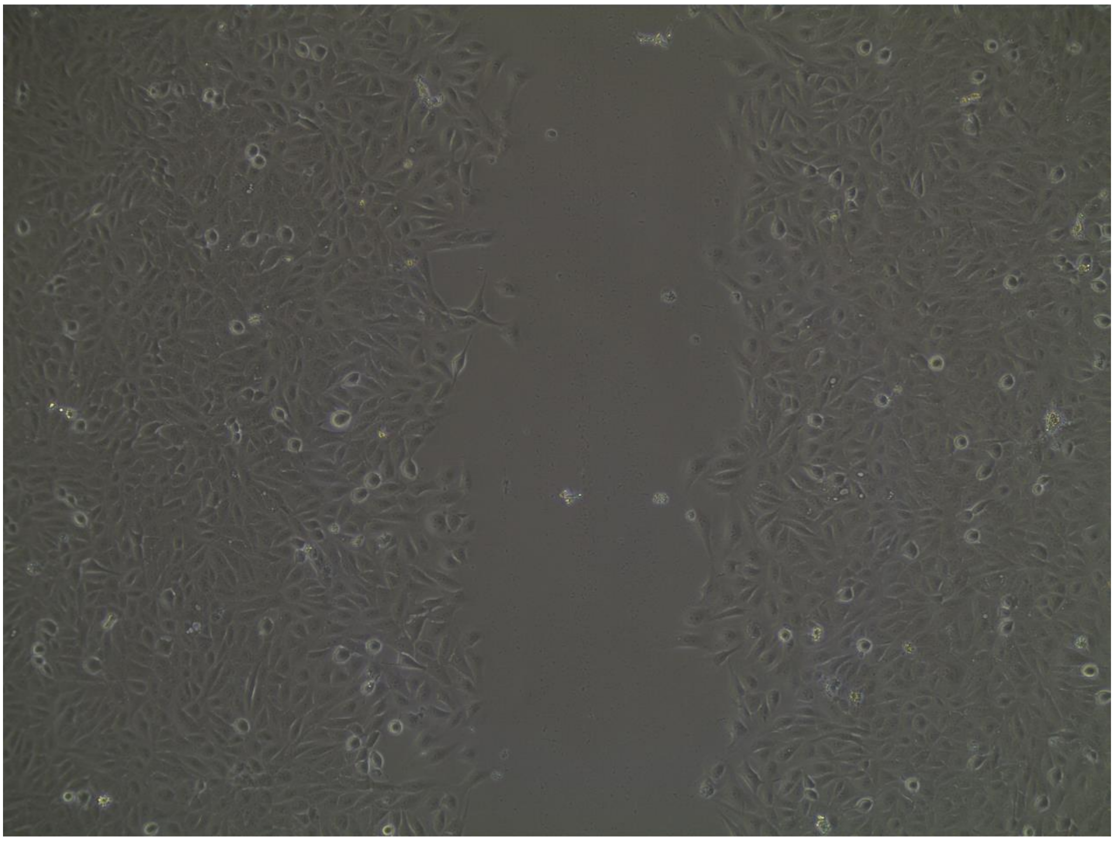

MG-63 siNC 24h 100-2

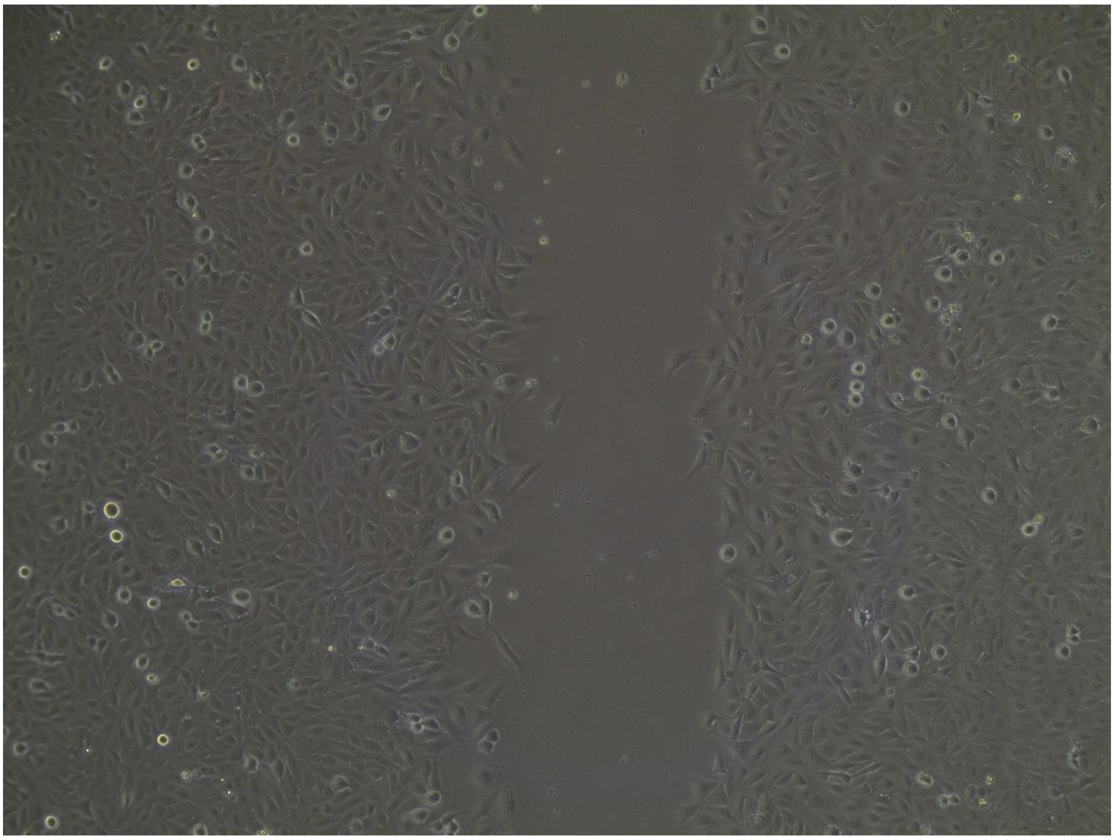

MG-63 siNC 24h 100-3

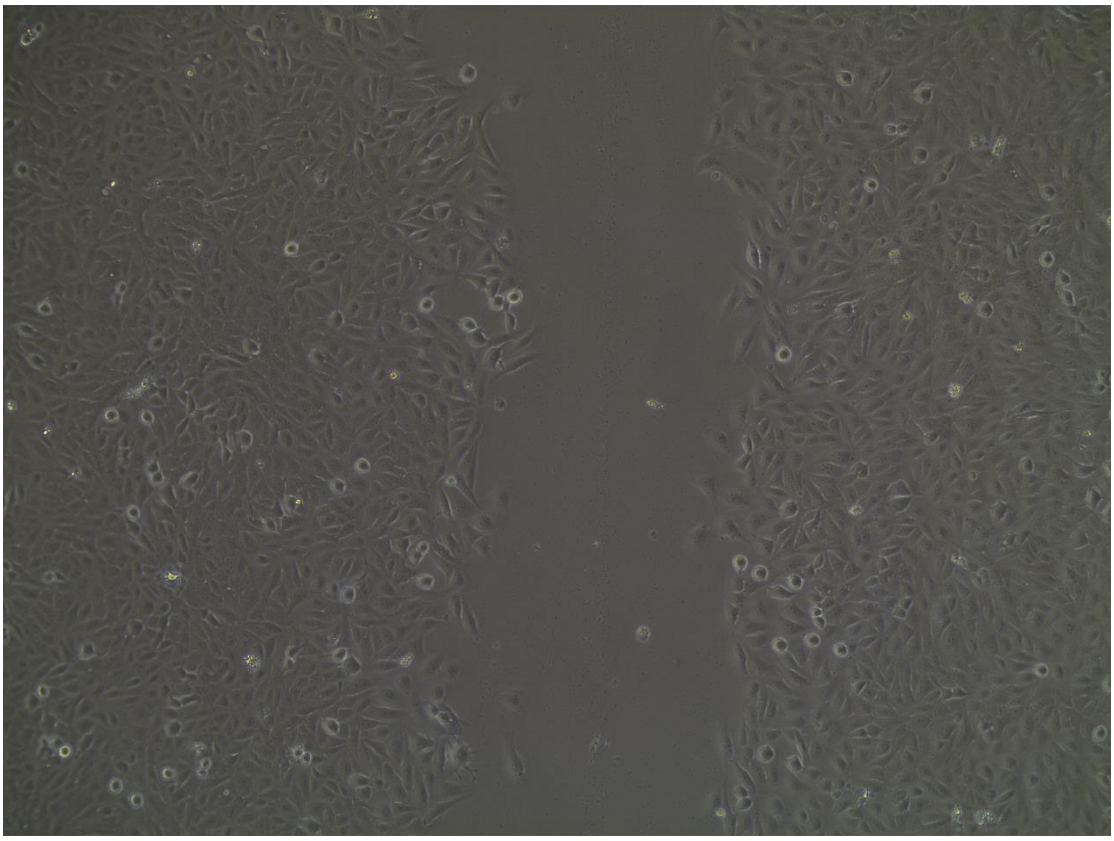

U20S si-NC 0h 100-1

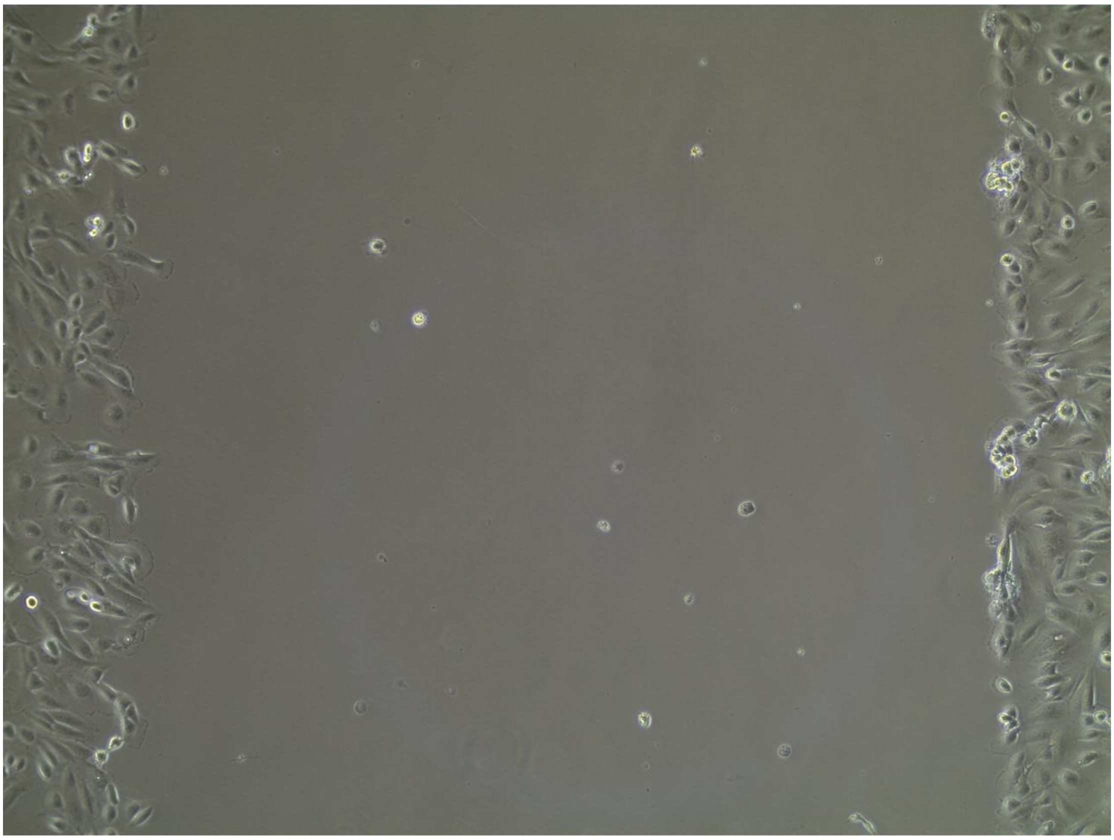

U20S si-NC 0h 100-2

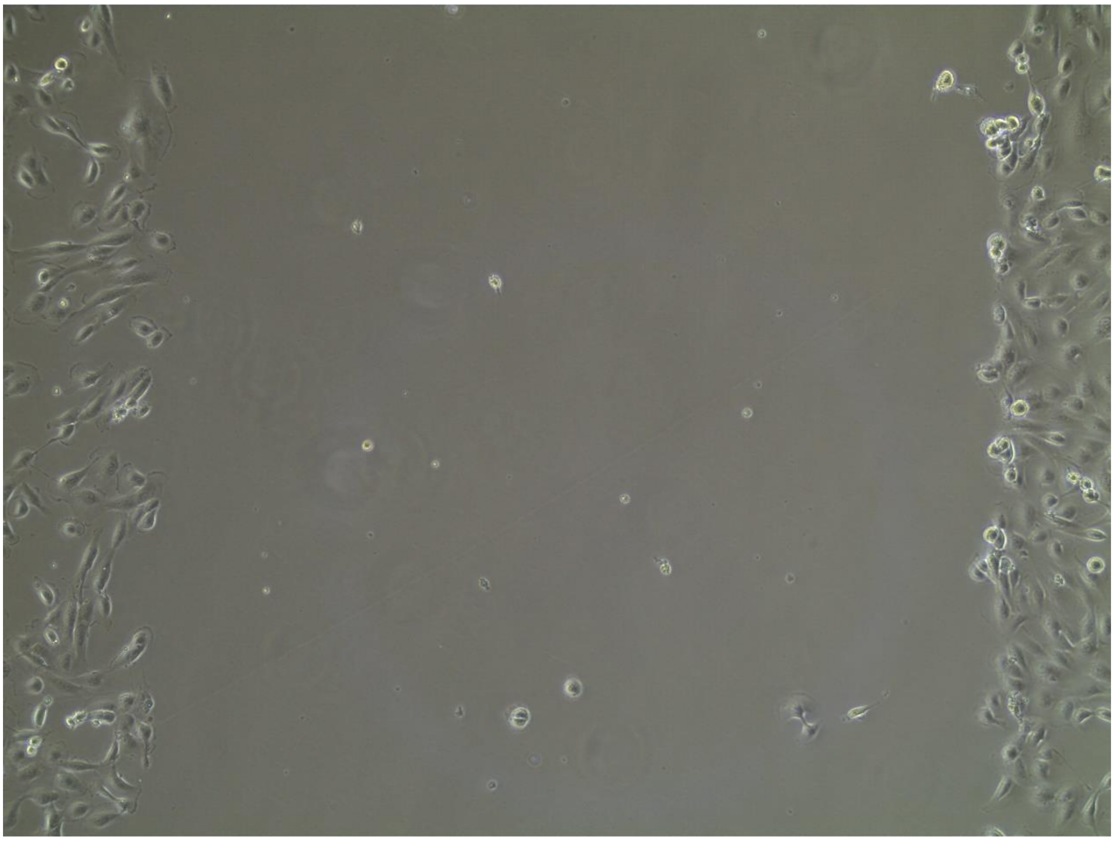

U20S si-NC 0h 100-3

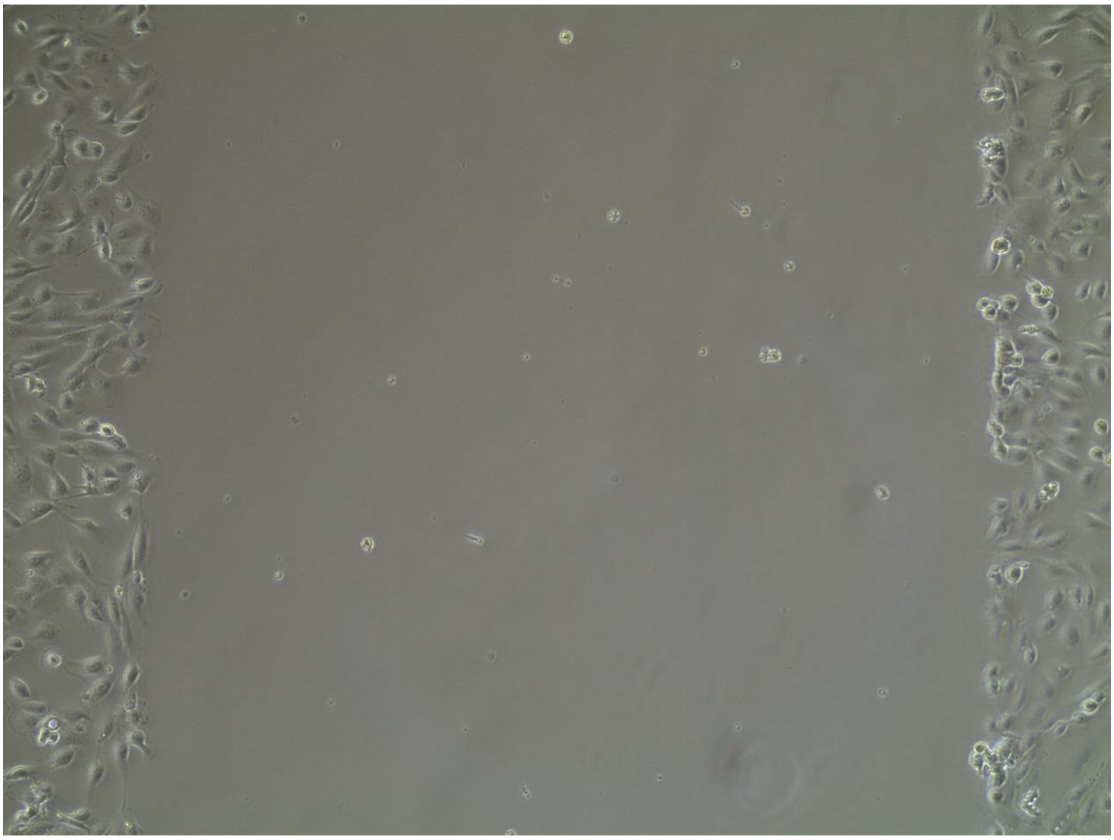

U20S si-NC 24h 100-1

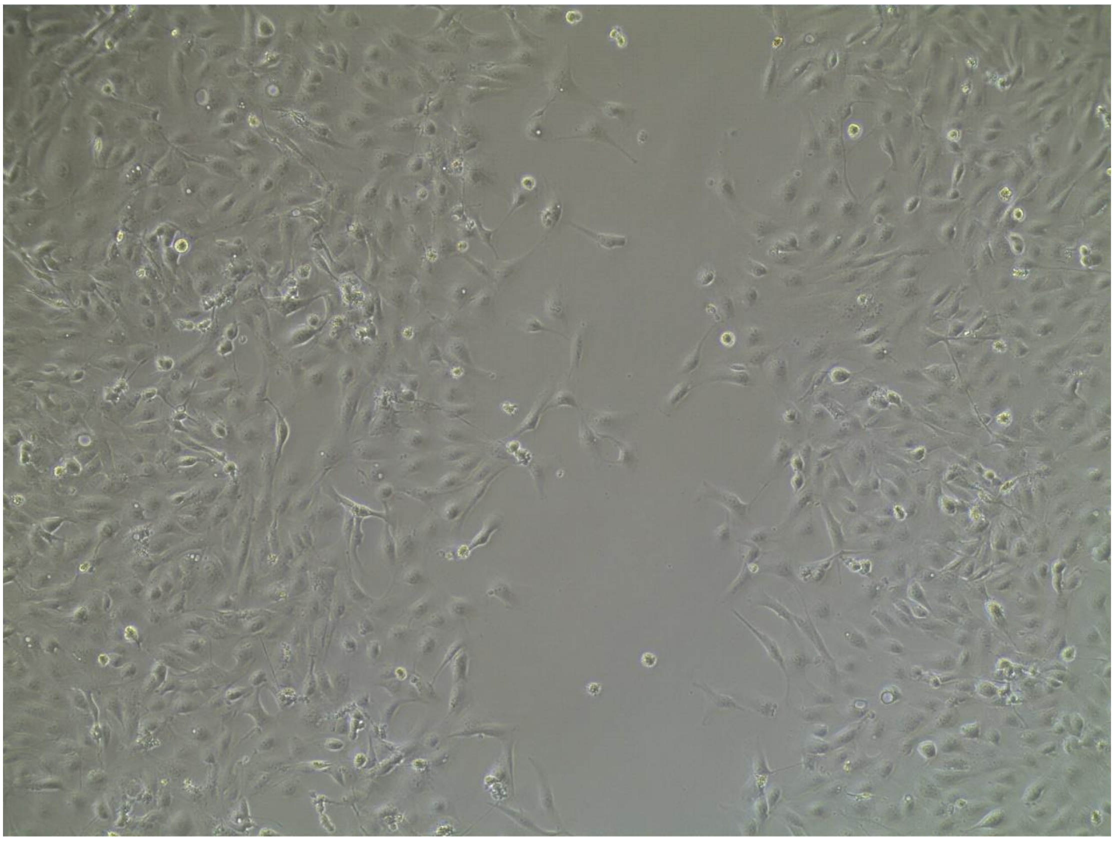

U20S si-NC 24h 100-2

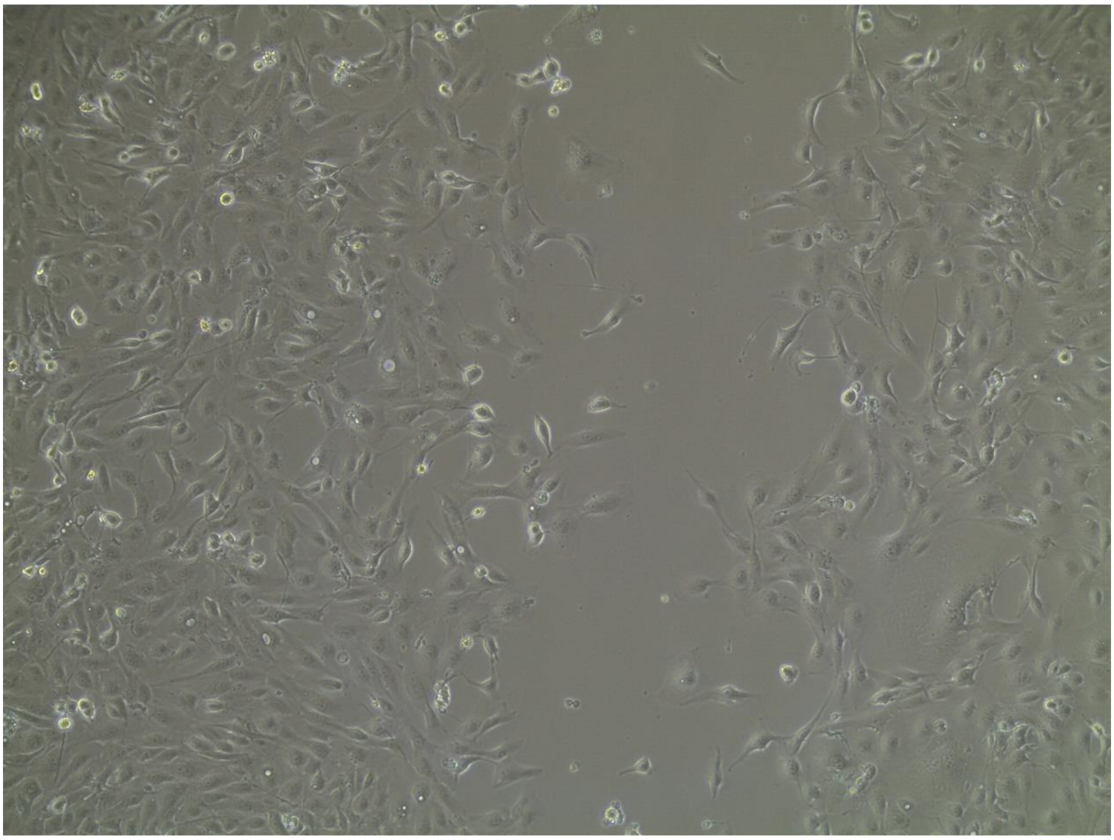

U20S si-NC 24h 100-3

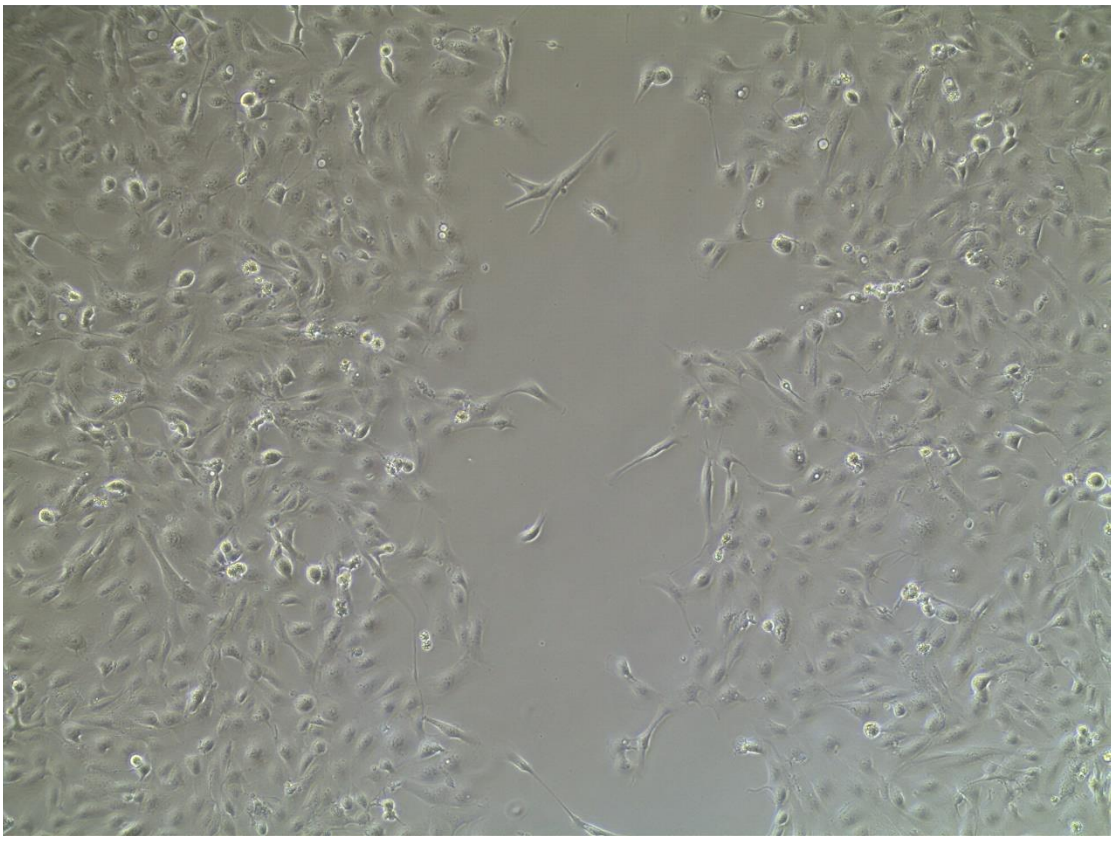

U20S si-S100A16 0h 100-1

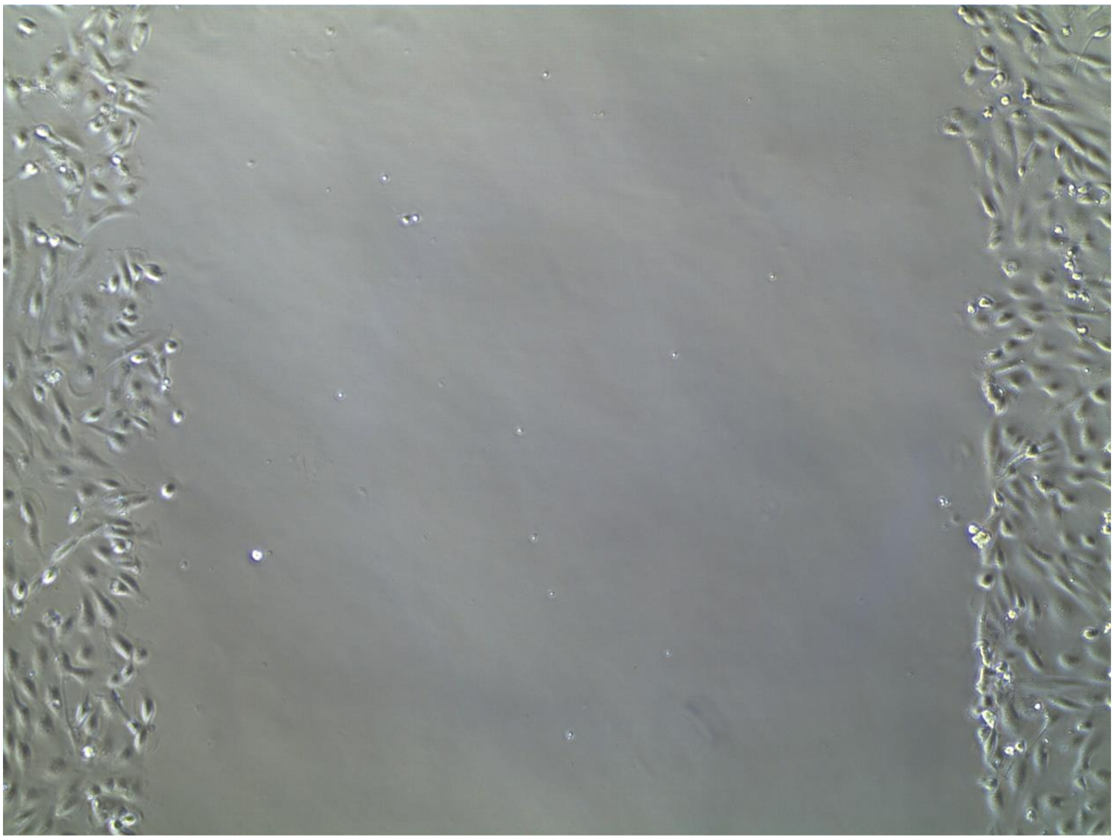

U20S si-S100A16 0h 100-2

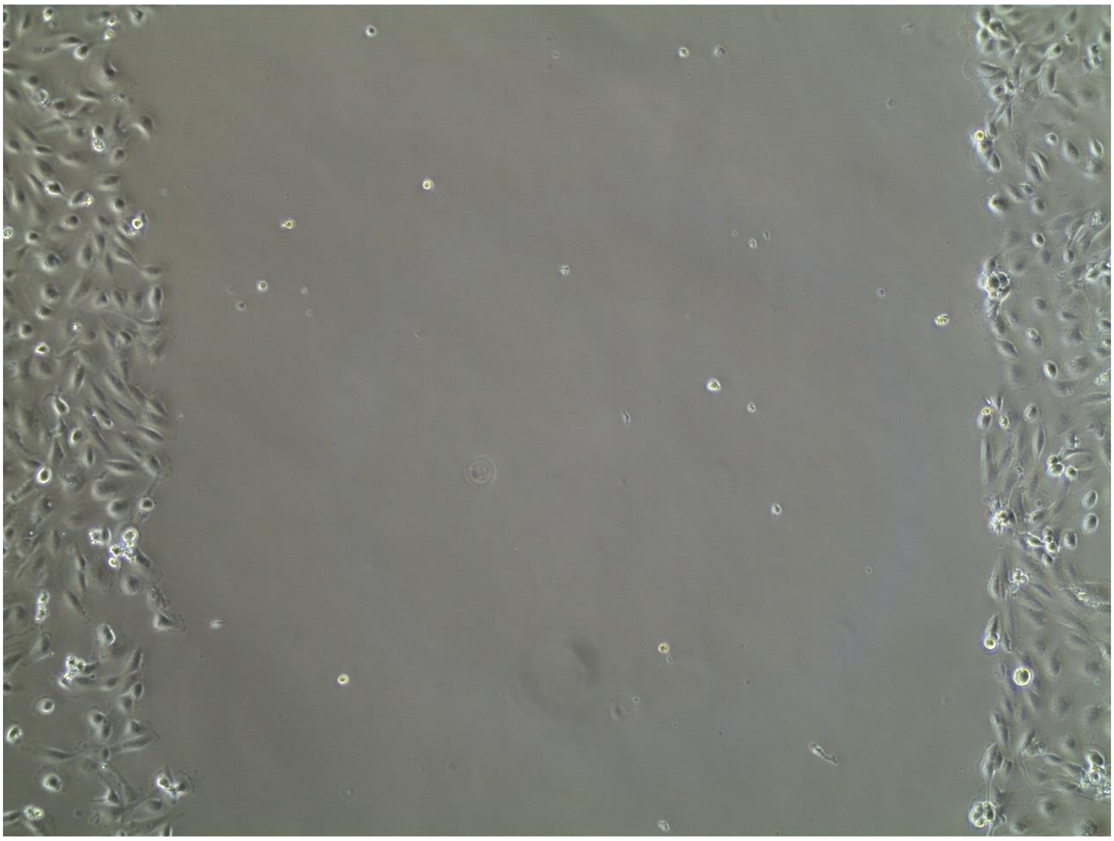

U20S si-S100A16 0h 100-3

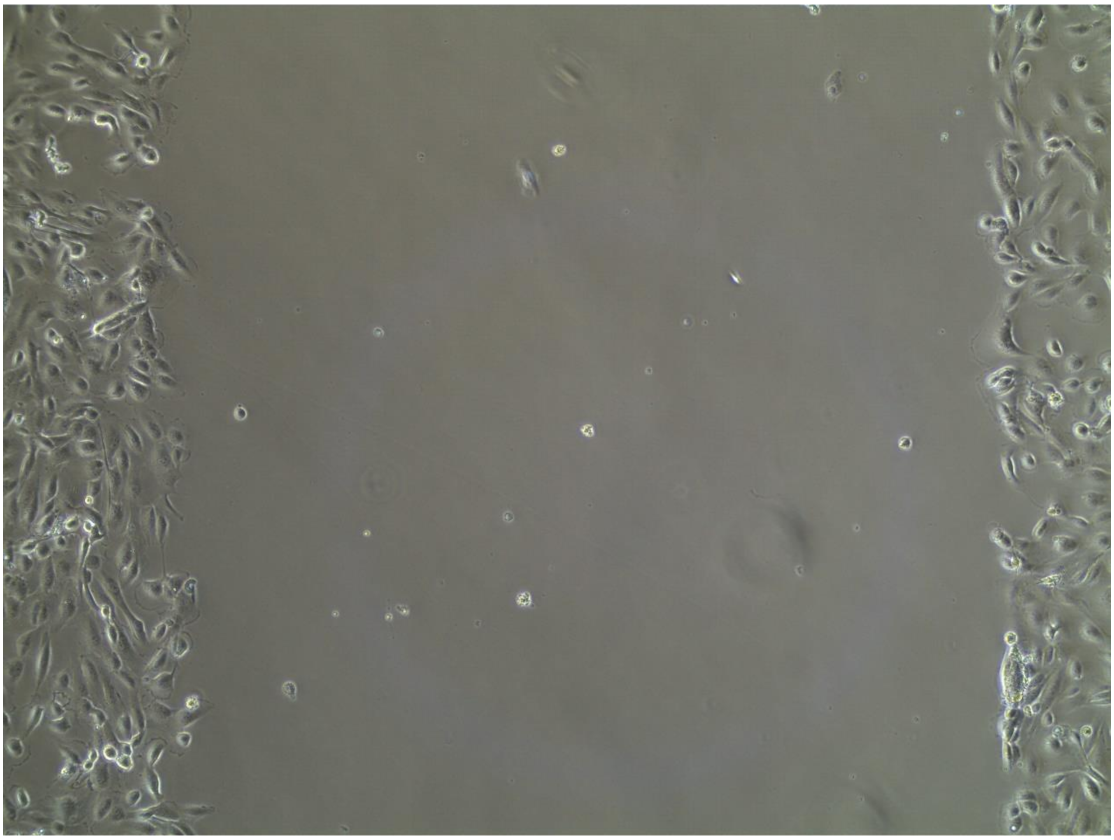

U20S si-S100A16 24h 100-1

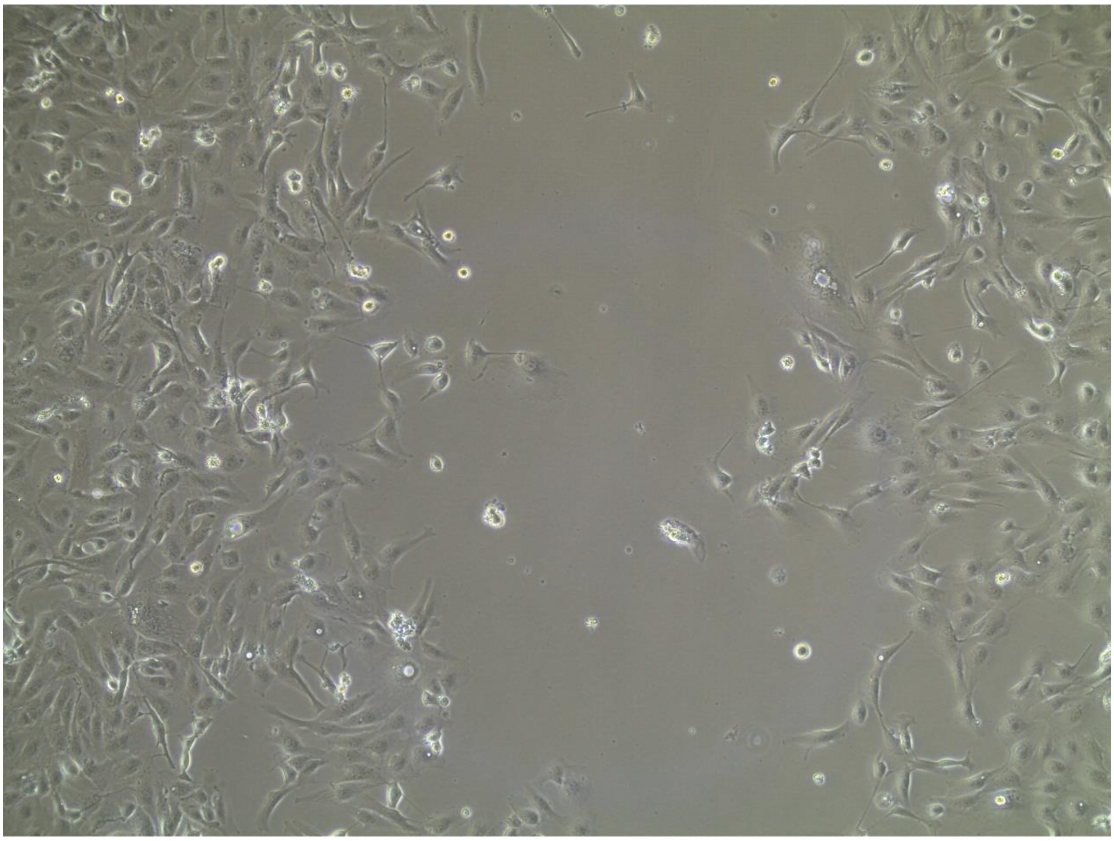

U20S si-S100A16 24h 100-2

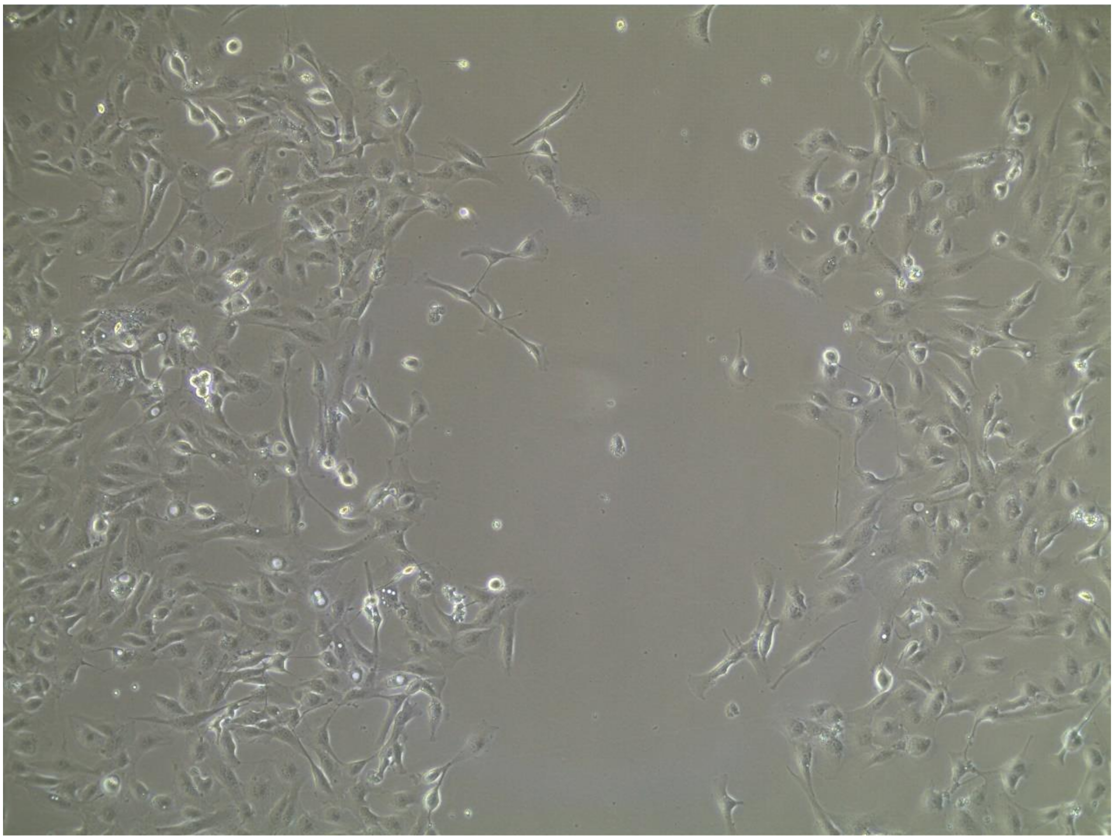

U20S si-S100A16 24h 100-3

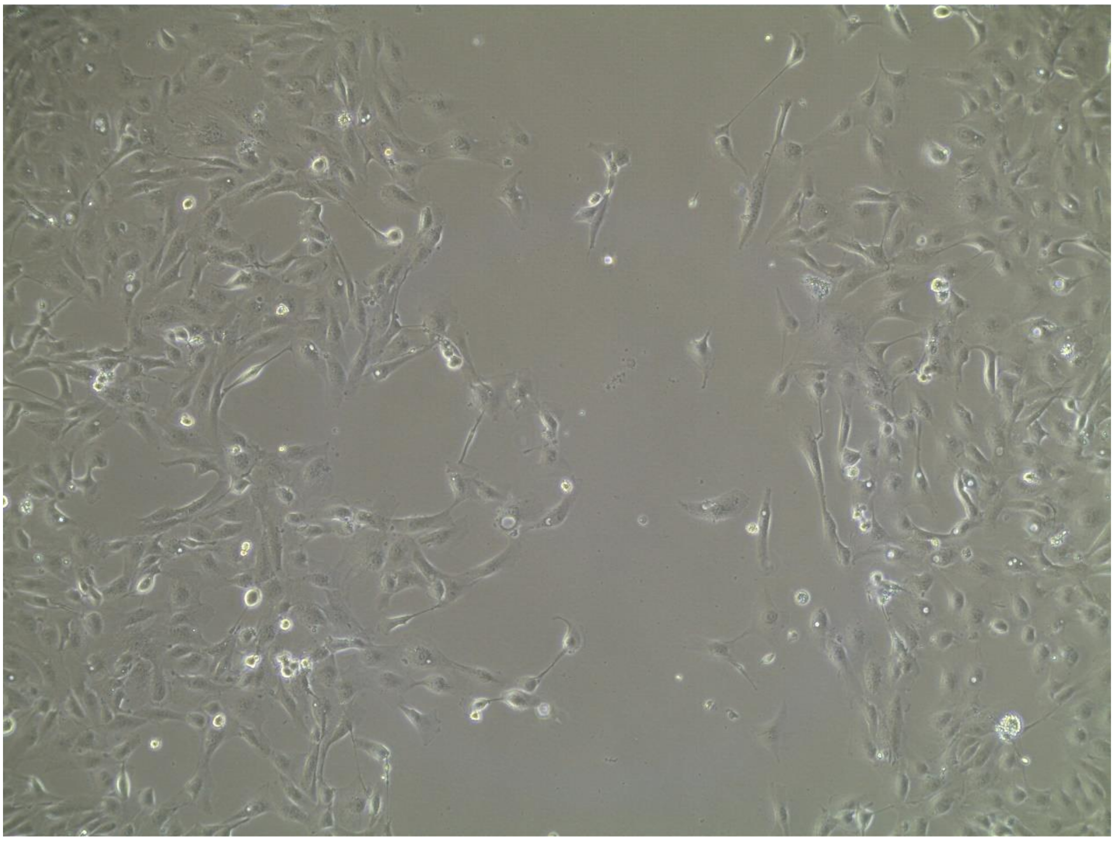

Fig 3 transwell
MG63 si-NC 200-1

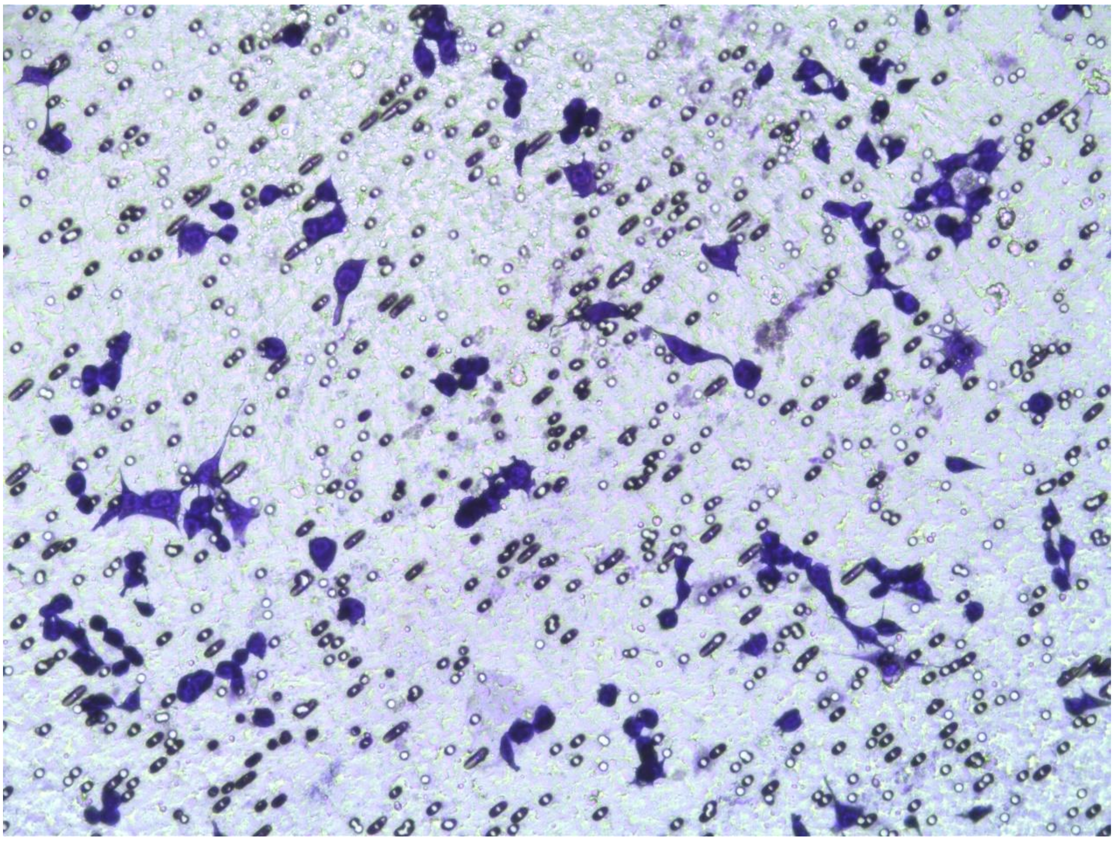

MG63 si-NC 200-2

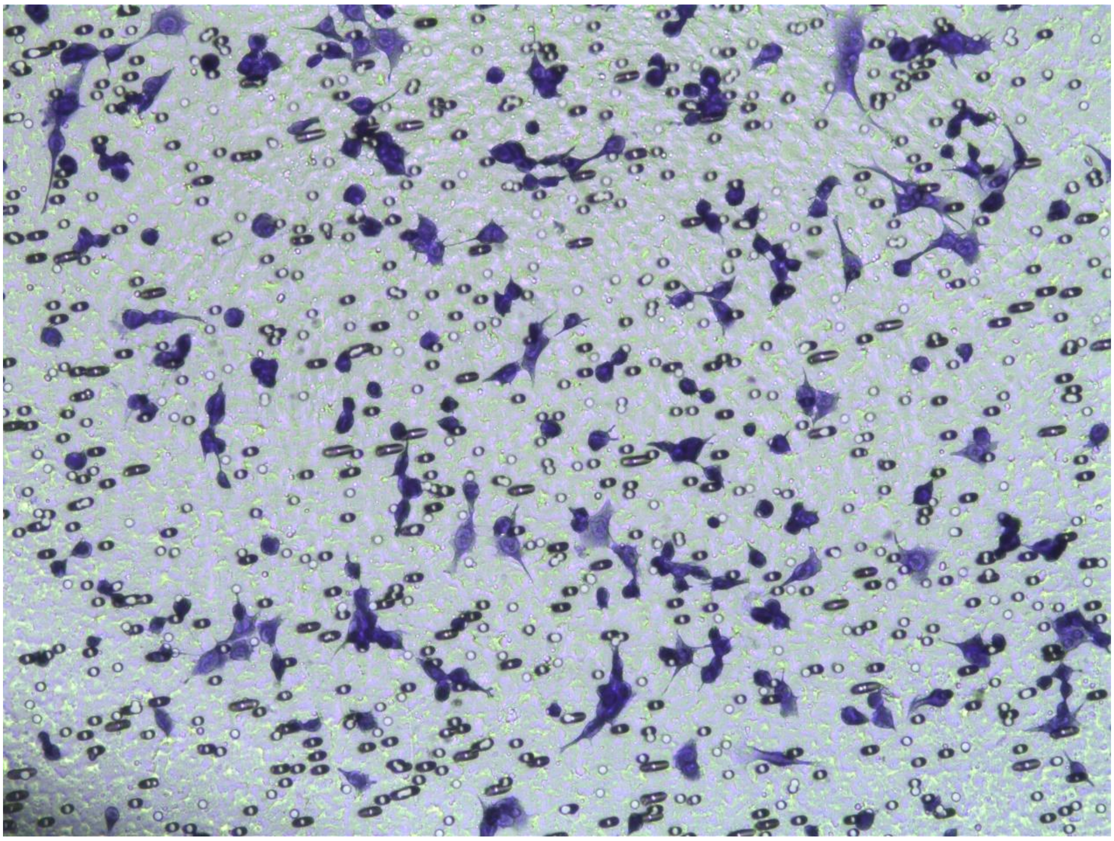

MG63 si-NC 200-3

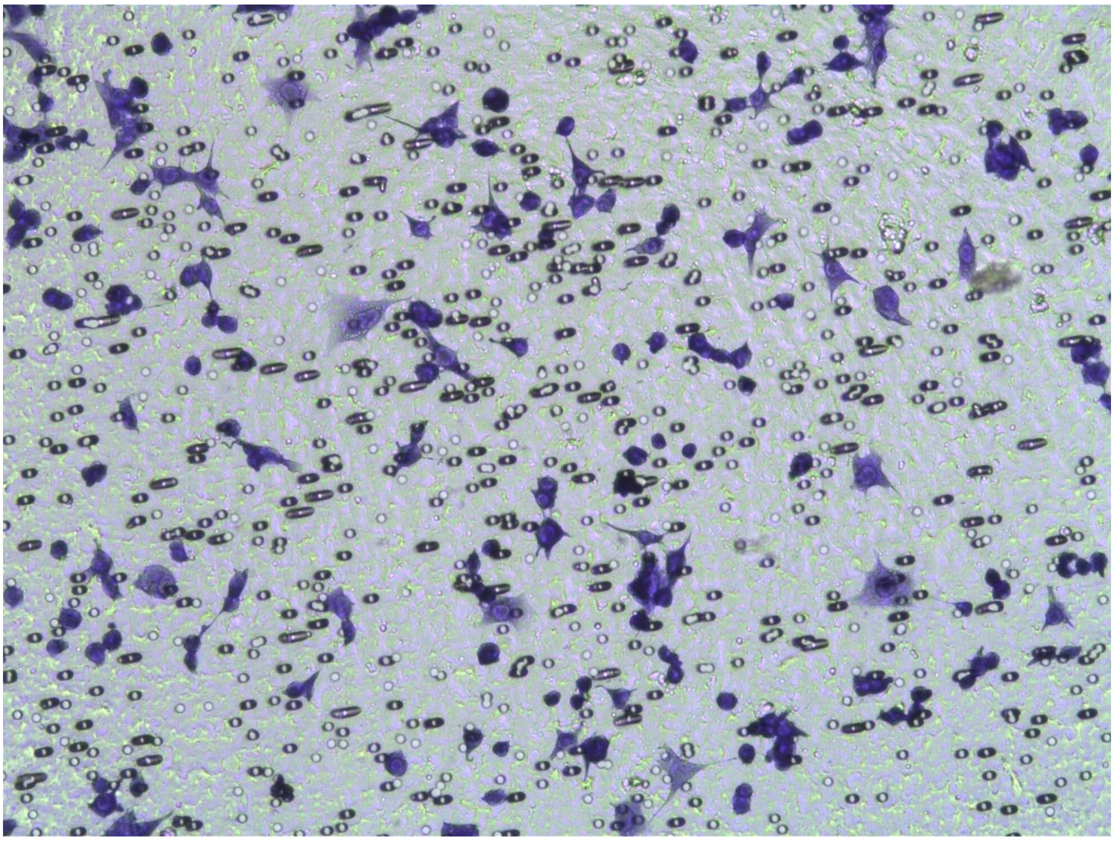

MG63 si-NC 200-4

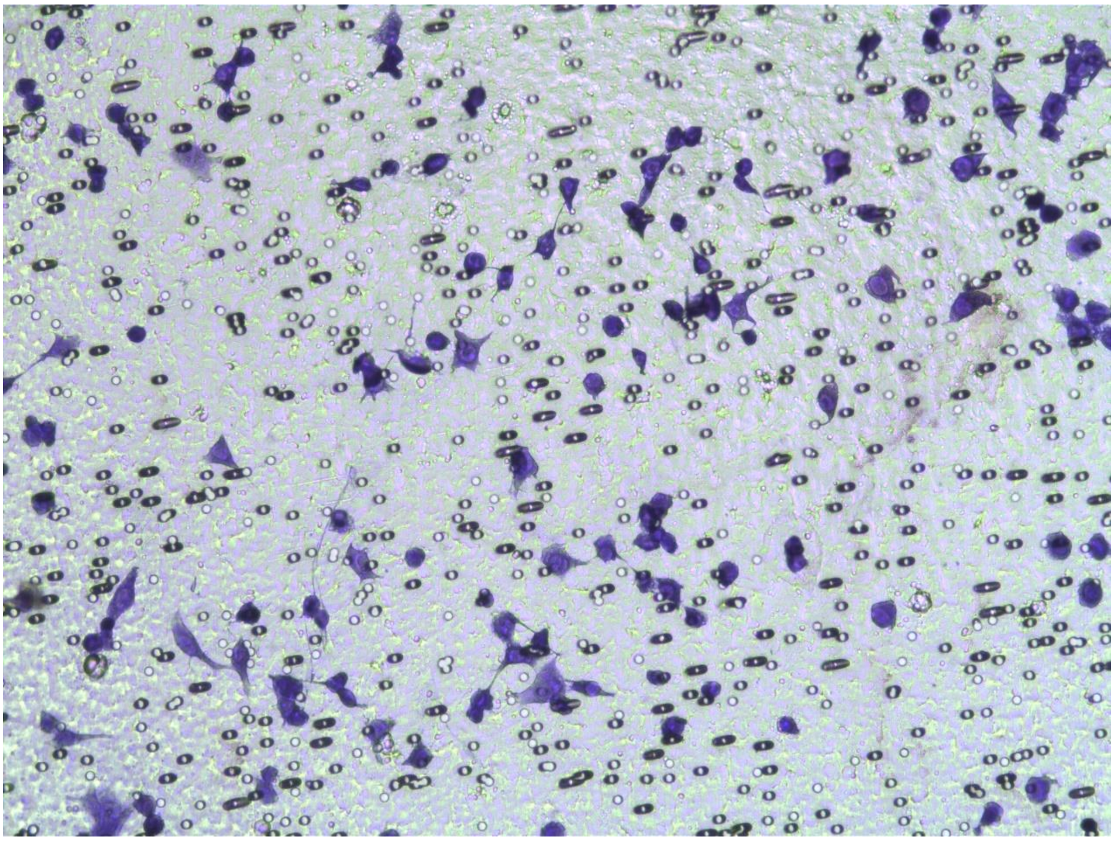

MG63 si-NC 200-5

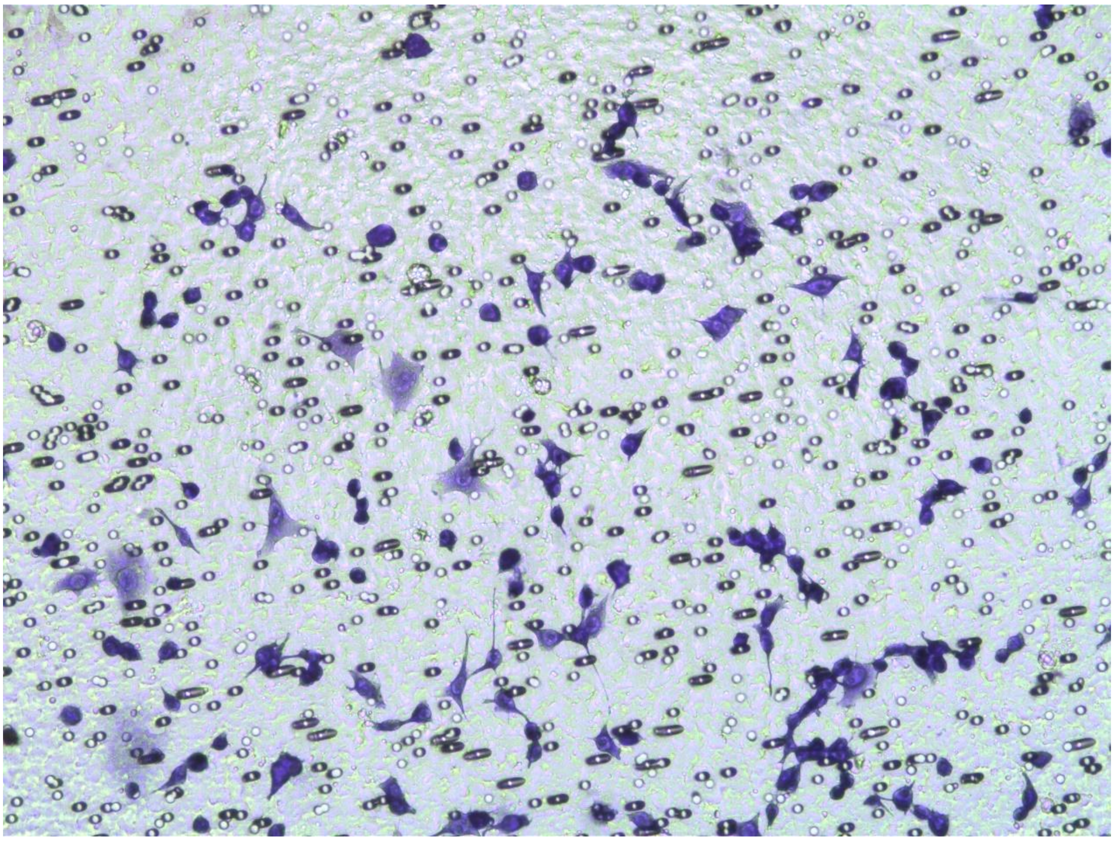

MG63 si-S100A16 200-1

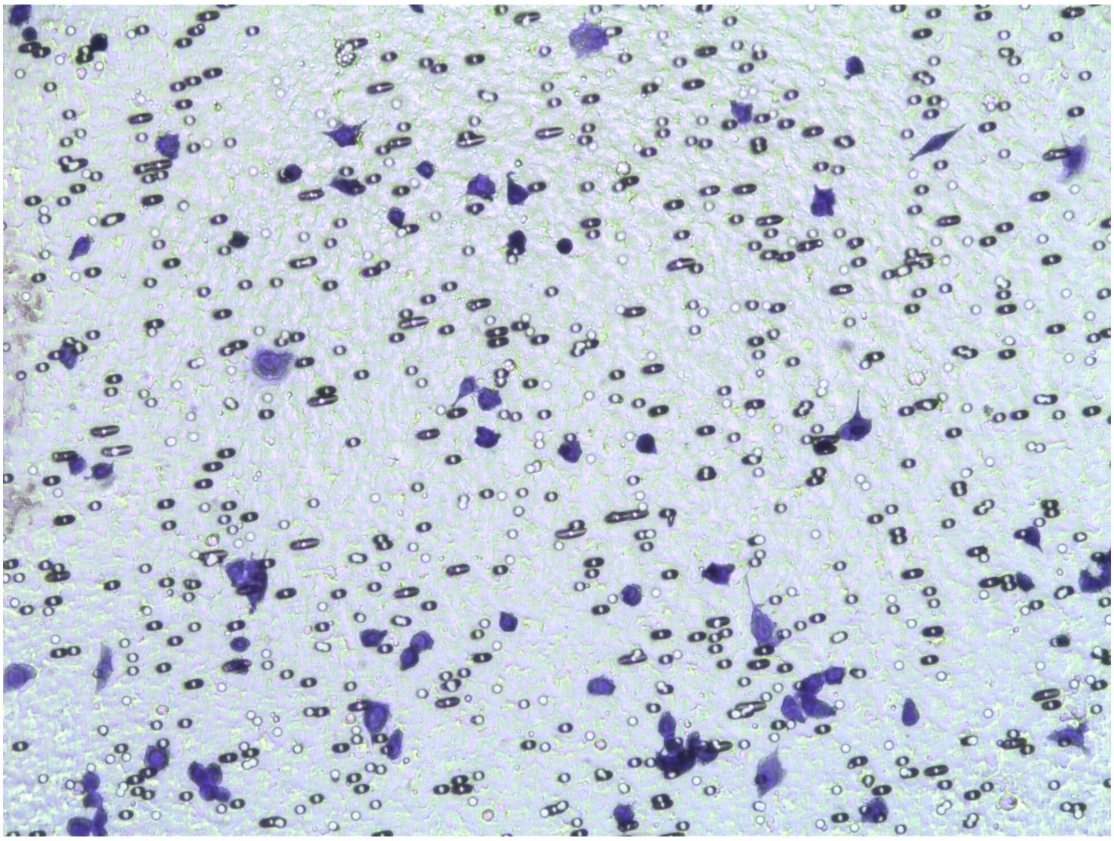

MG63 si-S100A16 200-2

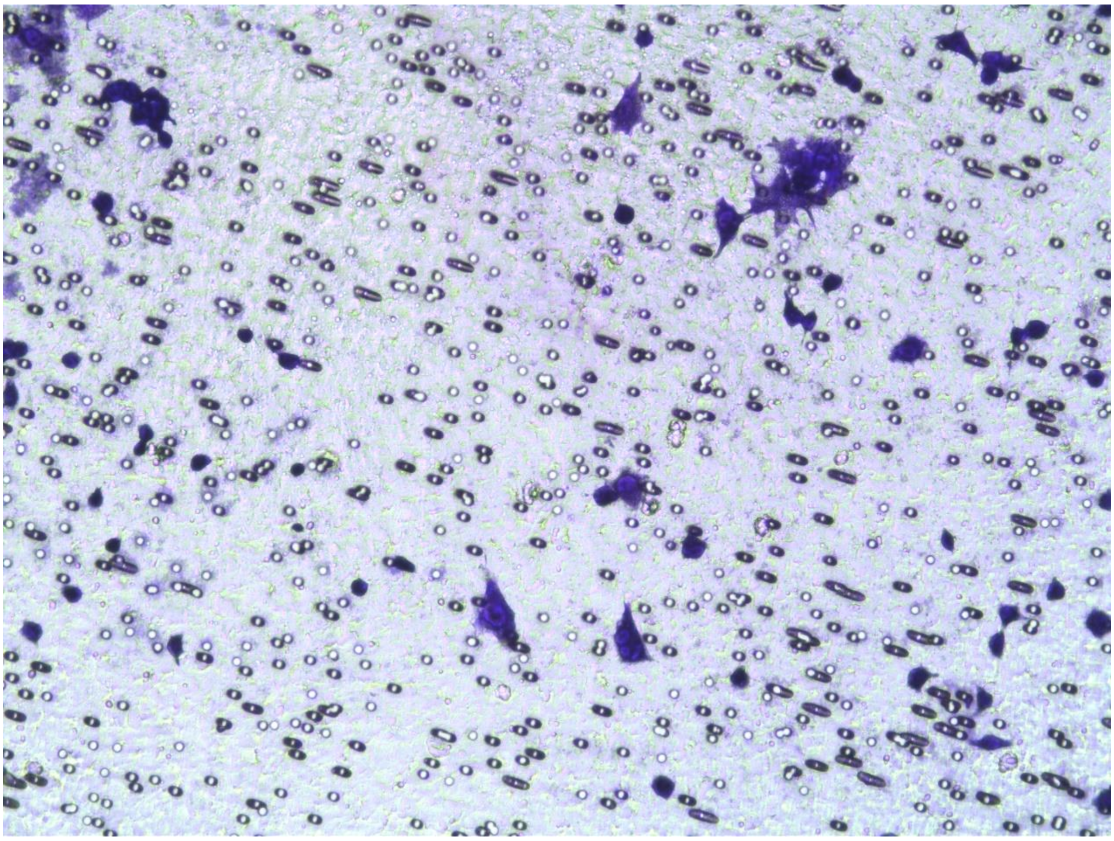

MG63 si-S100A16 200-3

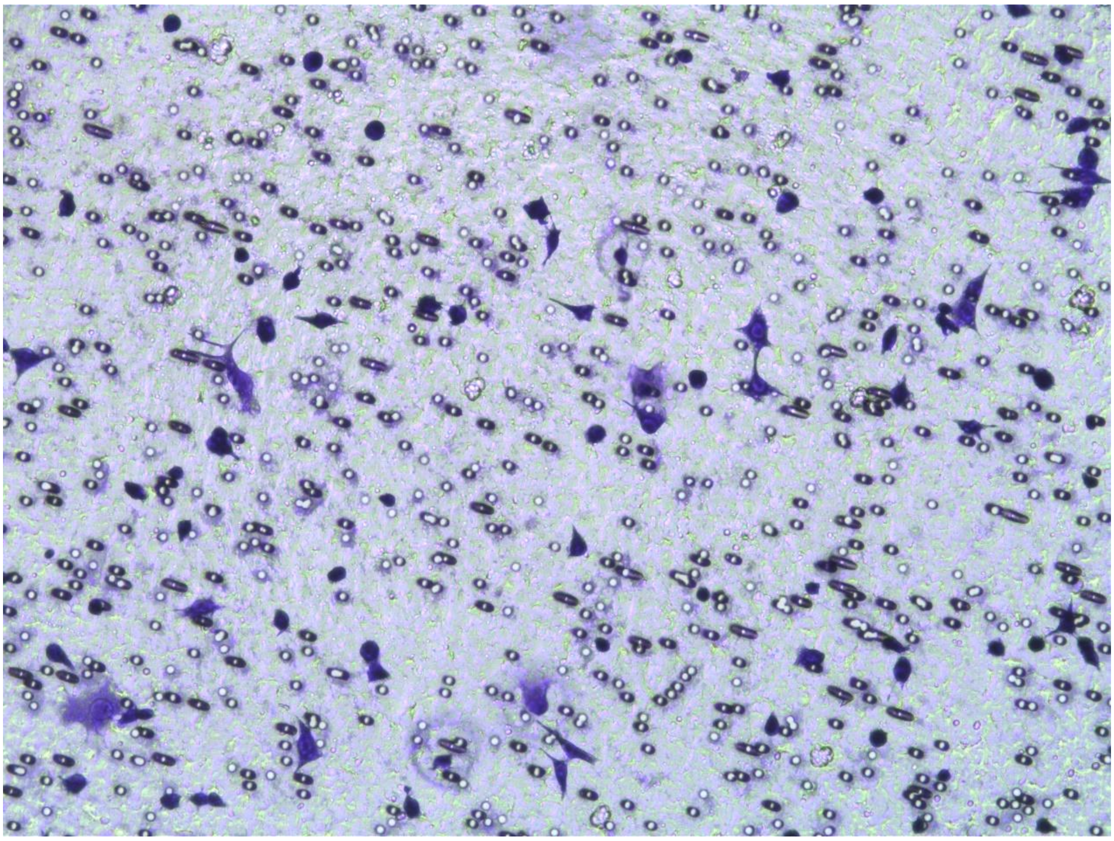

MG63 si-S100A16 200-4

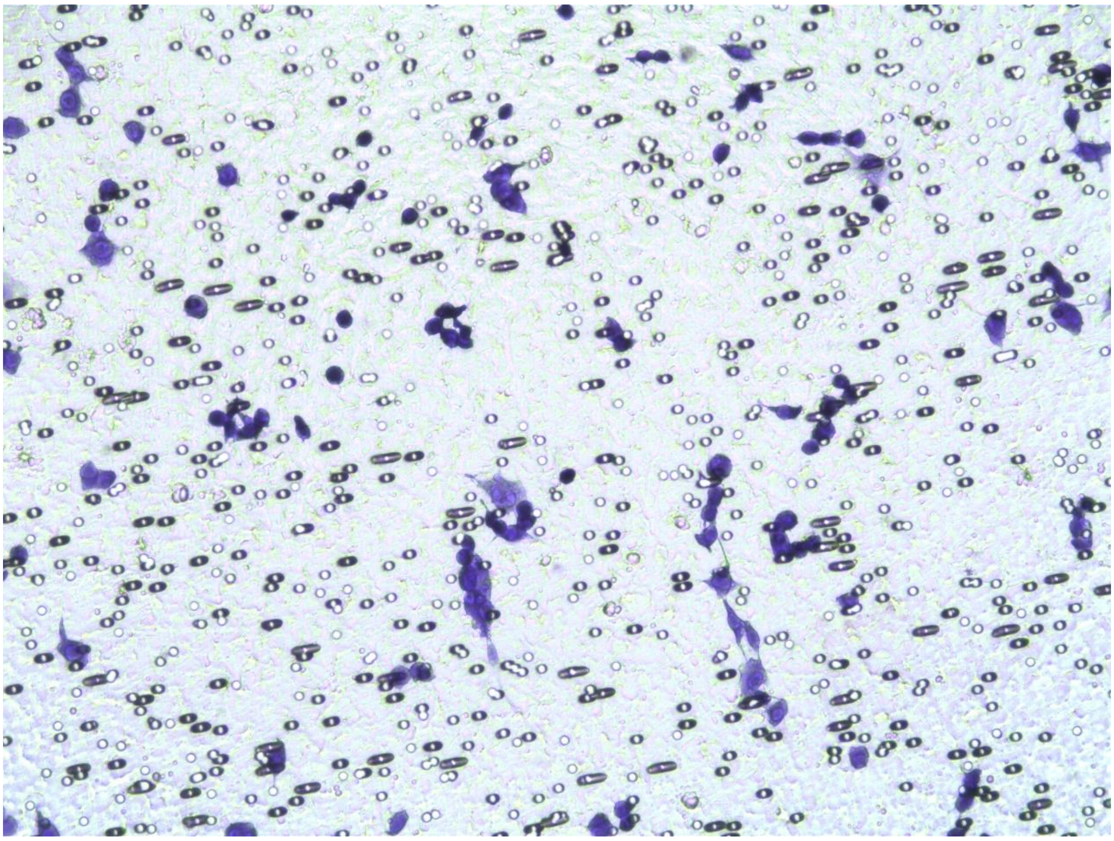

MG63 si-S100A16 200-5

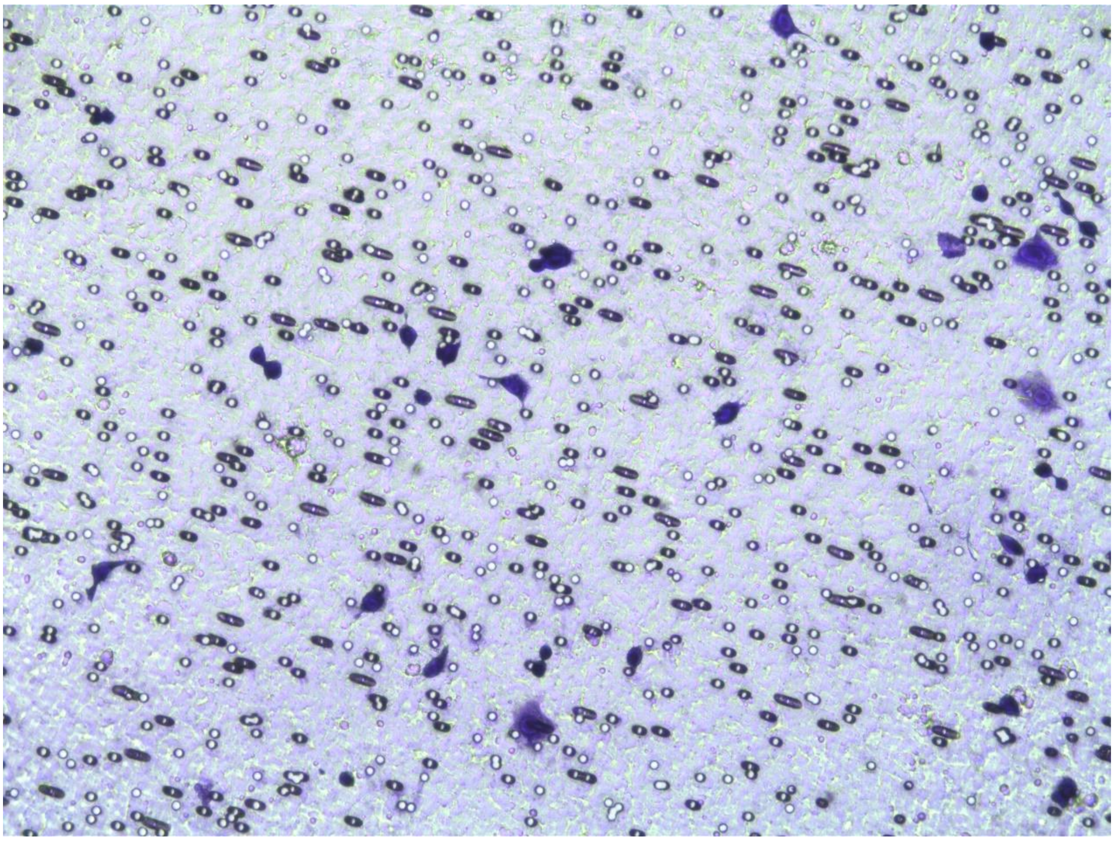

U20S si-NC 200-1

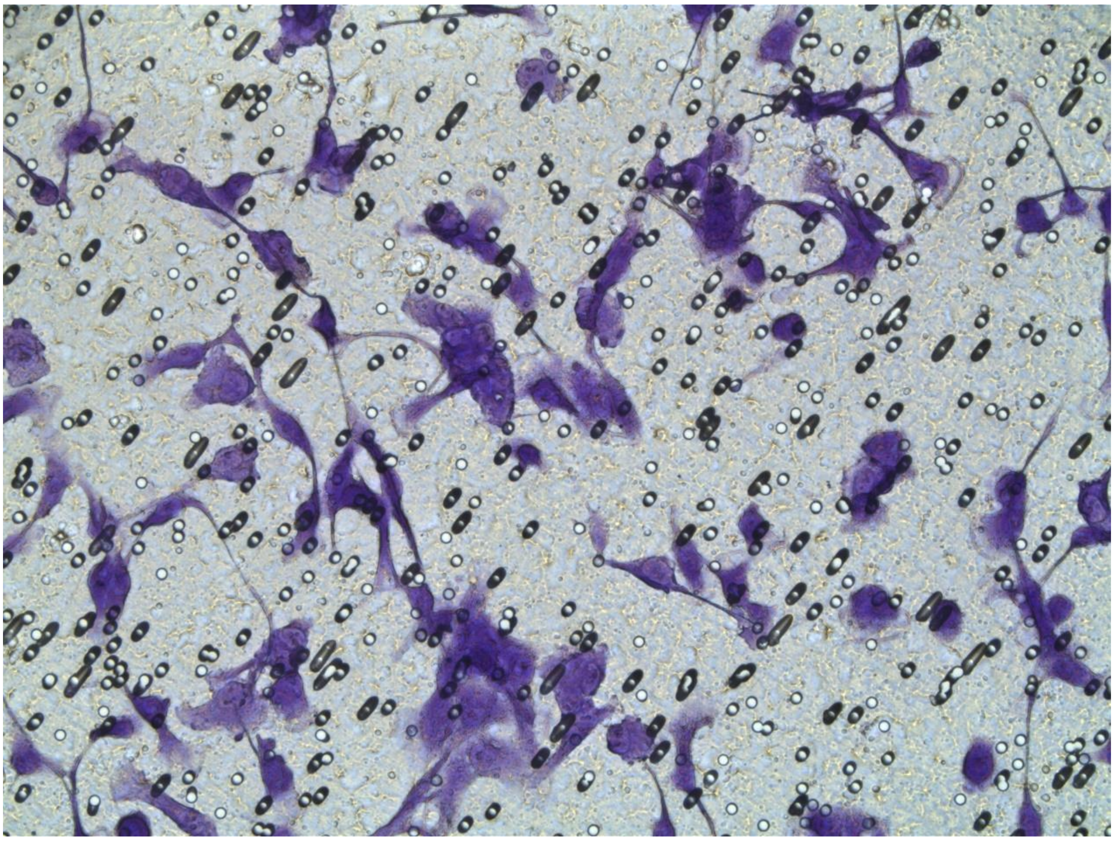

U20S si-NC 200-2

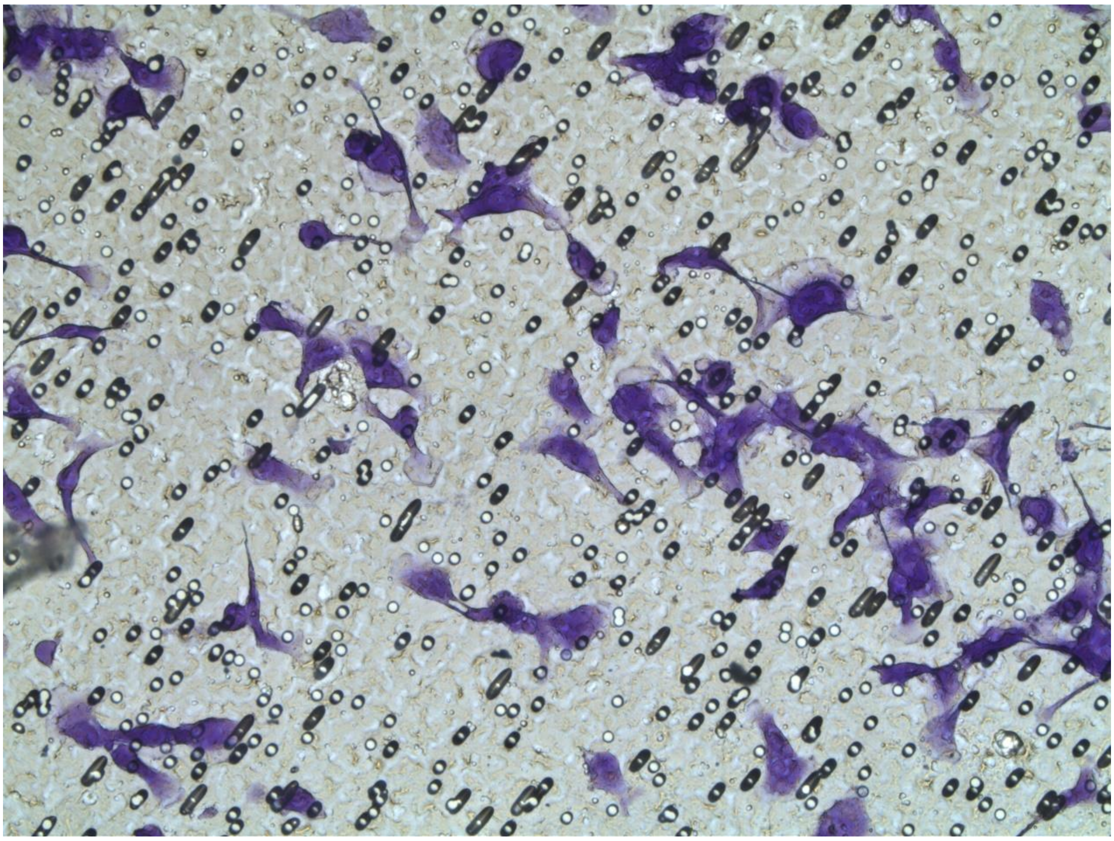

U20S si-NC 200-3

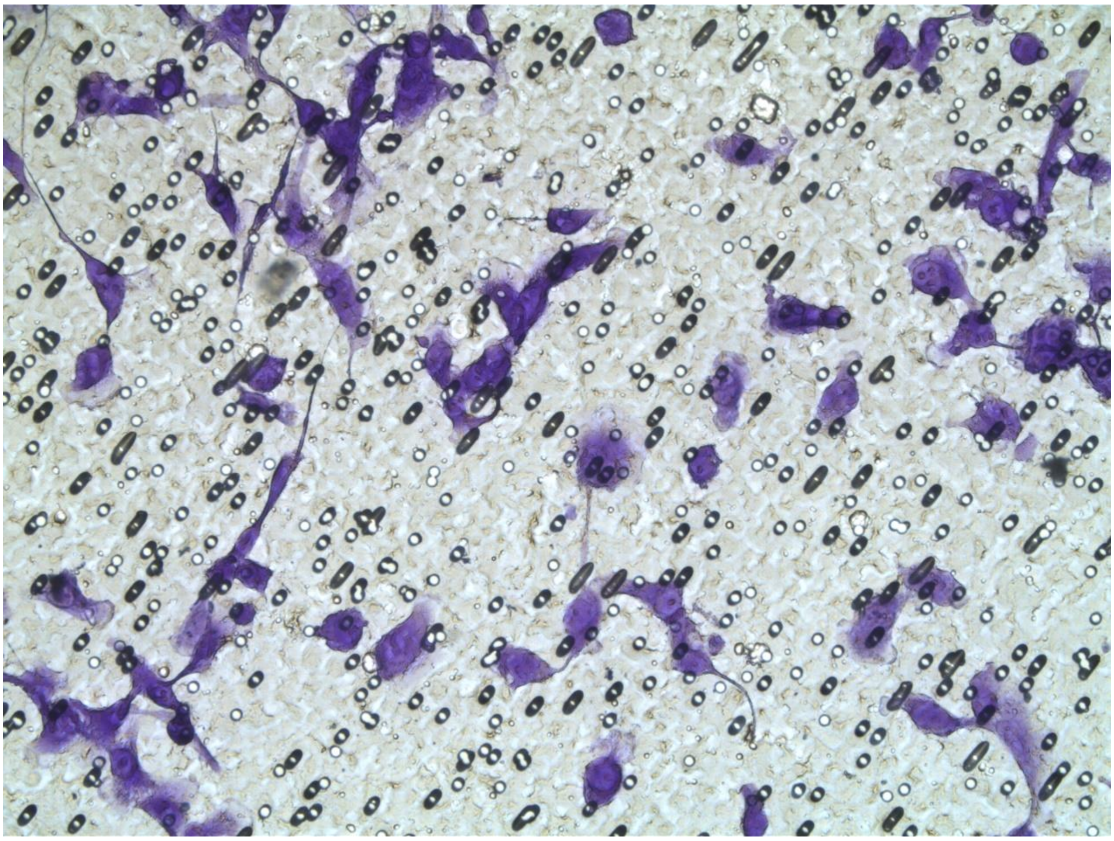

U20S si-NC 200-4

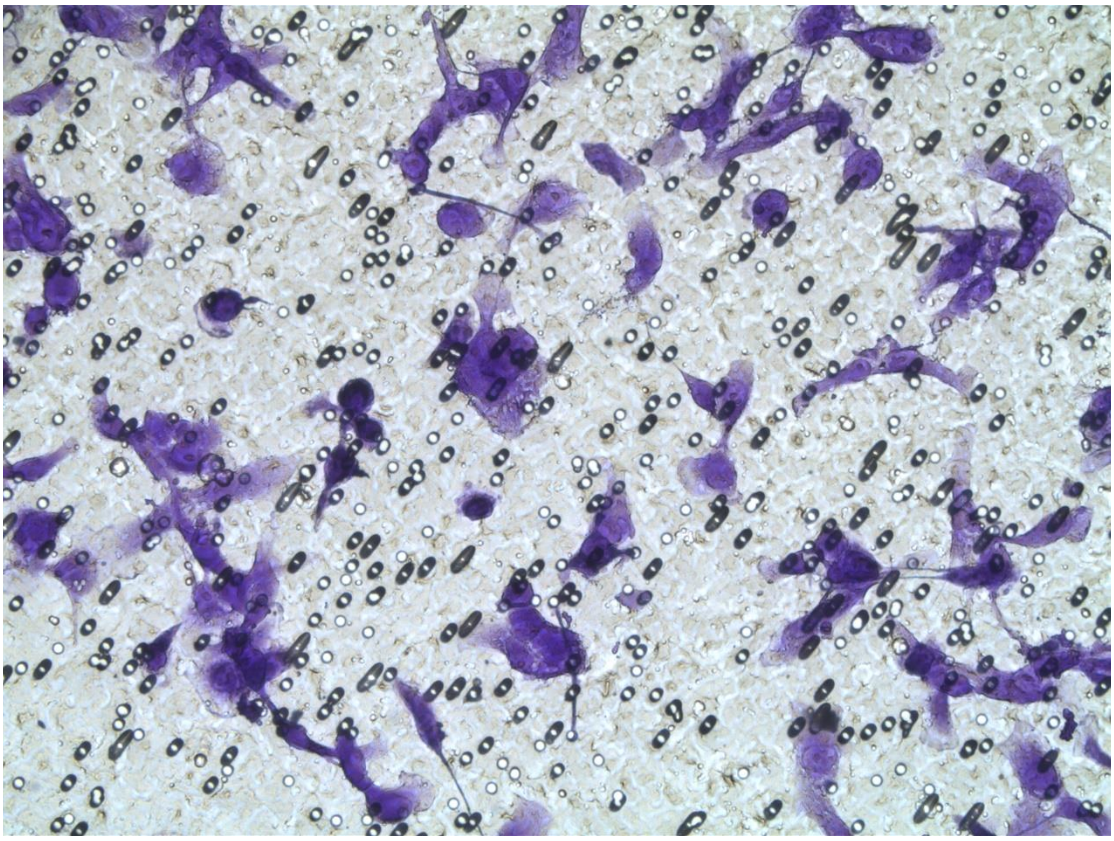

U20S si-NC 200-5

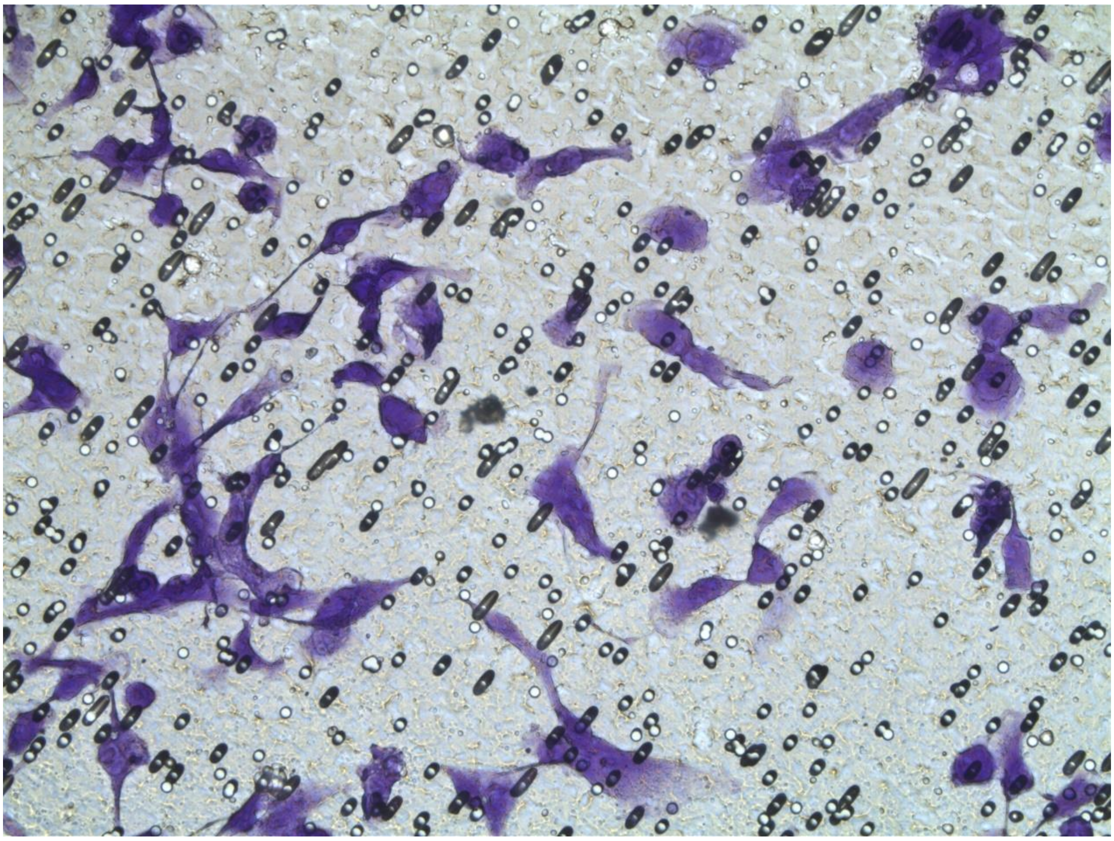

U20S si-S100A8 200-1

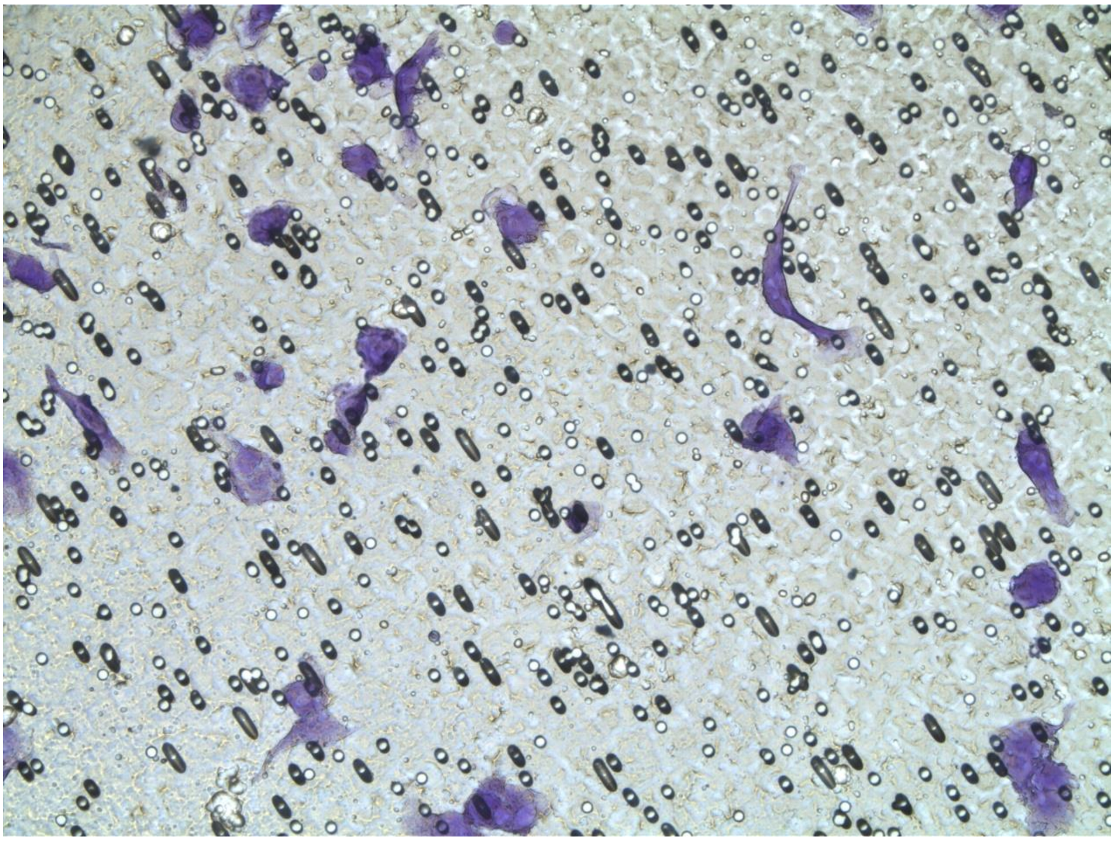

U20S si-S100A8 200-2

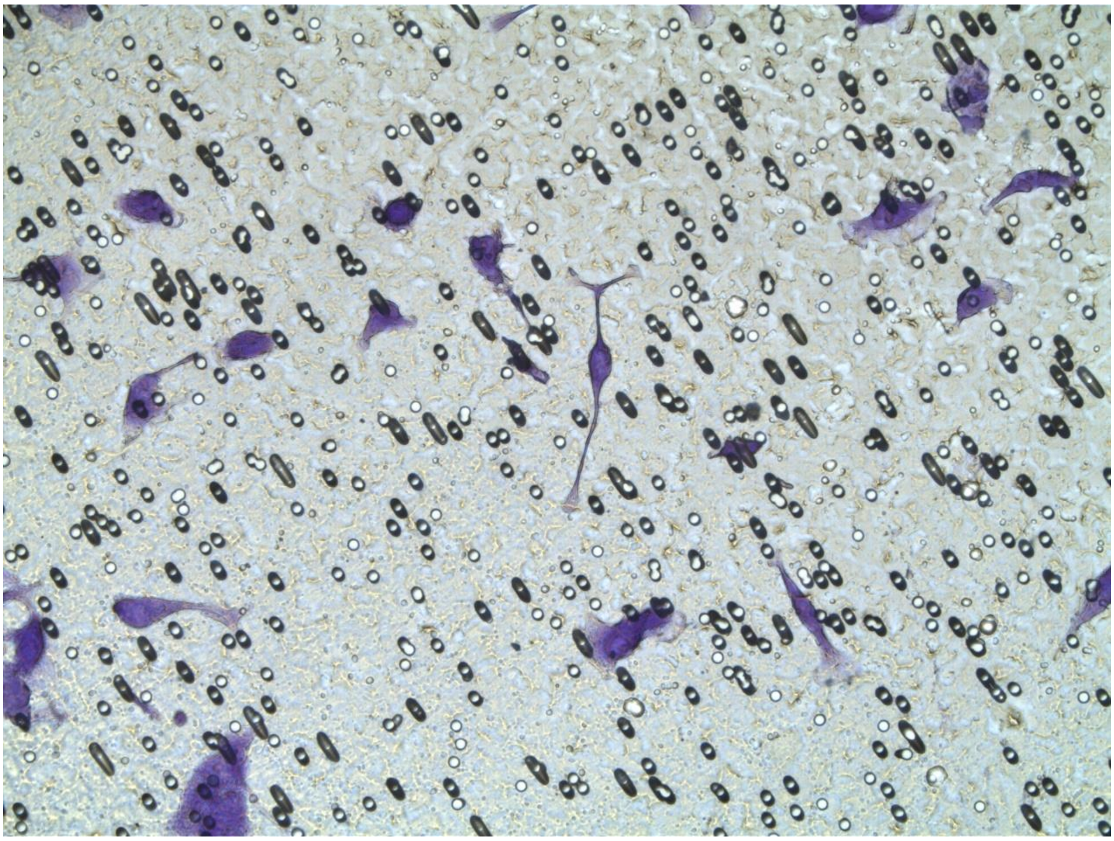

U20S si-S100A8 200-3

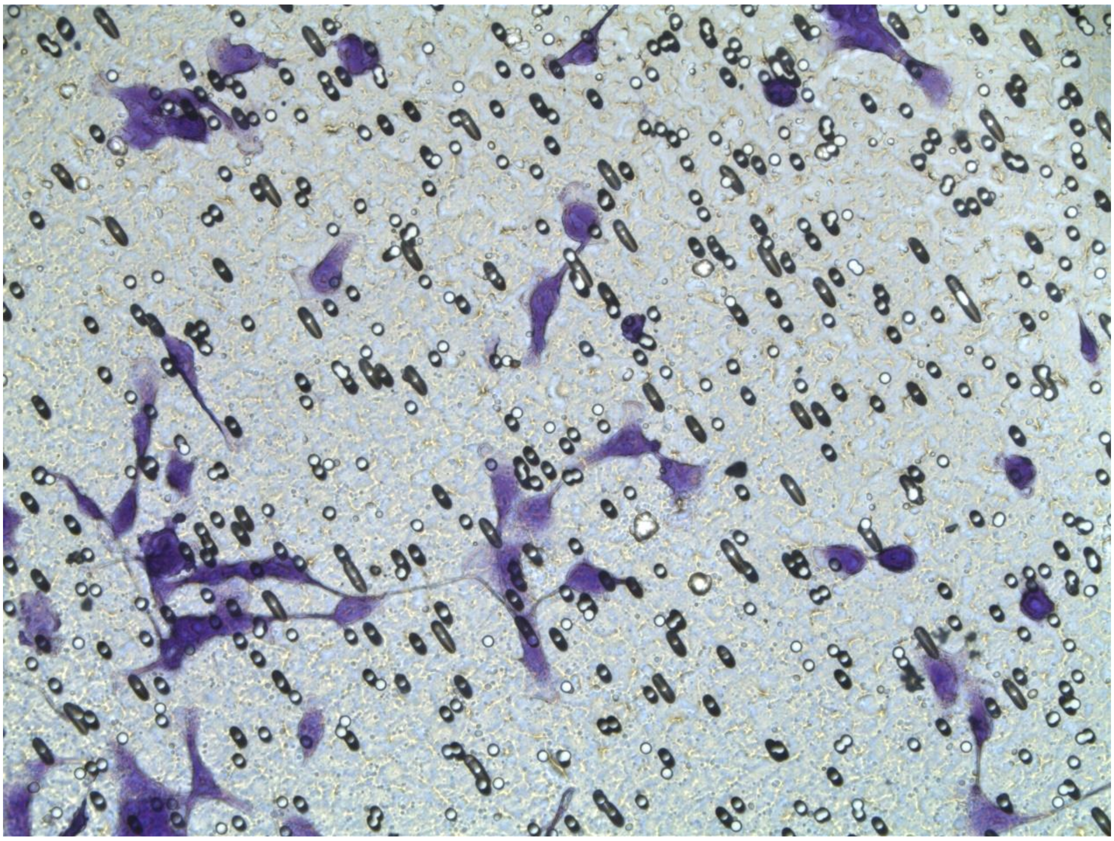

U20S si-S100A8 200-4

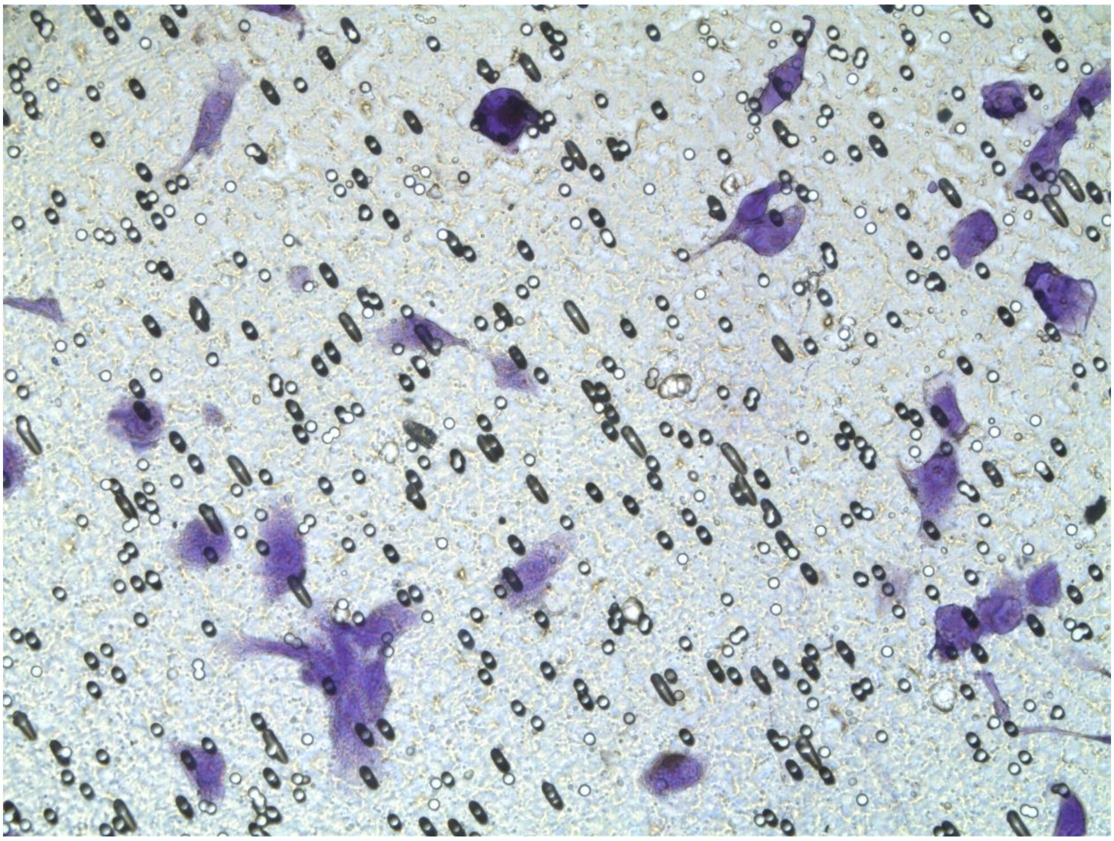

U20S si-S100A8 200-5

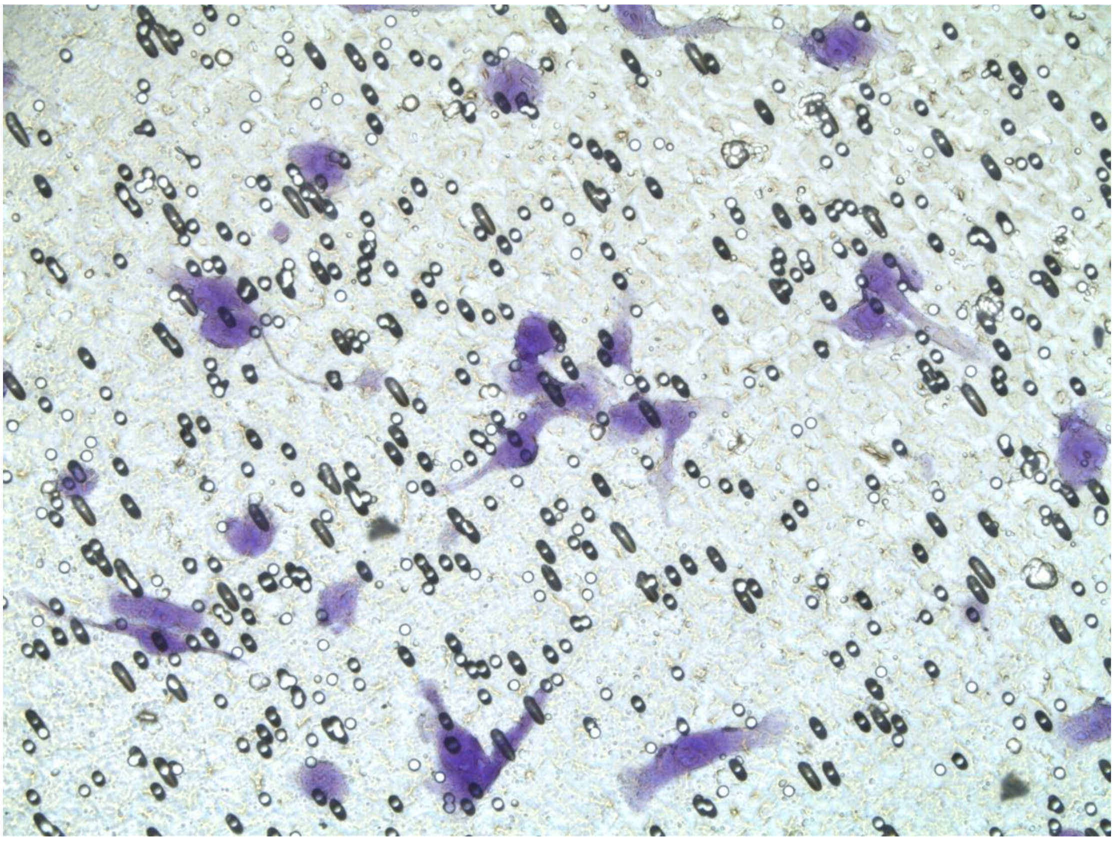

Fig 3 WB

GAPDH

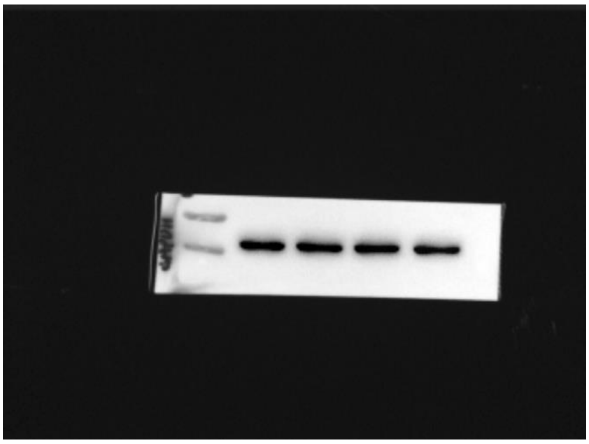

S100A16

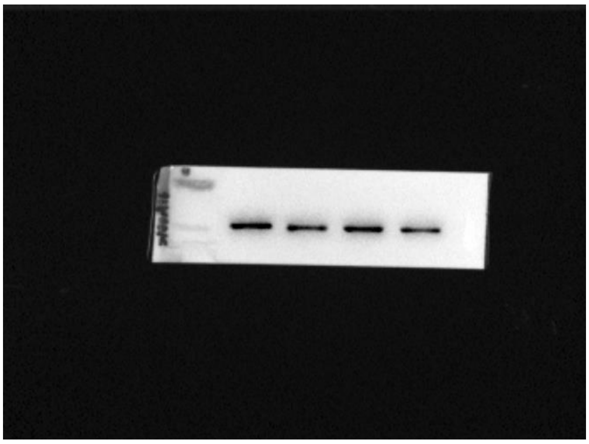

Fig 5 transwell

MG63 NC 200-1

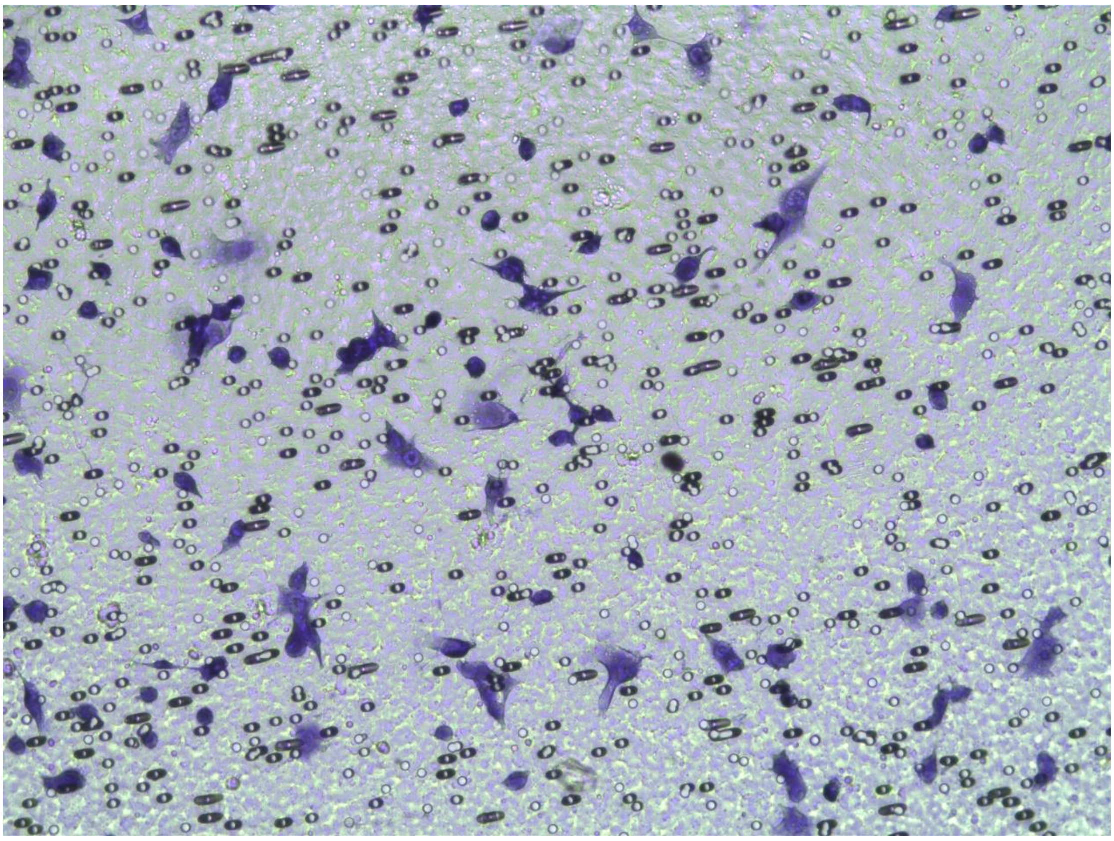

MG63 NC 200-2

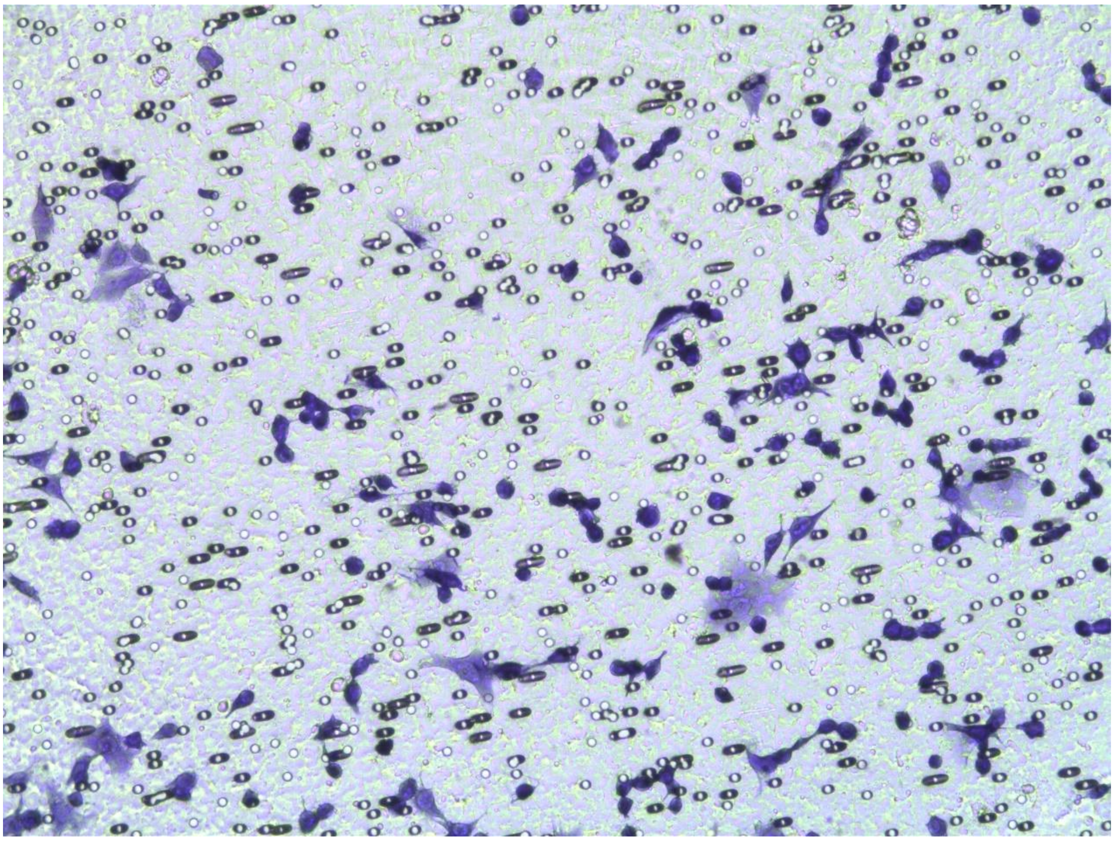

MG63 NC 200-3

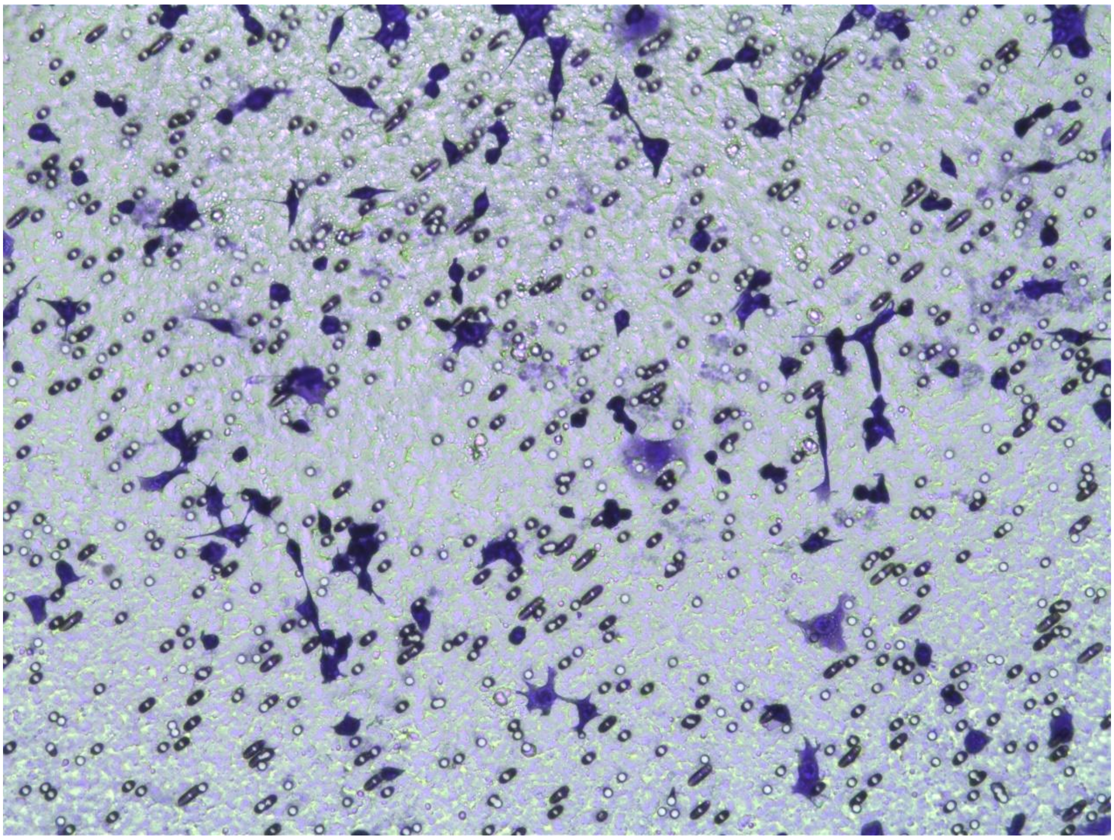

MG63 NC 200-4

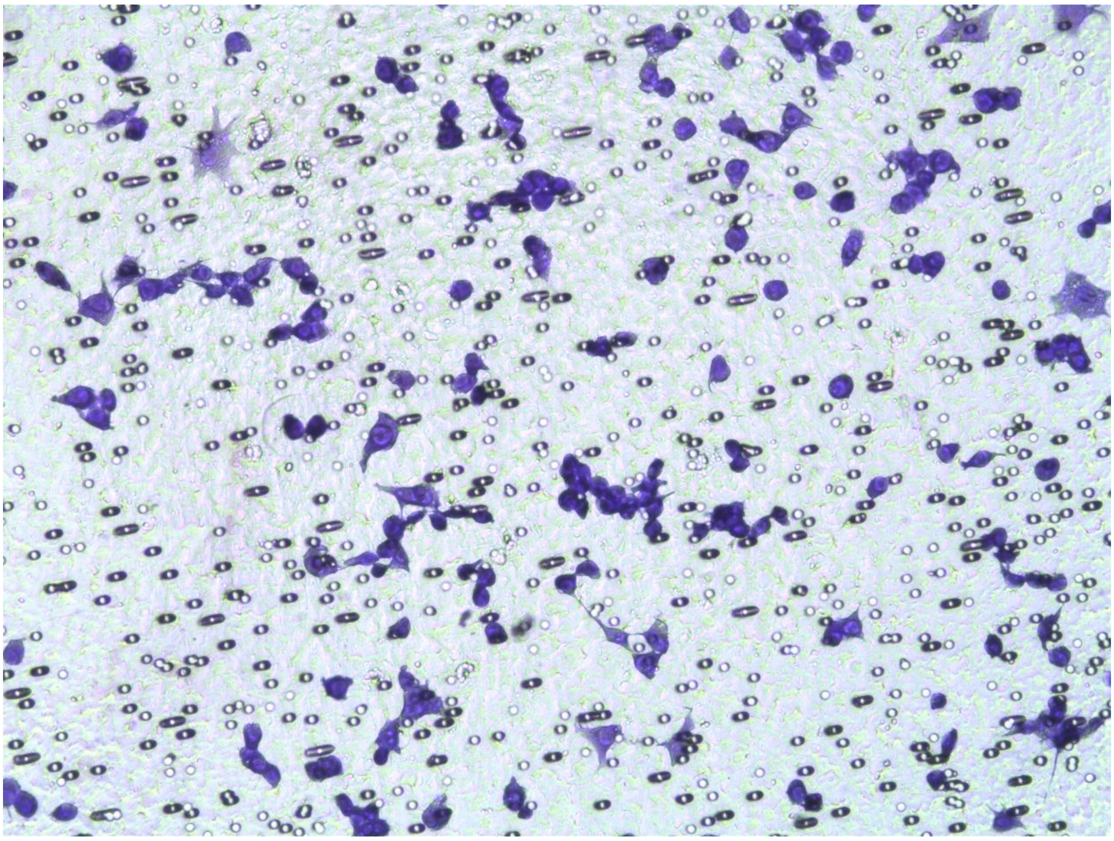

MG63 NC 200-5

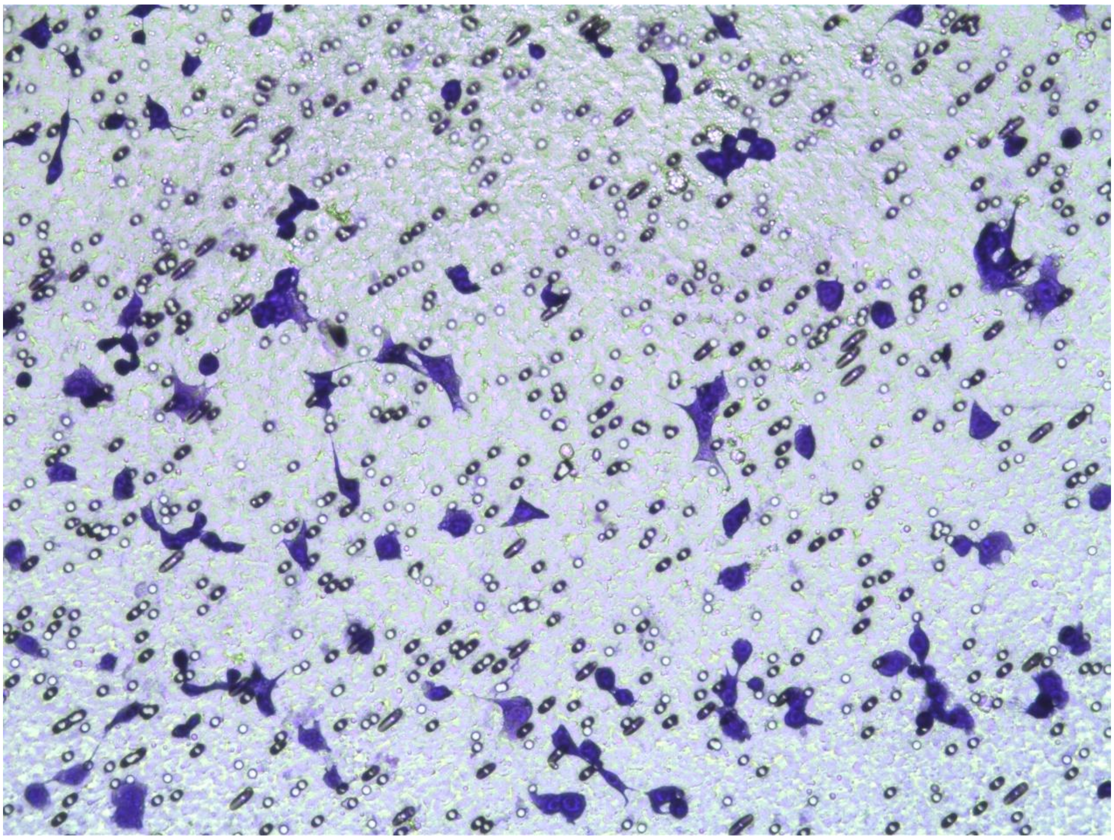

MG63 OE 200-1

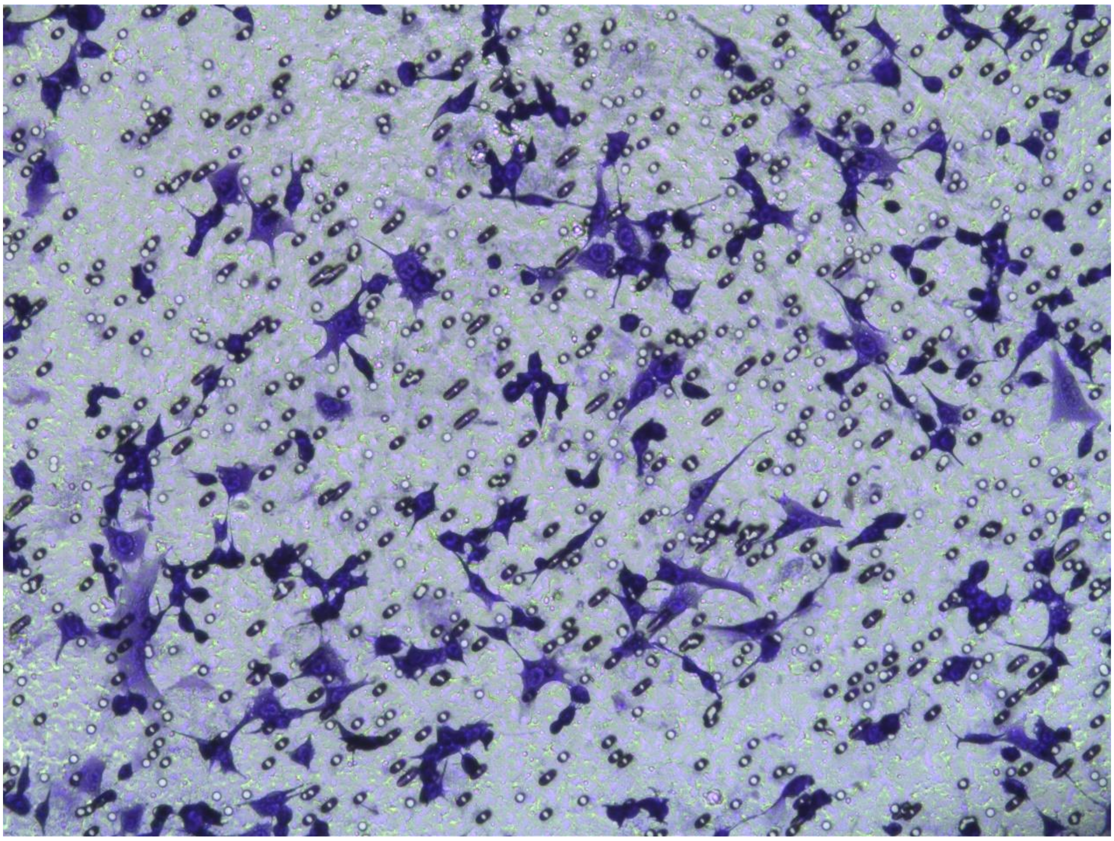

MG63 OE 200-2

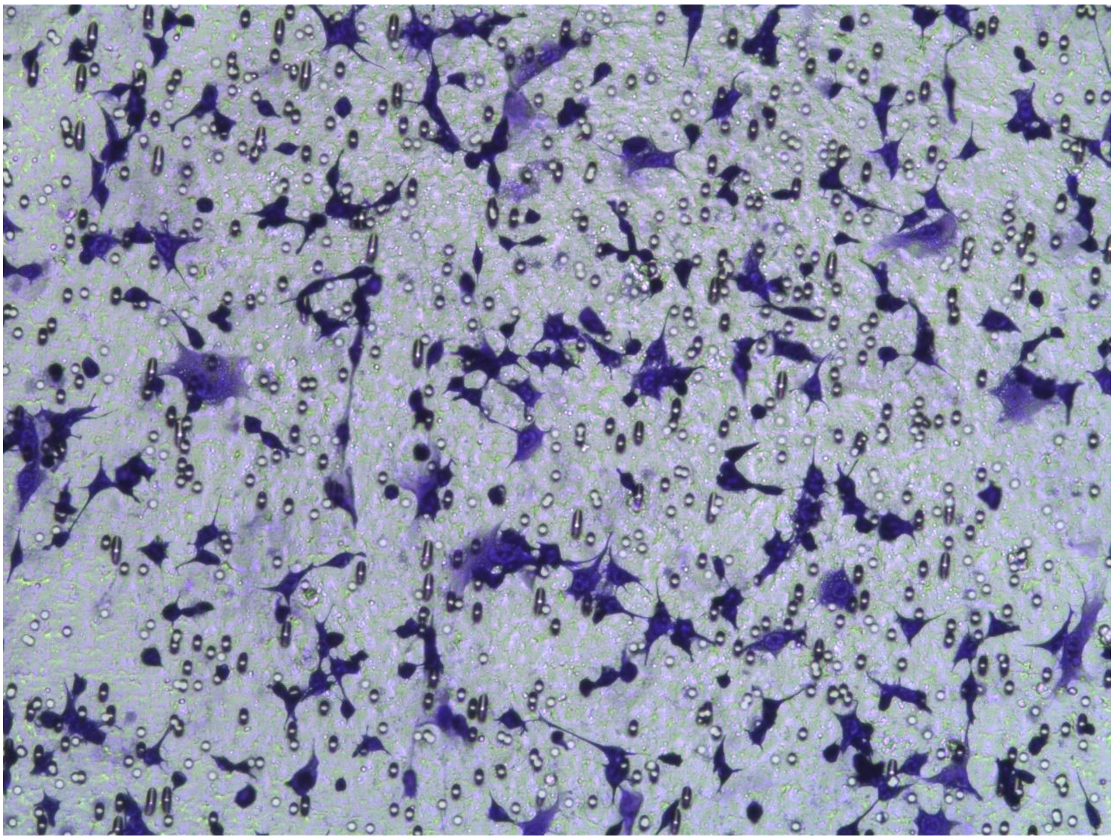

MG63 OE 200-3

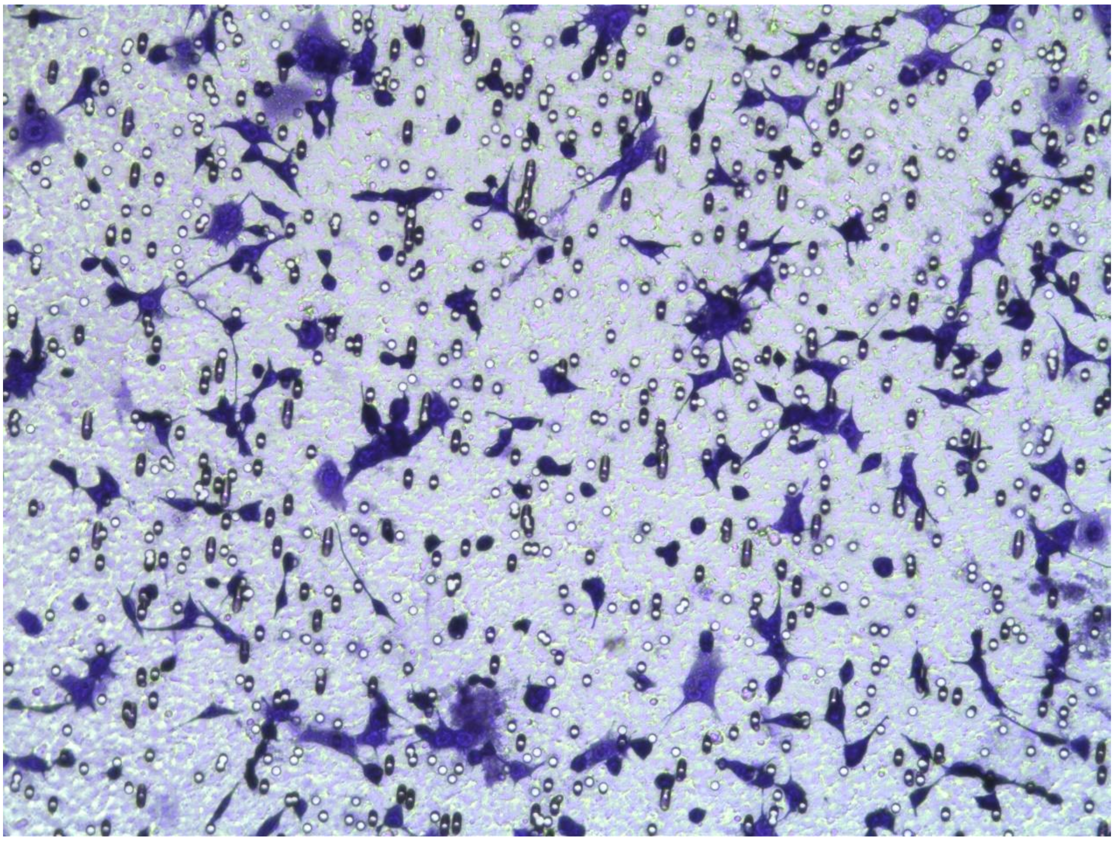

MG63 OE 200-4

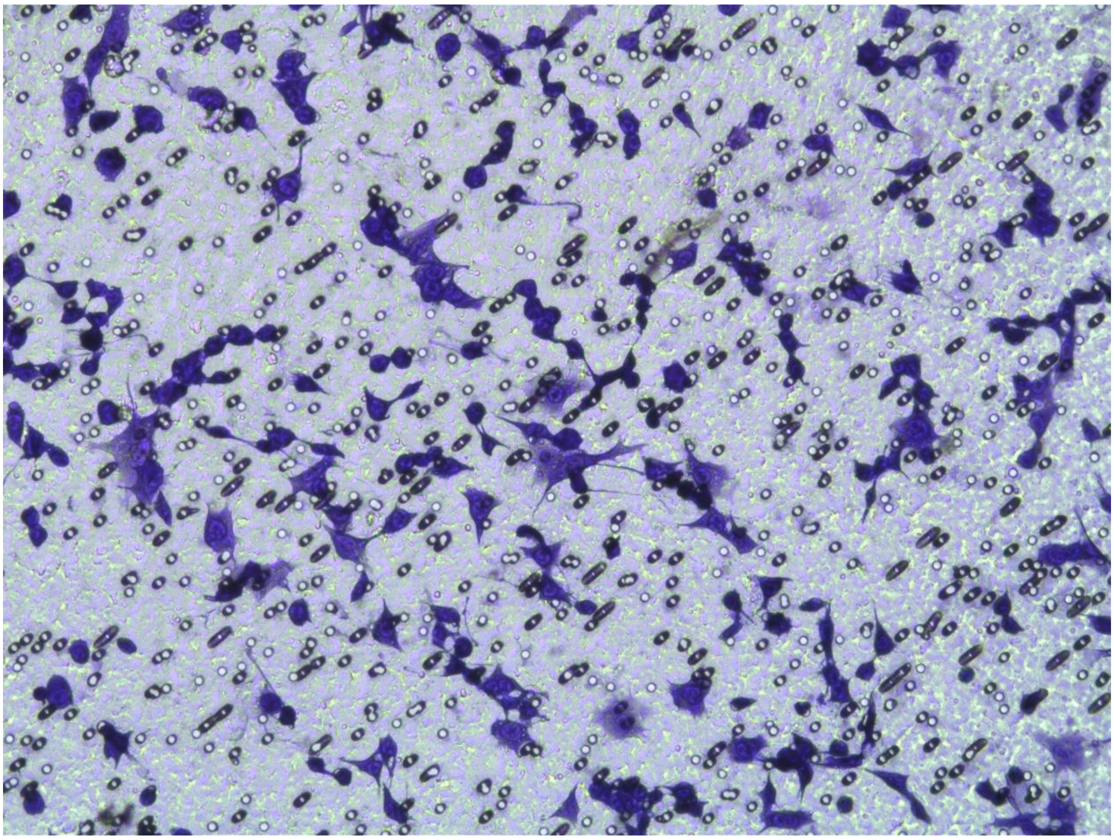

MG63 OE 200-5

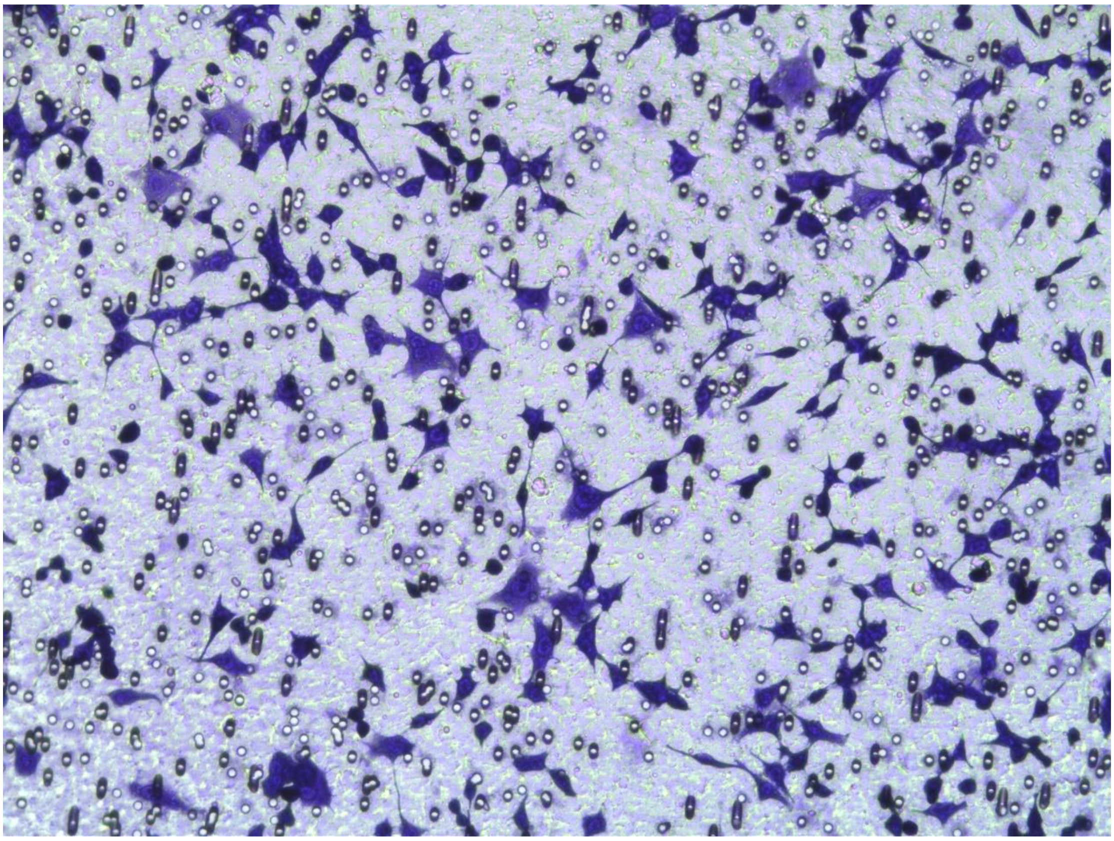

MG63 OE+DMSO 200-1

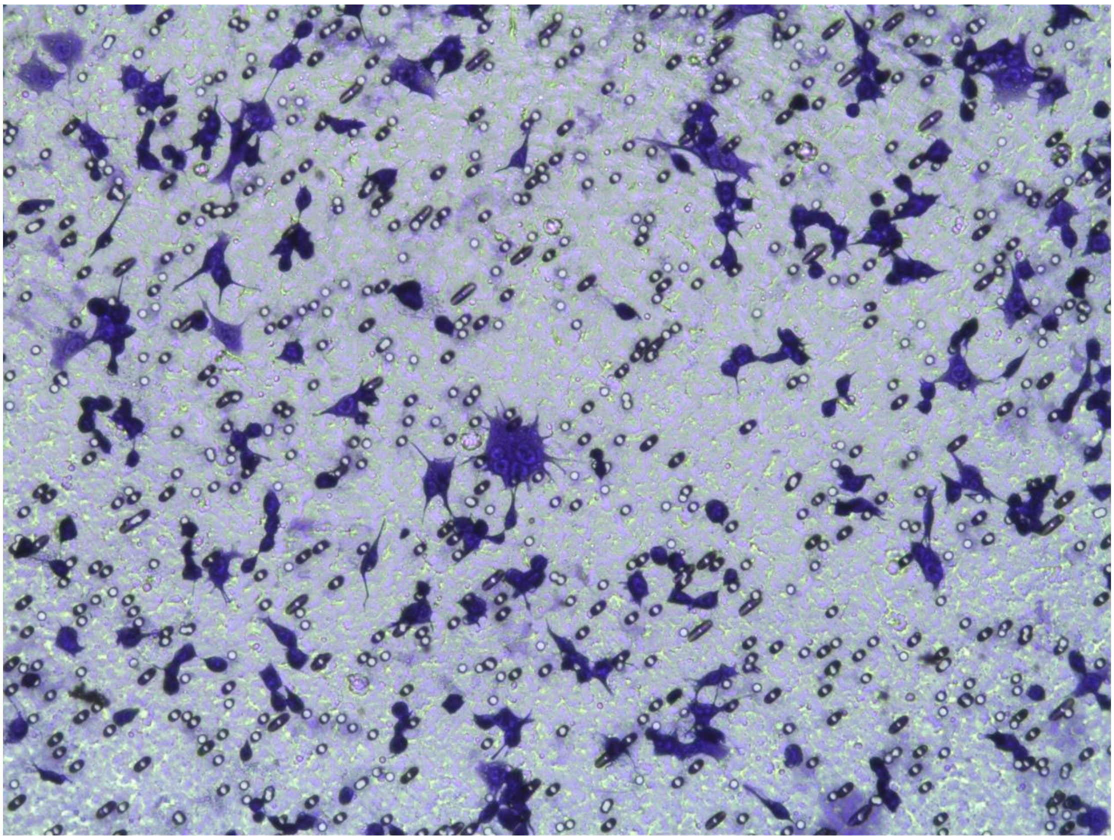

MG63 OE+DMSO 200-2

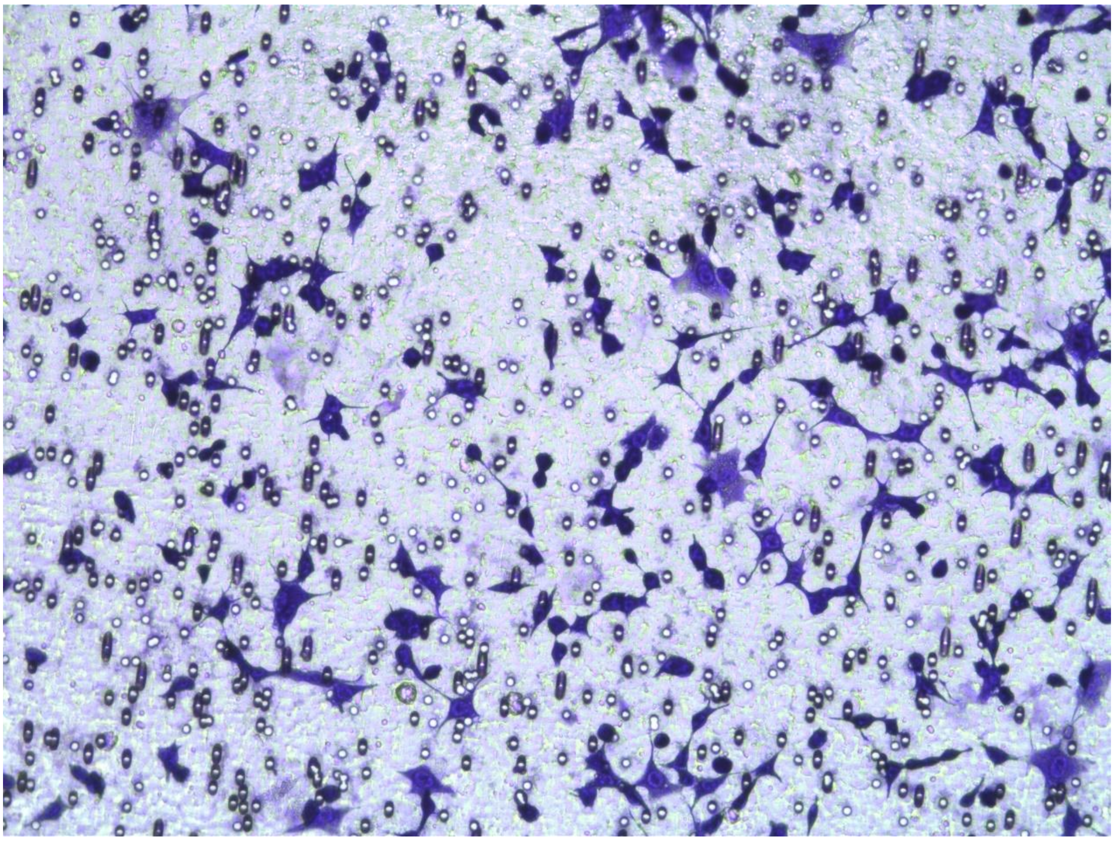

MG63 OE+DMSO 200-3

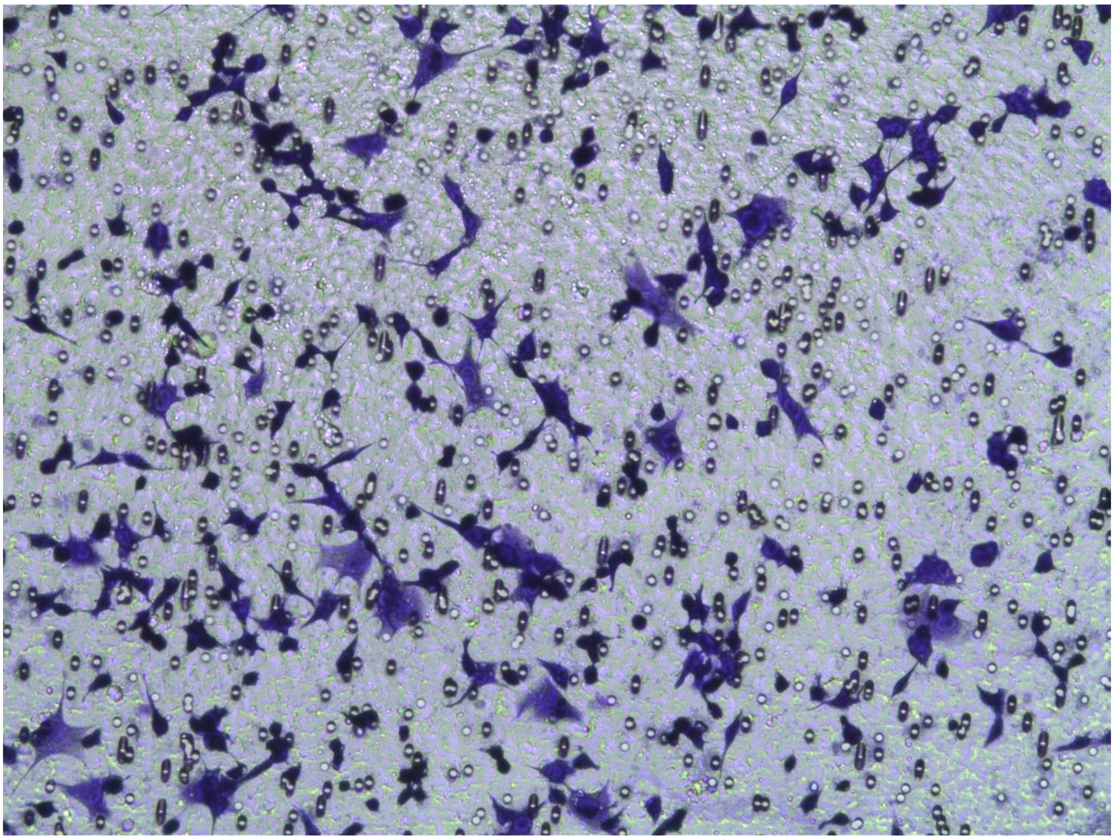

MG63 OE+DMSO 200-4

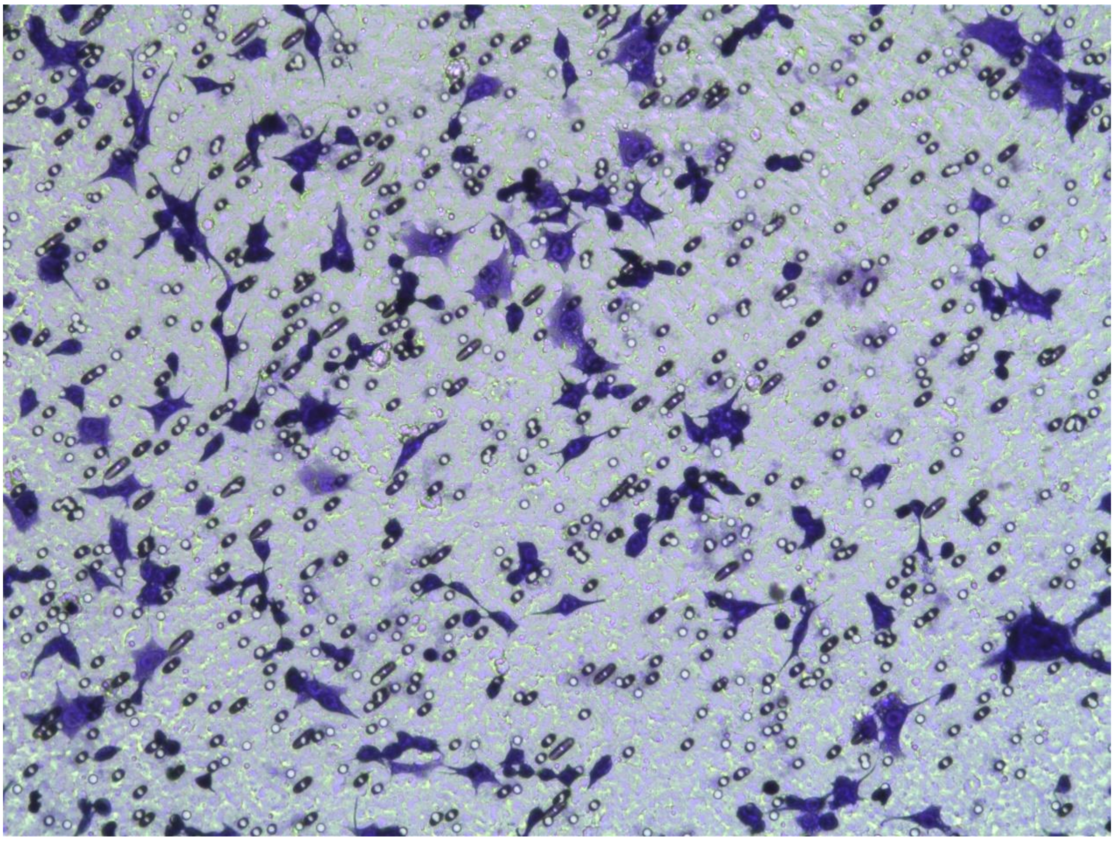

MG63 OE+DMSO 200-5

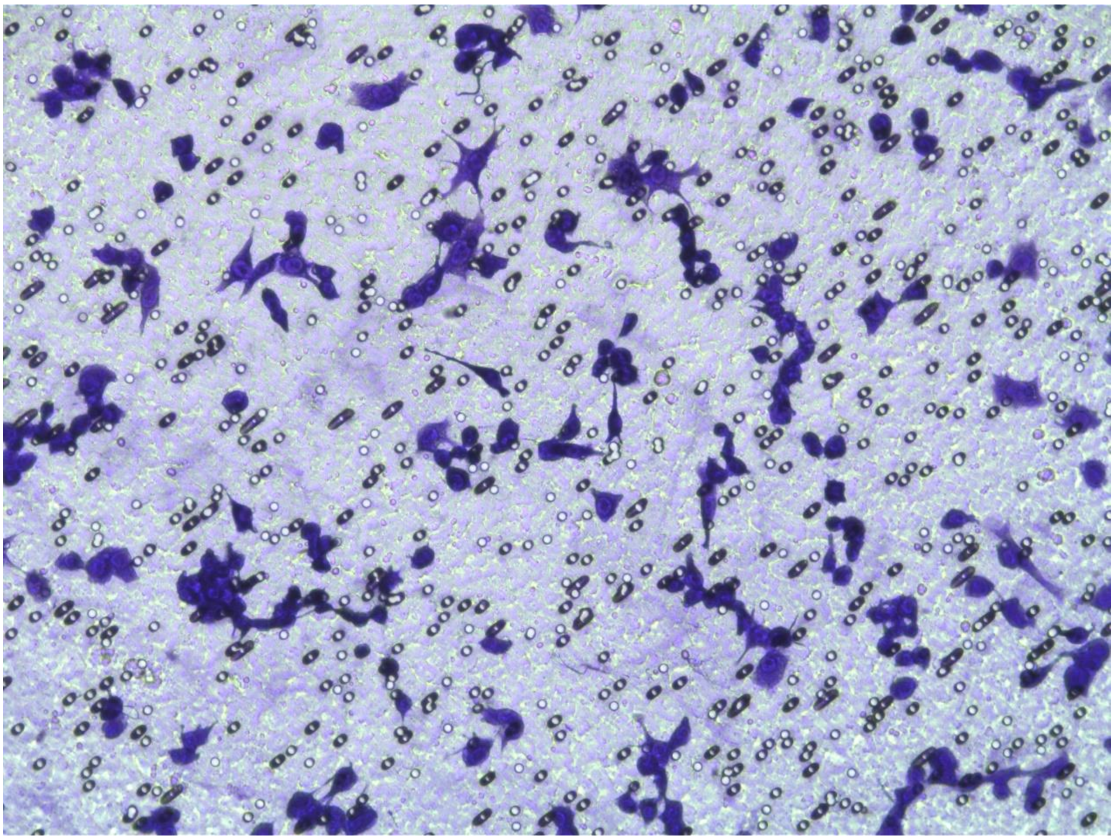

MG63 OE+PI3K 200-1

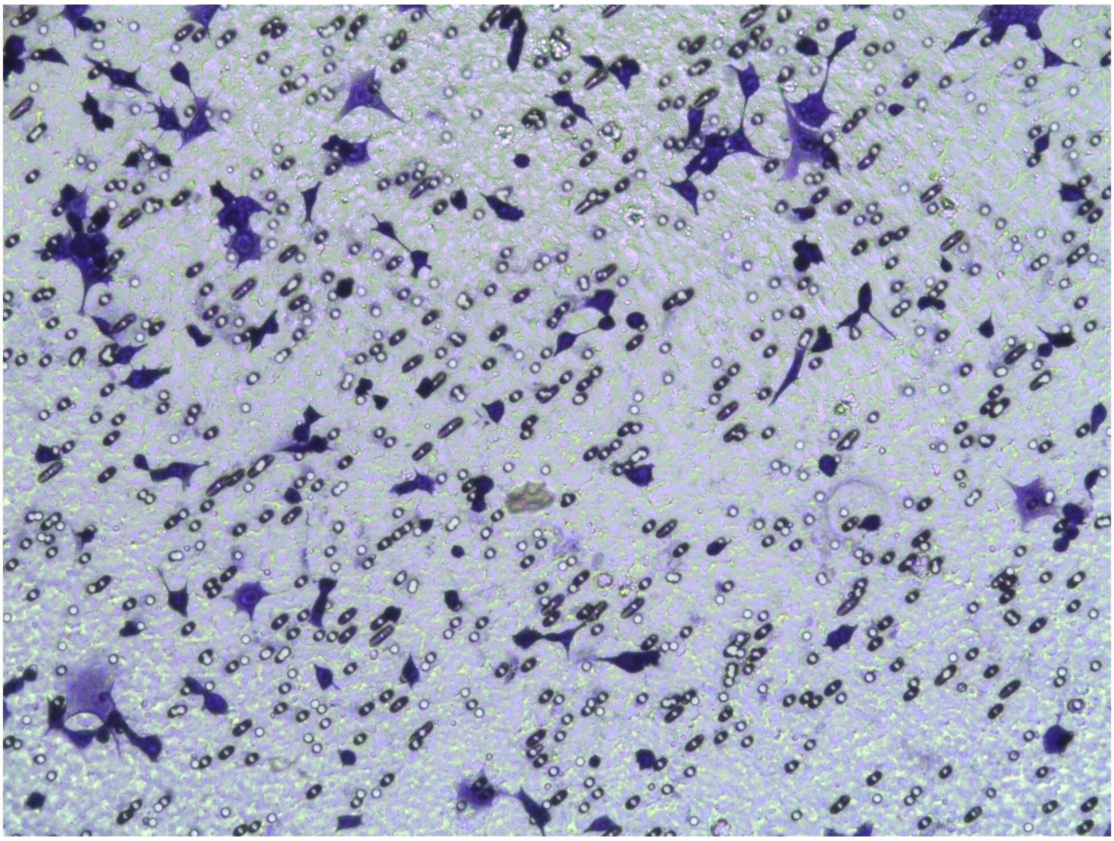

MG63 OE+PI3K 200-2

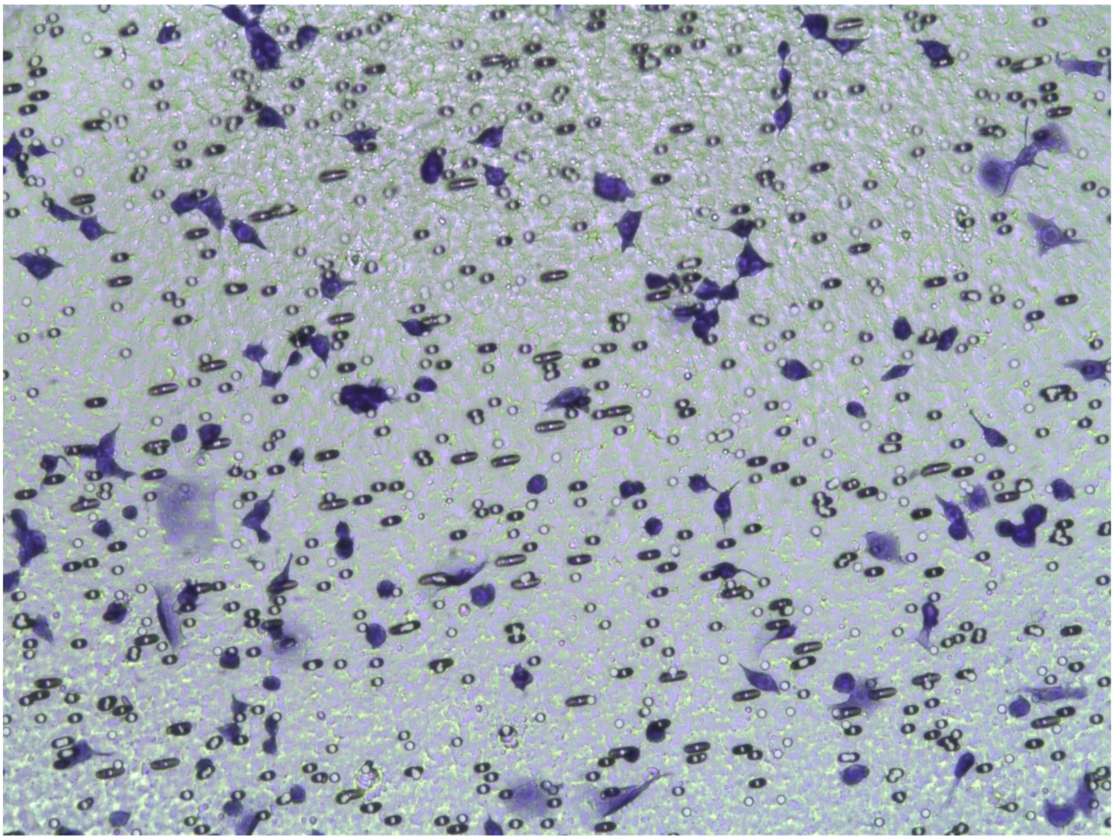

MG63 OE+PI3K 200-3

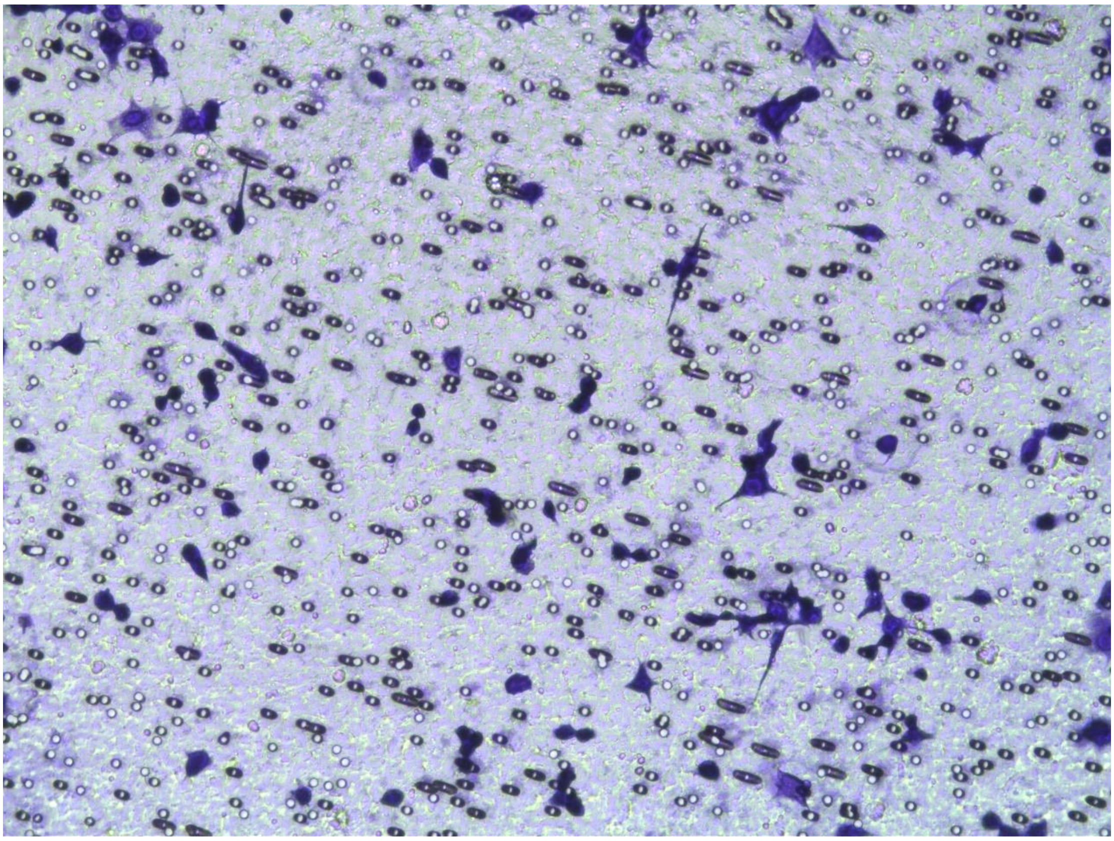

MG63 OE+PI3K 200-4

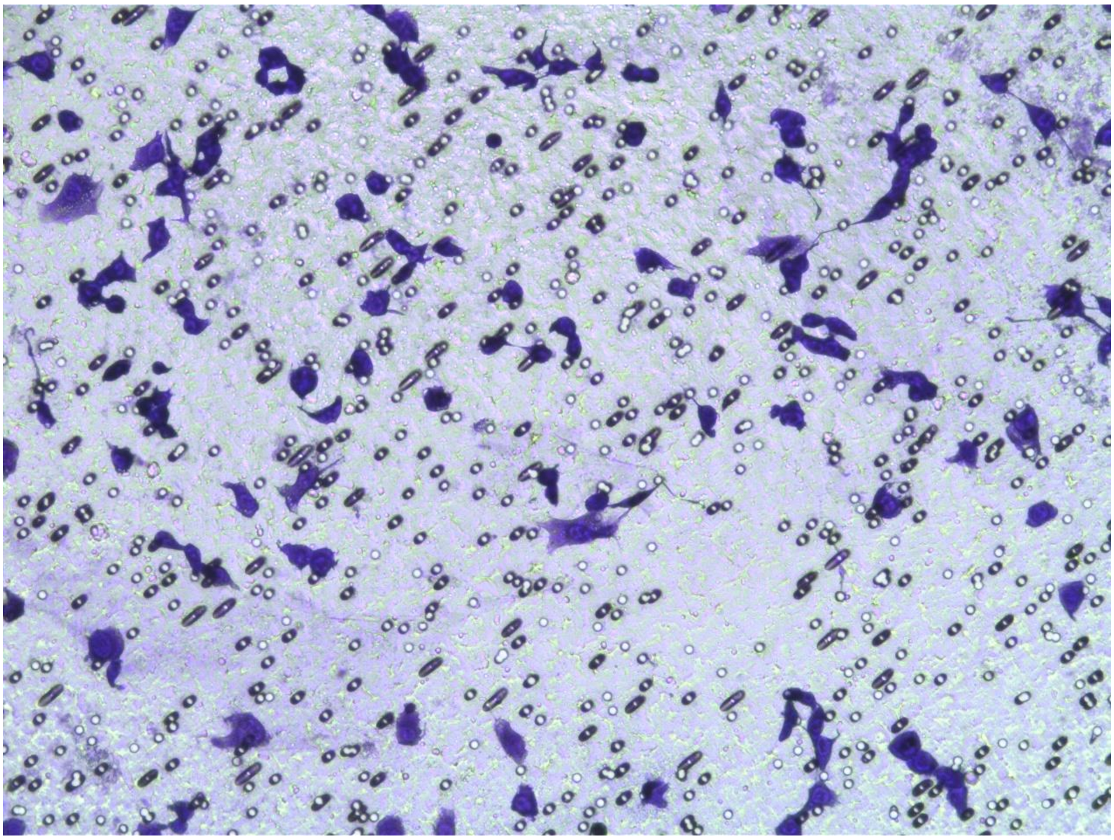

MG63 OE+PI3K 200-5

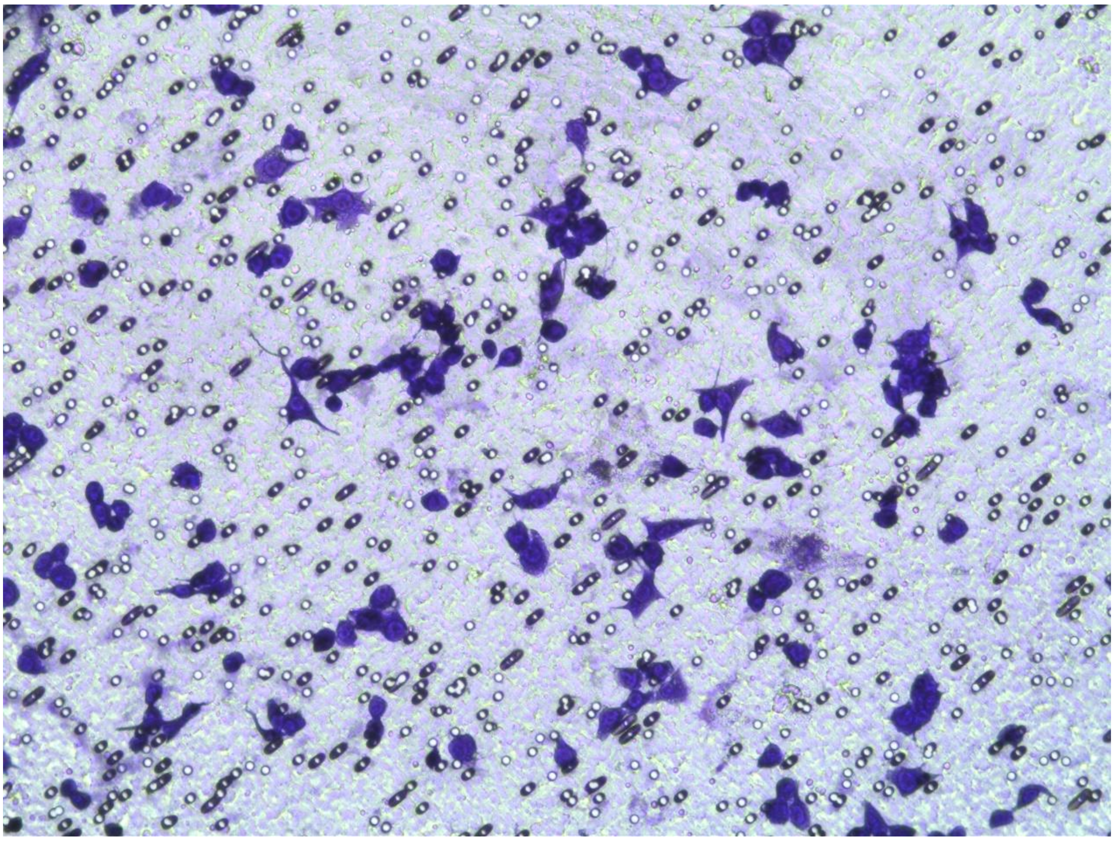

U20S NC 200-1

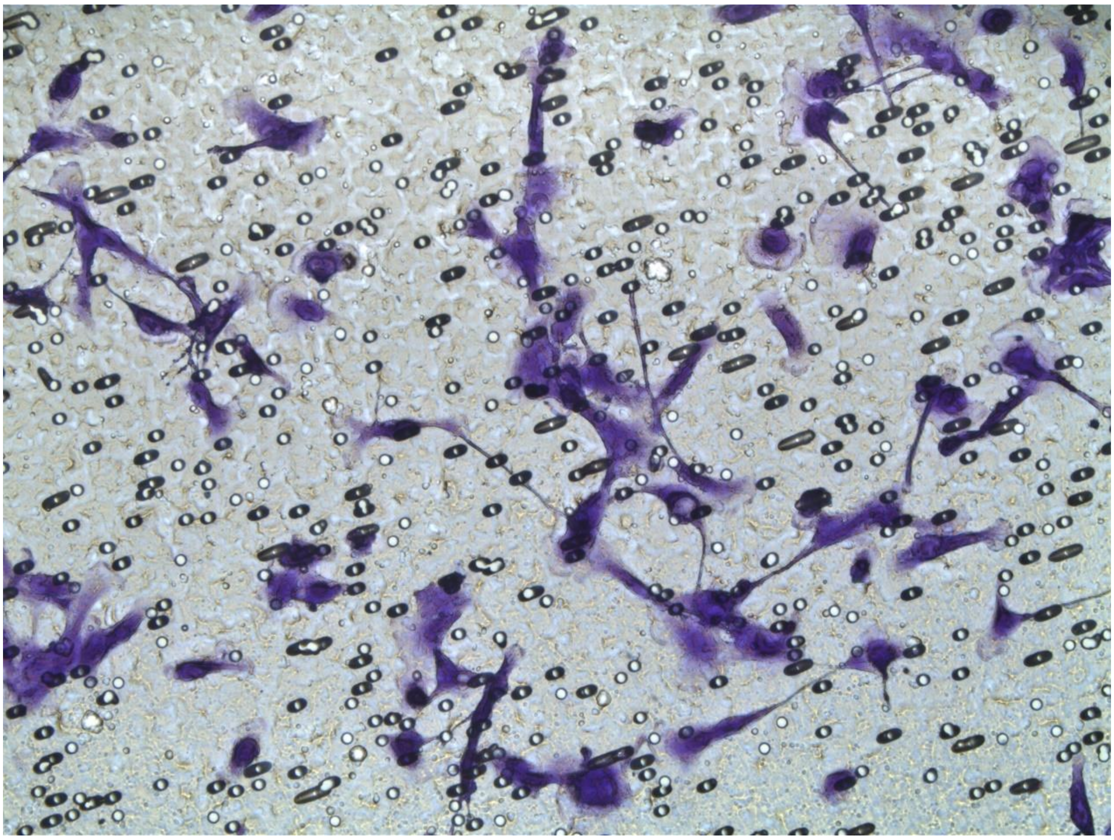

U20S NC 200-2

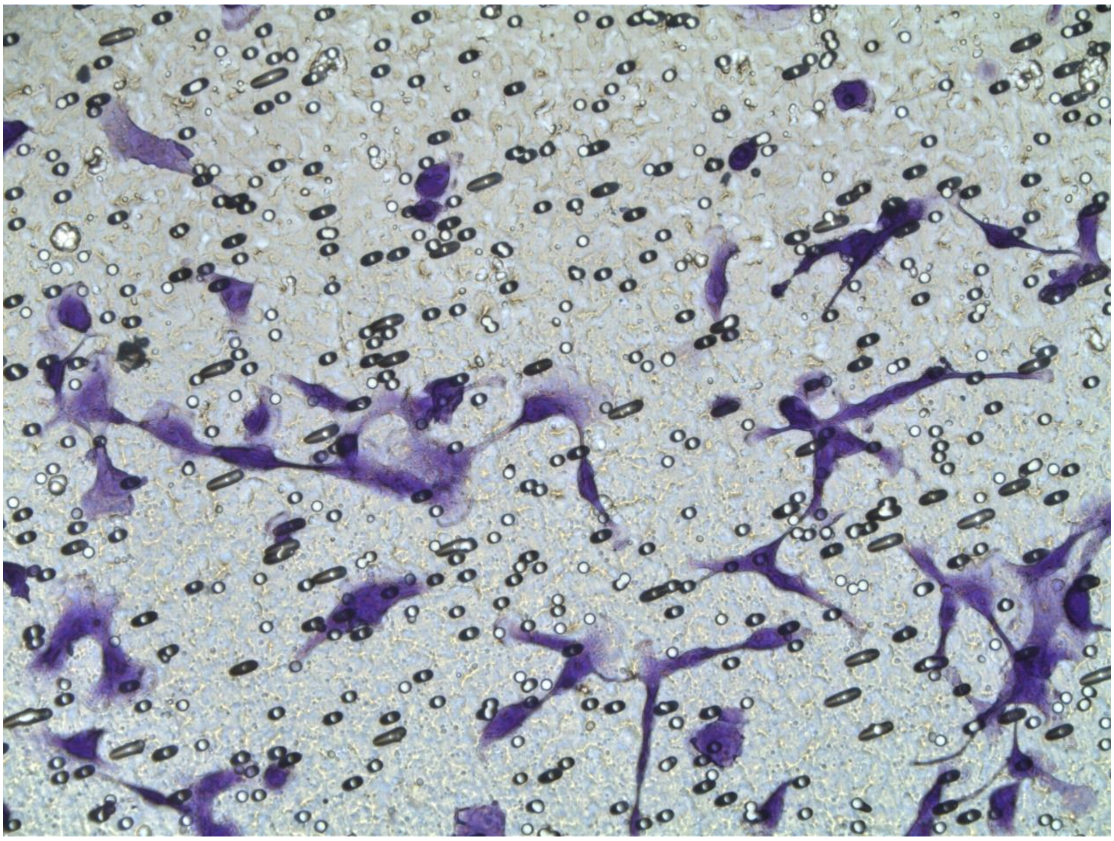

U20S NC 200-3

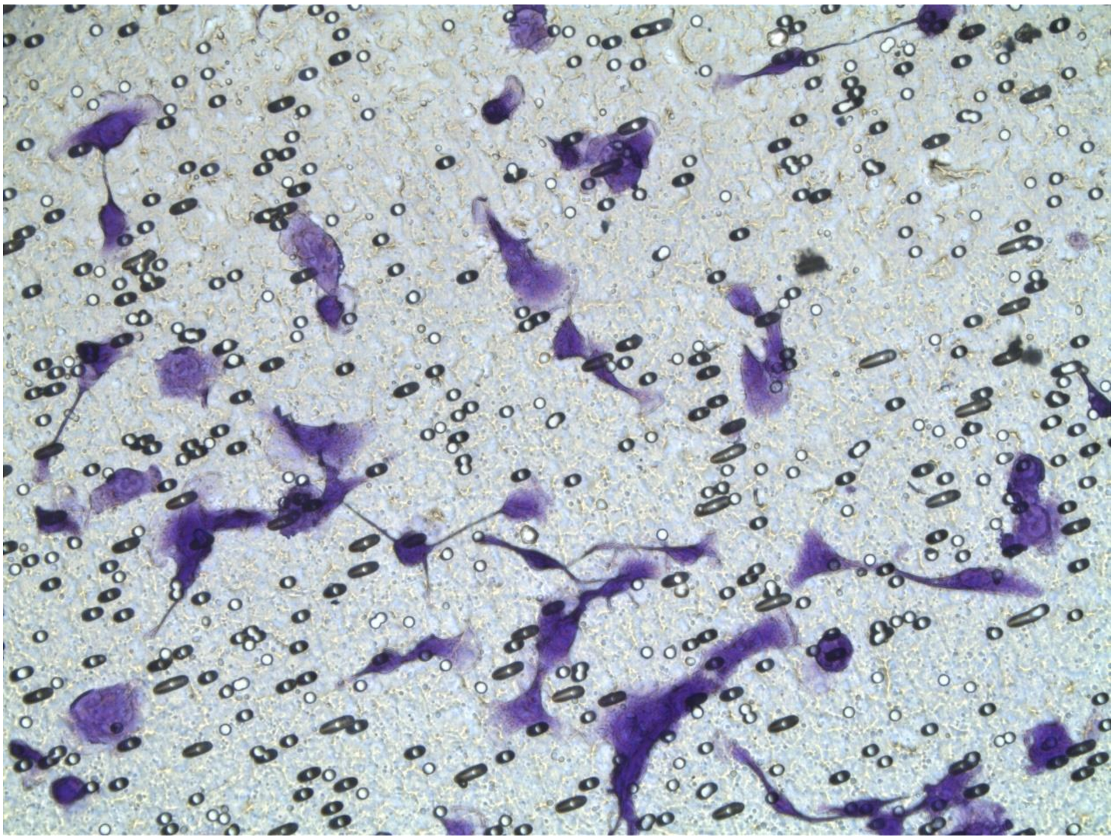

U20S NC 200-4

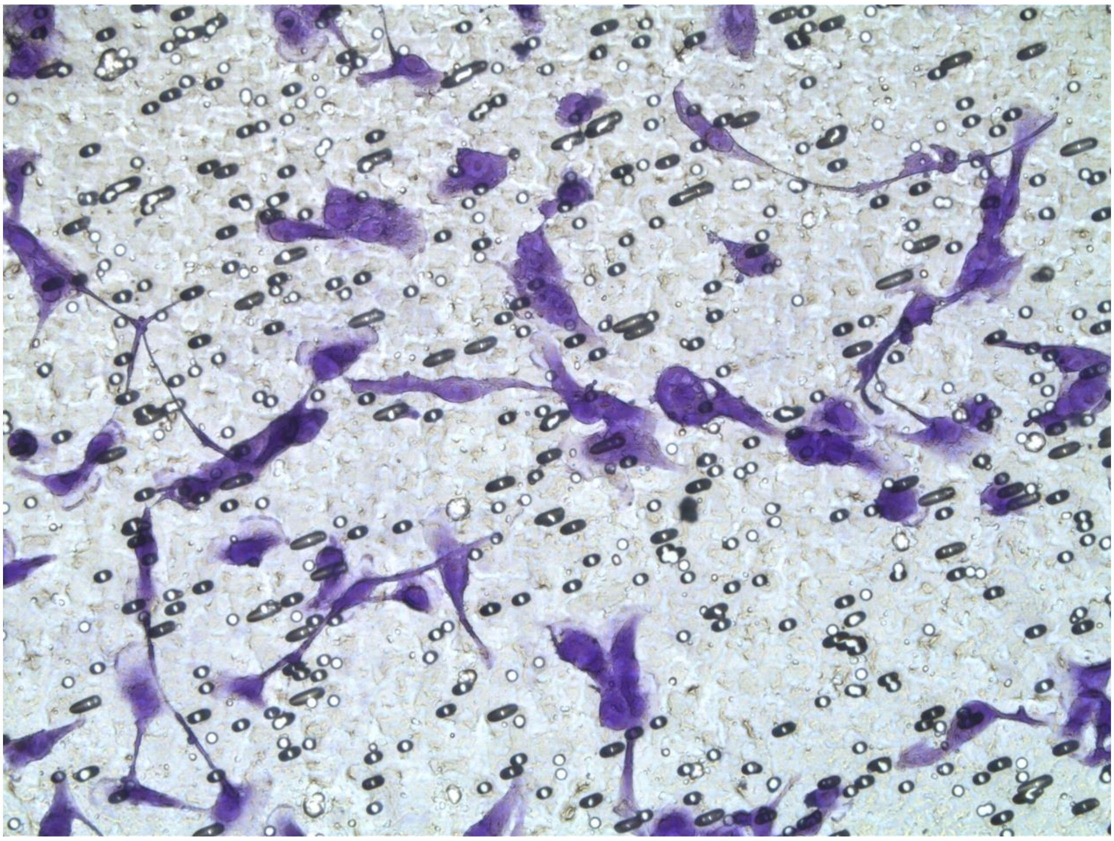

U20S NC 200-5

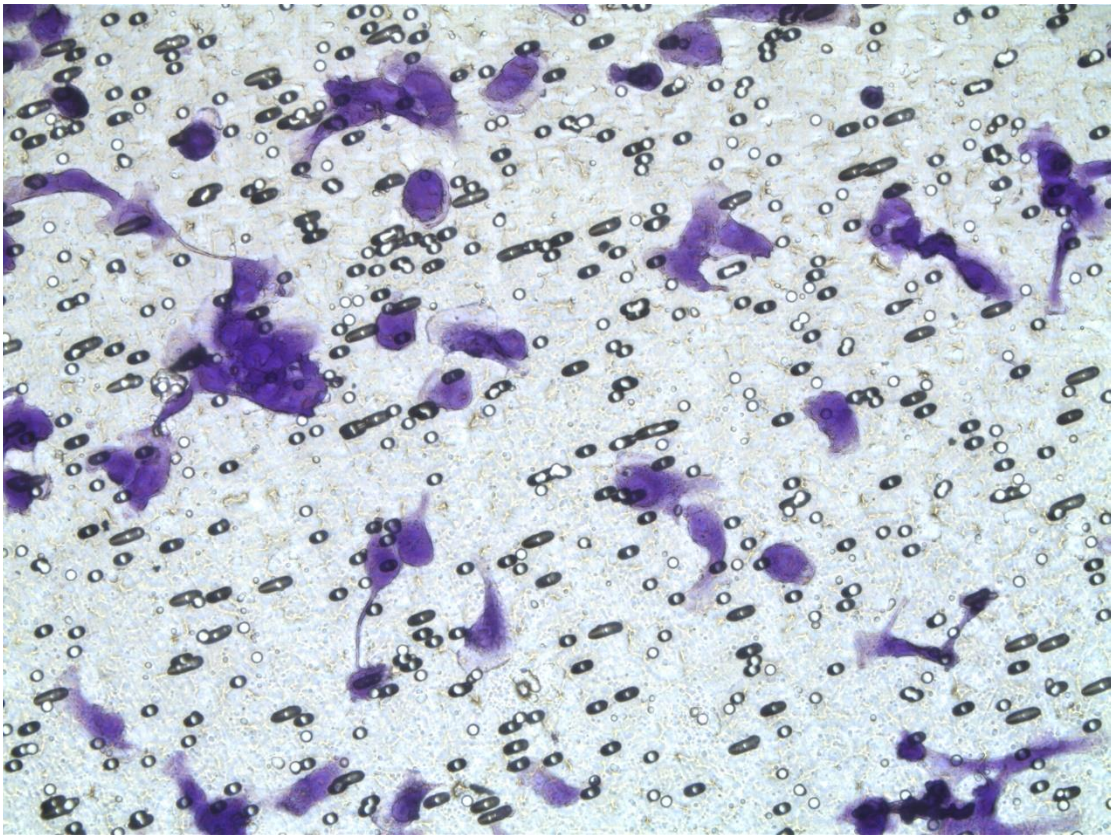

u20s oe 200-1

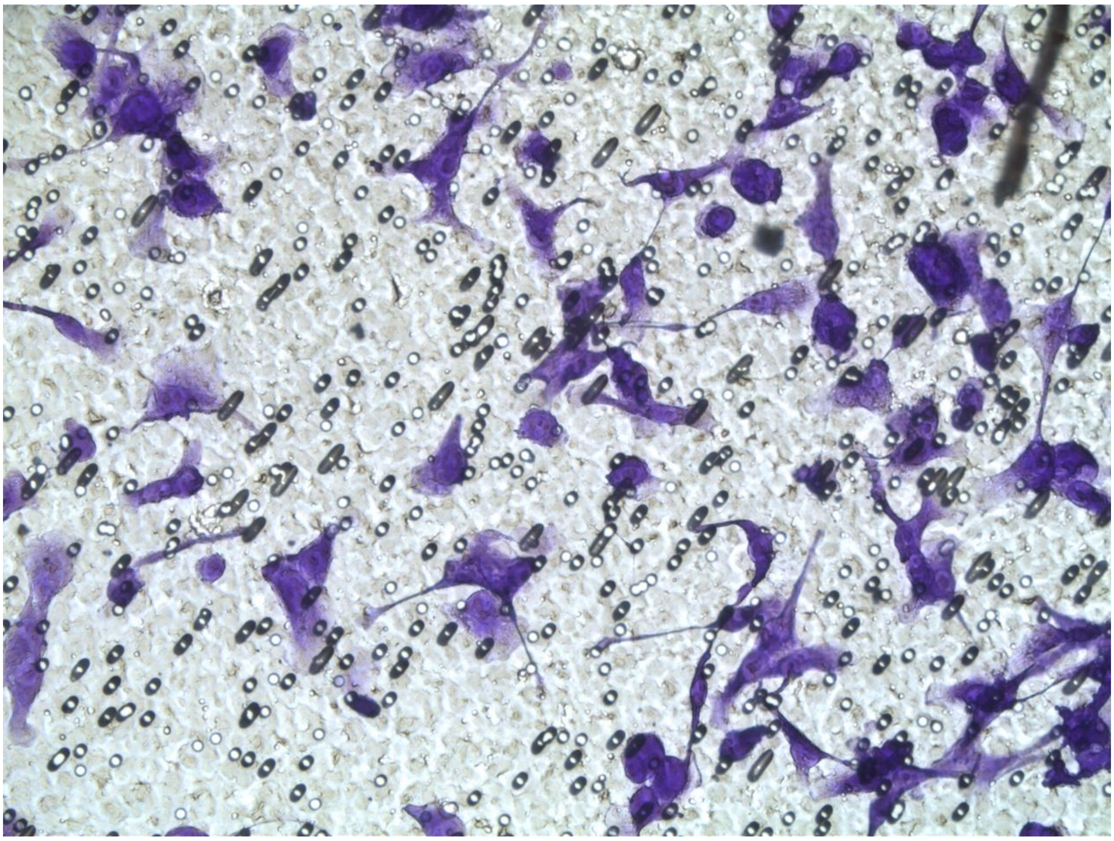

u20s oe 200-2

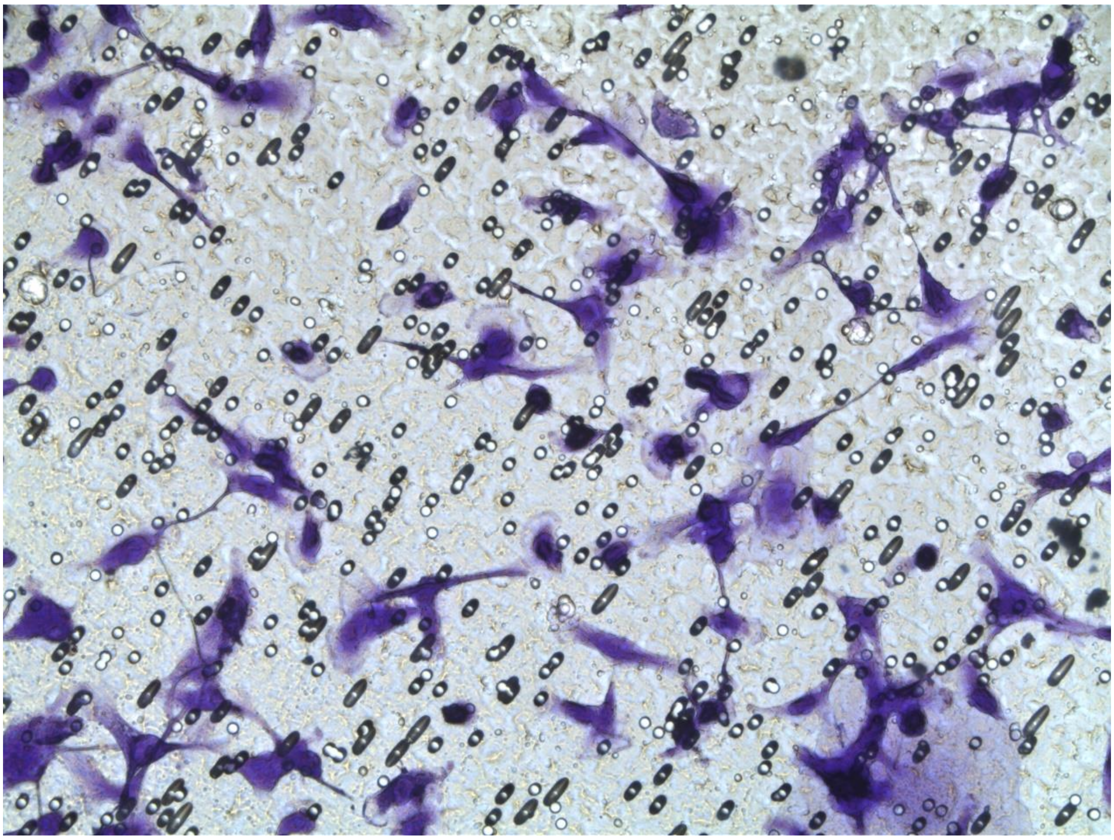

u20s oe 200-3

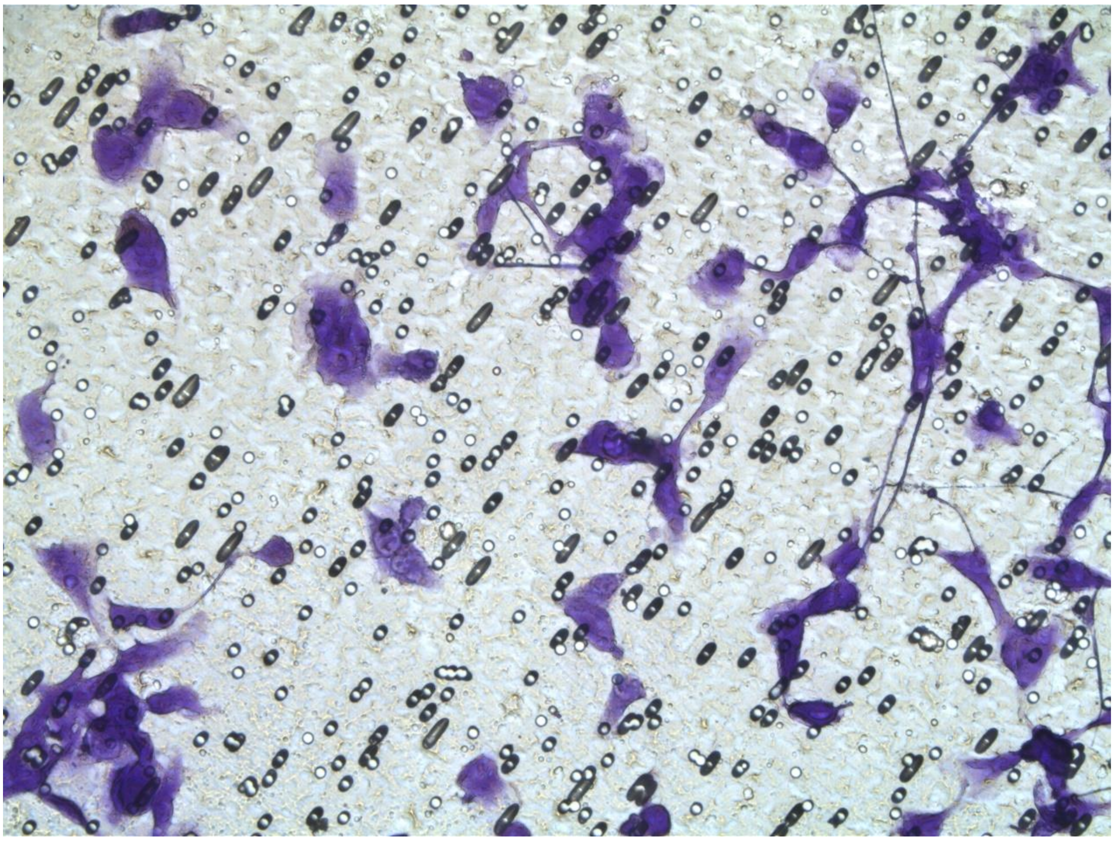

u20s oe 200-4

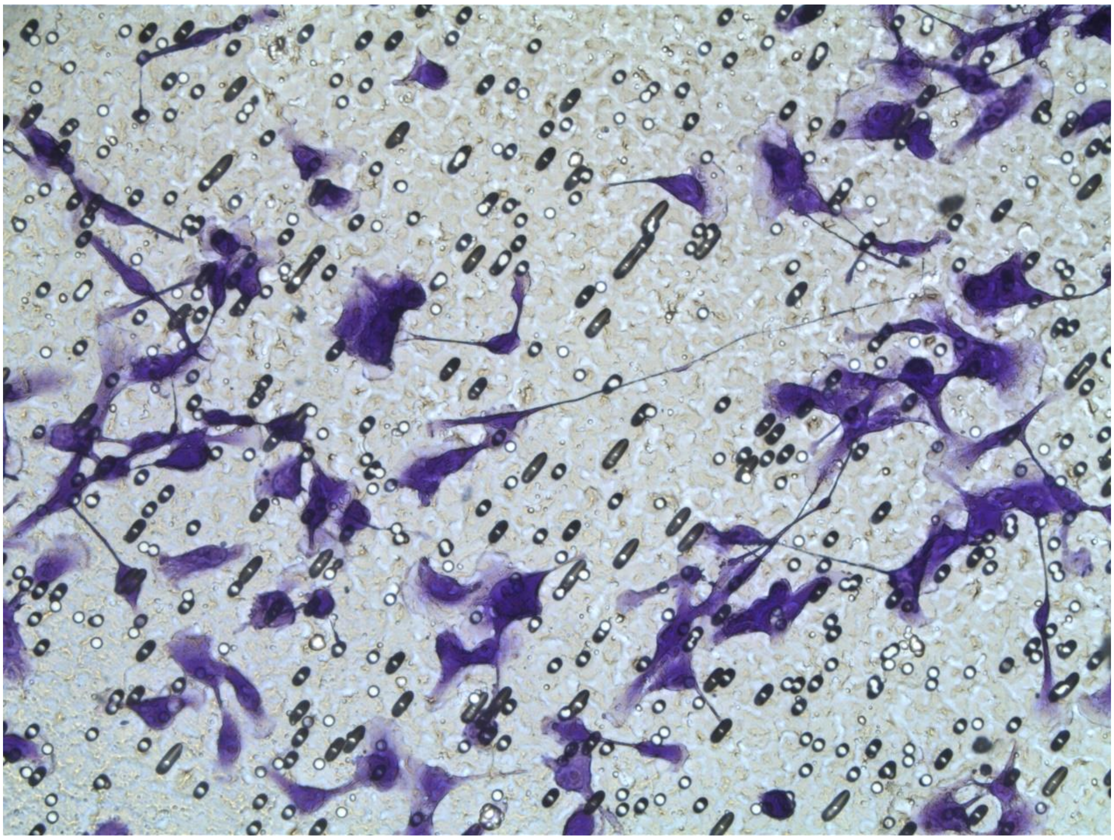

u20s oe 200-5

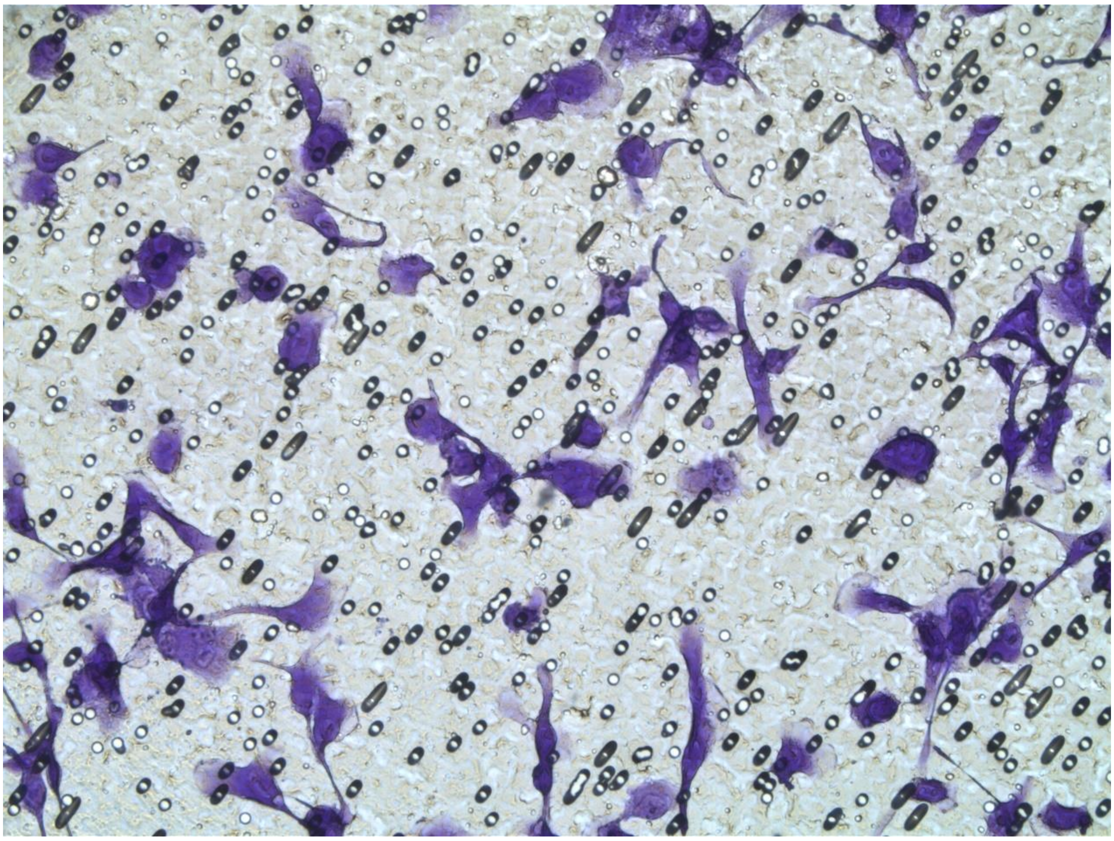

u20s oe+DMSO 200-1

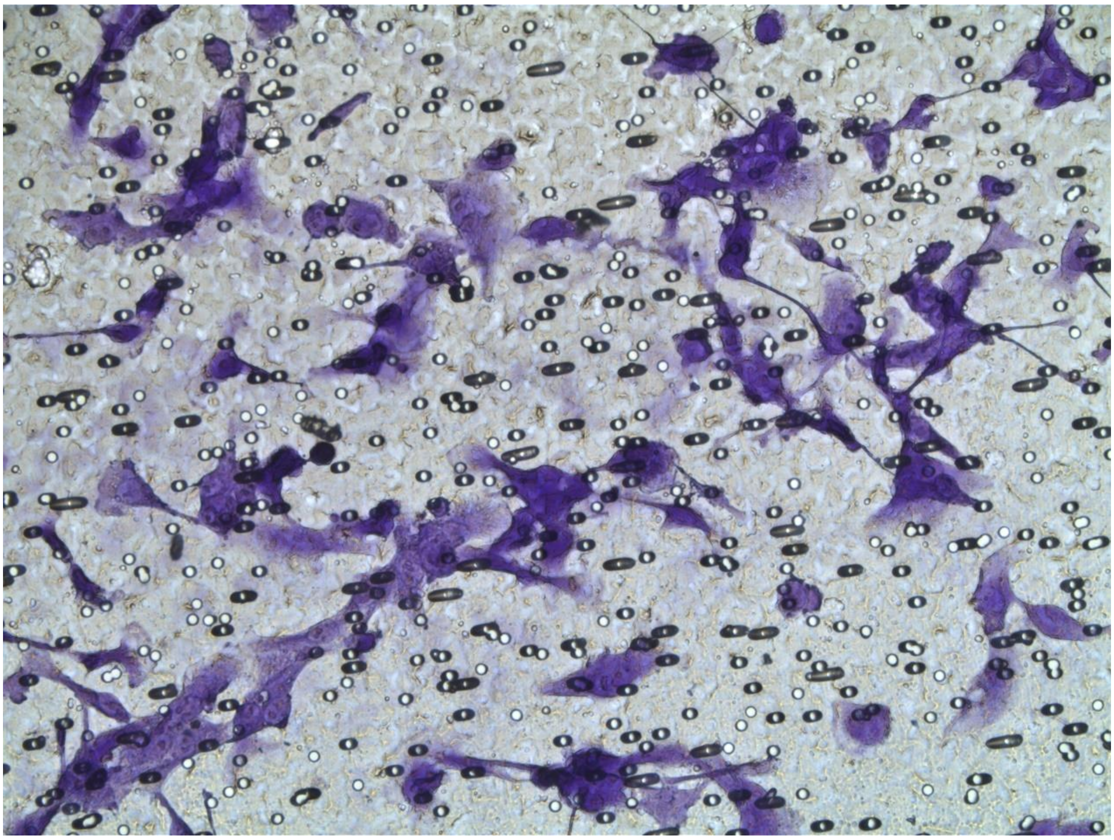

u20s oe+DMSO 200-2

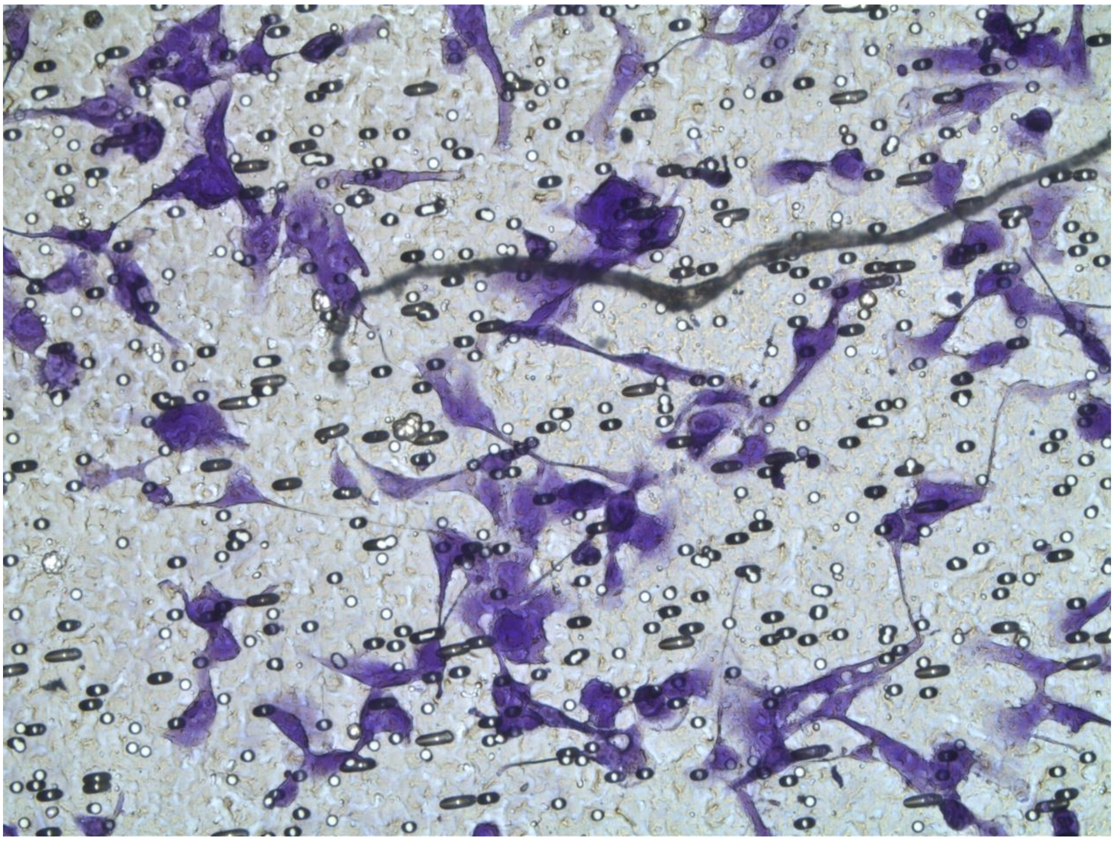

u20s oe+DMSO 200-3

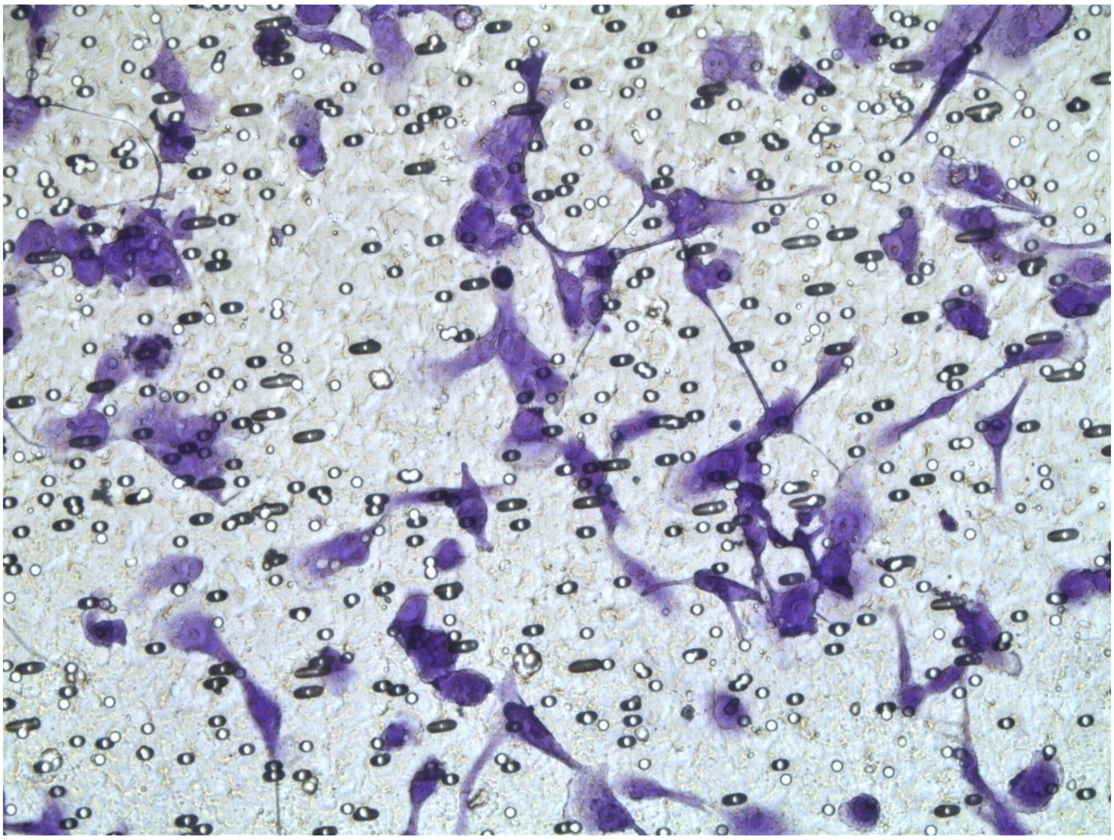

u20s oe+DMSO 200-4

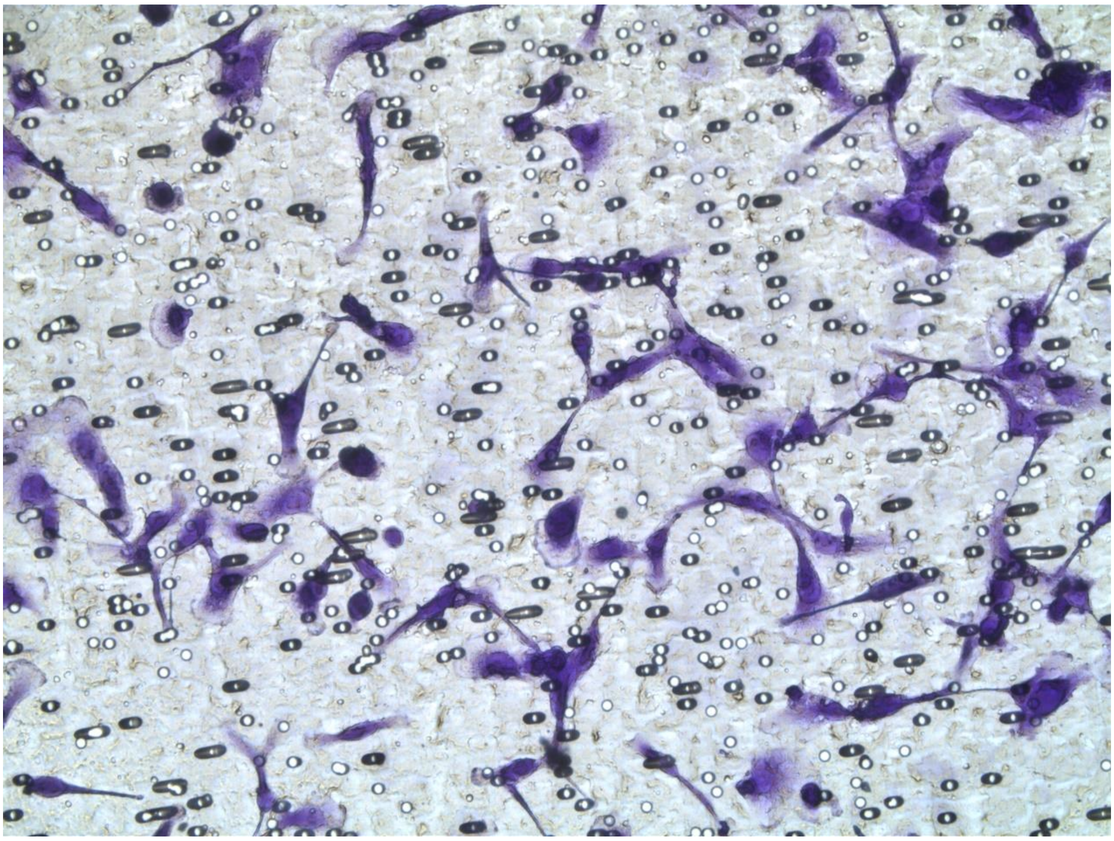

u20s oe+DMSO 200-5

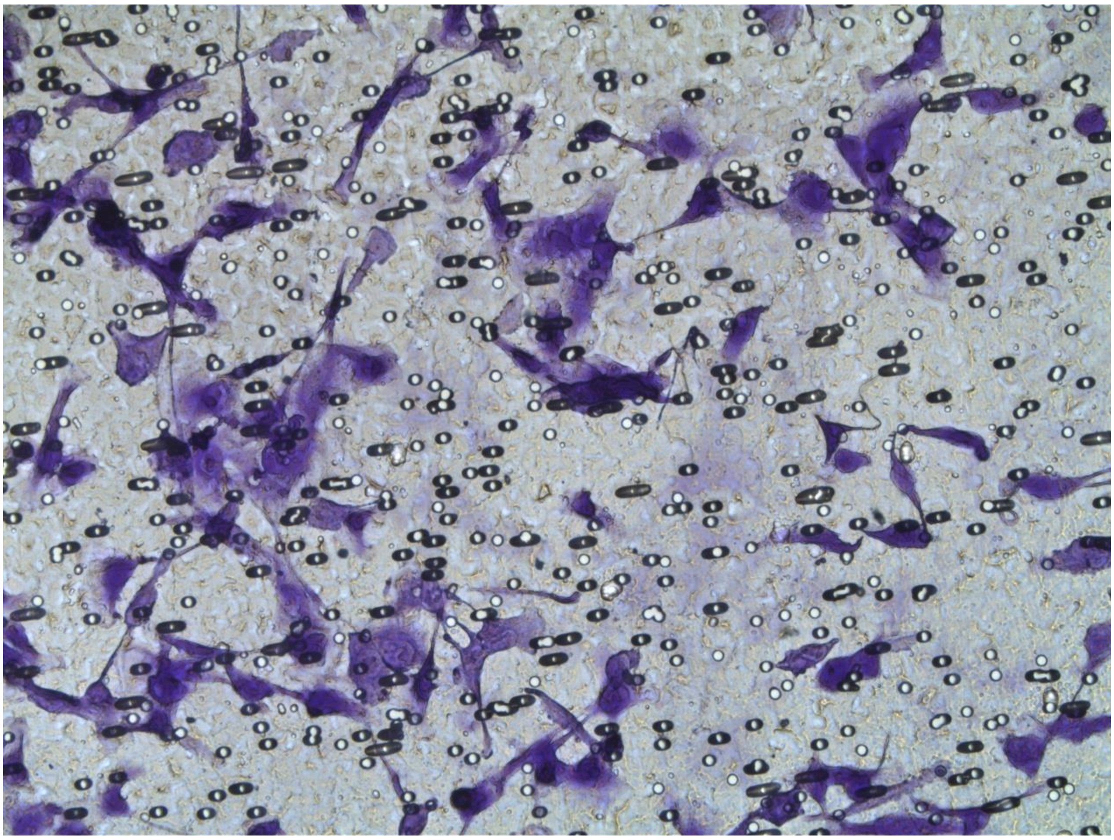

U20S OE+PI3K 200-1

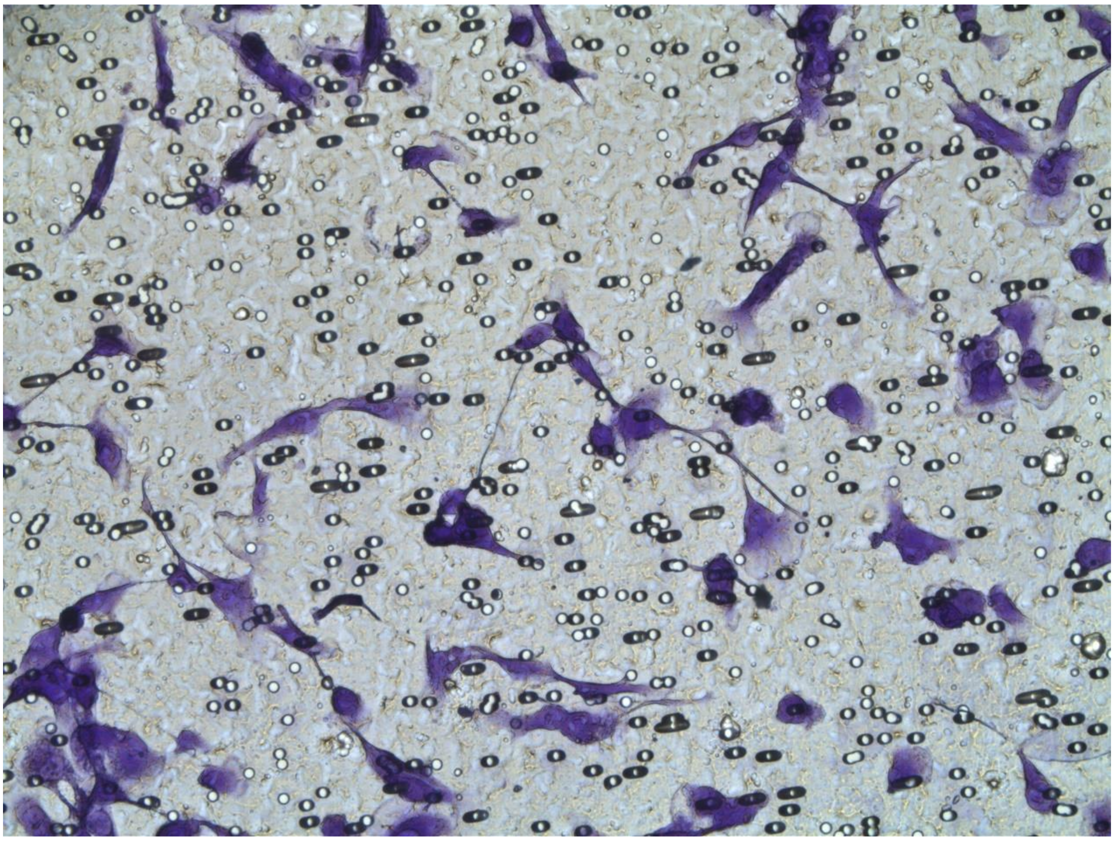

U20S OE+PI3K 200-2

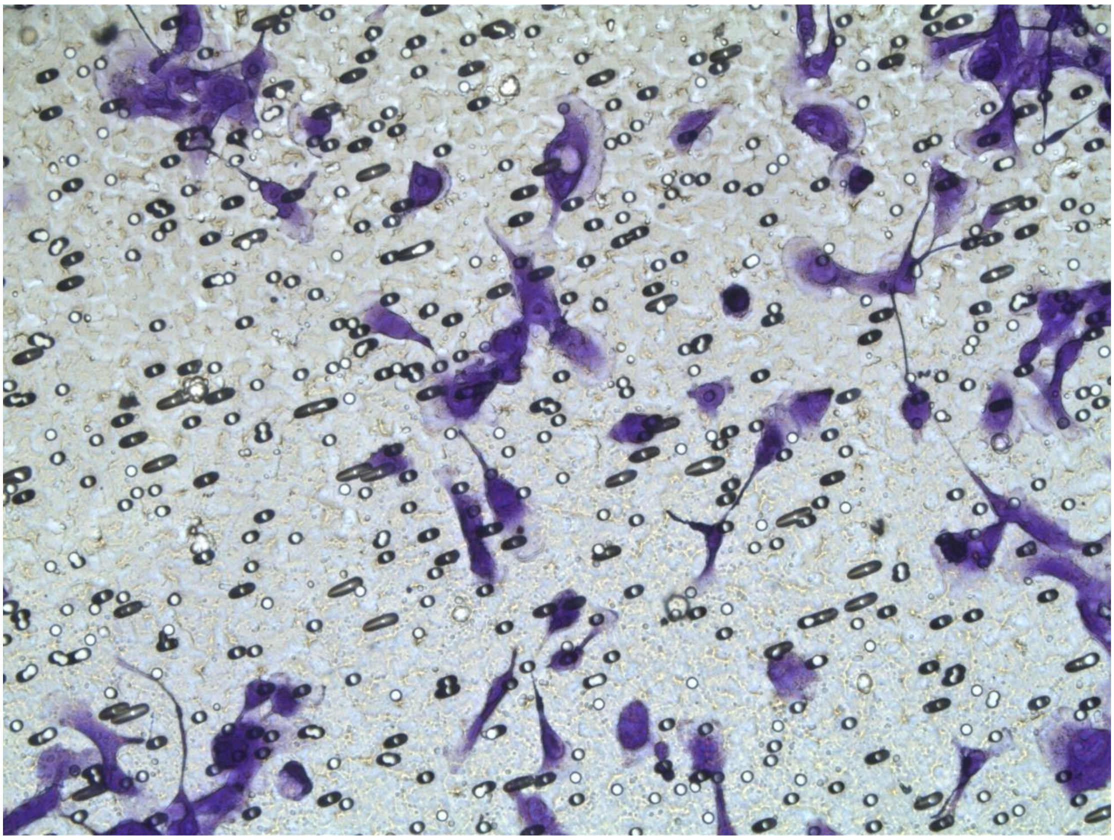

U20S OE+PI3K 200-3

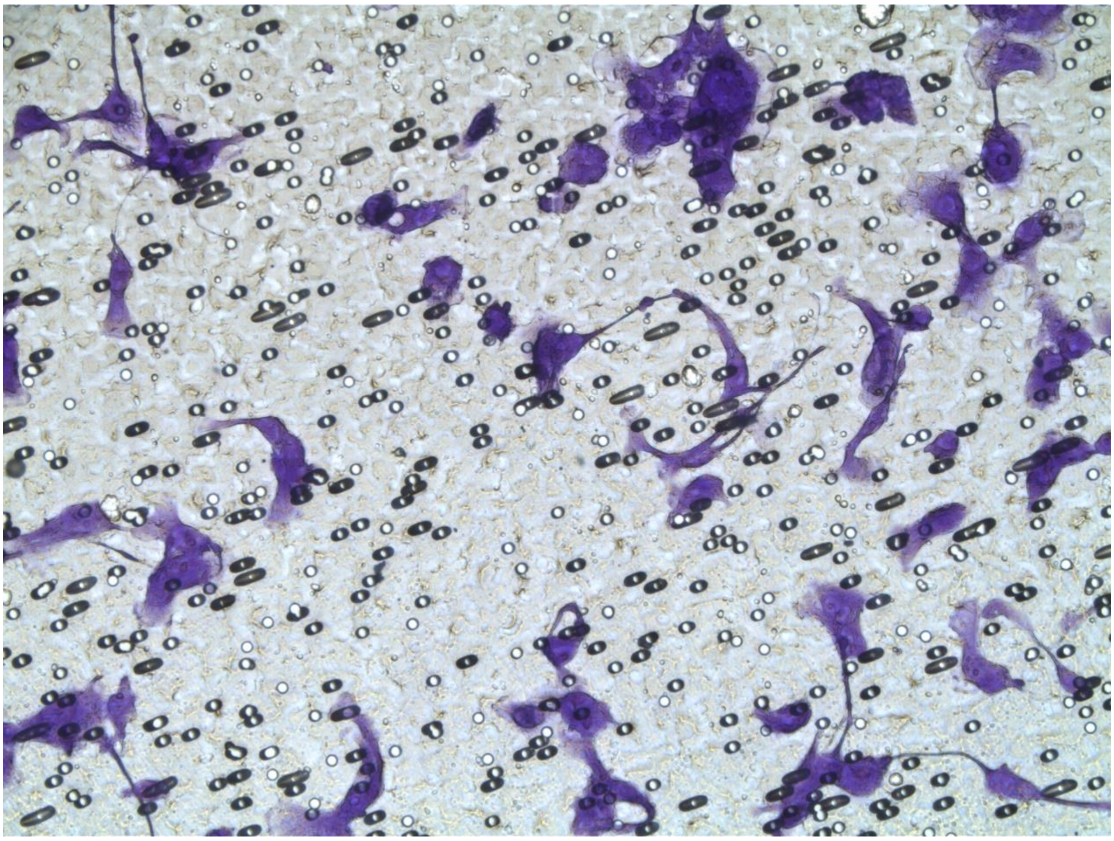

U20S OE+PI3K 200-4

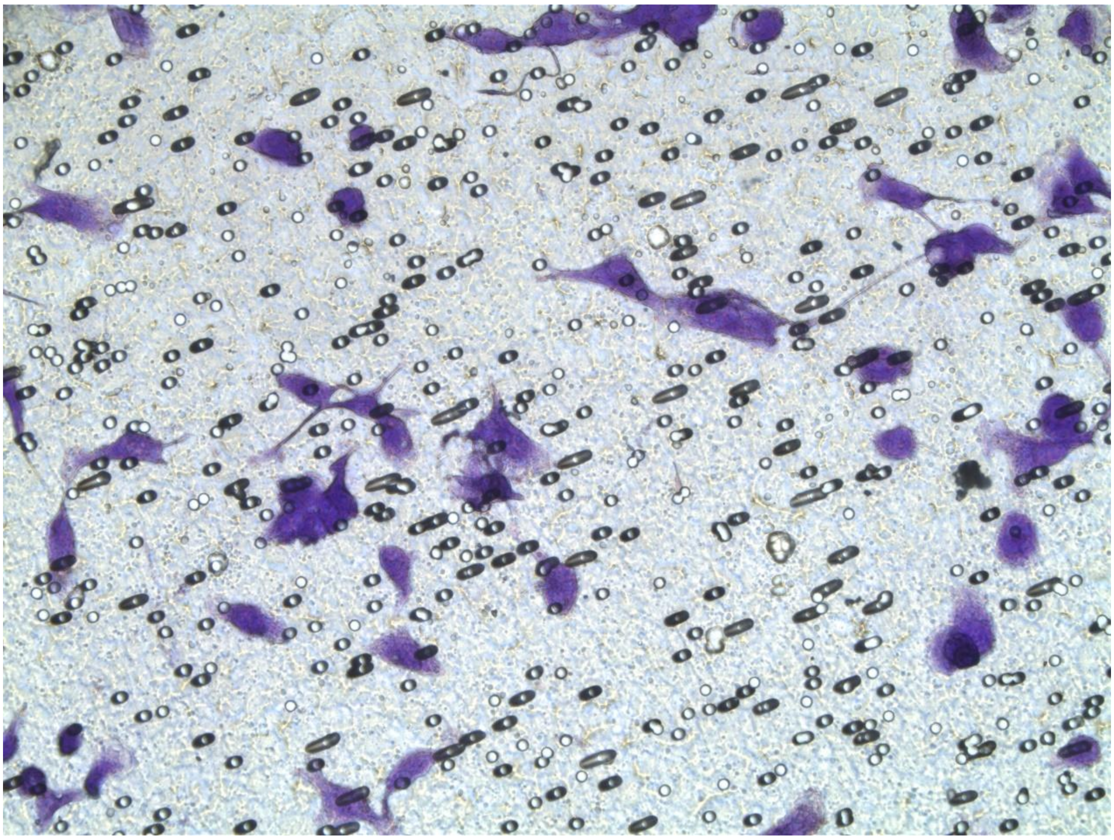

U20S OE+PI3K 200-5

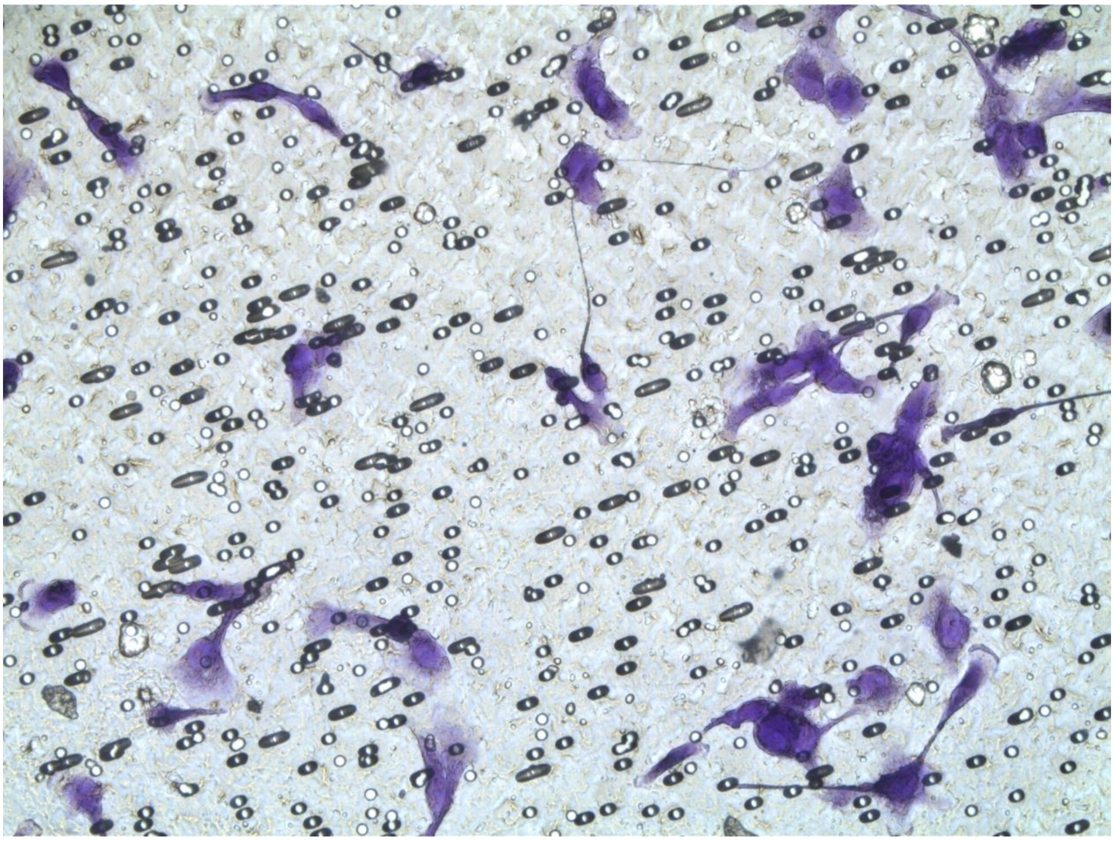

Fig 5 WB (MG63)

AKT

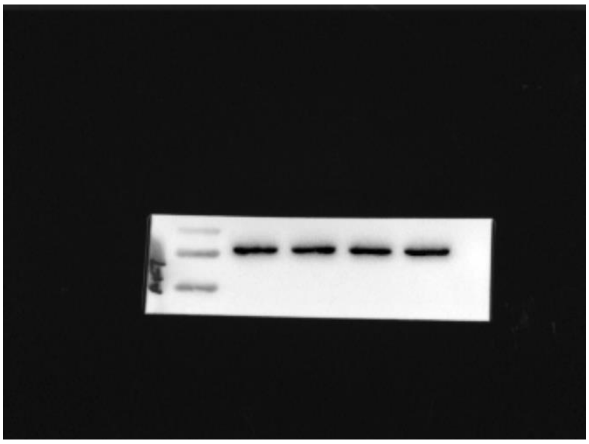

GAPDH

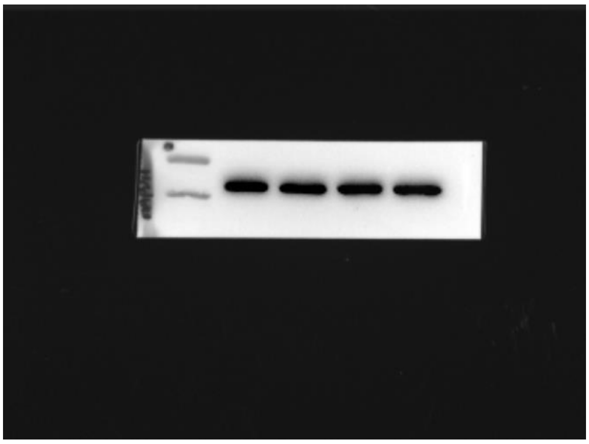

P-AKT

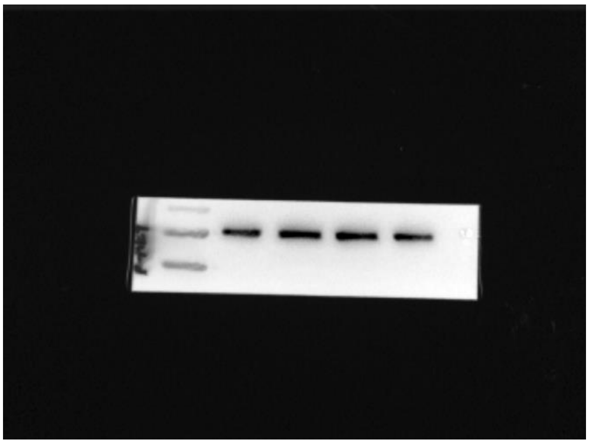

PI3K

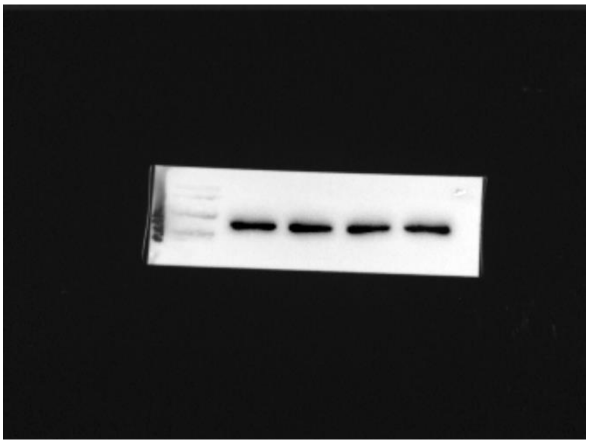

P-PI3K

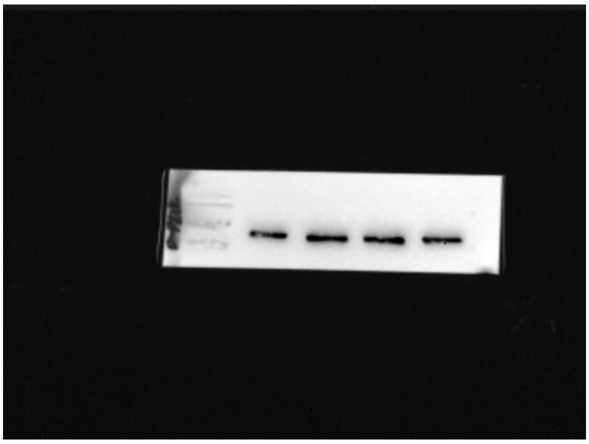

Fig 5 WB (U2OS)

AKT

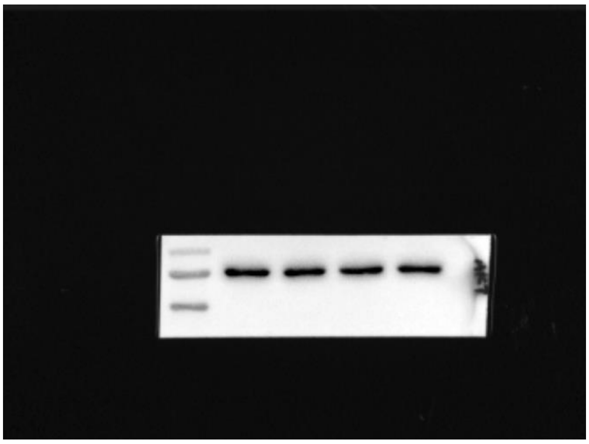

GAPDH

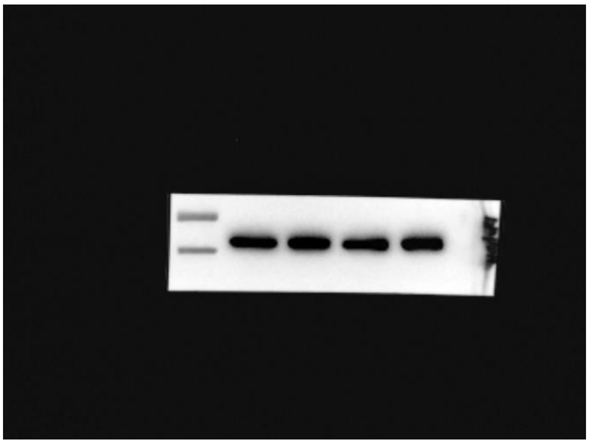

P-AKT

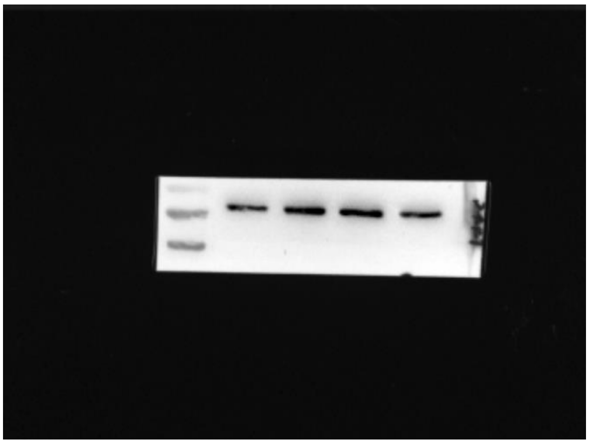

PI3K

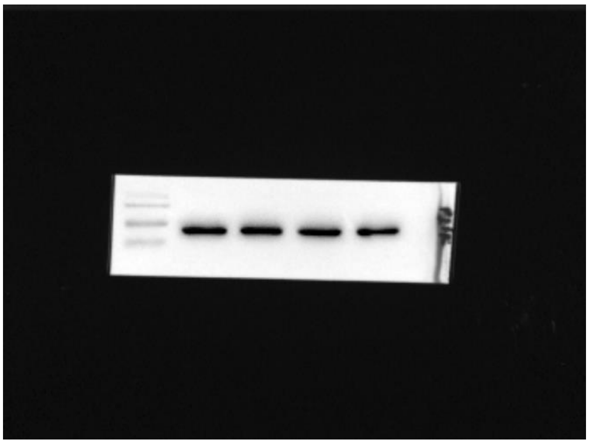

P-PI3K

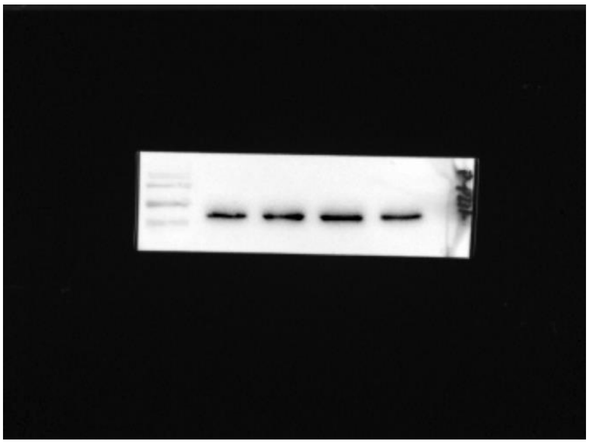

Fig 6D WB

ANXA2

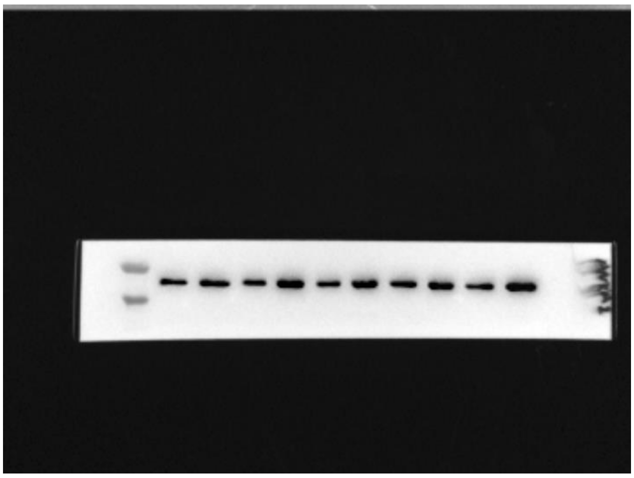

GAPDH

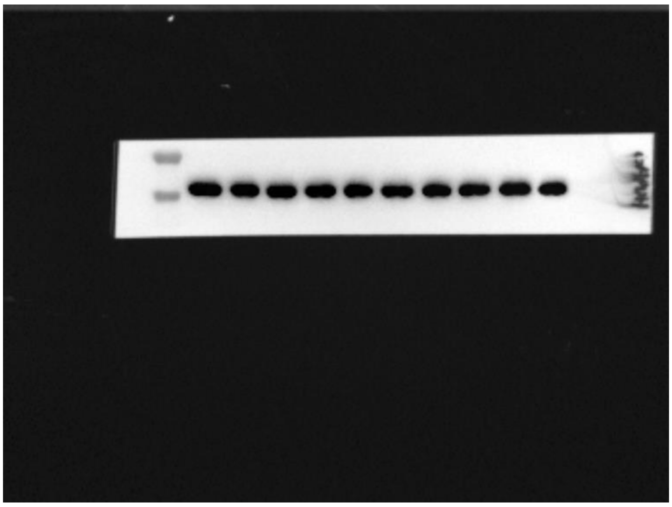

Fig 6F WB

ANXA2

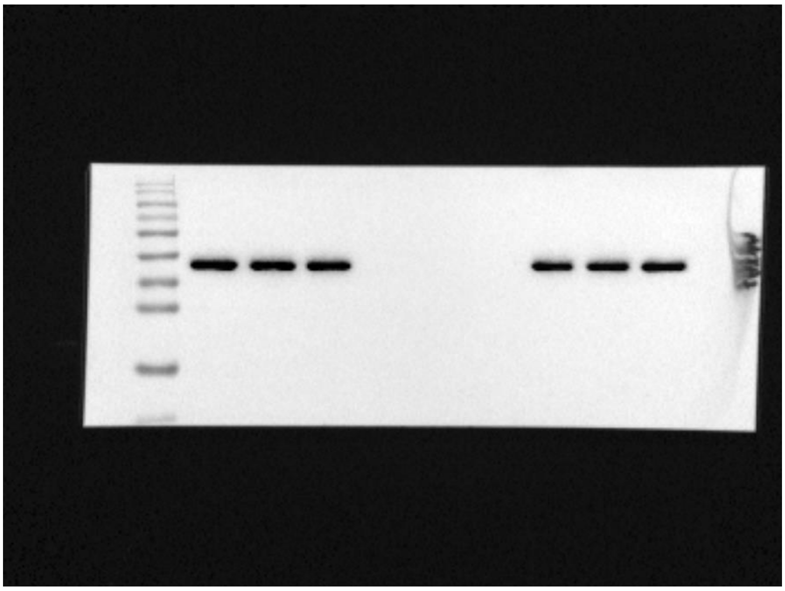

S100A16

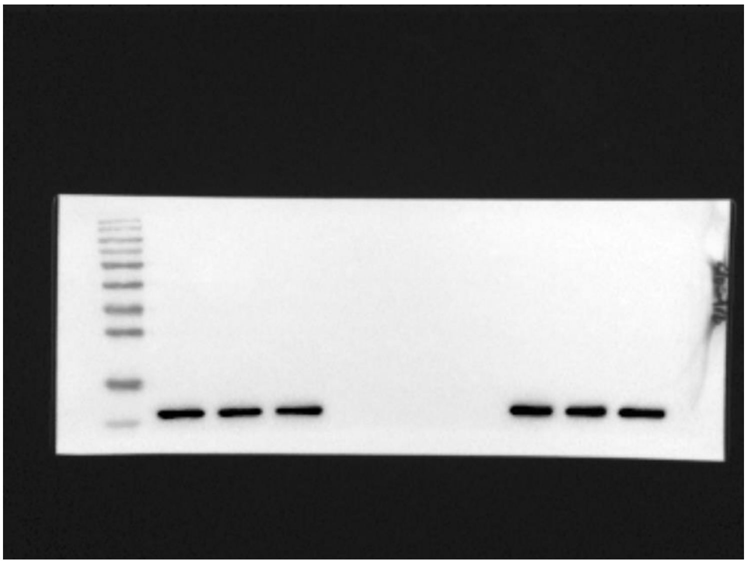

Fig 6G WB

MG63 ANXA2 (OE S100A16)

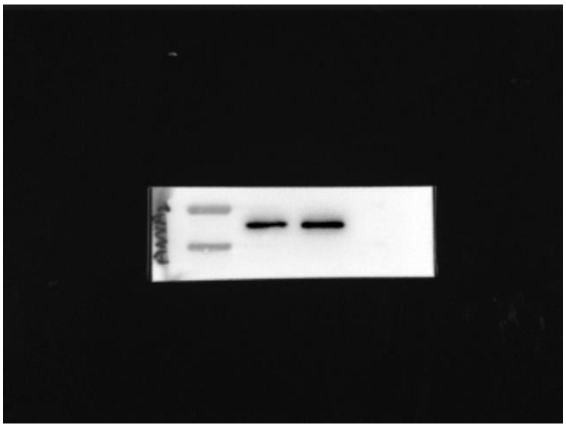

MG63 GAPDH (OE S100A16)

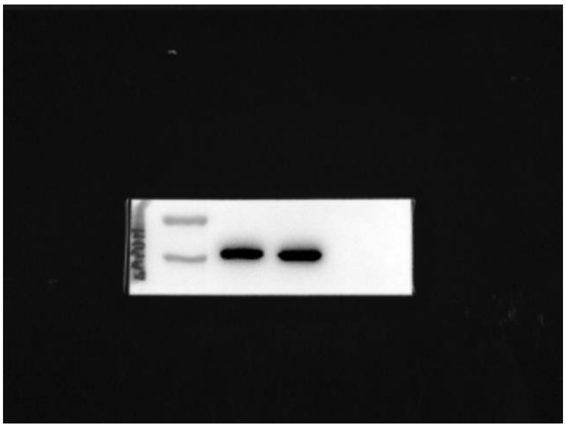

U2OS ANXA2 (OE S100A16)

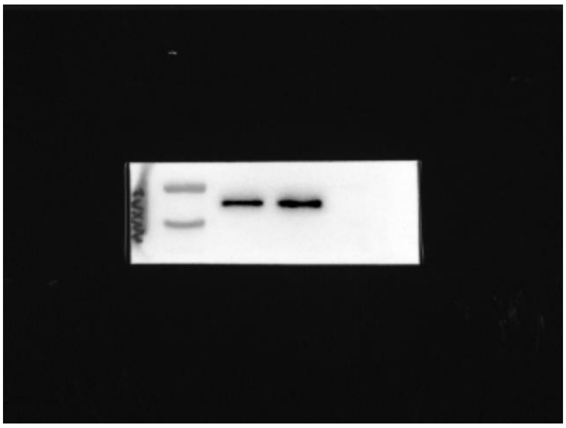

U2OS GAPDH (OE S100A16)

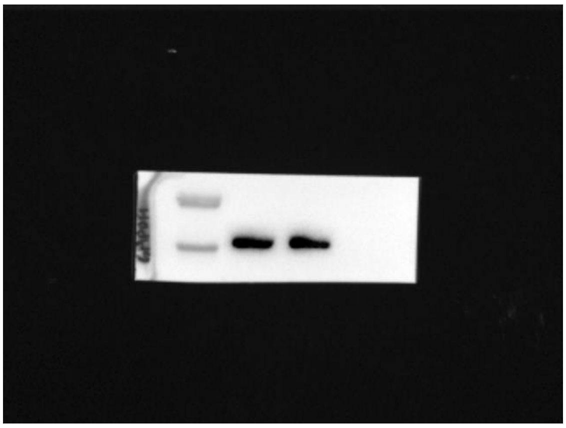

MG63 ANXA2 (Sh S100A16)

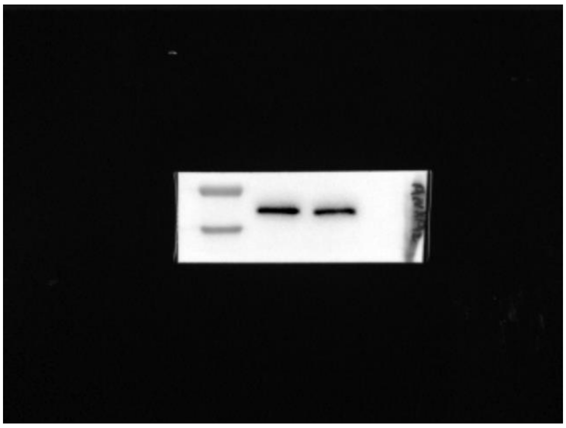

MG63 GAPDH (Sh S100A16)

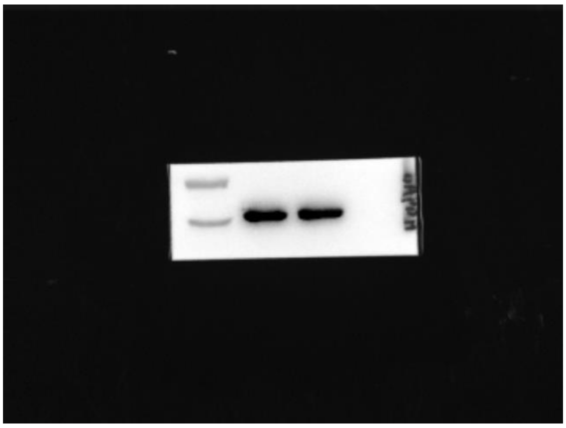

U2OS ANXA2 (Sh S100A16)

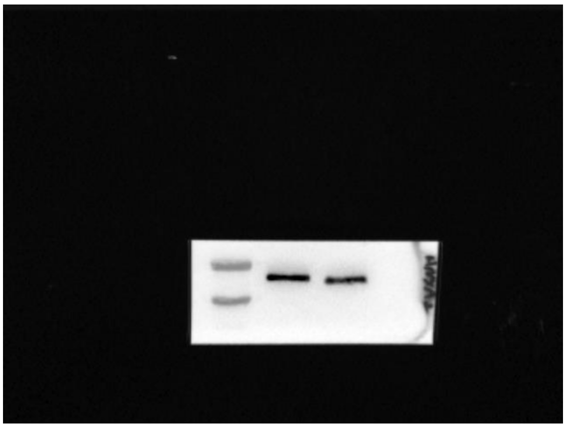

U2OS GAPDH (Sh S100A16)

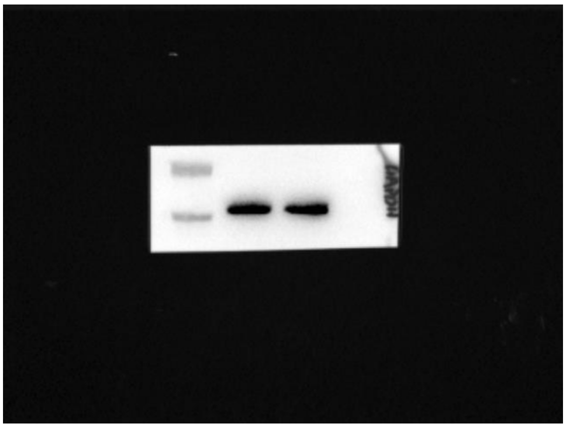

Fig 7 WB (MG-63)
AKT

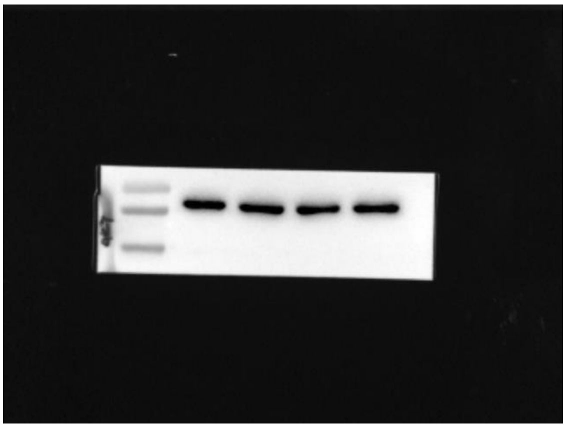

GAPDH

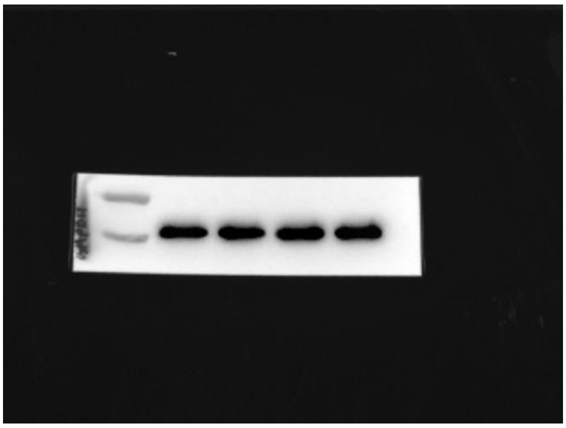

P-AKT

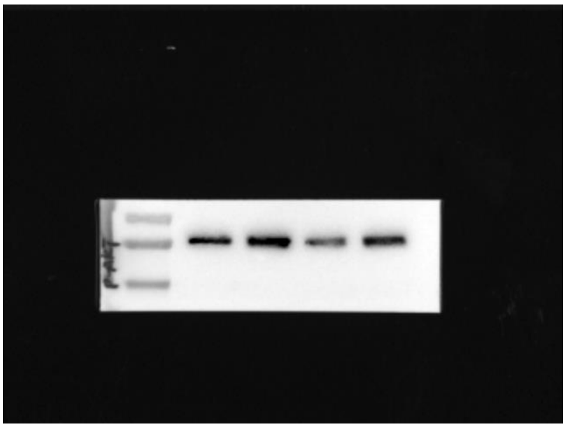

PI3K

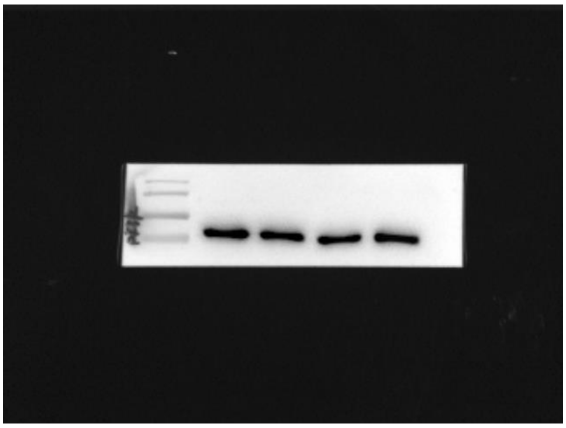

P-PI3K

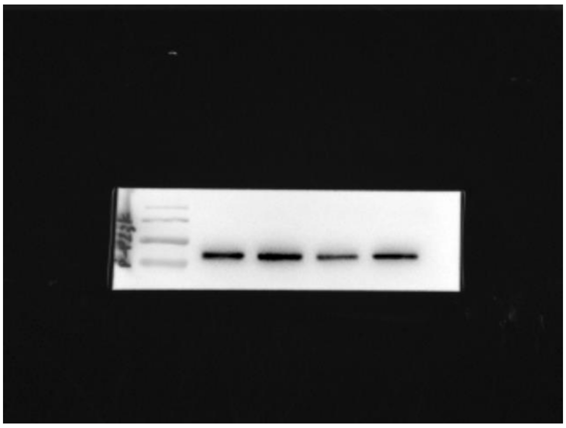

Fig 7 WB (U2OS)
AKT

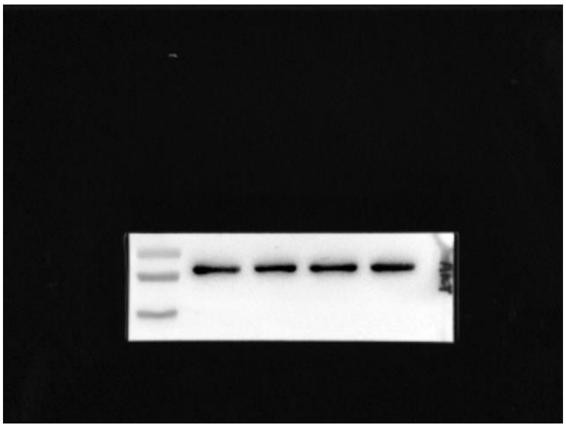

GAPDH

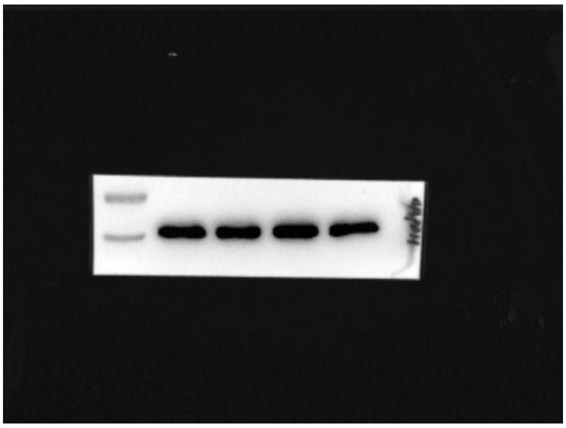

P-AKT

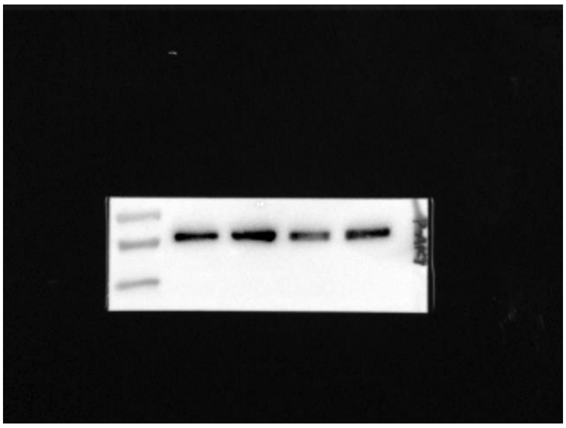

PI3K

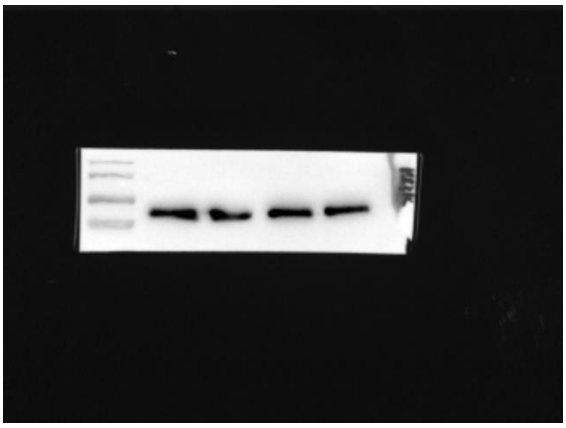

P-PI3K

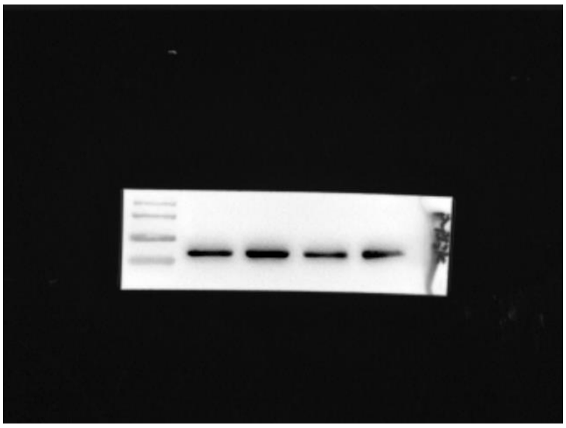

Fig 7 Transwell

MG63 shNC+OEANXA2 200-1

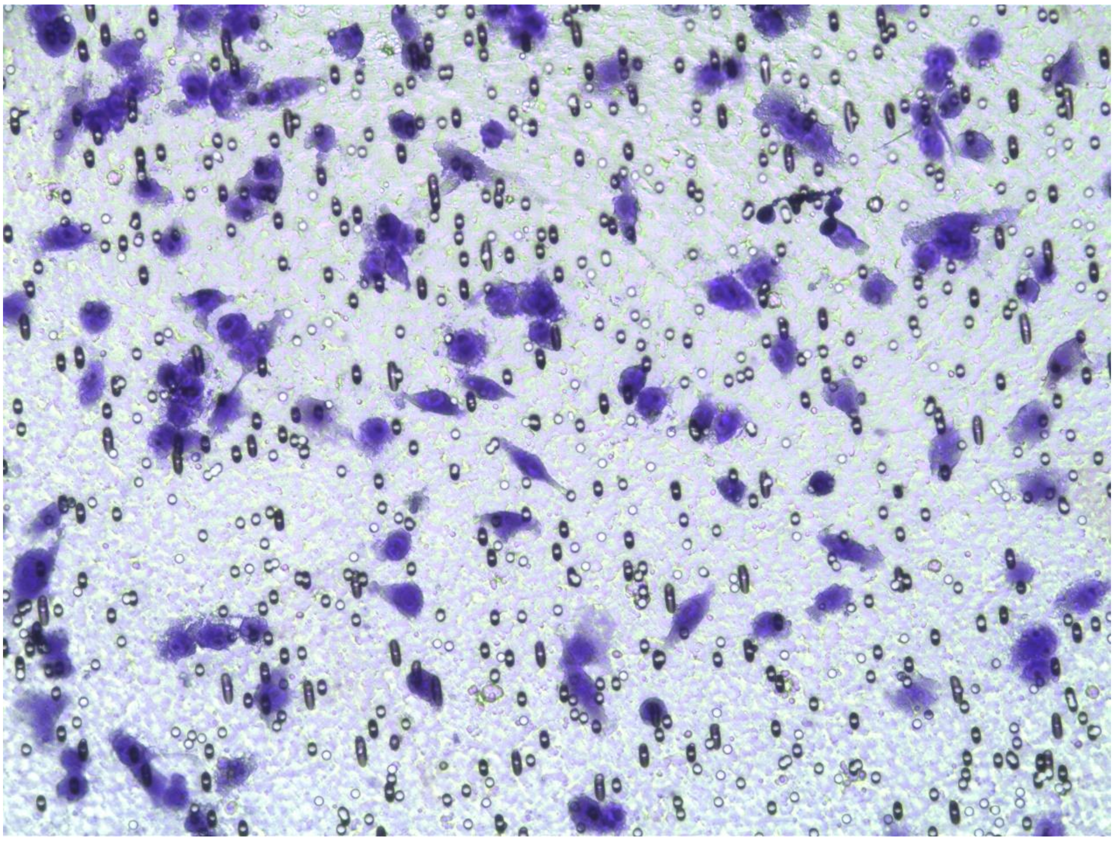

MG63 shNC+OEANXA2 200-2

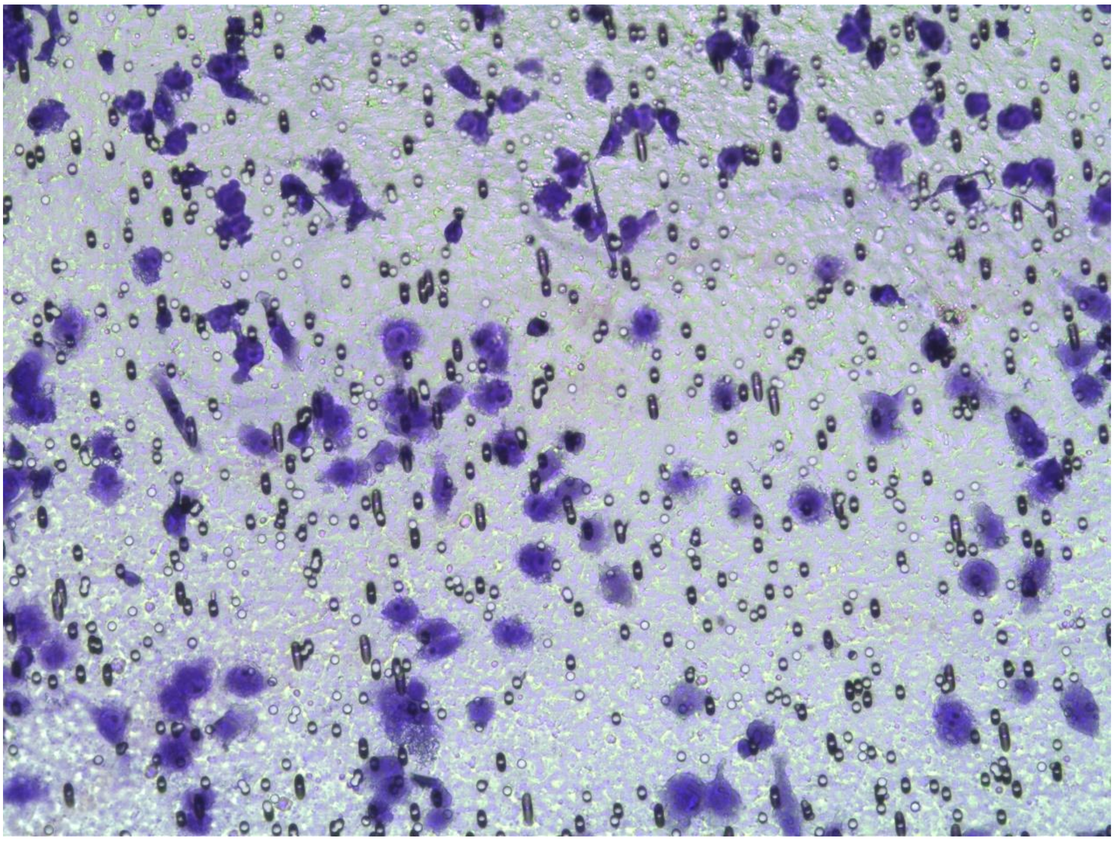

MG63 shNC+OEANXA2 200-3

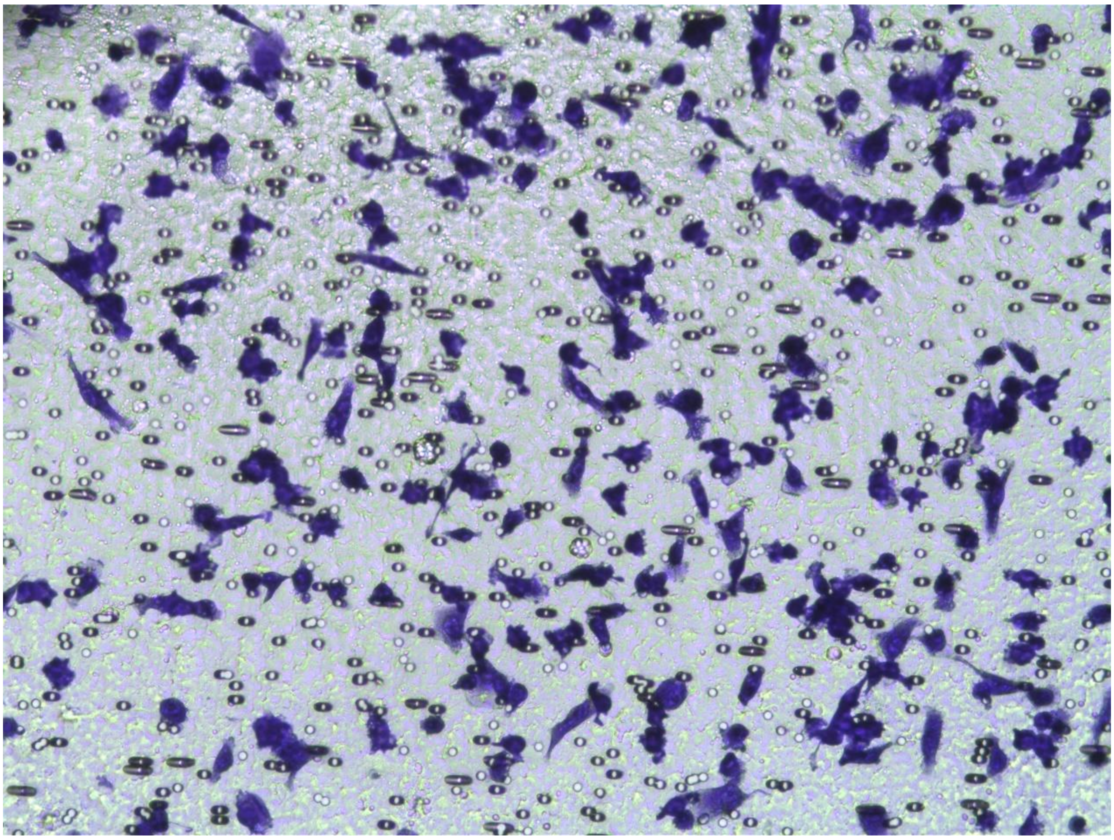

MG63 shNC+OEANXA2 200-4

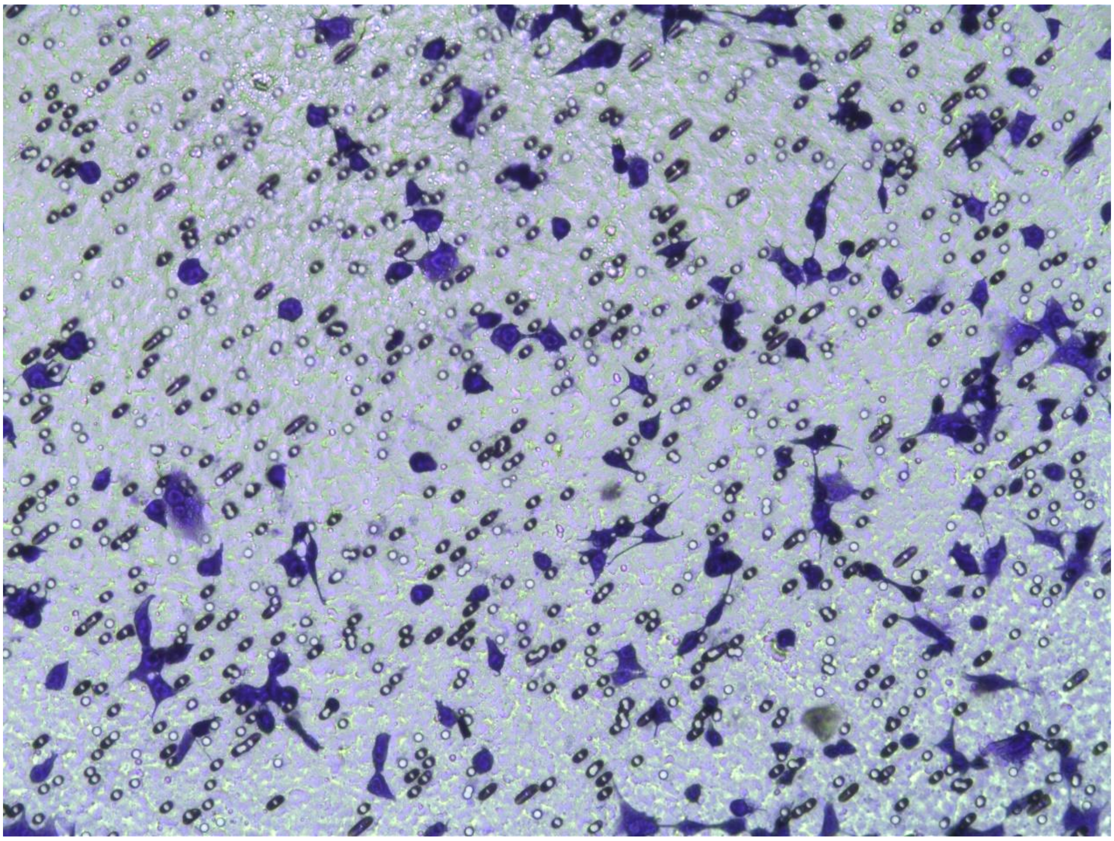

MG63 shNC+OEANXA2 200-5

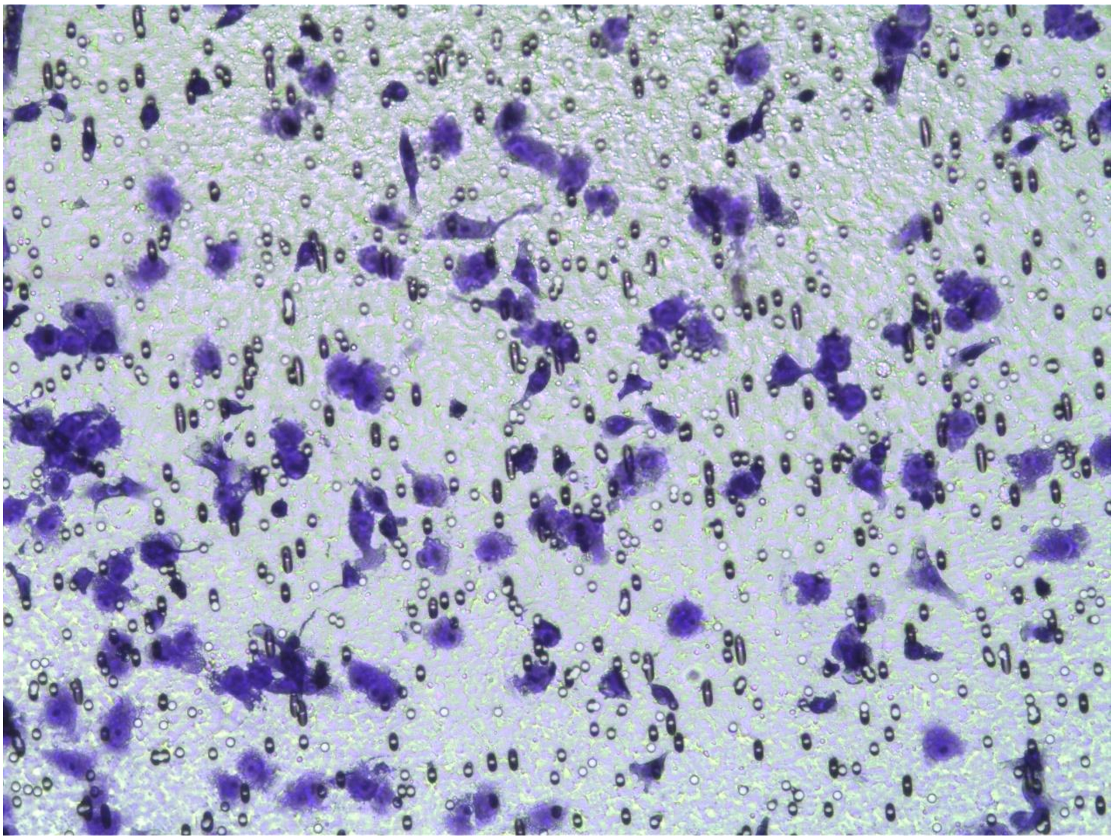

MG63 shNC+vector 200-1

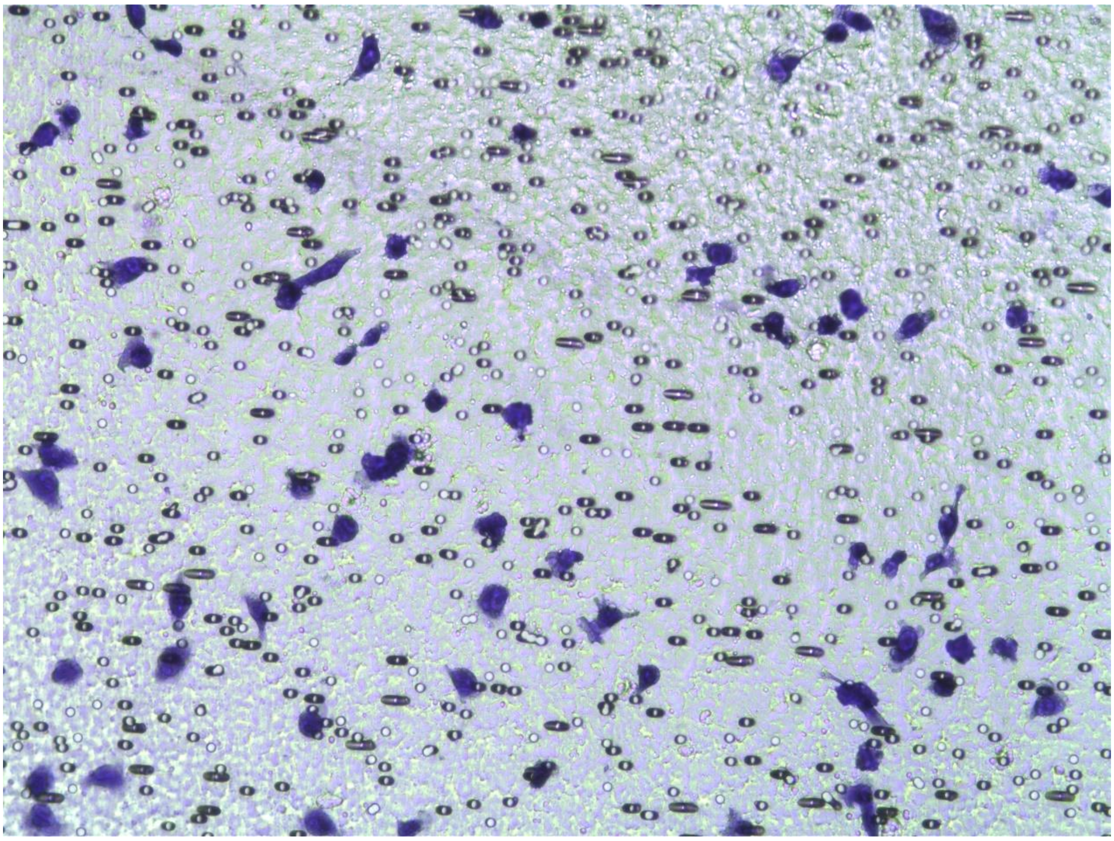

MG63 shNC+vector 200-2

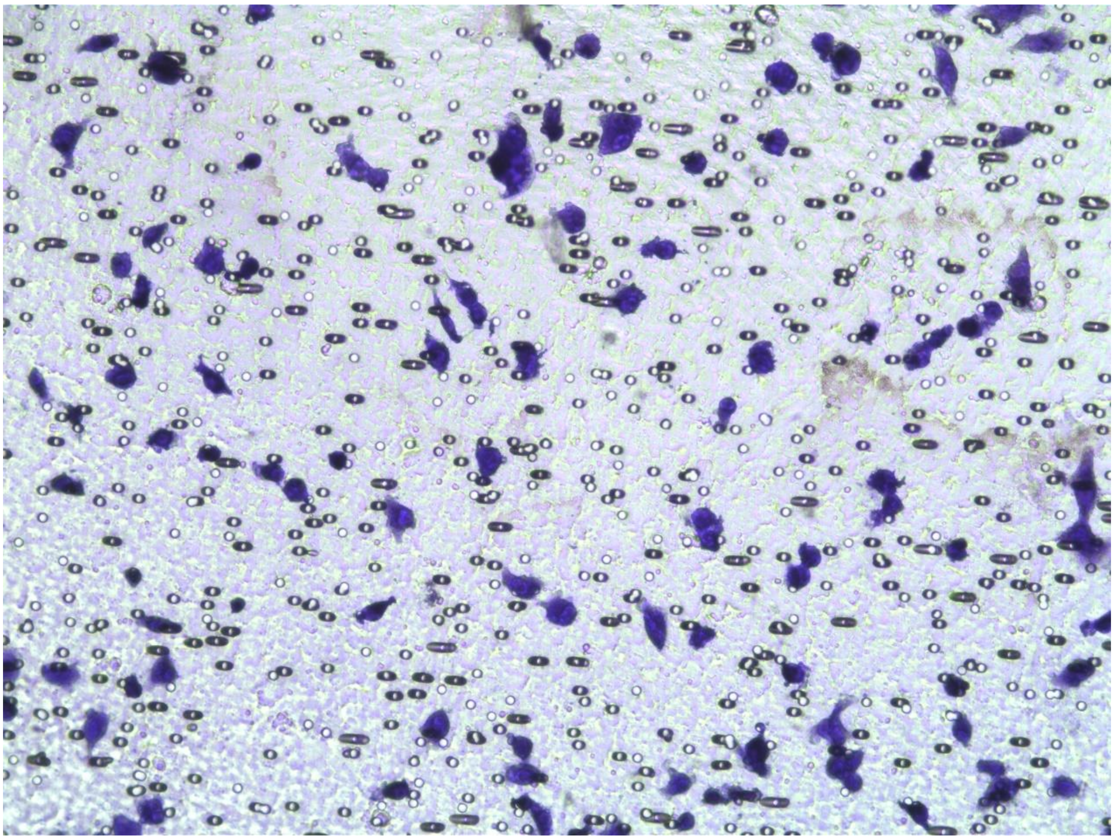

MG63 shNC+vector 200-3

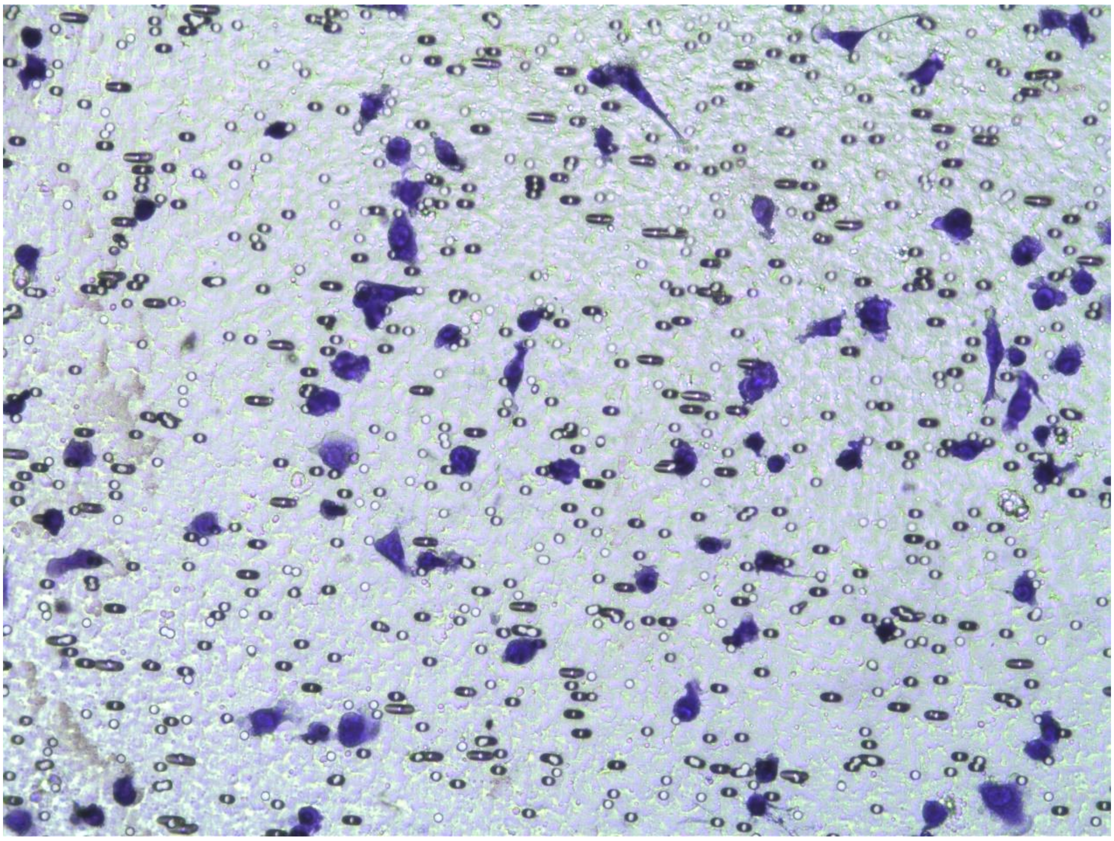

MG63 shNC+vector 200-4

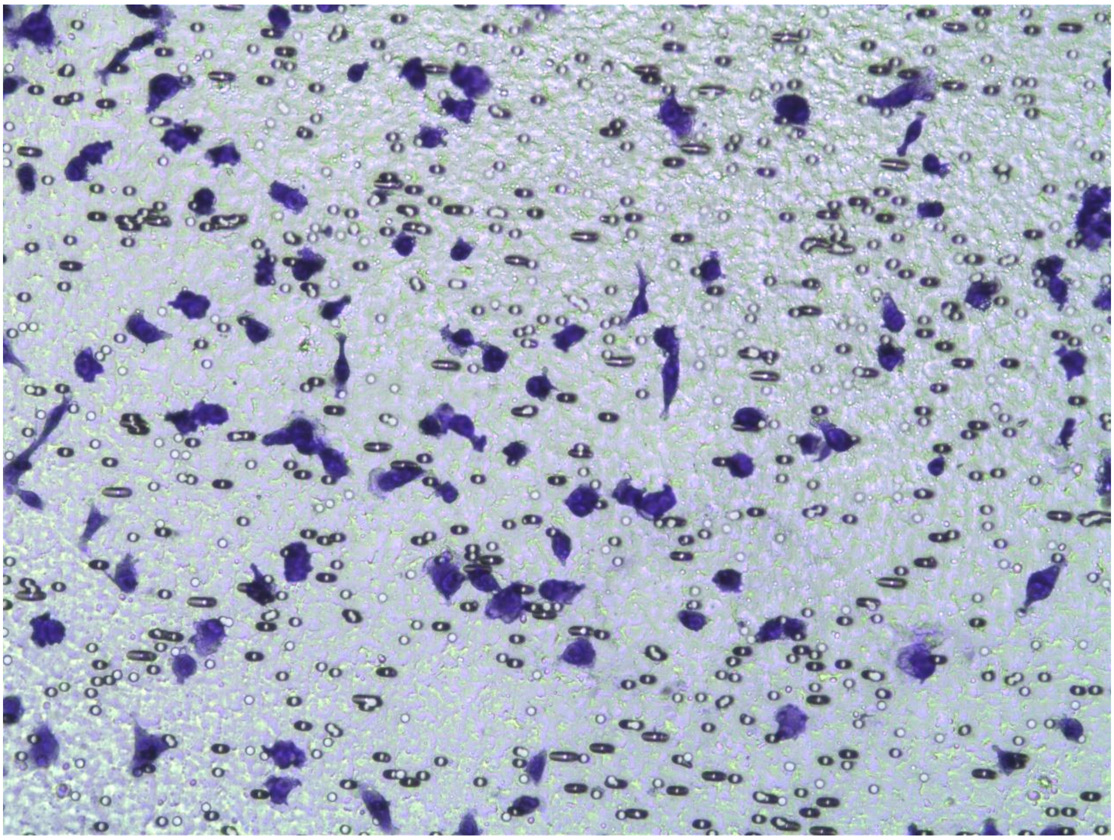

MG63 shNC+vector 200-5

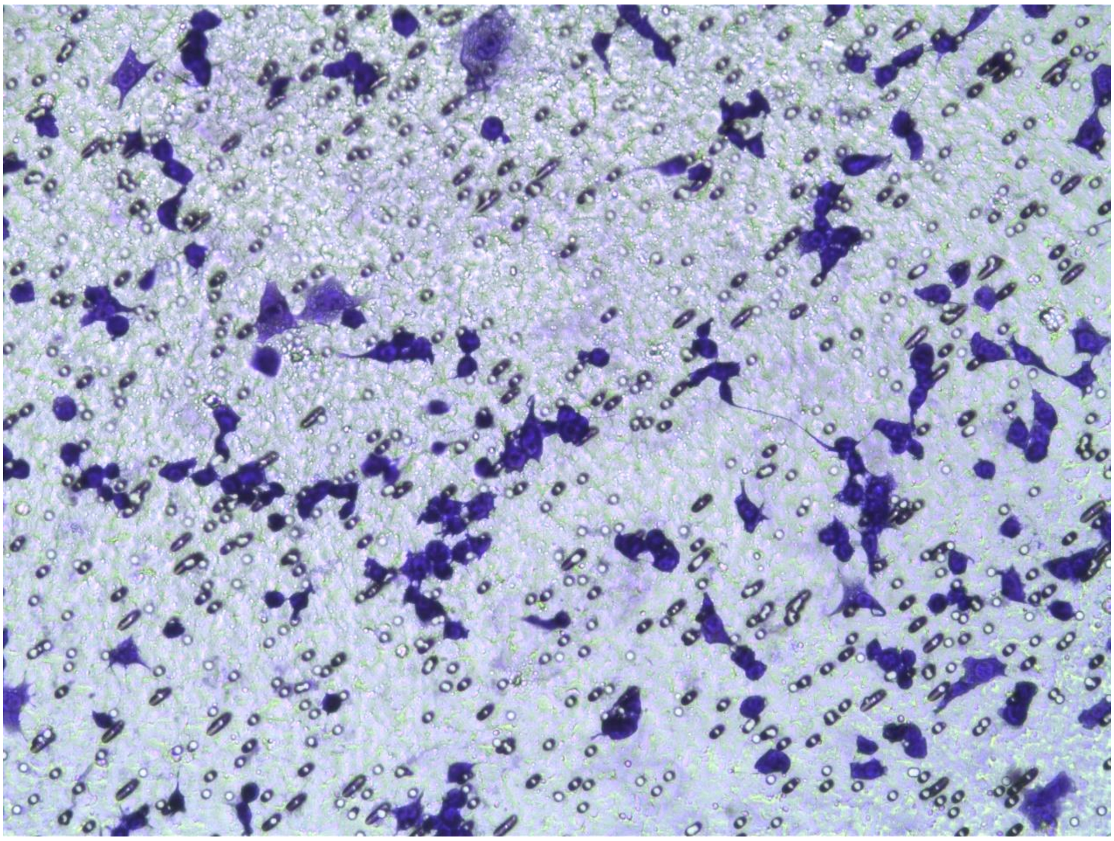

MG63 shS100A16+OEANXA2 200-1

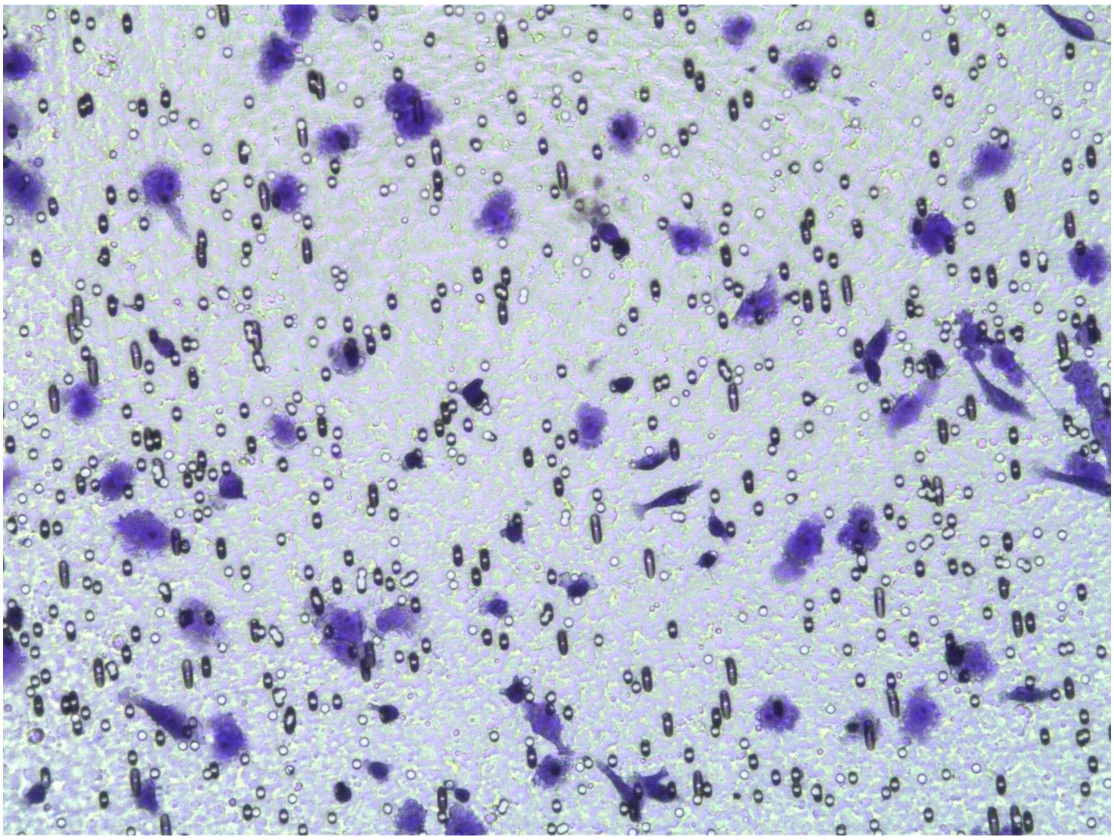

MG63 shS100A16+OEANXA2 200-2

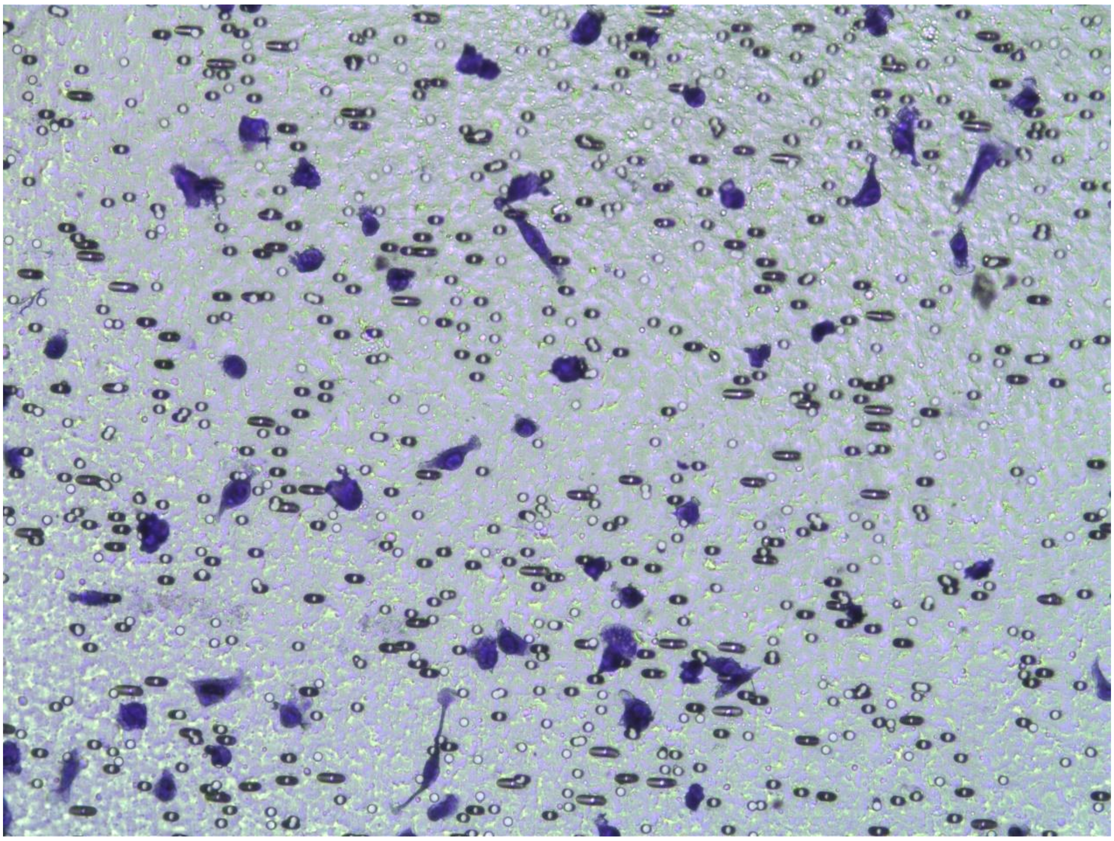

MG63 shS100A16+OEANXA2 200-3

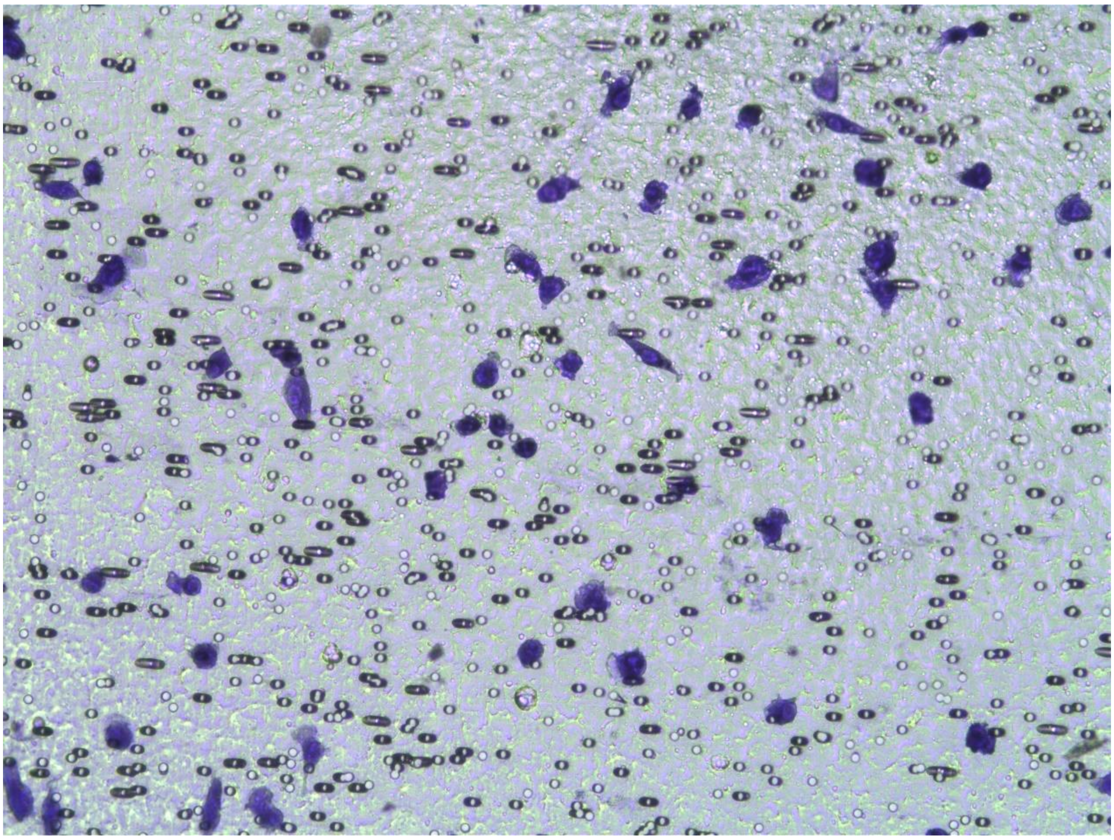

MG63 shS100A16+OEANXA2 200-4

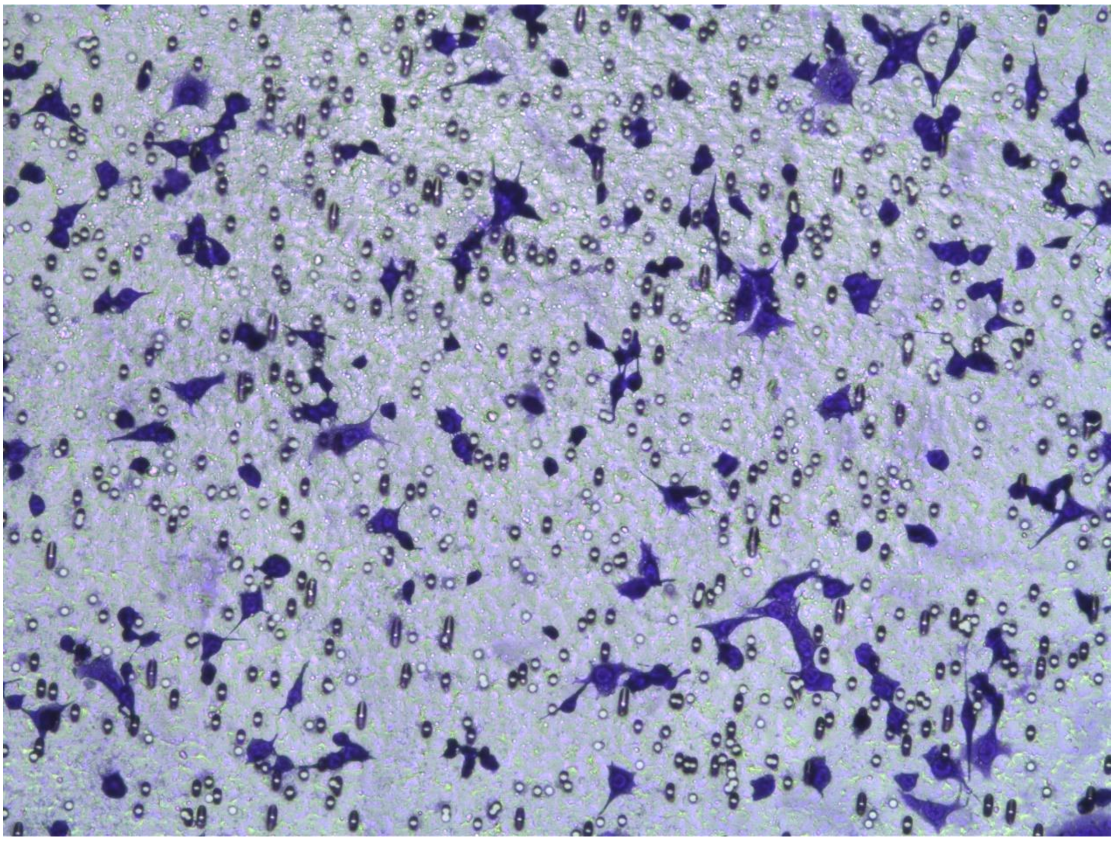

MG63 shS100A16+OEANXA2 200-5

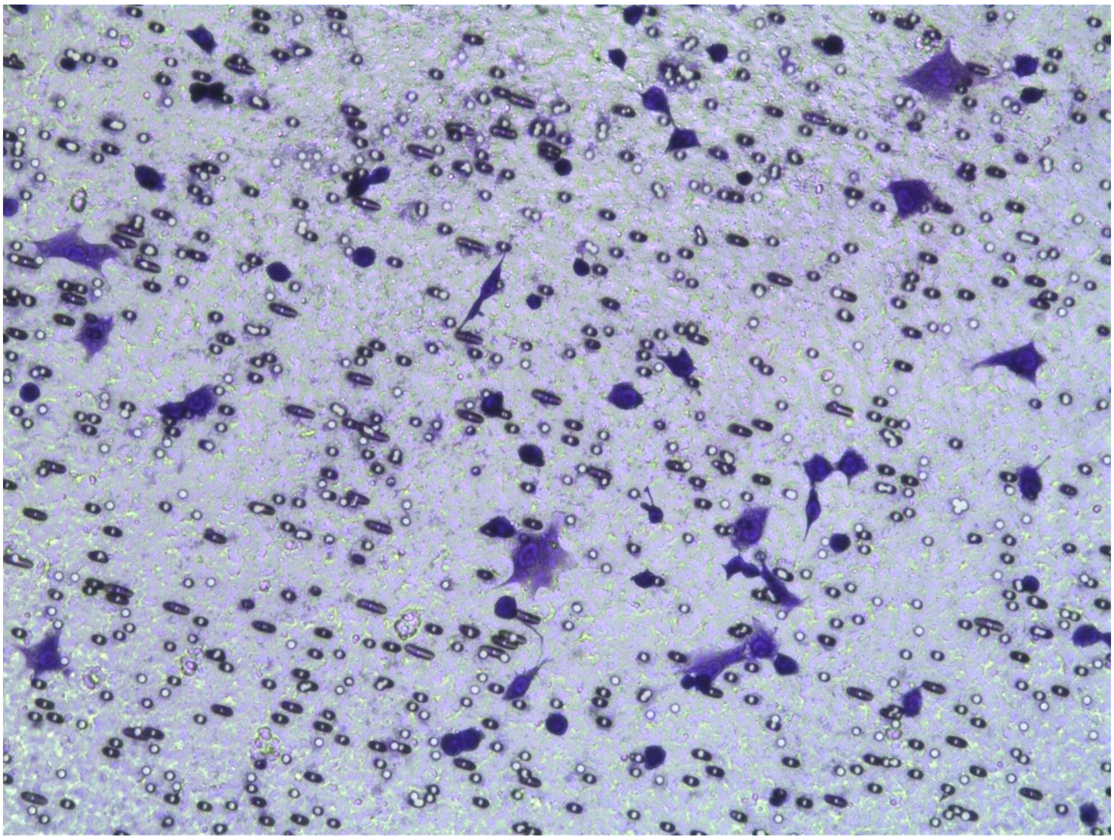

MG63 shS100A16+VECTOR 200-1

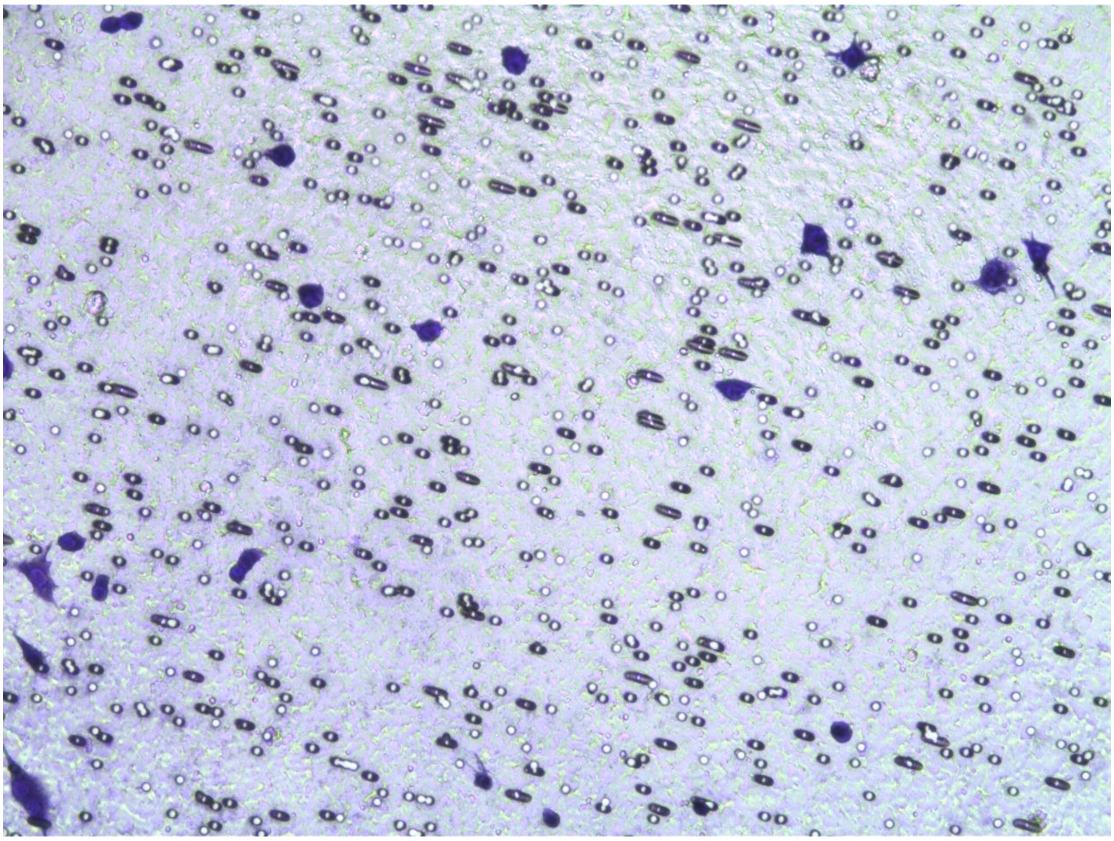

MG63 shS100A16+VECTOR 200-2

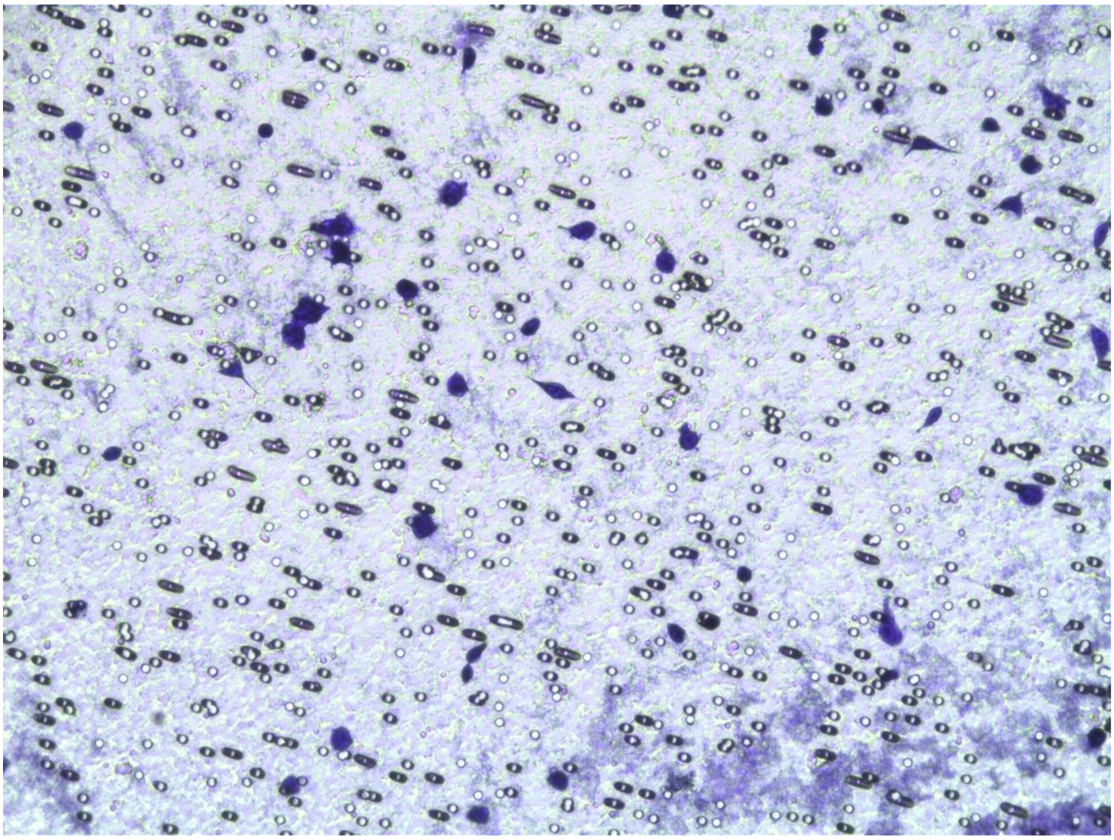

MG63 shS100A16+VECTOR 200-3

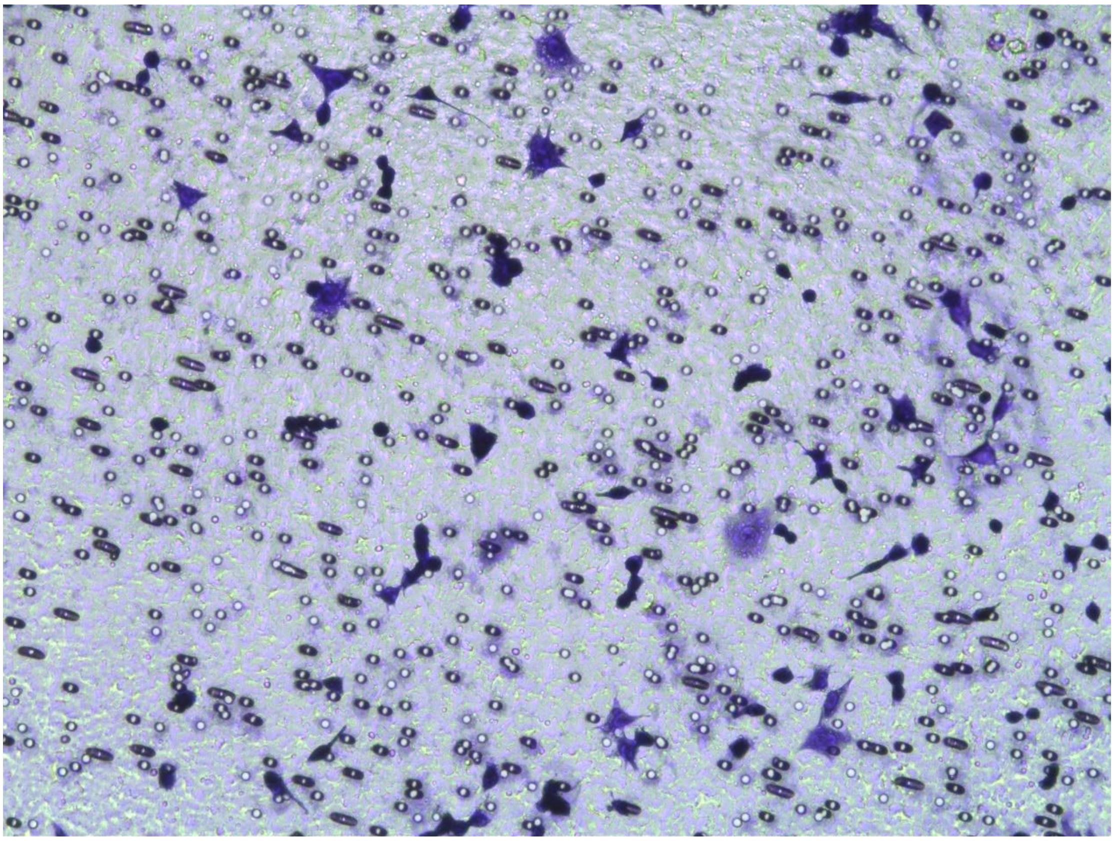

MG63 shS100A16+VECTOR 200-4

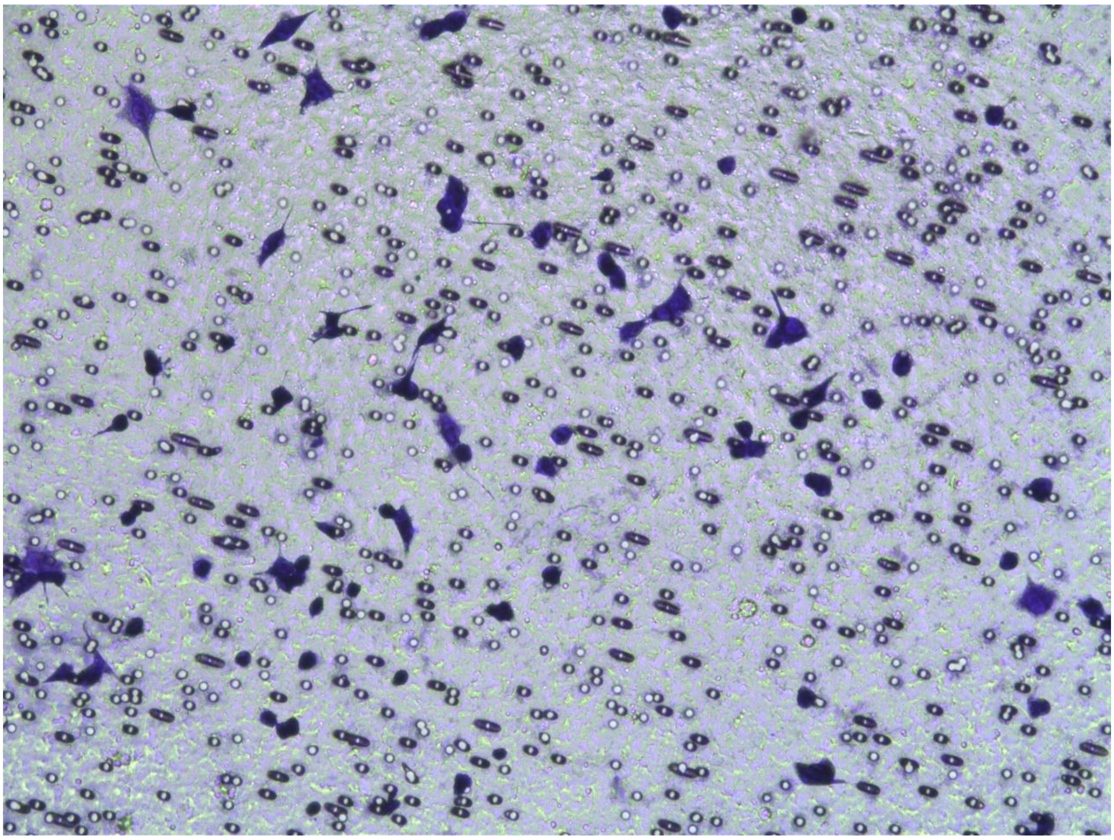

MG63 shS100A16+VECTOR 200-5

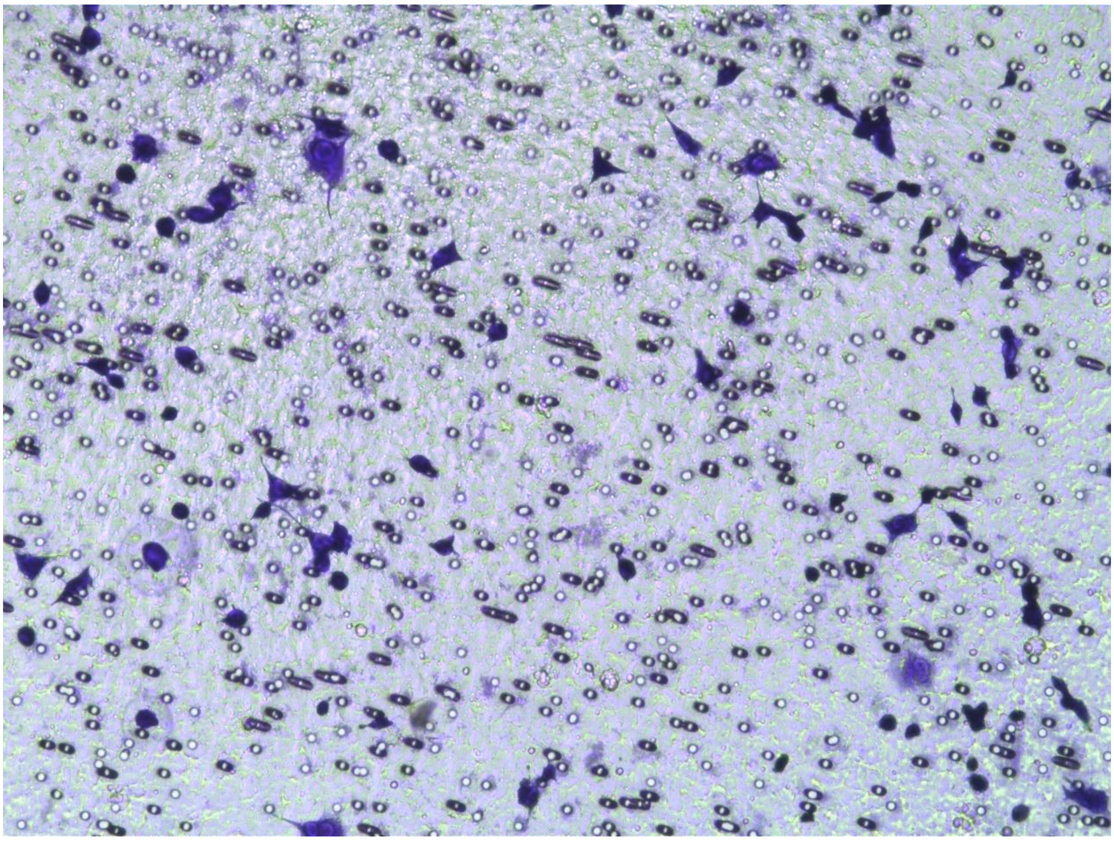

U20S shNC+OEANXA2 200-1

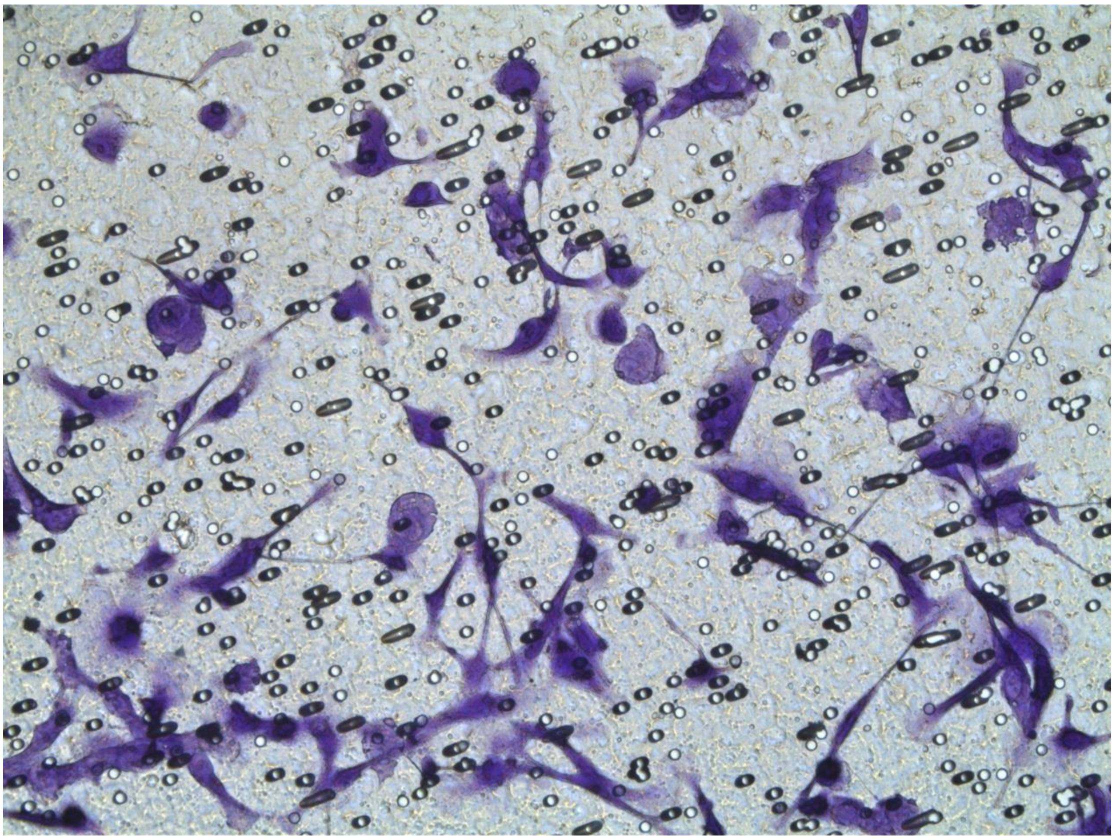

U20S shNC+OEANXA2 200-2

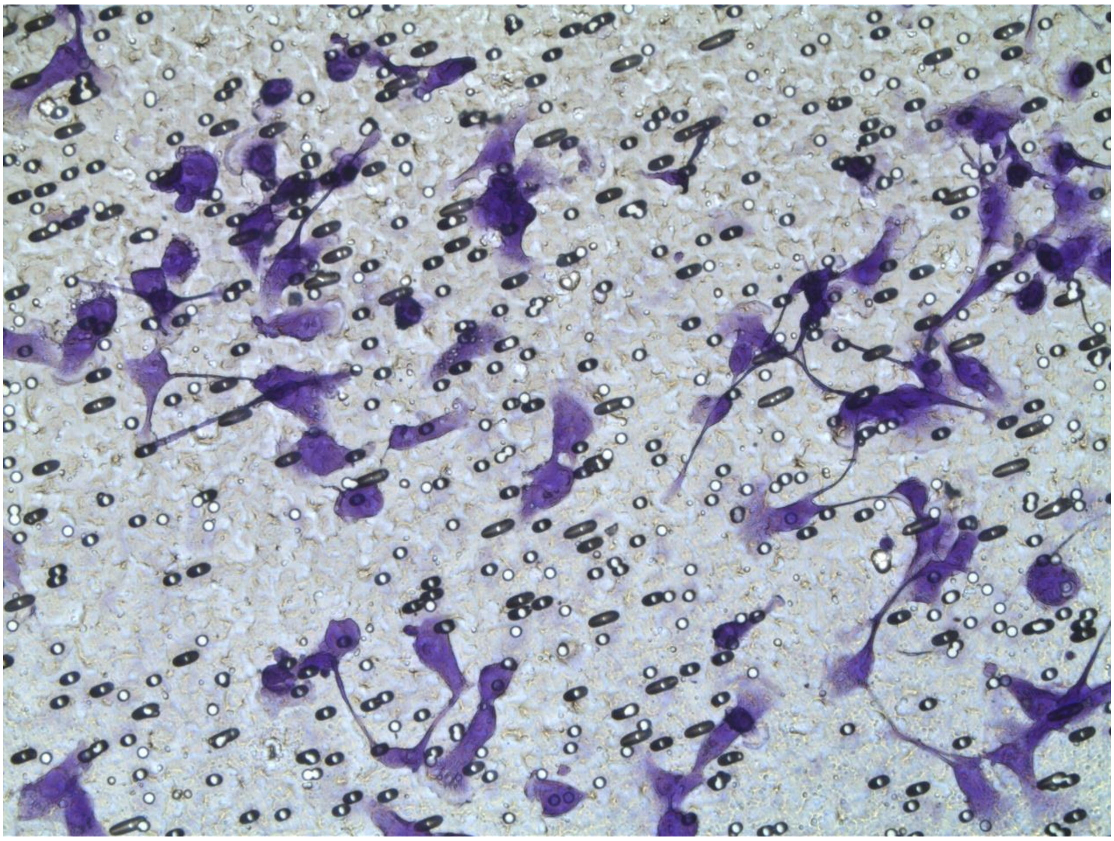

U20S shNC+OEANXA2 200-3

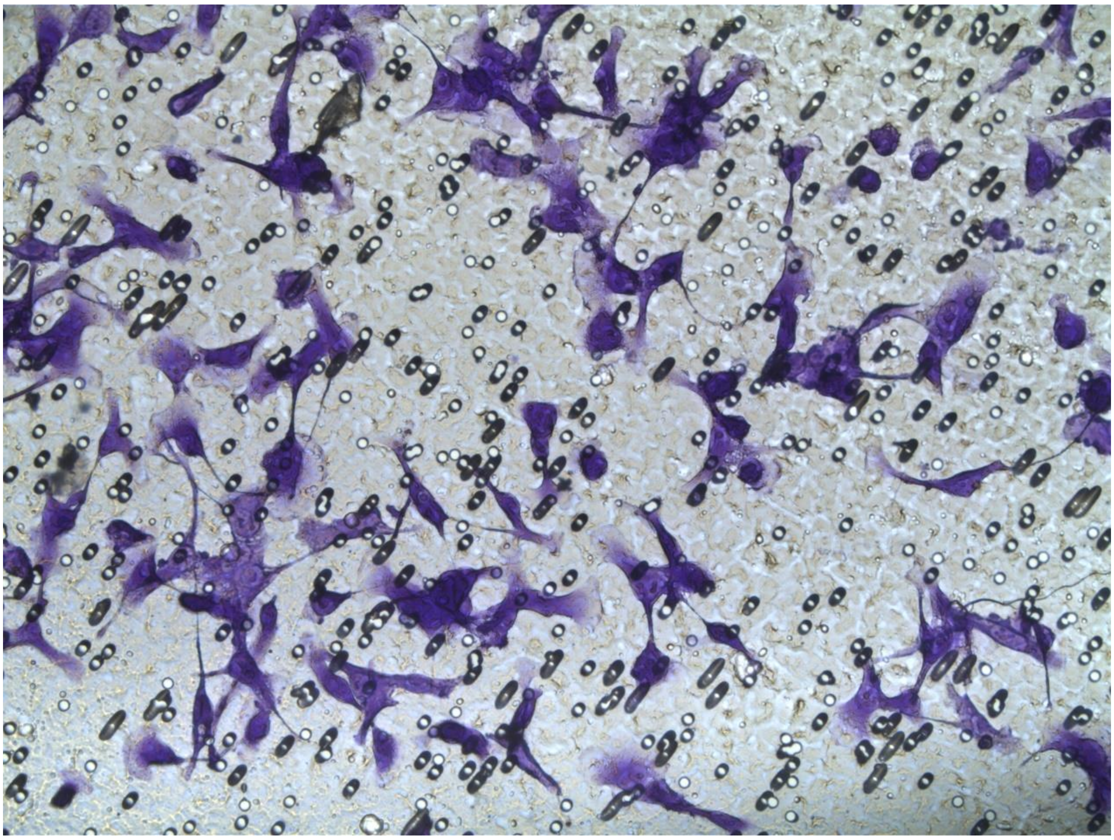

U20S shNC+OEANXA2 200-4

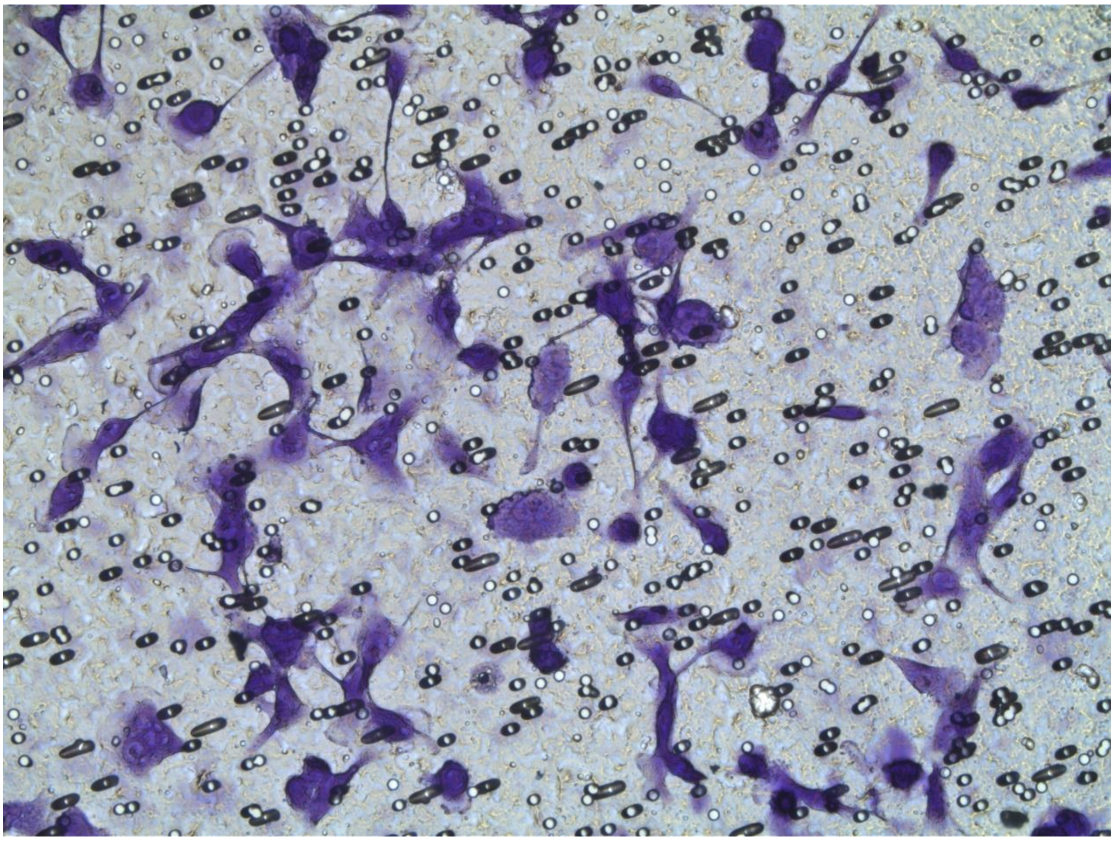

U20S shNC+OEANXA2 200-5

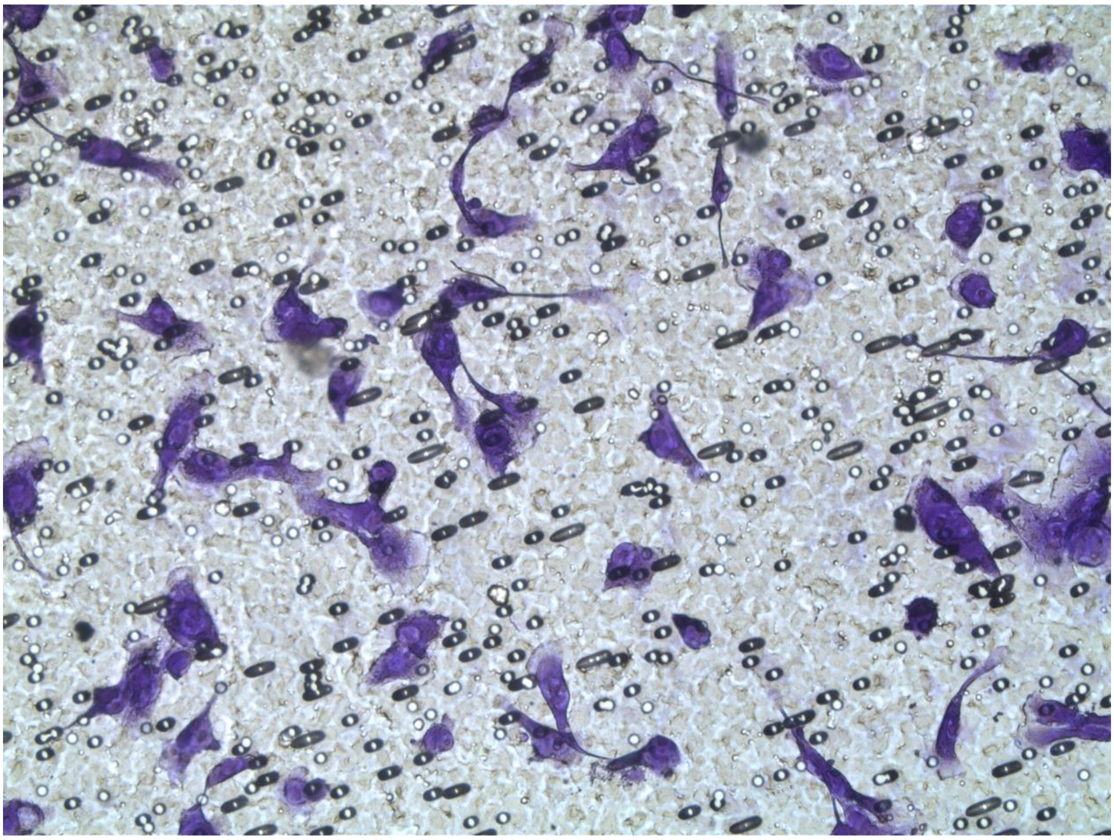

U20S shNC+VECTOR 200-1

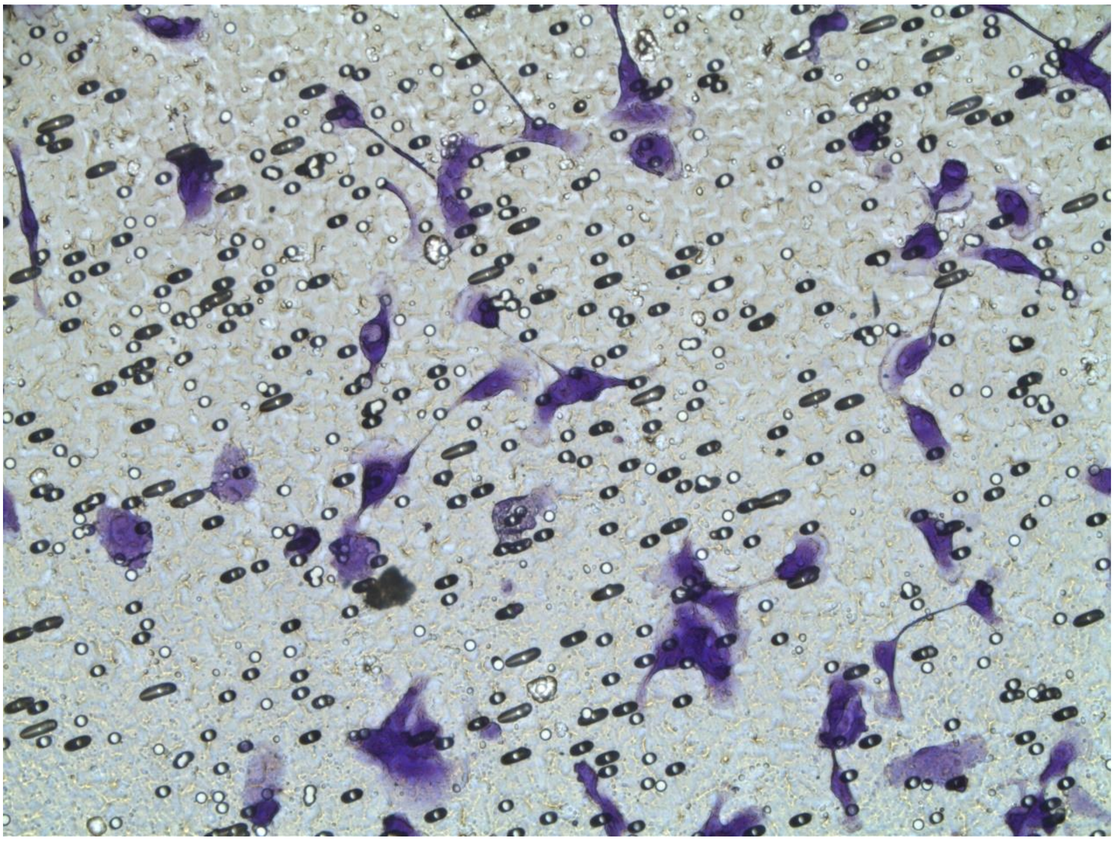

U20S shNC+VECTOR 200-2

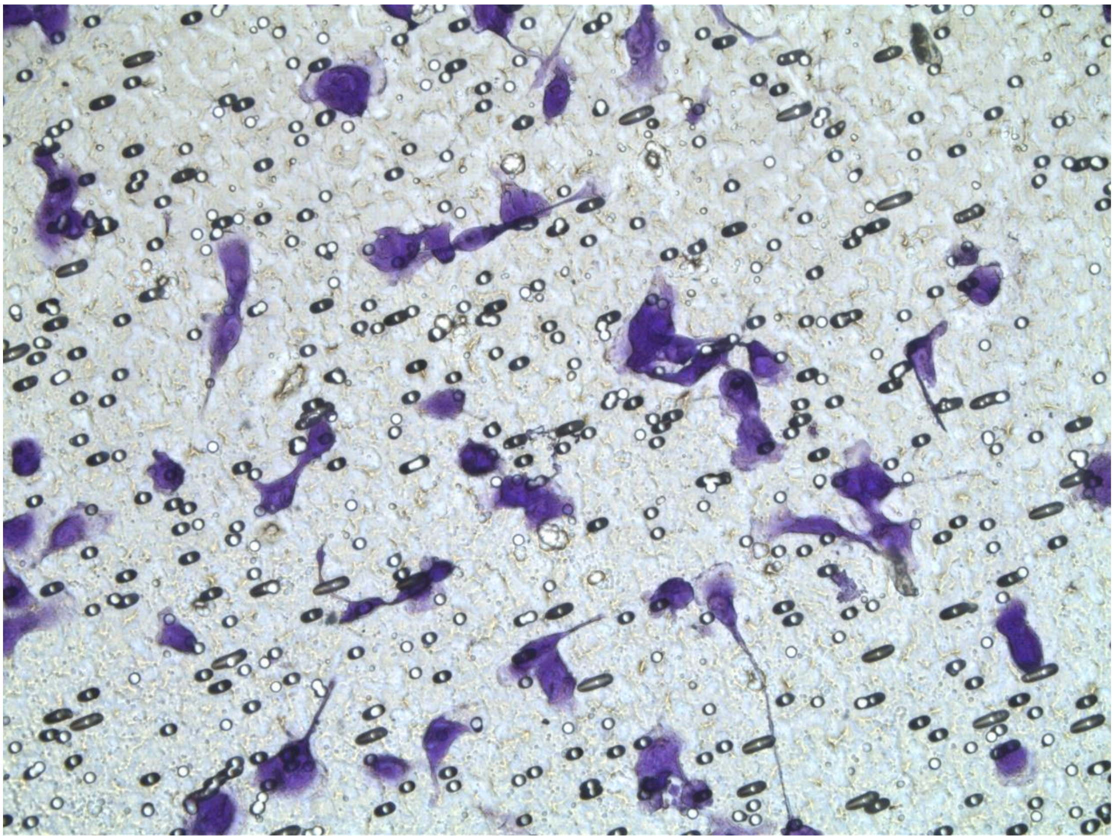

U20S shNC+VECTOR 200-3

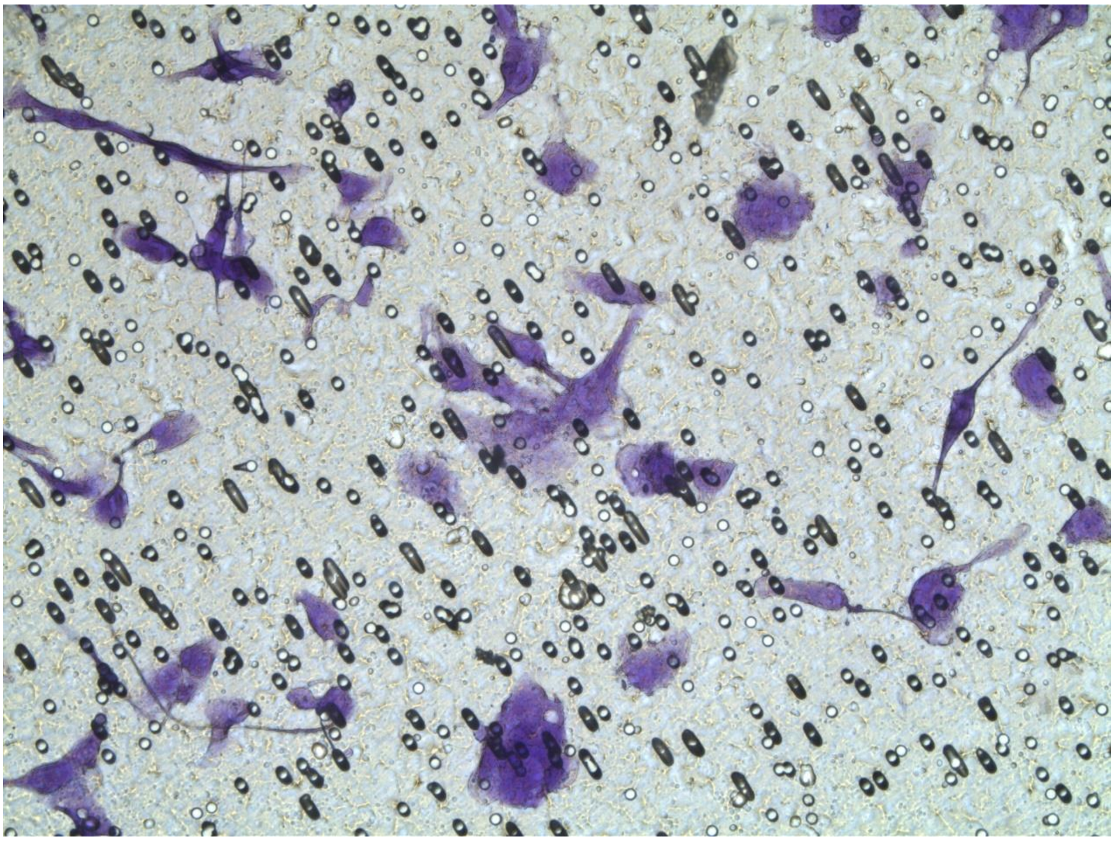

U20S shNC+VECTOR 200-4

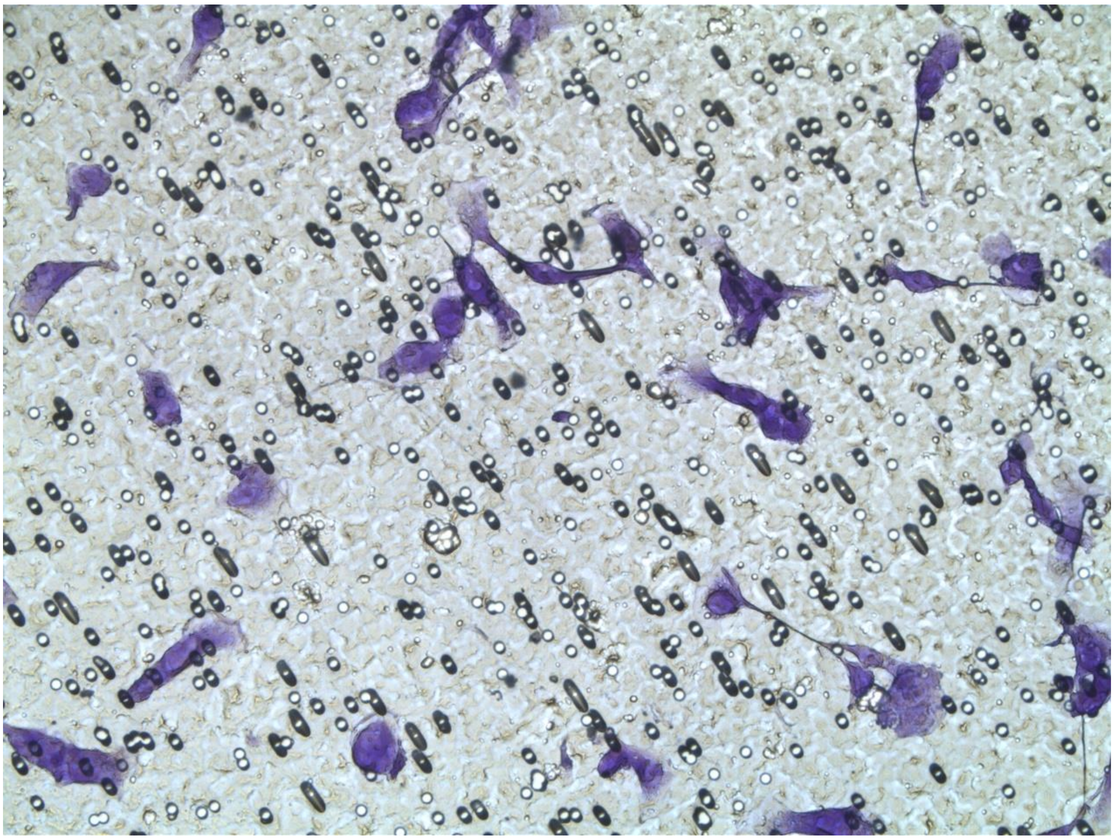

U20S shNC+VECTOR 200-5

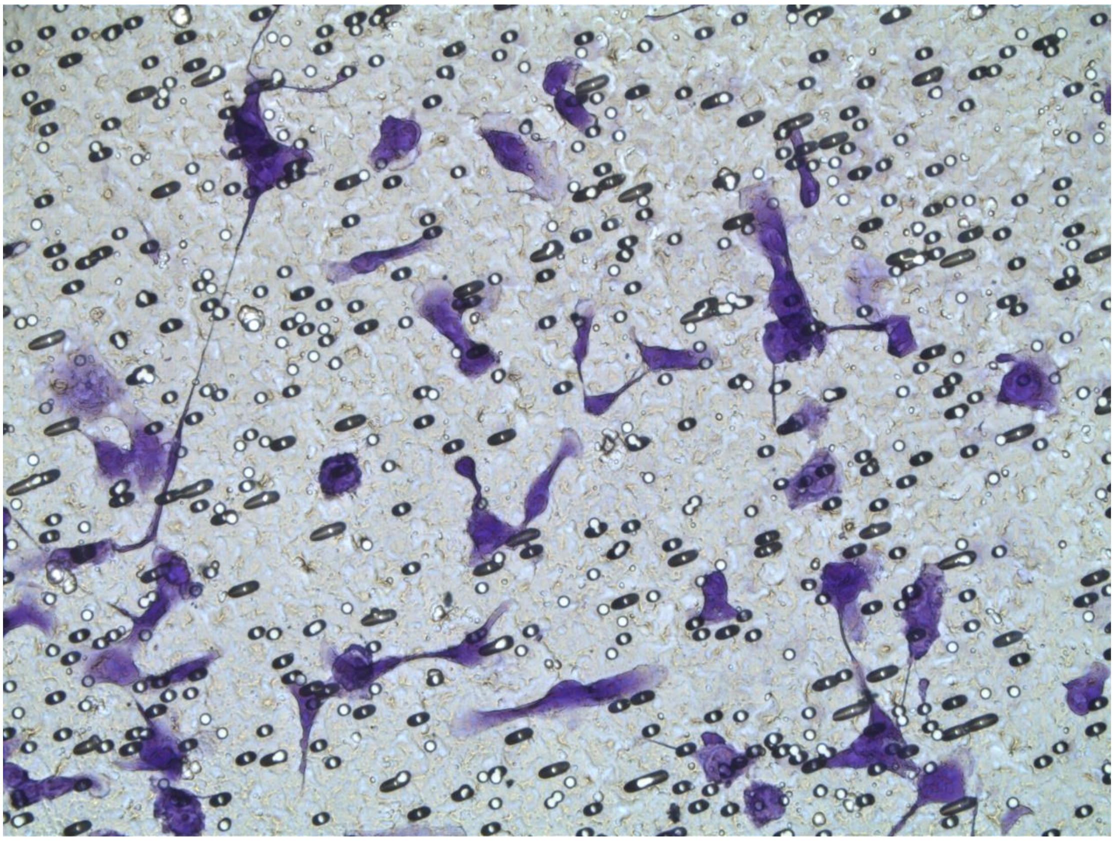

U20S shS100A8+OEANXA2 200-1

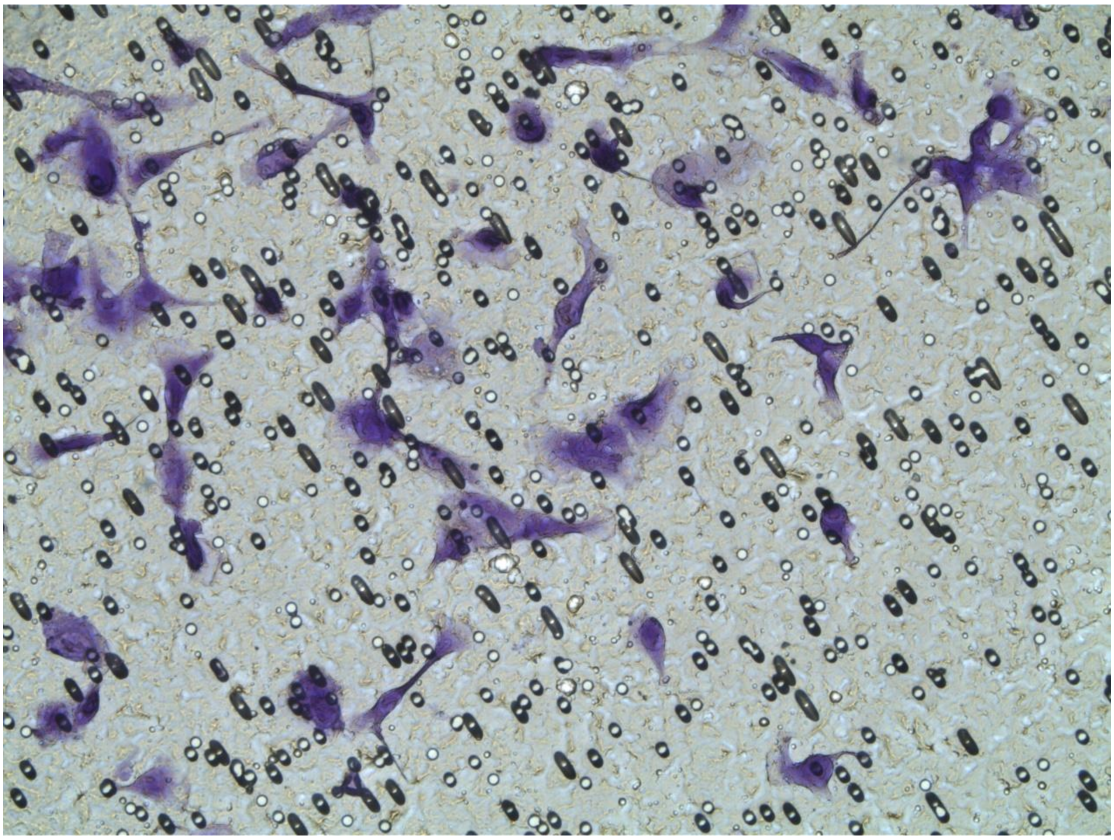

U20S shS100A8+OEANXA2 200-2

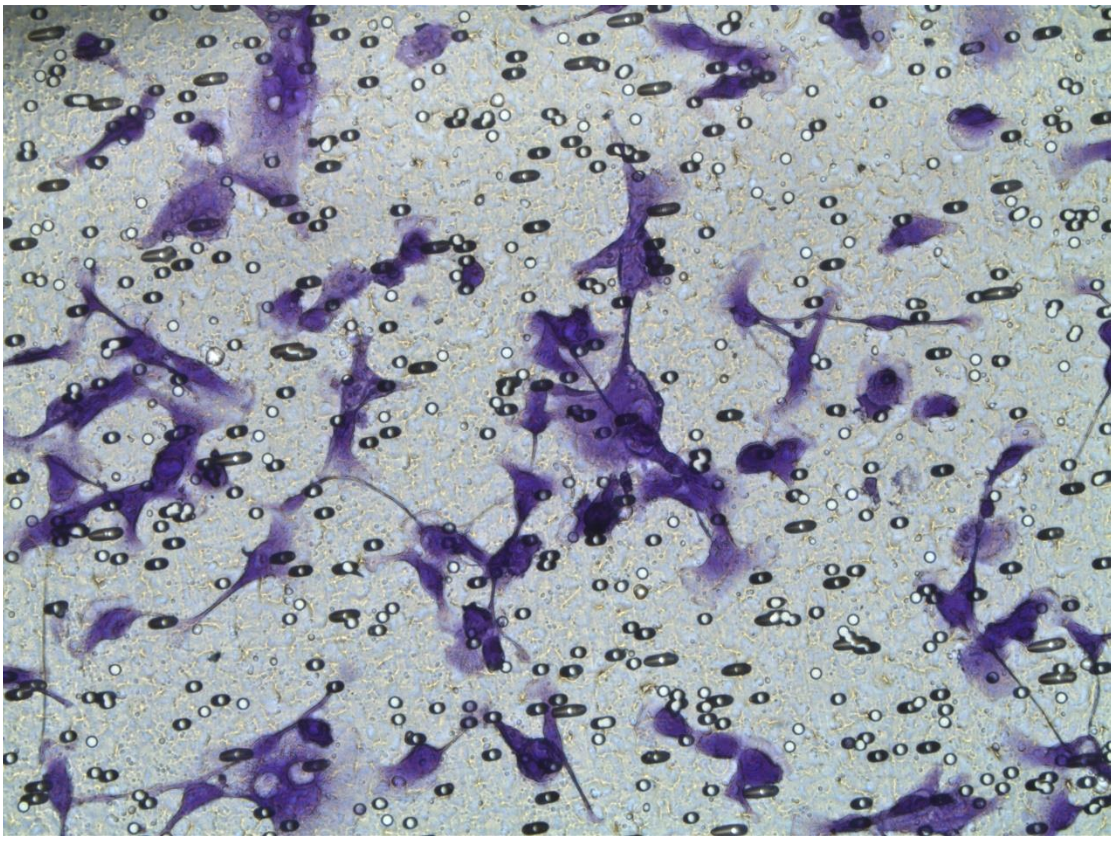

U20S shS100A8+OEANXA2 200-3

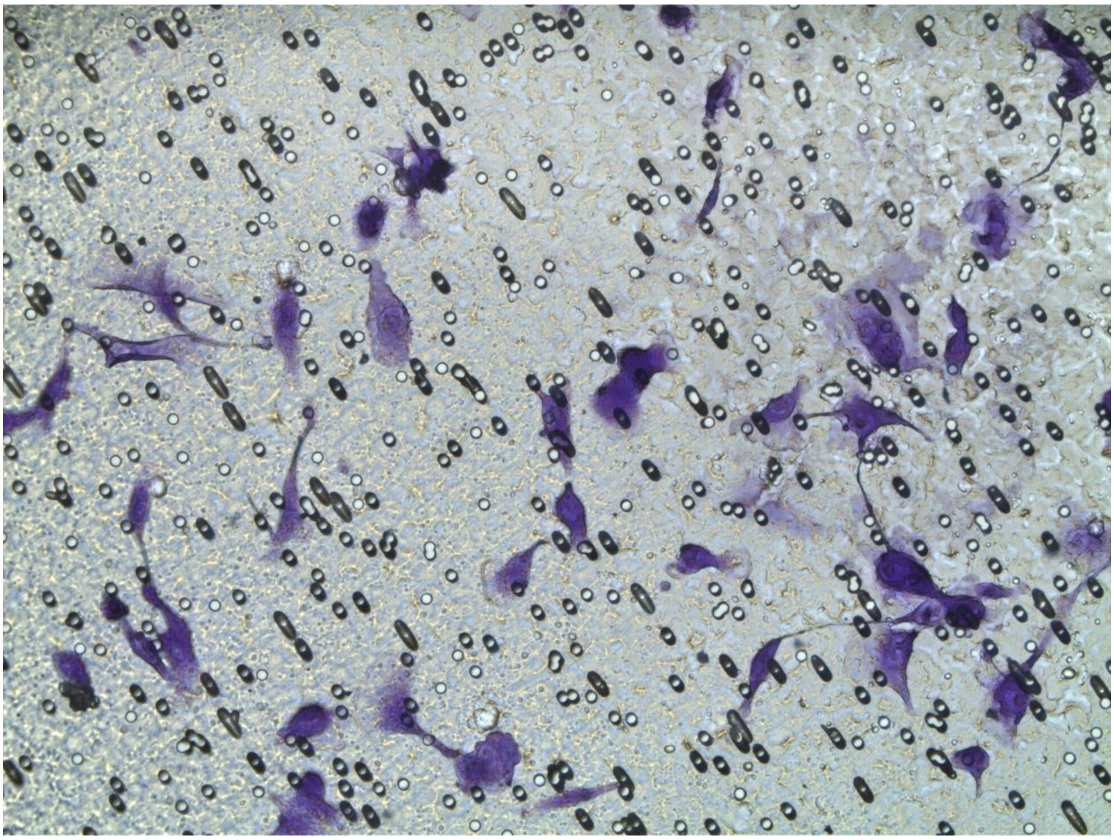

U20S shS100A8+OEANXA2 200-4

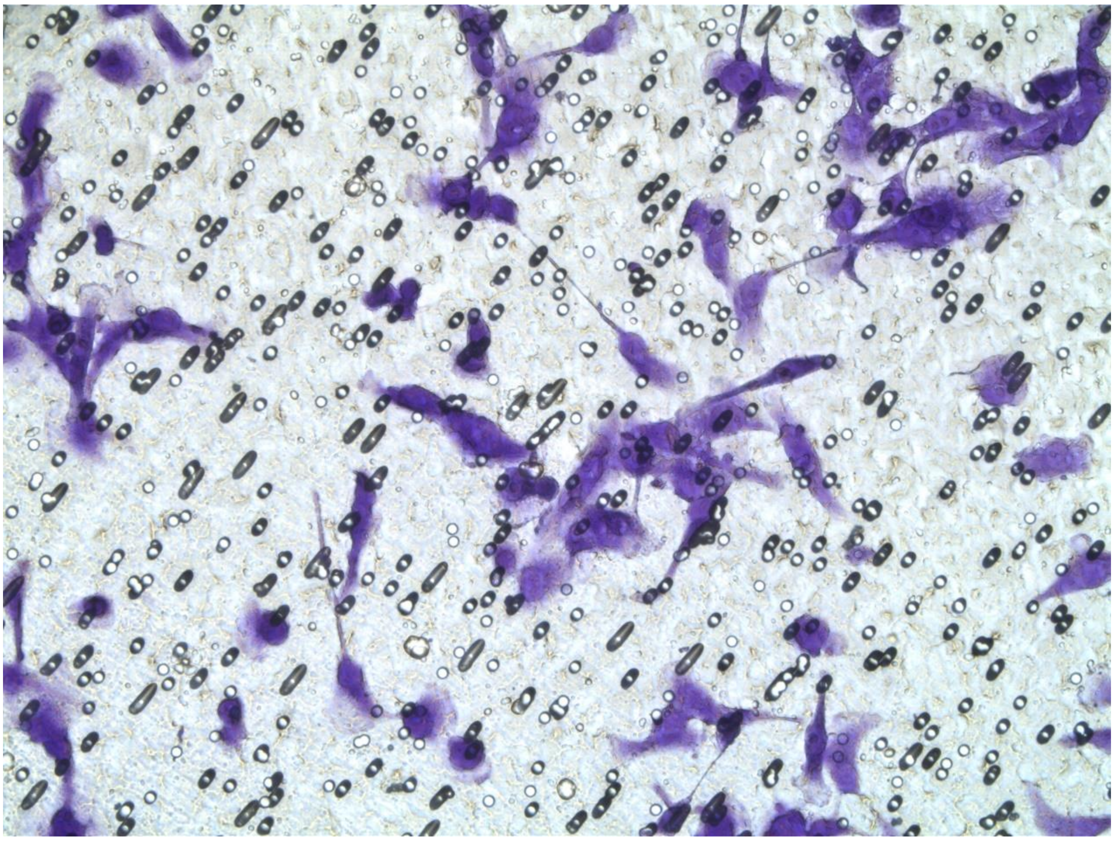

U20S shS100A8+OEANXA2 200-5

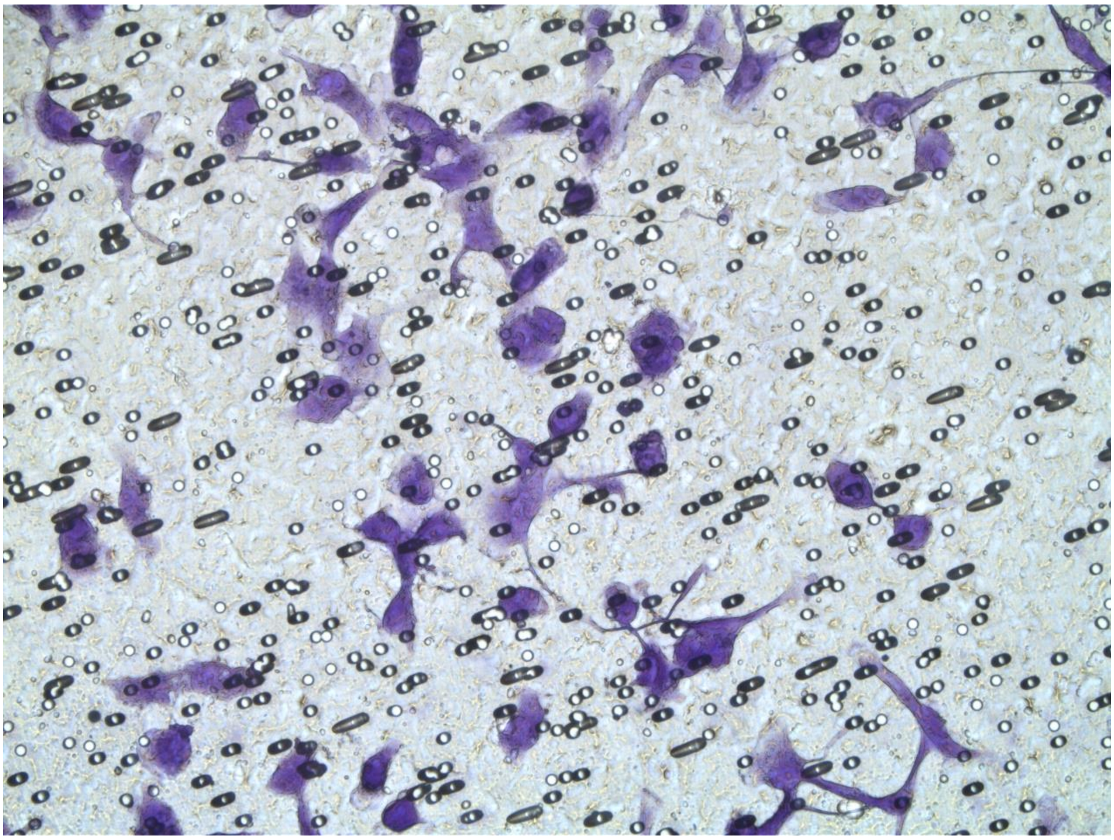

U20S shS100A16+VECTOR 200-1

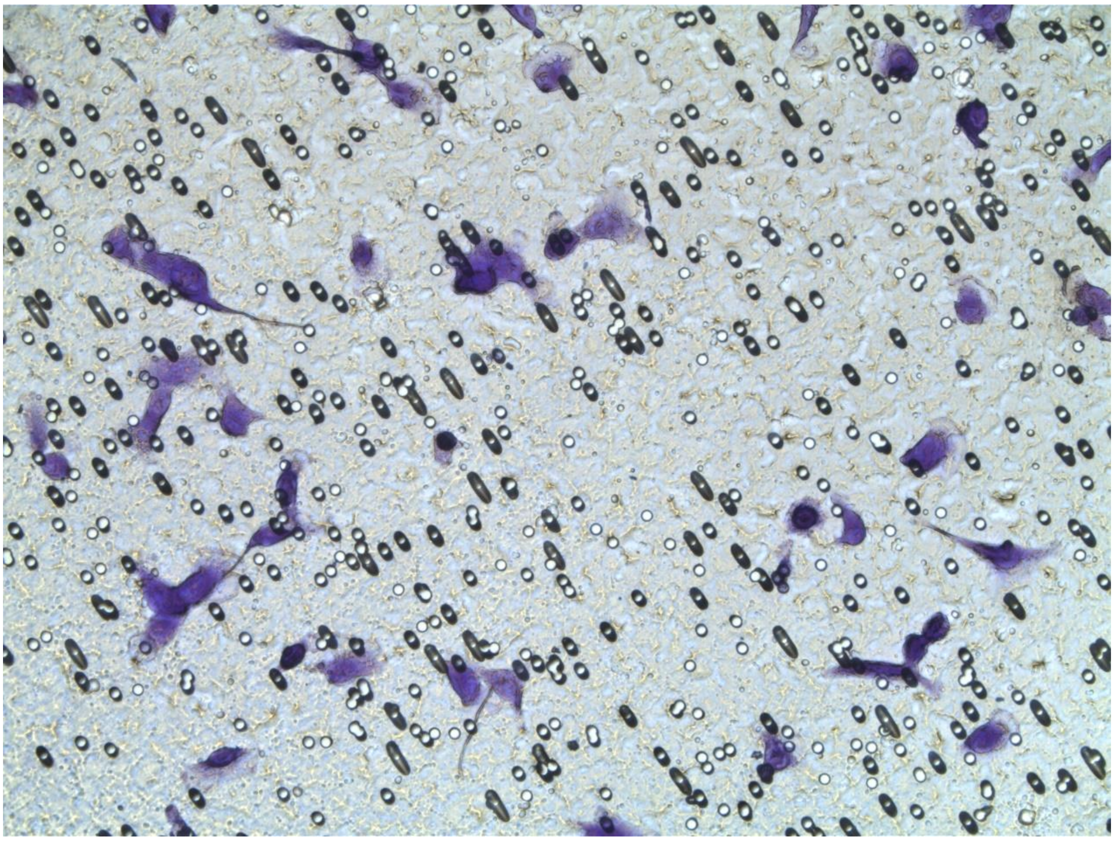

U20S shS100A16+VECTOR 200-2

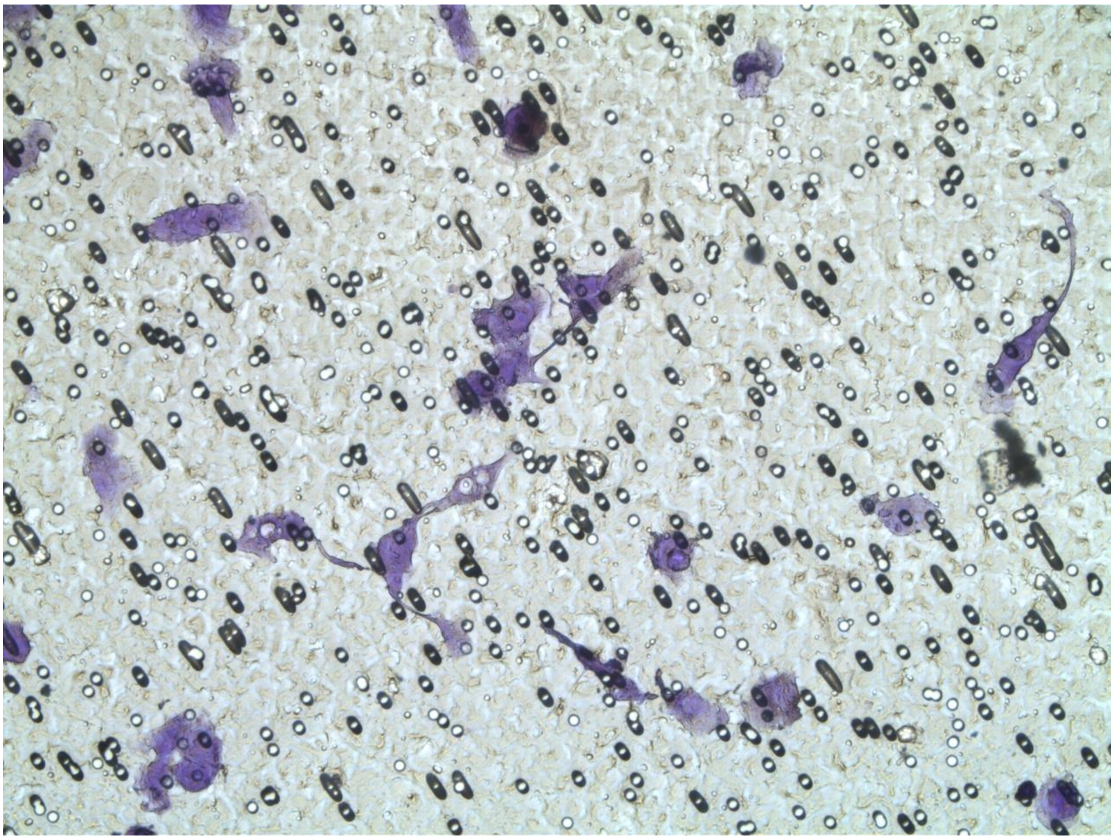

U20S shS100A16+VECTOR 200-3

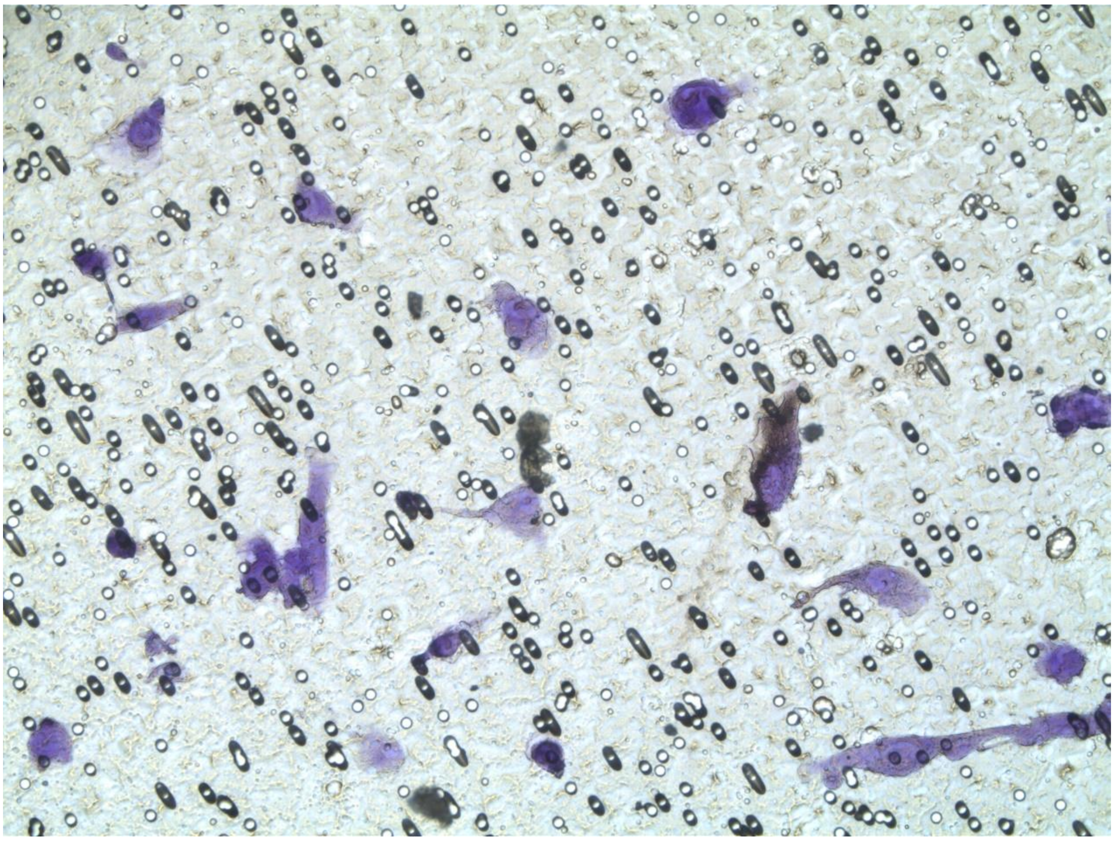

U20S shS100A16+VECTOR 200-4

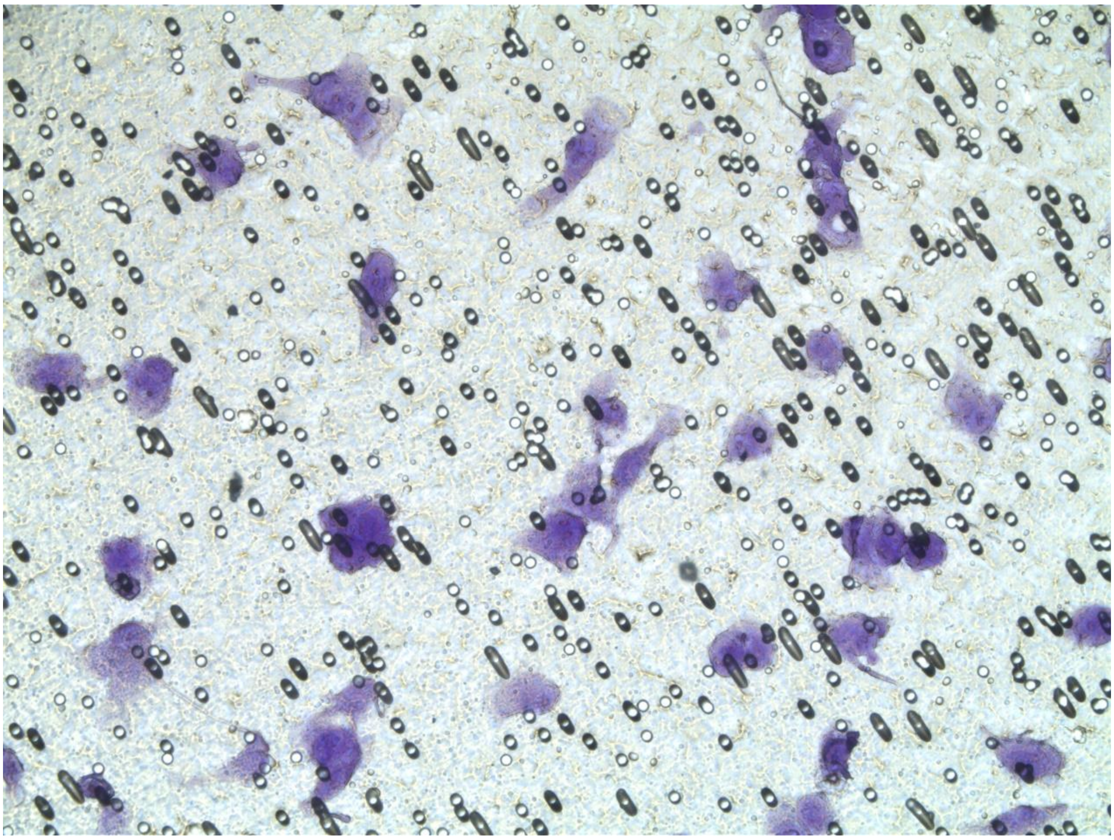

U20S shS100A16+VECTOR 200-5

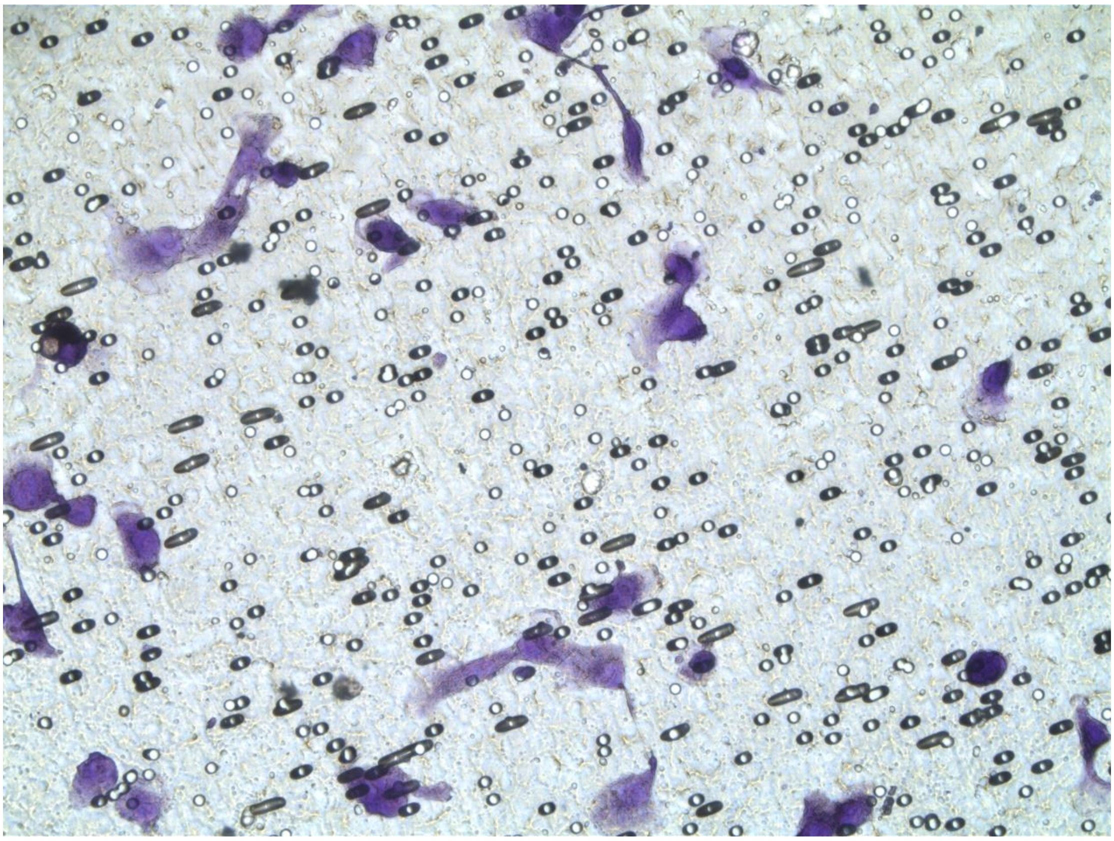

Supplement: Supplementary file 1 — Supplementary Material 1 [file 41598_2025_5293_MOESM1_ESM.pdf]
